# PCIP-seq: simultaneous sequencing of integrated viral genomes and their insertion sites with long reads

**DOI:** 10.1186/s13059-021-02307-0

**Published:** 2021-04-06

**Authors:** Maria Artesi, Vincent Hahaut, Basiel Cole, Laurens Lambrechts, Fereshteh Ashrafi, Ambroise Marçais, Olivier Hermine, Philip Griebel, Natasa Arsic, Frank van der Meer, Arsène Burny, Dominique Bron, Elettra Bianchi, Philippe Delvenne, Vincent Bours, Carole Charlier, Michel Georges, Linos Vandekerckhove, Anne Van den Broeke, Keith Durkin

**Affiliations:** 1 Unit of Animal Genomics, GIGA, Université de Liège (ULiège), Avenue de l’Hôpital 11, 4000 Liège, Belgium; 2 Laboratory of Experimental Hematology, Institut Jules Bordet, Université Libre de Bruxelles (ULB), Boulevard de Waterloo 121, 1000 Brussels, Belgium; 3 Laboratory of Human Genetics, GIGA, Université de Liège (ULiège), Avenue de l’Hôpital 11, 4000 Liège, Belgium; 4 HIV Cure Research Center, Department of Internal Medicine and Pediatrics, Ghent University Hospital and Ghent University, 9000 Ghent, Belgium; 5 BioBix, Department of Data Analysis and Mathematical Modelling, Faculty of Bioscience Engineering, Ghent University, Ghent, Belgium; 6 Department of Animal Science, Faculty of Agriculture, Ferdowsi University of Mashhad, Mashhad, Iran; 7 Service d’hématologie, Hôpital Universitaire Necker, Université René Descartes, Assistance Publique Hôpitaux de Paris, Paris, France; 8 Vaccine and Infectious Disease Organization, VIDO-Intervac, University of Saskatchewan, 120 Veterinary Road, Saskatoon, S7N 5E3 Canada; 9 Faculty of Veterinary Medicine: Ecosystem and Public Health, Calgary, AB Canada; 10 Department of Pathology, University Hospital (CHU), University of Liège, Liège, Belgium; 11 Department of Human Genetics, University Hospital (CHU), University of Liège, Liège, Belgium

**Keywords:** Viral genome, Integration site analysis, Clonal expansion, NGS, Long-read sequencing, Retrovirus, HTLV-1, BLV, HIV, HPV

## Abstract

The integration of a viral genome into the host genome has a major impact on the trajectory of the infected cell. Integration location and variation within the associated viral genome can influence both clonal expansion and persistence of infected cells. Methods based on short-read sequencing can identify viral insertion sites, but the sequence of the viral genomes within remains unobserved. We develop PCIP-seq, a method that leverages long reads to identify insertion sites and sequence their associated viral genome. We apply the technique to exogenous retroviruses HTLV-1, BLV, and HIV-1, endogenous retroviruses, and human papillomavirus.

**Supplementary Information:**

The online version contains supplementary material available at 10.1186/s13059-021-02307-0.

## Background

The integration of viral DNA into the host genome is a defining feature of the retroviral life cycle, irreversibly linking provirus and cell. This intimate association facilitates viral persistence and replication in somatic cells and with integration into germ cells bequeaths the provirus to subsequent generations. Considerable effort has been expended to understand patterns of proviral integration, both from a basic virology stand point and due to the use of retroviral vectors in gene therapy [1]. The application of next-generation sequencing (NGS) over the last ~ 10 years has had a dramatic impact on our ability to explore the landscape of retroviral integration for both exogenous and endogenous retroviruses. Methods based on ligation mediated PCR and Illumina sequencing have facilitated the identification of hundreds of thousands of insertion sites in exogenous viruses such as human T cell leukemia virus-1 (HTLV-1) [2] and human immunodeficiency virus (HIV-1) [3–6]. These techniques have shown that in HTLV-1 [2], bovine leukemia virus (BLV) [7], and avian leukosis virus (ALV) [8] integration sites are not random, pointing to clonal selection. In HIV-1, it has also become apparent that provirus integration can drive clonal expansion [3, 4, 6, 9], magnifying the HIV-1 reservoir and placing a major road block in the way of a complete cure.

Current methods based on short-read sequencing identify the viral insertion point, but the proviral genome associated with that integration site is largely unexplored. Whether variation in the provirus influences the fate of the clone remains difficult to investigate. Work in HTLV-1 points to the potential importance of such variation as studies using long-range PCR [10] and biotin capture probes [11] have shown that defective proviruses are selected for in both HTLV-1 induced adult T cell leukemia (ATL) and asymptomatic HTLV-1 carriers. Methods such as Full-Length Individual Proviral Sequencing (FLIPS) have been developed to identify functional proviruses [12], but without identifying the provirus integration site. More recently, matched integration site and proviral sequencing (MIP-Seq) and multiple-displacement amplification-single-genome sequencing (MDA-SGS) allowed the sequence of individual proviruses to be linked to the integration site in the genome [6, 13]. However, these methods rely on whole genome amplification of isolated HIV-1 genomes, with separate reactions to identify the integration site and sequence the associated provirus [6]. As a result, these methods are quite labor intensive, limiting the number of proviruses one can reasonably interrogate.

Retroviruses are primarily associated with the diseases they provoke through the infection of somatic cells. Over the course of evolutionary time they have also played a major role in shaping the genome. Retroviral invasion of the germ line has occurred multiple times, resulting in the remarkable fact that endogenous retrovirus (ERV)-like elements comprise a larger proportion of the human genome (8%) than protein coding sequences (~ 1.5%) [14]. With the availability of multiple vertebrate genome assemblies, much of the focus has been on comparison of ERVs between species. However, single genomes represent a fraction of the variation within a species, prompting some to take a population approach to investigate ERV–host genome variation [15]. While capable of identifying polymorphic ERVs in the population, approaches relying on conventional paired-end libraries and short reads cannot capture the sequence of the provirus beyond the first few hundred bases of the proviral long terminal repeat (LTR), leaving the variation within uncharted.

In contrast to retroviruses, papillomaviruses do not integrate into the host genome as part of their lifecycle. Human papillomavirus (HPV) is usually present in the cell as a multicopy circular episome (~ 8 kb in size); however, in a small fraction of infections, it can integrate into the host genome leading to the dysregulation of the viral oncogenes E6 and E7 [16]. Genome wide profiling of HPV integration sites via capture probes and Illumina sequencing has also identified hotspots of integration indicating that disruption of host genes may also play a role in driving clonal expansion [17]. As a consequence, HPV integration is a risk factor for the development of cervical carcinoma [18]; however, its study is hampered by the unpredictability of the breakpoint sites in the integrated HPV genome. This limits the applicability of approaches based on ligation-mediated PCR and short-read sequencing.

The application of NGS as well as Sanger sequencing before has had a large impact on our understanding of both exogenous and endogenous proviruses. The development of long-read sequencing, linked-read technologies, and associated computational tools [19] have the potential to explore questions inaccessible to short reads. Groups investigating long interspersed nuclear elements-1 (LINE-1) insertions [20] and the koala retrovirus, KoRV [21], have highlighted this potential and described techniques utilizing the Oxford Nanopore and PacBio platforms, to investigate insertion sites and retroelement structure.

To more fully exploit the potential of long reads, we developed Pooled CRISPR Inverse PCR sequencing (PCIP-seq), a method that leverages selective cleavage of circularized DNA fragments carrying proviral DNA with a pool of CRISPR guide RNAs, followed by inverse long-range PCR and multiplexed sequencing on the Oxford Nanopore MinION platform. Using this approach, we can now simultaneously identify the integration site and track clone abundance while also sequencing the provirus inserted at that position. We have successfully applied the technique to the retroviruses HTLV-1, HIV-1, and BLV, endogenous retroviruses in cattle and sheep, and HPV18.

## Results

### Overview of PCIP-seq (pooled CRISPR inverse PCR-sequencing)

**Fig. 1:**
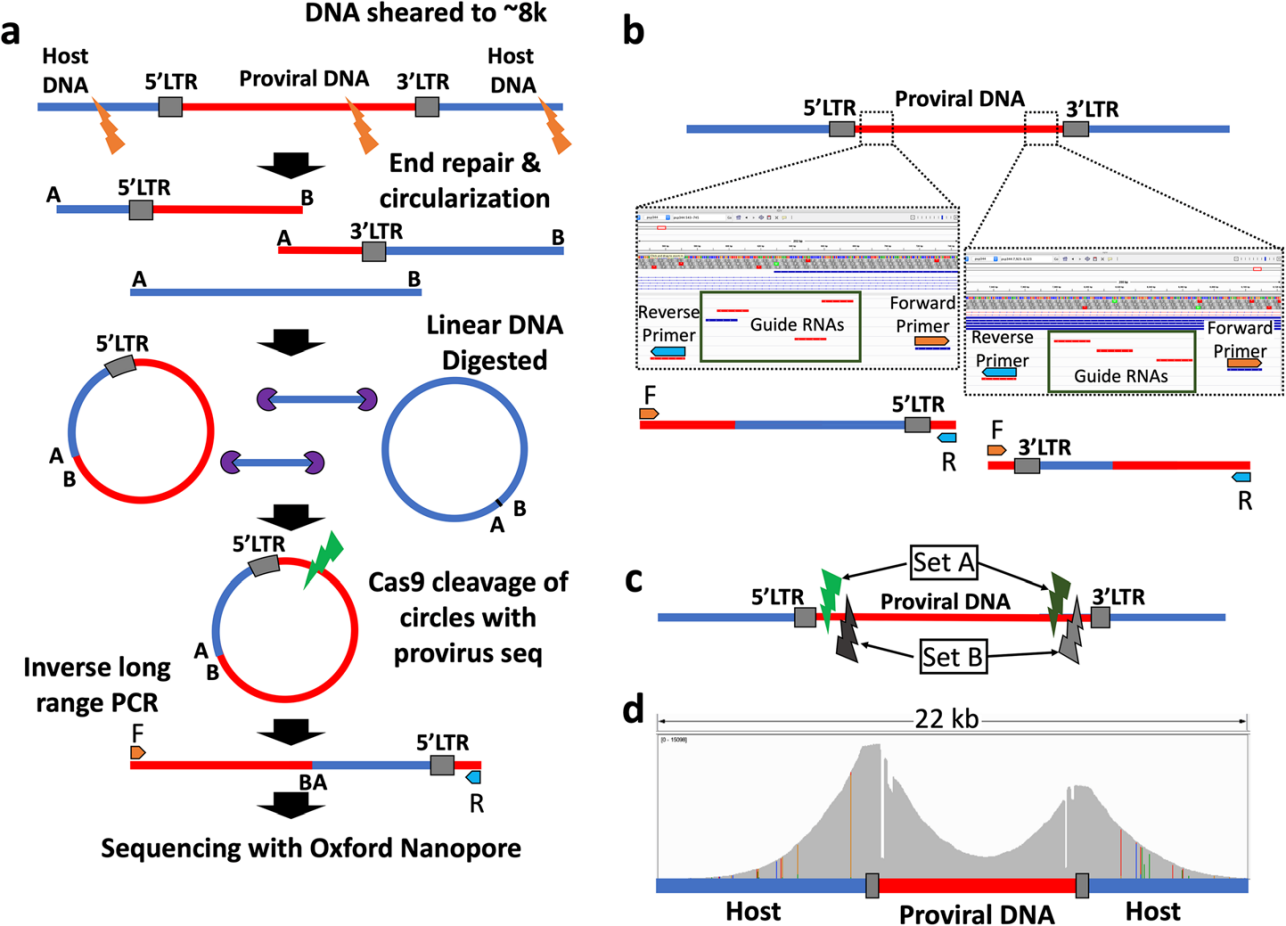
Overview of the PCIP-seq method. **a** Simplified outline of the method. Only 5′ LTR-containing circles and fragments are represented. Detailed outline available in Supplementary Methods. **b** A pool of CRISPR guide-RNAs targets each region, the region is flanked by PCR primers. Guides and primers adjacent to 5′ and 3′ LTRs are multiplexed. **c** As the region between the PCR primers is not sequenced, we created two sets of guides and primers (sets A and B). Following circularization, the sample is split, with CRISPR-mediated cleavage and PCR occurring separately for each set. After PCR, the products of the two sets of guides and primers are combined for sequencing. **d** Distribution of coverage across a BLV provirus (red line) and host DNA (blue line) in an expanded clone. Gray boxes: LTRs. The large drops in coverage adjacent to the LTRs correspond to the region between the PCR primers. The colored lines represent SNPs in the host genome

The genome size of the viruses targeted ranged from 6.8 to 9.7 kb; therefore, we chose to shear the DNA to ~ 8 kb in length. In most cases, this creates two fragments for each provirus, one containing the 5′ end with host DNA upstream of the insertion site and the second with the 3′ end and downstream host DNA. Depending on the shear site, the amount of host and proviral DNA in each fragment will vary (Fig. 1a). To facilitate identification of the provirus insertion site via inverse PCR we carry out intramolecular ligation, followed by digestion of the remaining linear DNA. To selectively linearize the circular DNA containing proviral sequences (this helps increase PCR efficiency), regions adjacent to the 5′ and 3′ LTRs in the provirus are targeted for CRISPR-mediated cleavage. We sought a balance between ensuring that the majority of the reads contained part of the flanking DNA (for clone identification) while also generating sufficient reads extending into the midpoint of the provirus. We found that using a pool of CRISPR guides for each region increased the efficiency and by multiplexing the guide pools and PCR primers for the 5′ and 3′ ends we could generate coverage for the majority of a clonally expanded provirus in a single reaction (Fig. 1b). The multiplexed pool of guides and primers leaves coverage gaps in the regions flanked by the primers. To address these coverage gaps, we designed a second set of guides and primers. Following separate CRISPR cleavage and PCR amplification, the products of these two sets of guides and primers were combined for sequencing (Fig. 1c). This approach ensured that the complete provirus was sequenced (Fig. 1d).

### Identifying genomic insertions and internal variants in HTLV-1

**Fig. 2:**
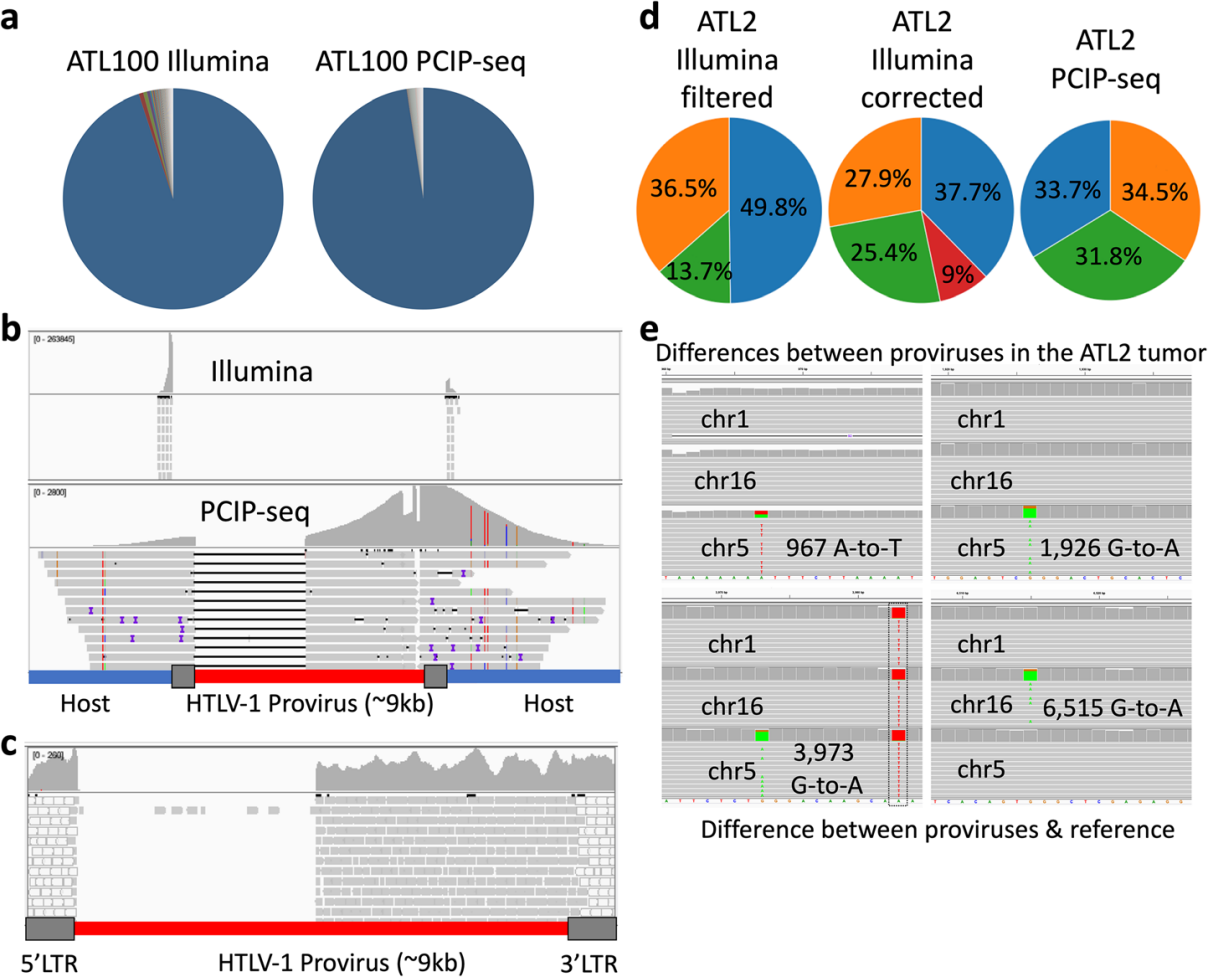
PCIP-seq applied to ATL. **a** In ATL100, both ligation-mediated PCR with Illumina sequencing (targeting the 5′ and 3′ LTRs) and PCIP-seq with Nanopore show a single predominant HTLV-1 insertion site. **b** Reads from both approaches have been mapped to a custom genome where the HLTV-1 provirus has been incorporated into the host genome. The long PCIP-seq Nanopore reads show this provirus has a ~ 3600 bp internal deletion, removing the binding sites of the guides/primers adjacent to the 5′ LTR. **c** Internal deletion confirmed via long range PCR and Illumina sequencing (gray reads map to a single position, the white reads map to both LTRs). **d** ATL2 clonality pie charts generated from ligation-mediated PCR with Illumina- and PCIP-seq-based sequencing data. The ATL2 tumor clone contains three proviruses inserted in chr 1, 5, and 16 (green, orange and blue slices respectively) named according to the chromosome inserted into. The provirus on chr1 (green slice) is inserted into a repetitive element (LTR) and short reads generated from host DNA flanking the insertion site by Illumina sequencing map to multiple positions in the genome. Filtering out multi-mapping reads causes an underestimation of the abundance of this insertion site (13.6%, left pie-chart). This can be partially corrected by retaining multi-mapping reads at this position (25.4%, central pie-chart). However, that approach can cause the potentially spurious inflation of other integration sites (red slice 9%). The long PCIP-seq reads can span repetitive elements and produce even coverage for each provirus without correction (right pie chart). **e** Screen shot from IGV shows representative PCIP-seq reads coming from the three proviruses (named chr 1, chr16, and chr5) and mapped to four distinct regions of the HTLV-1 proviral genome at positions where de novo mutations were observed

Adult T cell leukemia (ATL) is an aggressive cancer induced by HTLV-1. It is generally characterized by the presence of a single dominant malignant clone, identifiable by a unique proviral integration site. We and others have developed methods based on ligation-mediated PCR and Illumina sequencing to simultaneously identify integration sites and determine the abundance of the corresponding clones [2, 7]. We initially applied PCIP-seq to two HTLV-1 induced cases of ATL, both previously analyzed with our Illumina-based method (ATL2 [7] and ATL100 [22]). In ATL100, both methods identify a single dominant clone, with > 95% of the reads mapping to a single insertion site on chr18 (Fig. 2a, b and Table 1). Using the integration site information, we extracted the PCIP-seq hybrid reads spanning the provirus/host insertion site, uncovering a ~ 3600 bp deletion within the provirus (Fig. 2c).

**Table 1:** Number of unique insertion sites (IS) identified via PCIP-seq

Sample name	Virus	Host	PVL	Template μg	Raw reads	Chimeric reads (%)	Pure host / pure viral reads	Insertion sites	Largest clone (%)
ATL2	HTLV-1	HSA	nd	4	81,219	68.21	0.0037 / 31.8	160	49.5
ATL100	HTLV-1	HSA	106	4	4838	64.14	9.16 / 26.7	13	89.624
233	BLV	OAR	78.3	7	524,698	53.4	0.04 / 46.53	5311	5.22
221 (022016)	BLV	OAR	63	4	180,276	67.14	3.59 / 29.27	8023	0.625
221 (032014)	BLV	OAR	16	4	32,266	68.69	0.11 / 31.20	5374	0.279
220	BLV	OAR	3.8	2	44,876	67.38	0 / 32.62	1352	3.55
1439	BLV	BosT	45	3	181,055	70.52	0.19 / 29.29	5773	1.17
560	BLV	BosT	0.644	1	6802	69.83	1.12 / 29.06	172	4.59
1053	BLV	BosT	23.5	6	367,454	72.13	0.04 / 27.83	17,903	0.353
HIV_U1	HIV-1	HSA	200	2	94,086	54.66	2.75 / 42.59	728	47.2
Jurkat U1–0.1	HIV-1	HSA	0.2	5	252,913	43.33	0.04 / 56.62	4	71.7
Jurkat U1–0.01	HIV-1	HSA	0.02	5	234,421	43.33	0.04 / 56.52	2	90.2
Jurkat neg	HIV-1	HSA	0	5	12,137	0	100 / 0	0	0
02006	HIV-1	HSA	0.46	12	240,641	51.63	1.10 / 47.27	158	7.82
06042	HIV-1	HSA	0.56	8	226,685	21.18	0.41 / 78.41	73	4.77
HPV18_PX	HPV18	HAS	nd	4	180,550	21.36	0.29 / 78.35	55	nd
HPV18_PY	HPV18	HAS	nd	4	82,807	0.09	0.05 / 99.86	19	nd

*Chimeric reads* reads containing host and viral DNA, cover the integration site, *Pure host/pure viral reads* reads containing either host or viral DNA, do not include the integration site, *Largest clone %* insertion site with highest number of reads in that sample, *PVL* proviral load. (Percentage cells carrying a single copy of integrated provirus or number proviral copies per 100 cells)

In the case of ATL2, PCIP-seq showed three major proviruses located on chr5, chr16, and chr1, each responsible for ~ 33% of the HTLV-1/host hybrid reads. We had previously established that these three proviruses are in a single clone via examination of the T cell receptor gene rearrangement [7]. However, it is interesting to note that this was not initially obvious using our Illumina-based method, as the proviral insertion site on chr1 falls within a repetitive element (LTR) causing many of the reads to map to multiple regions in the genome. If multi-mapping reads are filtered out, the chr1 insertion site accounted for 13.7% of the remaining reads, while retaining multi-mapping produces values closer to reality (25.4%). In contrast, the long reads from PCIP-seq allow unambiguous mapping and closely matched the expected 33% for each insertion site (Fig. 2d), highlighting the advantage long reads have in repetitive regions. Looking at the three proviruses, proviral reads revealed all to be full length. Three de novo mutations were observed in one provirus and a single de novo mutation was identified in the second (Fig. 2e).

### Insertion sites identified in samples with multiple clones of low abundance

The samples utilized above represent a best-case scenario, with ~ 100% of cells infected and a small number of major clones. We next applied PCIP-seq to four samples from BLV infected sheep (experimental infection [23]) and three cattle (natural infection) to explore its performance on polyclonal and low proviral load (PVL) samples and compared PCIP-seq to our previously published Illumina method [7]. PCIP-seq revealed all samples to be highly polyclonal (Additional file 1: Fig. S1 and Table 1) with the number of unique insertion sites identified varying from 172 in the bovine sample 560 (1 μg template, PVL 0.644%) to 17,903 in bovine sample 1053 (6 μg template, PVL 23.5%). In general, PCIP-seq identified more insertion sites, using less input DNA than our Illumina-based method (Additional file 1: Table S1). Comparison of the results showed a significant overlap between the two methods. When we consider insertion sites supported by more than three reads in both methods (larger clones, more likely to be present in both samples), in the majority of cases > 50% of the insertion sites identified in the Illumina data were also observed via PCIP-seq (Additional file 1: Table S1). These results show the utility of PCIP-seq for insertion site identification, especially considering the advantages long reads have in repetitive regions of the genome.

### Identifying SNPs in BLV proviruses

Portions of the proviruses with more than ten supporting reads (PCR duplicates removed) were examined for SNPs with LoFreq [24]. For the four sheep samples, the variants were called relative to the pBLV344 provirus (used to infect the animals). For the bovine samples 1439 and 1053, custom consensus BLV sequences were generated for each and the variants were called in relation to the appropriate reference (SNPs were not called in 560). Across all the samples, 3209 proviruses were examined, 934 SNPs were called, and 680 (21%) of the proviruses carried one or more SNPs (Additional file 1: Table S2). We validated 10 BLV SNPs in the ovine samples and 15 in the bovine via clone-specific long-range PCR and Illumina sequencing (Additional file 1: Fig. S2). For Ovine 221, which was sequenced twice over a two-year interval, we identified and validated three instances where the same SNP and provirus were observed at both time points (Additional file 1: Fig. S2). We noted a small number of positions in the BLV provirus prone to erroneous SNP calls. By comparing allele frequencies from bulk Illumina and Nanopore data, these problematic positions could be identified and excluded (Additional file 1: Fig. S3a).

**Fig. 3:**
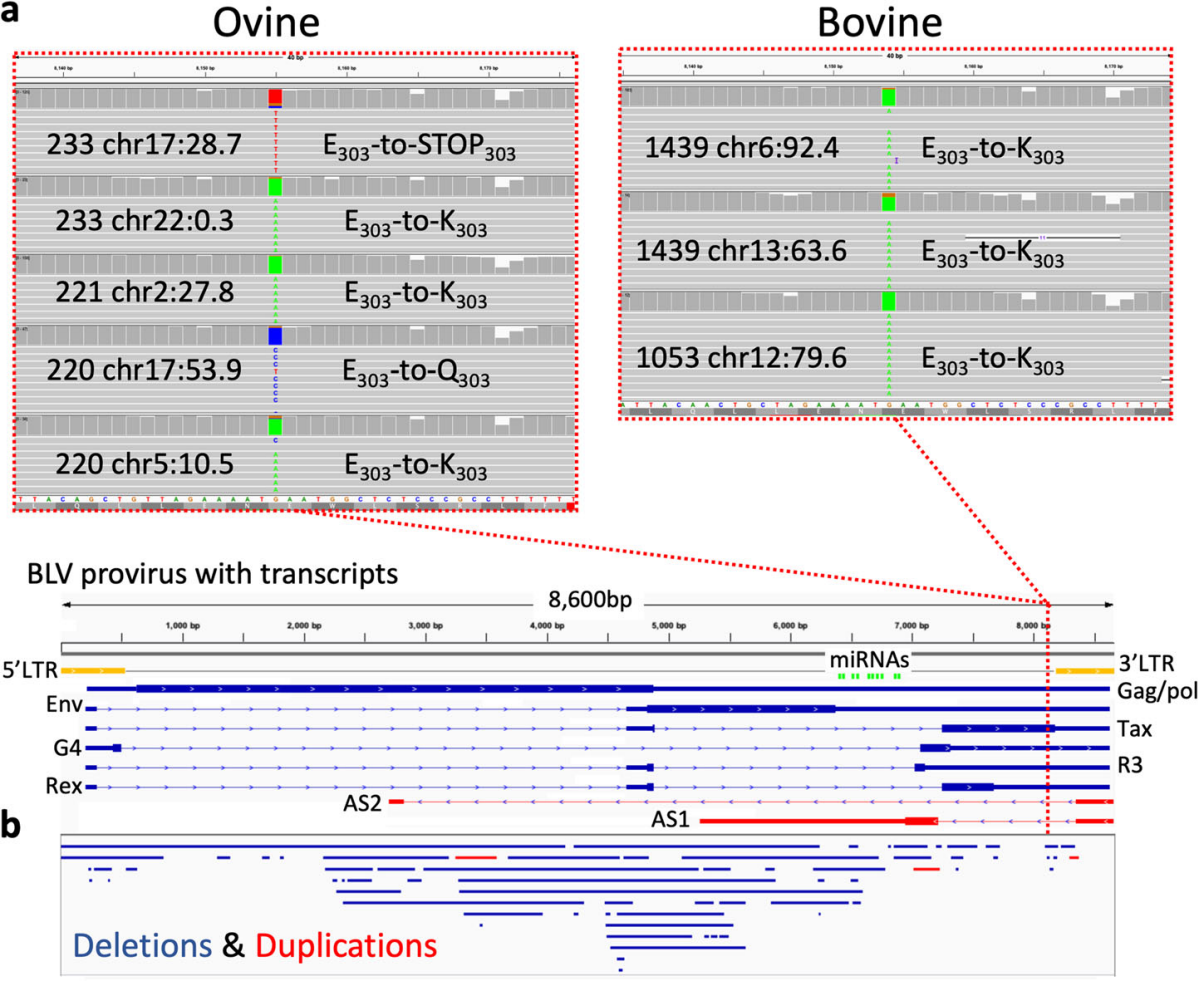
Variation in the BLV provirus. **a** Screen shot from IGV shows representative reads from a subset of the clones from each BLV-infected animal with a mutation in the first base of codon 303 in the viral protein Tax. Reads were mapped to the BLV proviral reference. Dotted red line shows approximate position within the BLV proviral genome represented below. **b** Structural variants observed in the BLV provirus. Deletions (blue bars) and duplications (red bars) in BLV proviruses identified in both ovine and bovine samples sequenced by PCIP-seq are represented below the BLV proviral genome

Approximately half of the SNPs (47.1% sheep, 51.6% cattle) were found in multiple proviruses. Generally, SNPs found at the same position in multiple proviruses were concentrated in a single individual, indicating their presence in a founder provirus or via a mutation in the very early rounds of viral replication (Additional file 1: Fig. S3b). Alternatively, a variant may also rise in frequency due to increased fitness of clones carrying a mutation in that position. In this instance, we would expect to see the same position mutated in multiple individuals. One potential example is found in the first base of codon 303 (position 8155) of the viral protein Tax, a potent viral transactivator, stimulator of cellular proliferation and highly immunogenic [25]. A variant was observed at this position in five proviruses for sheep 233 and three for sheep 221 as well as one provirus from bovine 1439 (Fig. 3a). Using less stringent criteria for the inclusion of a proviral region (> 10 reads, not filtered for PCR duplicates), we found 34 proviruses in the ovine and 3 in the bovine carrying a variant in this position. The majority of the variants observed were G-to-A transitions (results in E-to-K amino acid change); however, we also observed G-to-T (E-to-STOP) and G-to-C (E-to-Q) transversions. It has been previously shown that the G-to-A mutation abolishes the Tax protein transactivator activity [25, 26]. The repeated selection of variants at this specific position suggests that they reduce viral protein recognition by the immune system, while preserving the Tax proteins’ other proliferative properties.

Patterns of provirus-wide APOBEC3G [27] induced hypermutation (G-to-A) were not observed in BLV. However, three proviruses (two from sheep 233 and one in bovine 1053) showed seven or more A-to-G transitions, confined to a ~ 70-bp window in the first half of the U3 portion of the 3′LTR (Additional file 1: Fig. S4). The pattern of mutation, as well as their location in the provirus, suggests the action of RNA adenosine deaminases 1 (ADAR1) [28, 29].

### PCIP-seq identifies BLV structural variants in multiple clones

Proviruses were also examined for structural variants (SVs) using a custom script and via visualization in IGV (see “Methods”). Between the sheep and bovine samples, we identified 66 deletions and 3 tandem duplications, with sizes ranging from 15 to 4152 bp, with a median of 113 bp (Additional file 1: Table S3). We validated 14 of these via clone-specific PCR (Additional file 1: Fig. S5). As seen in Fig. 3b, SVs were found throughout the majority of the provirus, encompassing the highly expressed microRNAs [30] as well as the second exon of the constitutively expressed antisense transcript *AS1* [31]. Only two small regions at the 3′ end lacked any SVs. More proviruses will need to be examined to see if this pattern holds, but these results again suggest the importance of the 3′LTR and its previously reported interactions with adjacent host genes [7].

### Identifying HIV-1 integration sites and the associated provirus

Despite the effectiveness of combination antiretroviral therapy (ART) in suppressing HIV-1 replication, cART is not capable of eliminating latently infected cells, ensuring a viral rebound if cART is suspended [32]. This HIV-1 reservoir represents a major obstacle to a HIV cure [33], making its exploration a priority. However, this task is complicated by its elusiveness, with only ~ 0.1% of CD4^+^ T cells carrying integrated HIV-1 DNA [34]. To see if PCIP-seq could be applied to these extremely low proviral loads, we initially carried out dilution experiments using U1 [35], an HIV-1 cell line containing replication-competent proviruses [36]. PCIP-seq on undiluted U1 DNA found the major insertion sites on chr2 and chrX (accounting for 47% and 41% of the hybrid reads respectively) and identified the previously reported variants that disrupt Tat function [37] in both proviruses (Additional file 1: Fig. S6a). In addition to the two major proviruses, we identified an additional ~ 700 low abundance insertion sites (Table 1), including one on chr19 (0.8%) reported by Symons et al. [36] that is actually a product of recombination between the major chrX and chr2 proviruses (Additional file 1: Fig. S6b). We then serially diluted U1 DNA in Jurkat cell line DNA. PCIP-seq was carried out with 5 μg of template DNA where U1 represents 0.1% and 0.01% of the total DNA. We also processed 5 μg of Jurkat DNA in parallel as a negative control. We were able to detect the major proviruses on chr2 and chrX in both dilutions. We estimate that in the 0.01% dilution, we captured ~ 3.2% of the proviruses present in the original sample (Additional file 1: Fig. S7a and Table 1). No reads mapping to HIV-1 were observed in the negative control (Additional file 1: Fig. S7b and Table 1).

**Fig. 4:**
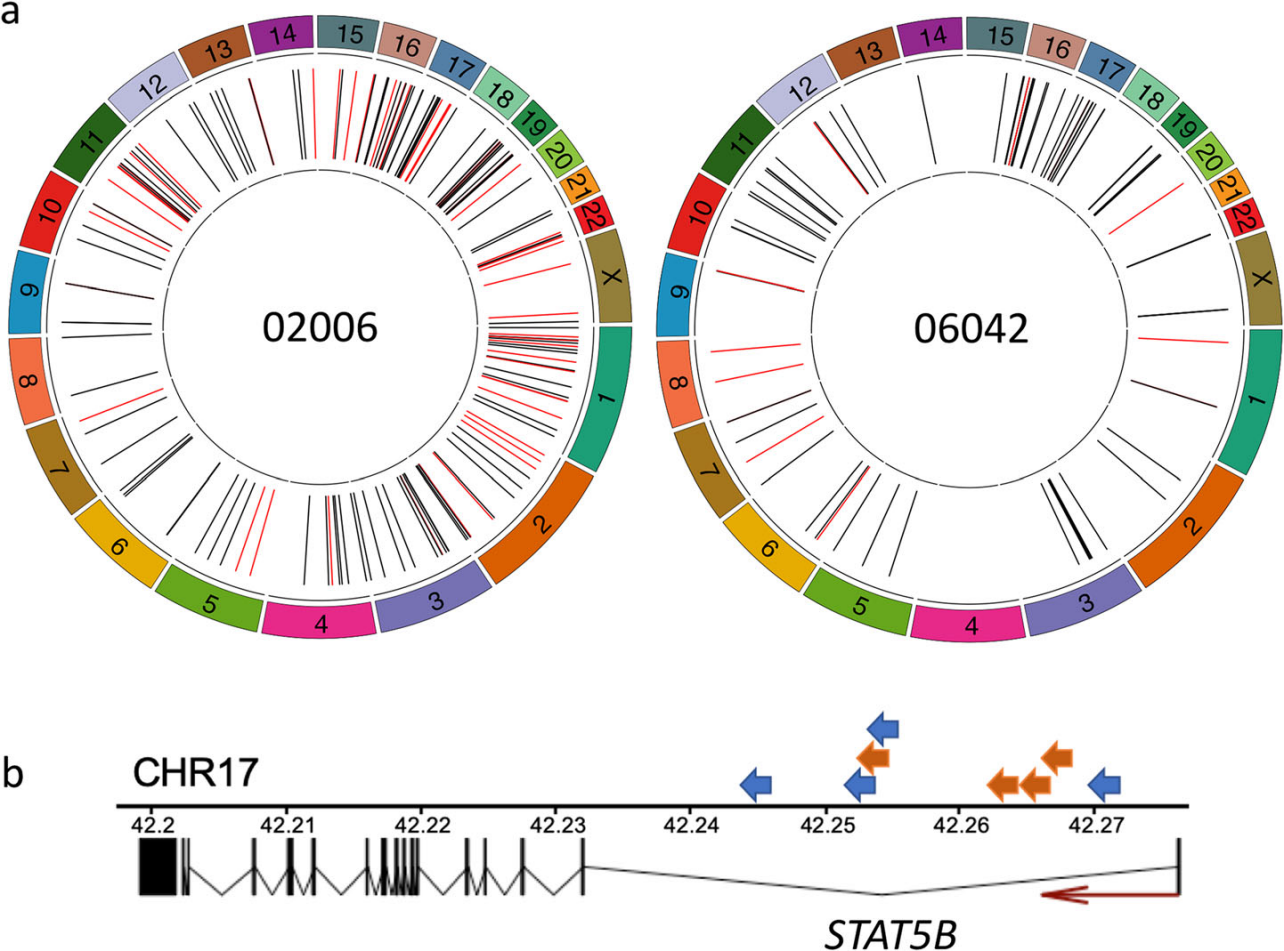
Location of HIV-1 proviral integration sites identified by PCIP-seq in patients on cART. **a** HIV-1 proviral integration sites identified by PCIP-seq in two HIV-1 patients (02006 and 06042). Black lines represent integration sites where the portion of the provirus sequence shows no evidence of a large deletion, and red lines indicate sites where a large deletion was observed in the provirus. Detailed information for each HIV-1 integration site identified by PCIP-seq is available in Additional file 2. **b** A hotspot of proviral integration in intron 1 of *STAT5B.* Arrows represent individual proviruses (02006 = blue, 06042 = orange), and direction indicates the orientation of the provirus. All proviruses have the same transcriptional orientation as *STAT5B*

We next carried out PCIP-seq on DNA extracted from the CD4^+^ T cells of two HIV-1-infected patients (06042 and 02006) on long-term cART (Additional file 1: Table S4). Using 8 μg of template DNA, we identified 73 unique integration sites in 06042. In 02006, using 12 μg template DNA, we identified 158 (Fig. 4 and Additional file 2). Examination of the shear sites in the reads at each integration site via IGV allowed us to differentiate between integration sites sequenced from a single provirus and a provirus in clonally expanded cells, where multiple shear sites in the host genome can be observed.

We validated the integration sites of 5 proviruses using clone-specific PCR (Additional file 2). In the majority of the integration sites, only a subset of the associated provirus is sequenced; however, it was still possible to identify 12 proviruses from 06042 and 52 in 02006 with large deletions (Additional file 1: Fig. S8a and Additional file 2). Additionally, we generated approximately 500 kb of HIV provirus sequence that can be linked to specific integration sites. In 02006, we found four clonally expanded full-length proviruses with reads covering the entire provirus (Additional file 2). One contained a ~ 115 bp deletion just upstream of *gag*, disrupting the packaging signal (Ψ) (Additional file 1: Fig. S8b). Two of them had sufficient coverage to generate a consensus sequence of the full-length provirus, and both appear to be intact (Additional file 3). One maps to a segmentally duplicated region just below the centromere on chr10 and chr1 respectively, while the other has flanking sequence that matches the satellite repeats of the centromeres of chr13, chr14, chr21, and chr22. Both patients had four integration sites in intron 1 of *STAT5B*, all were in the same transcriptional orientation as *STAT5B* (Fig. 4). An enrichment of HIV-1 integrations in this region has previously been reported [3, 4, 6], with recent work showing them to cause insertional activation of *STAT5B*, which favors T regulatory cell persistence [38].

In order to explore a way of reducing the amount of starting DNA for HIV-1-infected primary samples, we carried out multiple displacement amplification (MDA) prior to carrying PCIP-seq. Using 10 ng and 100 ng of DNA as template for MDA, we generated ~ 10 μg of amplified DNA and used 4 μg of this as template for PCIP-seq. For 02006, we identified 13 integration sites in the 100 ng MDA sample and 3 in the 10 ng MDA. Two of the 10 ng MDA integrations were also observed in the 100 ng MDA sample, giving a total of 14 integration sites for both. All but 4 of these proviruses had been identified by PCIP-seq in the previous libraries. For 06042, we identified 23 proviruses in the 100 ng MDA and 2 in the 10 ng MDA sample (25 in total). Only one of these proviruses had been seen in the in the previous PCIP-seq libraries from this patient (Additional file 2). The differing levels of overlap between libraries suggests a higher proportion of clonally expanded cells in patient 02006, a trend that was also visible in the non MDA PCIP-seq libraries (Additional file 2).

### Identifying full-length and polymorphic endogenous retroviruses in cattle and sheep

**Fig. 5:**
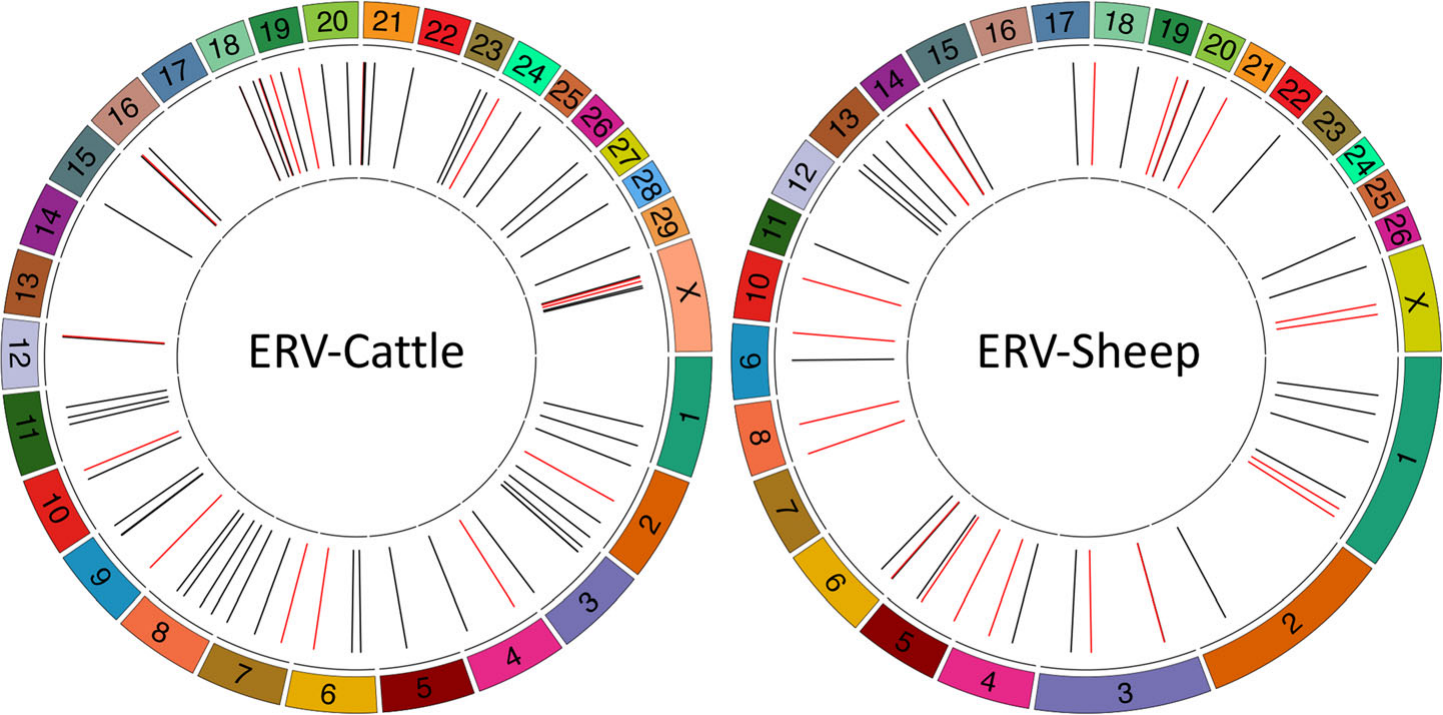
Location of endogenous retroviruses identified by PCIP-seq in cattle and sheep genomes. Based on three cattle and two sheep. Black lines represent full-length proviruses, and red lines represent proviruses containing large deletions. Detailed information for each integration site identified by PCIP-seq is available in Tables S5 and S6

ERVs in the genome can be present as full length, complete provirus, or more commonly as solo-LTRs, the products of non-allelic recombination [39]. At the current time, conventional short-read sequencing, using targeted or whole genome approaches, cannot distinguish between the two classes. Examining full-length ERVs would provide a more complete picture of ERV variation, while also revealing which elements can produce de novo ERV insertions. As PCIP-seq targets inside the provirus we can preferentially amplify full length ERVs, opening this type of ERV to study in larger numbers of individuals. As a proof of concept, we targeted the class II bovine endogenous retrovirus BERVK2, known to be transcribed in the bovine placenta [40]. We applied the technique to three cattle, of which one (10201e6) was a Holstein suffering from cholesterol deficiency, an autosomal recessive genetic defect recently ascribed to the insertion of a 1.3 kb LTR in the *APOB* gene [41]. PCIP-seq clearly identified the *APOB* ERV insertion in 10201e6 and in contrast to previous reports [41] shows it to be a full-length element (Additional file 1: Fig. S9). We identified a total of 67 ERVs (Fig. 5), with eight present in all three samples (Additional file 1: Table S5). We validated three ERVs via long-range PCR and Illumina sequencing (Additional file 1: Fig. S10). We did not find any with an identical sequence to the *APOB* ERV, although the ERV BTA3_115.3 has an identical LTR sequence, highlighting that the sequence of the LTR cannot be used to infer the complete sequence of the ERV (Additional file 1: Fig. S11).

We also adapted PCIP-seq to amplify the Ovine endogenous retrovirus Jaagsiekte sheep retrovirus (enJSRV), a model for retrovirus-host co-evolution [42]. Using two sheep (220 and 221) as template, we identified a total of 48 enJSRV proviruses (Fig. 5) (33 in 220 and 38 in 221, with 22 common to both) and of these ~ 54% were full length (Additional file 1: Table S6). We validated seven proviruses via long-range PCR and Illumina sequencing (Additional file 1: Fig. S12).

### Extending PCIP-seq to human papillomaviruses (HPV)

**Fig. 6:**
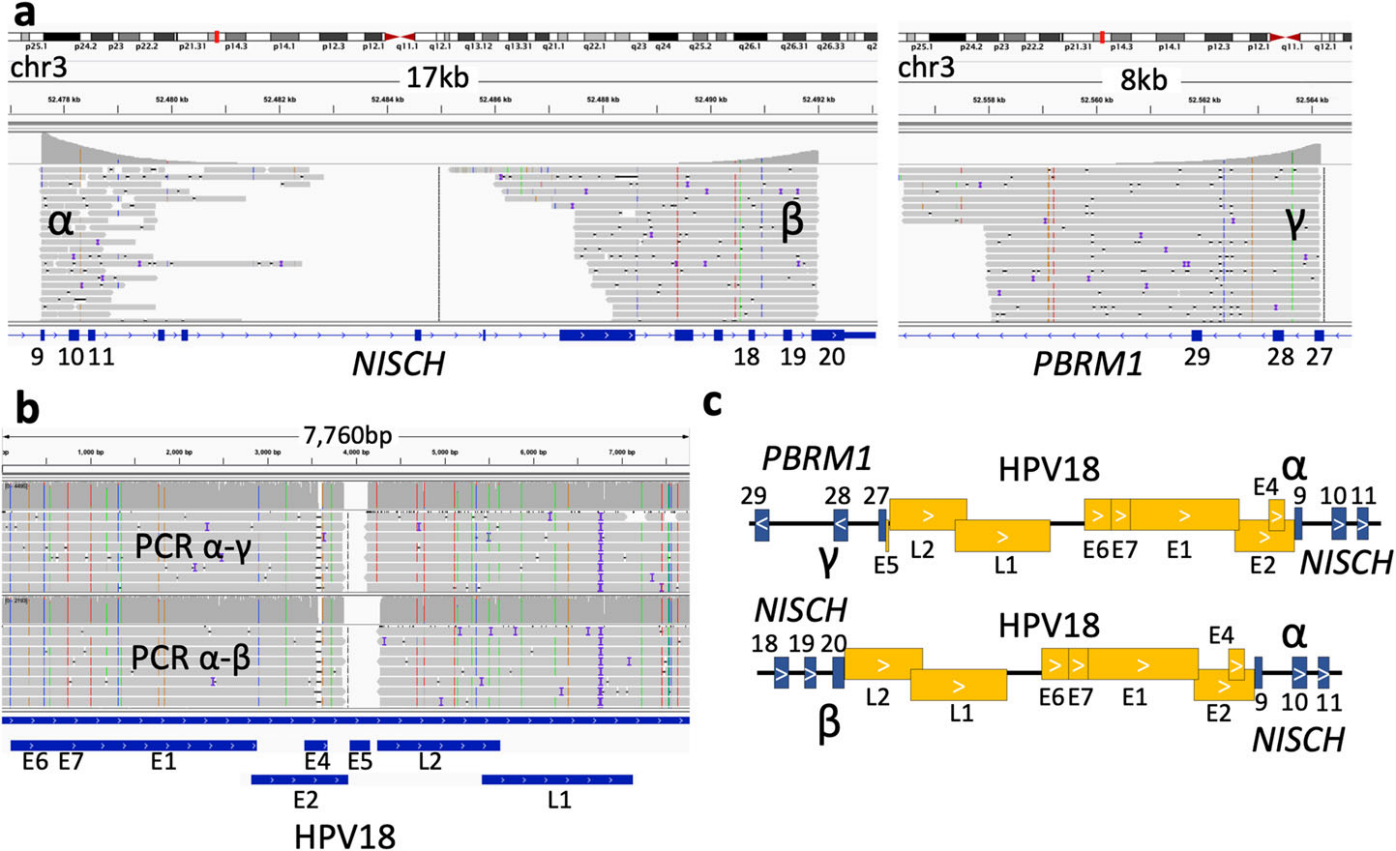
HPV integration site in an expanded clone. In this expanded clone HPV shows evidence of “looping” integration [18, 44] whereby noncontiguous genomic sequences are brought adjacent to one another. **a** PCIP-seq reads mapping to a ~ 87-kb region on chr3 revealed three HPV-host breakpoints. The large number of reads suggests expansion of the clone carrying these integrations. **b** PCR was carried out with primer pairs matching regions α and β, as well as α and γ. Both primer pairs produced a ~ 9 kb PCR product. Nanopore sequencing of the PCR products show the HPV genome connects these breakpoints. **c** Schematic of the breakpoints with the integrated HPV genome. This conformation indicates that this dramatic structural rearrangement in the host genome was generated via “looping” integration of the HPV genome

The majority of HPV infections clear or are suppressed within 1–2 years [43]; however, a minority evolve into cancer, and these are generally associated with integration of the virus into the host genome. This integration into the host genome is not part of the viral lifecycle and the breakpoint in the viral genome can occur at any point across its 8 kb circular genome [18]. As a consequence, the part of the viral genome found at the virus host breakpoint varies considerably, making the identifying of integration sites difficult using existing approaches [18]. The long reads employed by PCIP-seq mean that even when the breakpoint is a number of kilobases away from the position targeted by primers we should still capture the integration site. As a proof of concept, we applied PCIP-seq to two HPV18-positive cases (HPV18_PX and HPV18_PY) using 4 μg of DNA extracted from left over Papanicolaou tests (Pap smear). We identified 55 integration sites in HPV18_PX and 19 integration sites in HPV18_PY (Additional file 1: Table S7). In HPV18_PY, the vast majority of the reads only contained HPV sequences, and the integration sites identified were defined by single reads, suggesting little or no clonal expansion (Table 1). In HPV18_PX most integration sites were again defined by a single read; however, there were some exceptions (Additional file 1: Table S7). The most striking of these was a cluster of what appeared to be three integration sites located within the region chr3:52477576-52564190 (Fig. 6a). The unusual pattern of read coverage combined with the close proximity of the virus-host breakpoints indicated that these three integration sites were connected. Long-range PCR with primers spanning positions α-β and α-γ showed that a genomic rearrangement had occurred in this clonally expanded cell (Fig. 6a). Regions α and β are adjacent to one another with HPV integrated between; however, PCR also showed regions α and γ to be adjacent to one another, again with the HPV genome integrated between (Fig. 6b). The sequence of the virus found between α-β looks to be derived from the α-γ virus as it shares a breakpoint and is slightly shorter (Fig. 6b). This complex arrangement suggests that this rearrangement was generated via the recently described “looping” integration mechanism [18, 44]. The α and β breakpoints fall within exons of the *NISCH* gene while the γ breakpoint falls within exon 27 of *PBRM1* (Fig. 6c), a gene previously shown to be a cancer driver in renal carcinoma [45] and intrahepatic cholangiocarcinomas [46].

## Discussion

In the present report, we describe how PCIP-seq can be utilized to identify insertion sites while also sequencing parts of and in some cases the entire associated provirus, and confirm this methodology is effective with a number of different retroviruses as well as in HPV. For insertion site identification, the method was capable of identifying more than ten thousand BLV insertion sites in a single sample, using ~ 4 μg of template DNA. Even in samples with a PVL of 0.66%, it was possible to identify hundreds of insertion sites with only 1 μg of DNA as template. The improved performance of PCIP-seq in repetitive regions further highlights its utility, strictly from the standpoint of insertion site identification. In addition to its application in research, high-throughput sequencing of retrovirus insertion sites has shown promise as a clinical tool to monitor ATL progression [22]. Illumina-based techniques require access to a number of capital-intensive instruments. In contrast, PCIP-seq libraries can be generated, sequenced, and analyzed with the basics found in most molecular biology labs; moreover, preliminary results are available just minutes after sequencing begins [47]. As a consequence, the method may have use in a clinical context to track clonal evolutions in HTLV-1-infected individuals, especially as the majority of HTLV-1-infected individuals live in regions of the world with poor biomedical infrastructure [48].

One of the common issues raised regarding Oxford Nanopore data is read accuracy. Early versions of the MinION had read identities of less than 60% [49]; however, the development of new pores and base calling algorithms make read identities of > 90% achievable [50]. Accuracy can be further improved by generating a consensus from multiple reads, making accuracies of ~ 99.4% [50] possible. Recently, Greig et al. [51] compared the performance of Illumina and Oxford Nanopore technologies for SNP identification in two isolates of *Escherichia coli.* They found that after accounting for variants observed at 5-methylcytosine motif sequences only ~ 7 discrepancies remained between the platforms. It should be noted that as PCIP-seq sequences PCR amplified DNA, errors generated by base modifications will be avoided. Despite these improvements in accuracy, Nanopore-specific errors can be an issue at some positions (Additional file 1: Fig. S3a). Comparison with Illumina data is helpful in the identification of problematic regions and custom base calling models may be a way to improve accuracy in such regions [50]. More generally, we compensated for the higher error rate in Nanopore reads by only calling SNPs in regions of the provirus covered by more than 10 non-PCR duplicated reads (due to PCR duplicates coverage at these positions was generally substantially higher than 11×). Continued improvements in base calling accuracy and the development of new pores [52] mean these requirement are likely to be overly conservative in most instances. In the current study, we focused on SNPs observed in clonally expanded BLV proviruses. For viruses such as HIV-1, which have much lower proviral loads, more caution will be required as the majority of proviral sequences will be generated from single provirus, making errors introduced by PCR more of an issue. To address this concern, we carried out Illumina sequencing of the two HIV patient PCIP-seq libraries in order to call SNPs in the host DNA flanking the integration sites as proxy for the provirus itself (Additional file 1, Supplementary Note 2, Effect of coverage on SNP calling). Our data indicates that as coverage decreases the number of false negatives increases (rises to ~ 19% at 20×); however, there was no concomitant increase in false positives. Finally, while we have utilized Nanopore in the current study, PCIP-seq libraries could equally be sequenced using high accuracy long reads on the Pacific Biosciences platform [53]. As these reads have accuracies on a pair with Illumina reads, they would allow larger numbers of proviruses to be examined for SNPs.

When analyzing SNPs from BLV, the most striking result was the presence of the recurrent mutations at the first base of codon 303 in the viral protein Tax, a central player in the biology of both HTLV-1 [48] and BLV [54]. It has previously been reported that this mutation causes an E-to-K amino acid substitution which ablates the transactivator activity of the Tax protein [25]. Collectively, these observations suggest this mutation confers an advantage to clones carrying it, possibly contributing to immune evasion, while retaining Tax protein functions that contribute to clonal expansion. However, there is a cost to the virus as this mutation prevents infection of new cells due to the loss of Tax-mediated transactivation of the proviral 5′ LTR making it an evolutionary dead end. It will be interesting to see if PCIP-seq can provide a tool to identify other examples of variants that increase the fitness of the provirus in the context of an infected individual but hinder viral spread to new hosts. Additionally, the technique could be used to explore the demographic features of the proviral population within and between hosts, how these populations evolve over time and how they vary.

A second notable observation in BLV is the cluster of A-to-G transitions observed within a ~ 70-bp window in the 3′ LTR. Similar patterns have been ascribed to ADAR1 hypermutation in a number of viruses [28], including the close BLV relatives HTLV-2 and simian T cell leukemia virus type 3 (STLV-3) [55]. Given the small number of hypermutated proviruses observed, it appears to be a minor source of variation in BLV, although it will be interesting to see it this holds for different retroviruses and at different time points during infection.

Like the situation in HTLV-1/BLV, a number of methods based on linker-mediated PCR and Illumina sequencing have been developed to identify HIV-1 integration sites [3, 38]. Due to the limited number of cells carrying the HIV-1 provirus in patients undergoing cART, the number of integration sites typically recovered is generally low. For example, using DNA from 54 patients (1 μg for each), Cesana et al. [38] recovered 198 unique integration sites (median 3 integration sites per patient). Maldarelli et al. [3] recovered a median of 135 integration sites per patient/timepoint analyzed (using 9 μg of DNA as template). As the proviral loads of the samples in these studies are not given, a direct comparison of results is not possible; however, our recovery of 73 integration sites using 8 μg of template in 06042 and 158 using 12 μg in 02006, falls within the range observed in these studies. The integration sites recovered represent approximately 1.06% and 2.95% of the proviruses present in the starting DNA for patient 06042 and 02006 respectively (Additional file 2, Additional file 1: Table S8). For 02006, this is close to the 3.2% estimated from the dilution experiment using U1 cells. The lower efficiency in 06042 may be due to suboptimal guide and primer design. This highlights that PCIP-seq will be sensitive to the performance of primers and guides, especially in HIV-1 due to the polymorphic nature of the proviruses. Nevertheless, taken with the results for BLV/HTLV-1, it appears that PCIP-seq and methods based on ligation-mediated PCR followed by short read sequencing identify comparable numbers of integration sites, while PCIP-seq has the added advantage of sequencing within the provirus and in some cases the entire associated provirus (Additional file 1: Table S1 and S8).

More recently, it has been shown that DNA-capture-seq approaches using biotin capture probes and short reads can successfully identify integration sites in HTLV-1-infected patients [11] and HIV-1-infected cell lines [56]. In cases where highly expanded clones are present or where deletions affect the 5′ or 3′ ends of the provirus (which generates reads spanning the breakpoint and integration site), it is possible to link a variant to a provirus at a specific integration site. However, when the variant falls inside the provirus, beyond the reach of reads that contain both host and viral DNA (~ 700 bp), this is not possible. One of the first methods to address this problem was integration site loop amplification (ISLA) [4]. This method relies on diluting the sample to a point where each well contains on average 3 HIV-1 proviruses. Each well is then subjected to multiple rounds of linear PCR, exonuclease digestion, multiple rounds of exponential PCR, gel electrophoresis, and finally Sanger sequencing of the integration site and a portion of the *env* gene. As only ~ 30% of the wells are positive, if large numbers of integration sites are examined the cost and labor involved becomes substantial. PCIP-seq is more economical per integration site, while also having the advantage of generating proviral sequence (~ 500 kb in this experiment) that can be linked to specific integration sites (Additional file 2). More recently, others [6, 13] have developed methods to capture the entire proviral sequence as well as the associated integration site. However, these methods require even more extreme dilutions than ISLA, requiring that each well contains a single provirus. These wells are then subjected to whole genome amplification and split. Half of the DNA is then used for ISLA or another technique to identify the integration sites, while the other half is used to amplify the provirus via clone specific primers or with primers that encompass the majority of the provirus. This approach is obviously capable of capturing full-length provirus and the associated integration site, but is even more costly and labor intensive than ISLA alone, making it impractical to investigate more than a handful of patients.

Only a small fraction of proviruses (~ 2.4%) in the HIV-1 reservoir are intact, yet these are more than sufficient for the disease to rebound if antiretroviral therapy is interrupted [5]. As strategies are developed to target these intact proviruses, it will be essential to distinguish between intact and defective proviruses [5]. Due to the low proviral load and limited clonal expansion observed in patients on long-term cART, the majority of HIV-1 proviruses captured by PCIP-seq are only partially sequenced (on average ~ 2.4 kb). Nevertheless, despite this limitation, it is still possible to classify many as defective due to the presence of deletions or hypermutation. Additionally, in the case of patient 02006 (on cART for 15 years), we were able to generate sequences of two intact full-length proviruses present in clonally expanded cells. These proviruses are integrated within highly repetitive/heterochromatic regions and as a result they are likely to be resistant to reactivation. Recently, Jiang et al. [57] observed that 20.7% of intact proviral sequences are imbedded in centromeric satellite or microsatellite DNA, while Einkauf et al. [6] showed an enrichment of intact HIV-1 proviruses in non-genic chromosomal positions. These results indicate that proviruses integrated into parts of the genome that provide an unfavorable environment for viral expression are protected against recognition by the host immune system, favoring their survival in patients on long-term cART.

In the current study, we focused our analysis on retroviruses and ERVs. However, as this methodology is potentially applicable to a number of different targets, we extended its use to HPV as a proof of concept. It is estimated that HPV is responsible for > 95% of cervical carcinoma and ~ 70% of oropharyngeal carcinoma [58]. While infection with a high-risk HPV strain (HPV16 and HPV18) is generally necessary for the development of cervical cancer, it is not sufficient and the majority of infections resolve without adverse consequences [43]. The use of next-generation sequencing has highlighted the central role HPV integration plays in driving the development of cervical cancer [18]. Our results show that PCIP-seq can be applied to identify HPV integration sites in early precancerous samples. This opens up the possibility of generating a more detailed map of HPV integrations as well as potentially providing a biomarker to identify HPV integrations on the road to cervical cancer.

Looking beyond viruses tested in the current study, hepatitis B virus (HBV) is an obvious candidate for PCIP-seq. Like HPV, it has a circular DNA genome that integrates into the host genome with variable breakpoints in the viral genome. HBV integrations contribute to genomic instability and play a key role in driving hepatocarcinogenesis [59]. Other potential applications include determining the insertion sites and integrity of retroviral vectors [60] and detecting transgenes in genetically modified organisms. We envision that in addition to the potential applications outlined above many other novel targets/questions could be addressed using this method.

## Conclusions

The genomic location of viral integration as well as variation within the virus plays a role in determining the fate of infected cells. Up to now, linking the sequence of the viral genome to a specific integration site and measuring the abundance of the corresponding clone has been difficult. We have shown that PCIP-seq can identify integration sites while sequence part and in many cases all of the associated provirus. For BLV, we identified thousands of SNPs and dozens of structural variants within integrated viruses. In HIV-1-infected patients, we identified intact HIV-1 provirus. Finally, we show that PCIP-seq is also adaptable to HPV, where it can identify integrations at early time points that are associated with clonal expansion.

## Methods

### Samples

Both the BLV-infected sheep [7] and HTLV-1 samples [7, 22] have been previously described. Briefly, the sheep were infected with the molecular clone pBLV344 [23], following the experimental procedures approved by the University of Saskatchewan Animal Care Committee based on the Canadian Council on Animal Care Guidelines (Protocol #19940212). The HTLV-1 samples [7, 22] were obtained with informed consent and the full study protocol received approval from the institutional review board of the Necker Hospital, University of Paris, France (CPP Ile de France II, CNIL: number 1692254, registration number 000001072), in accordance with the Declaration of Helsinki. The BLV bovine samples were natural infections, obtained from commercially kept adult dairy cows in Alberta, Canada. Sampling was approved by VSACC (Veterinary Sciences Animal care Committee) of the University of Calgary: protocol number: AC15-0159. The bovine 571 used for ERV identification was collected as part of this cohort. The two sheep samples used for Jaagsiekte sheep retrovirus (enJSRV) identification were the BLV-infected ovine samples (220 and 221 (032014)), with a PVL of 3.8 and 16% respectively. PBMCs were isolated using standard Ficoll-Hypaque separation. The DNA for the bovine Mannequin was extracted from sperm, while the DNA for bovine 10201e6 was extracted from whole blood using standard procedures. The HIV-1 U1 cell line DNA sequenced without dilution was provided by Dr. Carine Van Lint, IBMM, Gosselies, Belgium. The HIV-1 U1 cell line dilutions in Jurkat were generated at Ghent University Hospital. HIV-1-positive primary PBMCs were collected at the Ghent University Hospital from two HIV-1-positive individuals (patients 02006 and 06042, Additional file 1: Table S4) on cART for 15 and 8 years respectively. Patient PBMC samples were de-identified and the full study was approved by the Ethics Committee of Ghent University Hospital (Reference number: 2016/0457). HPV material was prepared from PAP smears obtained from HPV-infected patients at the CHU Liège University hospital. Both patients were PCR positive for HPV18, HPV18_PY was classified as having Atypical Squamous Cell of Undetermined Significance (ASC-US), while HPV18_PX was classified as having Atypical Glandular Cells (AGC). Patients provided written informed consent and the study was approved by the Comité d’Ethique Hospitalo-Facultaire Universitaire de Liège (Reference number: 2019/139). No statistical test was used to determine adequate sample size and the study did not use blinding.

### CD4 enrichment of HIV-1 patient PBMCs

CD4^+^ T cells were enriched from PBMCs by negative MACS selection using the EasySep™ Human CD4^+^ T Cell Isolation Kit (STEMCELL Technologies SARL, Grenoble, France), according to the manufacturer’s instructions.

### PCIP-seq

Total genomic DNA isolation was carried out using the Qiagen AllPrep DNA/RNA/miRNA kit (BLV-, HTLV-1-, and HPV-infected individuals) or the Qiagen DNeasy Blood and Tissue Kit (HIV-1 patients) according to the manufacturer’s protocol. High molecular weight DNA was sheared to ~ 8 kb using Covaris g-tubesTM (Woburn, MA) or a Megaruptor (Diagenode), followed by end-repair using the NEBNext EndRepair Module (New England Biolabs). In the case of MDA, 10 ng and 100 ng of DNA from HIV-1 patient samples was used as template for the illustra GenomiPhi V2 DNA Amplification Kit. The resultant amplified DNA was then treated the same as the equivalent amount of genomic DNA. Intramolecular circularization was achieved by overnight incubation at 16 °C with T4 DNA Ligase. Remaining linear DNA was removed with Plasmid-Safe-ATP-Dependent DNAse (Epicenter, Madison WI). Due to the inefficiency of intramolecular ligation 85–90% of the starting DNA is lost during this step. Guide RNAs were designed using chopchop (http://chopchop.cbu.uib.no/index.php). The EnGen™ sgRNA Template Oligo Designer (http://nebiocalculator.neb.com/#!/sgrna) provided the final oligo sequence. Oligos were synthesized by Integrated DNA Technologies (IDT). Oligos were pooled and guide RNAs synthesized with the EnGen sgRNA Synthesis kit, *S. pyogenes* (New England Biolabs). Selective linearization reactions were performed with the Cas-9 nuclease, *S. pyogenes* (New England Biolabs). (Additional file 1, Supplementary Text, rationale behind using of CRISPR-cas9 to cleave the circular DNA). PCR primers flanking the cut sites were designed using primer3 (http://bioinfo.ut.ee/primer3/). For HIV-1 samples, we first sequenced the parts of the provirus flanking the LTR and the consensus sequence of these regions was used to select a set of primers and guides tailored to the population of proviruses in the patient. Primers were tailed to facilitate the addition of Oxford Nanopore indexes in a subsequent PCR reaction. The linearized fragments were PCR amplified with LongAmp Taq DNA Polymerase (New England Biolabs) and purified using 1× AmpureXP beads (Beckman Coulter). A second PCR added the appropriate Oxford Nanopore index. PCR products were visualized on a 1% agarose gel, purified using 1× AmpureXP beads and quantified on a Nanodrop spectrophotometer. Indexed PCR products were multiplexed and Oxford Nanopore libraries prepared with either the Ligation Sequencing Kit 1D (SQK-LSK108) or 1D^2 Sequencing Kit (SQK-LSK308) (only the 1D were used). Resulting libraries were sequenced on Oxford Nanopore MinION R9.4 or R9.5 flow cells respectively. The endogenous retrovirus libraries were base called using albacore 2.3.1, all other PCIP-seq libraries were base called with Guppy 3.1.5 (https://nanoporetech.com) using the “high accuracy” base calling model. For the endogenous retrovirus libraries, demultiplexing was carried out via porechop (https://github.com/rrwick/Porechop) using the default setting. The HIV, HTLV-1, BLV, and HPV PCIP-seq libraries were subjected to a more stringent demultiplexing with the guppy_barcoder (https://nanoporetech.com) tool using the --require_barcodes_both_ends option. The output was also passed through porechop, again barcodes were required on both ends, adapter sequence was trimmed, and reads with middle adapters were discarded. Oligos used can be found in Additional file 4. A step by step description of PCIP-seq library preparation can be found in Additional file 1: Supplementary Methods.

### Identification of proviral integration sites in PCIP-seq

Reads were mapped with Minimap2 [61] to the host genome with the proviral genome as a separate chromosome. In-house R-scripts were used to identify integration sites (IS). Briefly, chimeric reads that partially mapped to at least one extremity of the proviral genome were used to extract virus-host junctions and shear sites. Junctions within a 200-bp window were clustered together to form an “IS cluster,” compensating for sequencing/mapping errors. The IS retained corresponded to the position supported by the highest number of virus-host junctions in each IS cluster. Clone abundance was estimated based on the number of reads supporting each IS cluster. Reads sharing the same integration site and same shear site were considered PCR duplicates. Custom software, code description, and detailed outline of the workflow are available on Github: https://github.com/GIGA-AnimalGenomics-BLV/PCIP.

### Measure of proviral load (PVL) and identification of proviral integration sites (Illumina)

PVLs and integration sites of HTLV-1- and BLV-positive individuals were determined as previously described in Rosewick et al. [7] and Artesi et al. [22]. PVL represents the percentage of infected cells, considering a single proviral integration per cell. Total HIV-1 DNA content of CD4 T cell DNA isolates was measured by digital droplet PCR (ddPCR; QX200 platform, Bio-Rad), as described by Rutsaert et al. [62] (Additional file 1, Supplementary Methods) and data was analyzed with ddpcRquant [63].

### Variant calling

After PCR duplicate removal, proviruses with an IS supported by more than 10 reads were retained for further processing. SNPs were identified using LoFreq [24] with default parameters, only SNPs with an allele frequency of > 0.6 in the provirus associated with the insertion site were considered. We also called variants on proviruses supported by more than 10 reads without PCR duplicate removal (this greatly increased the number of proviruses examined). This data was used to explore the number of proviruses carrying the Tax 303 variant. Deletions were called on proviruses supported by more than 10 reads without PCR duplicate removal using in house R-scripts. Briefly, samtools pileup [64] was used to compute coverage and deletions at base resolution. We used the changepoint detection algorithm PELT [65] to identify genomic windows showing an abrupt change in coverage. Windows that showed at least a 4-fold increase in the frequency of deletions (absence of a nucleotide for that position within a read) were flagged as deletions and visually confirmed in IGV [66].

### HIV-1 proviral sequences

Sequences of the two major proviruses integrated in chr2 and chrX of the U1 cell line were generated by mapping the reads from both platforms to the HIV-1 provirus, isolate NY5 (GenBank: M38431.1), where the 5′LTR sequence is appended to the end of the sequence to produce a full-length HIV-1 proviral genome reference. The sequence was then manually curated to produce the sequence for each provirus. To check for recombination, reads of selected clones were mapped to the sequence from the chrX provirus and the patterns of SNPs examined to determine if the variants matched the chrX or chr2 proviruses.

The consensus HIV-1 sequences for both patients were generated using the medaka consensus tool (https://github.com/nanoporetech/medaka), followed by manual correction guided by Illumina reads generated from the same PCIP-seq library. The Illumina libraries were prepared as described in Durkin et al. [31]. The consensus sequences of two full-length proviruses from 02006 were also generated via medaka consensus with manual correction. Hypermutation of the provirus was initially identified by manually inspecting the reads in IGV, the consensus sequence of the provirus was checked for hypermutation with Hypermut (https://www.hiv.lanl.gov/content/sequence/HYPERMUT/hypermut.html). We determined if the proviral sequences were intact using the Gene Cutter tool (https://www.hiv.lanl.gov/content/sequence/GENE_CUTTER/cutter.html). Proviruses that did not contain a frameshift or stop codons not observed in the consensus sequence generated for patient 02006 were classified as intact. Deletions in the HIV-1 proviruses were identified by manual inspection of the integration site and proviral reads in IGV.

### Endogenous retroviruses

The sequence of bovine *APOB* ERV was generated by PCR amplifying the full-length ERV with LongAmp Taq DNA Polymerase (New England Biolabs) from a Holstein suffering from cholesterol deficiency. The resultant PCR product was sequenced on the Illumina platform as described below. It was also sequenced with an Oxford Nanopore MinION R7 flow cell as previously described [31]. Full-length sequence of the element was generated via manual curation. Guide RNAs and primer pairs were designed using this ERV reference. For the Ovine ERV, we used the published enJSRV-7 sequence [42] as a reference to design PCIP-seq guide RNAs and PCR primers. As the ovine and bovine genome contains sequences matching the ERV, mapping ERV PCIP-seq reads back to the reference genome creates a large pileup of reads in these regions. To avoid this, we first used BLAST [67] to identify the regions in the reference genome containing sequences matching the ERV, we then used BEDtools [68] to mask those regions. The appropriate ERV reference was then added as an additional chromosome in the reference.

### PCR validation and Illumina sequencing

Clone-specific PCR products were generated by placing primers in the flanking DNA as well as inside the provirus. LongAmp Taq DNA Polymerase (New England Biolabs) was used for amplification following the manufacturer’s guidelines, and resultant PCR products were sequenced (Additional file 1, Supplementary Methods). To examine SNPs in host DNA, the PCIP-seq libraries generated from the HIV-1 patients were sheared to ~ 400 bp followed by sequencing on an Illumina MiSeq instrument (Additional file 1, Supplementary Methods).

### BLV references

The sequence of the pBLV344 provirus was generated via a combination of Sanger and Illumina-based sequencing with manual curation of the sequence to produce a full-length proviral sequence. The consensus BLV sequences for the bovine samples 1439 and 1053 were generated by first mapping the PCIP-seq Nanopore reads to the pBLV344 provirus. We then used Nanopolish [69] to create an improved consensus. PCIP-seq libraries sequenced on the Illumina and Nanopore platform were mapped to this improved consensus visualized in IGV and manually corrected.

### Genome references

Sheep: OAR3.1 Cattle: UMD3.1 Human: hg38 For HTLV-1 integration sites hg19 was used. HPV18: GenBank: AY262282.1 Sequences of the proviruses can be found in Additional file 3.

## Supplementary Information

**Additional file 1:** Figure S1. Clonality pie charts in sheep and cattle. Figure S2. SNP validation by clone specific PCR. Figure S3. Distinguishing between real SNPs and technical artifacts. Figure S4. Hypermutation in BLV proviruses. Figure S5. Validation of BLV structural variants by clone specific PCR. Figure S6. SNPs and recombination in the HIV-1 cell line U1. Figure S7. Estimation of the efficiency of PCIP-seq. Figure S8. Examples of HIV-1 proviruses from patient 02006. Figure S9. Insertion site of ERV causing cholesterol deficiency in Holstein cattle. Figure S10. Validated BERVK2 identified via PCIP-seq. Figure S11. ERV BTA3_115.3 LTRs match APOB (BTA11_77.9) ERV. Figure S12. Validated enJSRV. Table S1. Comparing PCIP-seq to ligation mediated PCR and Illumina sequencing. Table S2. SNPs identified in each sample. Table S3. BLV structural variants identified by PCIP-seq. Table S4. HIV-1 patients’ clinical information. Table S5. BERVK2s identified in cattle by PCIP-seq. Table S6. enJSRVs identified in sheep by PCIP-seq. Table S7. HPV integration sites identified in patients HPV18_PX and HPV18_PY. Table S8. PCIP-seq efficiency estimation in BLV. Supplementary note 1. Rationale behind the use of CRISPR-cas9 to cleave circular DNA. Supplementary note 2. Effect of coverage on SNP calling. Supplementary Methods. Supplementary References.

**Additional file 2:** Dataset S1. HIV-1 integration sites identified in patients 02006 and 06042.

**Additional file 3:** Dataset S2. Provirus consensus sequences.

**Additional file 4:** Dataset S3. PCIP-seq, ddPCR and validation oligos.

**Additional file 5:** Review history.

## Data Availability

Sequence data that support the findings of this study have been deposited in the European Nucleotide Archive (ENA) at EMBL-EBI under accession number PRJEB34495: https://www.ebi.ac.uk/ena/browser/view/PRJEB34495 [70]. All other relevant data are available within the article, its supplementary files, or from the corresponding author upon reasonable request. The code and detailed outline of the PCIP-seq analysis workflow are available on Github: https://github.com/GIGA-AnimalGenomics-BLV/PCIP. The DOI for the source code version used in this paper is available on Zenodo: 10.5281/zenodo.4543265 [71].

## References

[1] Bushman F, Lewinski M, Ciuffi A, Barr S, Leipzig J, Hannenhalli S (2005). Genome-wide analysis of retroviral DNA integration. Nat Rev Micro.

[2] Gillet NA, Malani N, Melamed A, Gormley N, Carter R, Bentley D (2011). The host genomic environment of the provirus determines the abundance of HTLV-1-infected T-cell clones. Blood..

[3] Maldarelli F, Wu X, Su L, Simonetti FR, Shao W, Hill S (2014). Specific HIV integration sites are linked to clonal expansion and persistence of infected cells. Science..

[4] Wagner TA, McLaughlin S, Garg K, Cheung CYK, Larsen BB, Styrchak S (2014). HIV latency. Proliferation of cells with HIV integrated into cancer genes contributes to persistent infection. Science..

[5] Bruner KM, Wang Z, Simonetti FR, Bender AM, Kwon KJ, Sengupta S (2019). A quantitative approach for measuring the reservoir of latent HIV-1 proviruses. Nature..

[6] Einkauf KB, Lee GQ, Gao C, Sharaf R, Sun X, Hua S (2019). Intact HIV-1 proviruses accumulate at distinct chromosomal positions during prolonged antiretroviral therapy. J Clin Invest.

[7] Rosewick N, Durkin K, Artesi M, Marcais A, Hahaut V, Griebel P (2017). Cis-perturbation of cancer drivers by the HTLV-1/BLV proviruses is an early determinant of leukemogenesis. Nat Commun.

[8] Malhotra S, Winans S, Lam G, Justice J, Morgan R, Beemon K (2017). Selection for avian leukosis virus integration sites determines the clonal progression of B-cell lymphomas. Bangham CRM, editor. Plos Pathog.

[9] Simonetti FR, Sobolewski MD, Fyne E, Shao W, Spindler J, Hattori J (2016). Clonally expanded CD4 +T cells can produce infectious HIV-1 in vivo. PNAS..

[10] Miyazaki M, Yasunaga J-I, Taniguchi Y, Tamiya S, Nakahata T, Matsuoka M (2007). Preferential selection of human T-cell leukemia virus type 1 provirus lacking the 5′ long terminal repeat during oncogenesis. J Virol.

[11] Katsuya H, Islam S, Tan BJY, Ito J, Miyazato P, Matsuo M, et al. The nature of the HTLV-1 provirus in naturally infected individuals analyzed by the viral DNA- capture-Seq approach. Cell Rep 2019;29:724–4. 10.1016/j.celrep.2019.09.016 31618639

[12] Hiener B, Horsburgh BA, Eden J-S, Barton K, Schlub TE, Lee E (2017). Identification of genetically intact HIV-1 proviruses in specific CD4+ T cells from effectively treated participants. Cell Rep.

[13] Patro SC, Brandt LD, Bale MJ, Halvas EK, Joseph KW, Shao W (2019). Combined HIV-1 sequence and integration site analysis informs viral dynamics and allows reconstruction of replicating viral ancestors. PNAS..

[14] Lander ES, Linton LM, Birren B, Nusbaum C, Zody MC, Baldwin J (2001). Initial sequencing and analysis of the human genome. Nature..

[15] Rivas-Carrillo SD, Pettersson ME, Rubin C-J, Jern P (2018). Whole-genome comparison of endogenous retrovirus segregation across wild and domestic host species populations. PNAS..

[16] Pett M, Coleman N (2007). Integration of high-risk human papillomavirus: a key event in cervical carcinogenesis?. J Pathol.

[17] Hu Z, Zhu D, Wang W, Li W, Jia W, Zeng X (2015). Genome-wide profiling of HPV integration in cervical cancer identifies clustered genomic hot spots and a potential microhomology-mediated integration mechanism. Nat Genet.

[18] Groves IJ, Coleman N (2018). Human papillomavirus genome integration in squamous carcinogenesis: what have next-generation sequencing studies taught us?. J Pathol.

[19] Sedlazeck FJ, Lee H, Darby CA, Schatz MC (2018). Piercing the dark matter: bioinformatics of long-range sequencing and mapping. Nat Rev Genet.

[20] Pradhan B, Cajuso T, Katainen R, Sulo PXI, Tanskanen T, Kilpivaara O (2017). Detection of subclonal L1 transductions in colorectal cancer by long-distance inverse-PCR and Nanopore sequencing. Sci Rep.

[21] Löber U, Hobbs M, Dayaram A, Tsangaras K, Jones K, Alquezar-Planas DE (2018). Degradation and remobilization of endogenous retroviruses by recombination during the earliest stages of a germ-line invasion. PNAS..

[22] Artesi M, Marçais A, Durkin K, Rosewick N, Hahaut V, Suarez F (2017). Monitoring molecular response in adult T-cell leukemia by high-throughput sequencing analysis of HTLV-1 clonality. Leukemia..

[23] Willems L, Kettmann R, Dequiedt F, Portetelle D, Vonèche V, Cornil I (1993). In vivo infection of sheep by bovine leukemia virus mutants. J Virol.

[24] Wilm A, Aw PPK, Bertrand D, Yeo GHT, Ong SH, Wong CH (2012). LoFreq: a sequence-quality aware, ultra-sensitive variant caller for uncovering cell-population heterogeneity from high-throughput sequencing datasets. Nucleic Acids Res.

[25] Van den Broeke A, Bagnis C, Ciesiolka M, Cleuter Y, Gelderblom H, Kerkhofs P (1999). In vivo rescue of a silent tax-deficient bovine leukemia virus from a tumor-derived ovine B-cell line by recombination with a retrovirally transduced wild-type tax gene. J Virol.

[26] Merimi M, Klener P, Szynal M, Cleuter Y, Bagnis C, Kerkhofs P (2007). Complete suppression of viral gene expression is associated with the onset and progression of lymphoid malignancy: observations in bovine leukemia virus-infected sheep. Retrovirology..

[27] Armitage AE, Deforche K, Chang C-H, Wee E, Kramer B, Welch JJ (2012). APOBEC3G-induced hypermutation of human immunodeficiency virus type-1 is typically a discrete “all or nothing” phenomenon. Worobey M, editor. Plos Genet.

[28] Samuel CE (2011). Adenosine deaminases acting on RNA (ADARs) are both antiviral and proviral. Virology..

[29] Cachat A, Alais S, Chevalier SA, Journo C, Fusil F, Dutartre H, et al. ADAR1 enhances HTLV-1 and HTLV-2 replication through inhibition of PKR activity. Retrovirology. 2014;11:7415–5. 10.1186/s12977-014-0093-9 PMC4245799 25389016

[30] Rosewick N, Momont M, Durkin K, Takeda H, Caiment F, Cleuter Y (2013). Deep sequencing reveals abundant noncanonical retroviral microRNAs in B-cell leukemia/lymphoma. PNAS..

[31] Durkin K, Rosewick N, Artesi M, Hahaut V, Griebel P, Arsic N (2016). Characterization of novel bovine leukemia virus (BLV) antisense transcripts by deep sequencing reveals constitutive expression in tumors and transcriptional interaction with viral microRNAs. Retrovirology..

[32] Finzi D, Blankson J, Siliciano JD, Margolick JB, Chadwick K, Pierson T (1999). Latent infection of CD4+ T cells provides a mechanism for lifelong persistence of HIV-1, even in patients on effective combination therapy. Nat Med.

[33] Anderson EM, Maldarelli F (2018). The role of integration and clonal expansion in HIV infection: live long and prosper. Retrovirology..

[34] Kiselinova M, De Spiegelaere W, Buzon MJ, Malatinkova E, Lichterfeld M, Vandekerckhove L (2016). Integrated and Total HIV-1 DNA predict ex vivo viral outgrowth. Swanstrom R, editor. PLoS Pathog.

[35] Folks TM, Justement J, Kinter A, Dinarello CA, Fauci AS (1987). Cytokine-induced expression of HIV-1 in a chronically infected promonocyte cell line. Science..

[36] Symons J, Chopra A, Malantinkova E, Spiegelaere W, Leary S, Cooper D (2017). HIV integration sites in latently infected cell lines: evidence of ongoing replication. Retrovirology..

[37] Emiliani S, Fischle W, Ott M, Van Lint C, Amella CA, Verdin E (1998). Mutations in the tat gene are responsible for human immunodeficiency virus type 1 postintegration latency in the U1 cell line. J Virol.

[38] Cesana D, de Sio FRS, Rudilosso L, Gallina P, Calabria A, Beretta S (2017). HIV-1-mediated insertional activation of STAT5B and BACH2 trigger viral reservoir in T regulatory cells. Nat Commun.

[39] Hughes JF, Coffin JM (2004). Human endogenous retrovirus K solo-LTR formation and insertional polymorphisms: implications for human and viral evolution. PNAS..

[40] Cornelis G, Heidmann O, Degrelle SA, Vernochet C, Lavialle C, Letzelter C (2013). Captured retroviral envelope syncytin gene associated with the unique placental structure of higher ruminants. PNAS..

[41] Menzi F, Besuchet-Schmutz N, Fragnière M, Hofstetter S, Jagannathan V, Mock T (2016). A transposable element insertion in APOB causes cholesterol deficiency in Holstein cattle. Anim Genet.

[42] Arnaud F, Caporale M, Varela M, Biek R, Chessa B, Alberti A (2007). A paradigm for virus–host coevolution: sequential counter-adaptations between endogenous and exogenous retroviruses. Plos Pathog.

[43] Schiffman M, Castle PE, Jeronimo J, Rodriguez AC, Wacholder S (2007). Human papillomavirus and cervical cancer. Lancet.

[44] Akagi K, Li J, Broutian TR, Padilla-Nash H, Xiao W, Jiang B (2014). Genome-wide analysis of HPV integration in human cancers reveals recurrent, focal genomic instability. Genome Res.

[45] Varela I, Tarpey P, Raine K, Huang D, Ong CK, Stephens P (2011). Exome sequencing identifies frequent mutation of the SWI/SNF complex gene PBRM1 in renal carcinoma. Nature..

[46] Jiao Y, Pawlik TM, Anders RA, Selaru FM, Streppel MM, Lucas DJ (2013). Exome sequencing identifies frequent inactivating mutations in BAP1, ARID1A and PBRM1 in intrahepatic cholangiocarcinomas. Nat Genet.

[47] Quick J, Duraffour S, Simpson JT, Severi E, Cowley L, Bore JA (2016). Real-time, portable genome sequencing for Ebola surveillance. Nature..

[48] Bangham CRM, Human T (2018). Cell leukemia virus type 1: persistence and pathogenesis. Annu Rev Immunol.

[49] Goodwin S, Gurtowski J, Ethe-Sayers S, Deshpande P, Schatz MC, McCombie WR (2015). Oxford Nanopore sequencing, hybrid error correction, and de novo assembly of a eukaryotic genome. Genome Res.

[50] Wick R (2019). Performance of neural network basecalling tools for Oxford Nanopore sequencing. Genome Biol Genome Biol.

[51] Greig DR, Jenkins C, Gharbia S, Dallman TJ (2019). Comparison of single-nucleotide variants identified by Illumina and Oxford Nanopore technologies in the context of a potential outbreak of Shiga toxin–producing Escherichia coli. GigaScience..

[52] R10.3: the newest nanopore for high accuracy nanopore sequencing. nanoporetech.com. 2020. Available from: https://nanoporetech.com/about-us/news/r103-newest-nanopore-high-accuracy-nanopore-sequencing-now-available-store. [cited 2020 Nov 26]

[53] Wenger AM, Peluso P, Rowell WJ, Chang P-C, Hall RJ, Concepcion GT (2019). Accurate circular consensus long-read sequencing improves variant detection and assembly of a human genome. Nat Biotechnol.

[54] Gillet N, Florins A, Boxus M, Burteau C, Nigro A, Vandermeers F (2007). Mechanisms of leukemogenesis induced by bovine leukemia virus: prospects for novel anti-retroviral therapies in human. Retrovirology..

[55] Ko NL, Birlouez E, Wain-Hobson S, Mahieux R, Vartanian JP (2012). Hyperediting of human T-cell leukemia virus type 2 and simian T-cell leukemia virus type 3 by the dsRNA adenosine deaminase ADAR-1. J Gen Virol.

[56] Iwase SC, Miyazato P, Katsuya H, Islam S, Yang BTJ, Ito J (2019). HIV-1 DNA-capture-seq is a useful tool for the comprehensive characterization of HIV-1 provirus. Sci Rep.

[57] Jiang C, Lian X, Gao C, Sun X, Einkauf KB, Chevalier JM (2020). Distinct viral reservoirs in individuals with spontaneous control of HIV-1. Nature..

[58] Schiffman M, Doorbar J, Wentzensen N, de Sanjose S, Fakhry C, Monk BJ (2016). Carcinogenic human papillomavirus infection. Nat Rev Dis Prim.

[59] Zhao L-H, Liu X, Yan H-X, Li W-Y, Zeng X, Yang Y (2016). Genomic and oncogenic preference of HBV integration in hepatocellular carcinoma. Nat Commun.

[60] Goodwin LO, Splinter E, Davis TL, Urban R, He H, Braun RE (2019). Large-scale discovery of mouse transgenic integration sites reveals frequent structural variation and insertional mutagenesis. Genome Res.

[61] Li H (2018). Minimap2: pairwise alignment for nucleotide sequences. Bioinformatics..

[62] Rutsaert S, De Spiegelaere W, De Clercq L, Vandekerckhove L (2019). Evaluation of HIV-1 reservoir levels as possible markers for virological failure during boosted darunavir monotherapy. J Antimicrob Chemother.

[63] Trypsteen W, Vynck M, De Neve J, Bonczkowski P, Kiselinova M, Malatinkova E (2015). ddpcRquant: threshold determination for single channel droplet digital PCR experiments. Anal Bioanal Chem.

[64] Li H, Handsaker B, Wysoker A, Fennell T, Ruan J, Homer N (2009). The Sequence Alignment/Map format and SAMtools. Bioinformatics..

[65] Killick R, Fearnhead P, Eckley IA (2012). Optimal detection of changepoints with a linear computational cost. J Am Stat Assoc.

[66] Thorvaldsdóttir H, Robinson JT, Mesirov JP (2013). Integrative Genomics Viewer (IGV): high-performance genomics data visualization and exploration. Brief Bioinform.

[67] Camacho C, Coulouris G, Avagyan V, Ma N, Papadopoulos J, Bealer K (2009). BLAST+: architecture and applications. BMC Bioinformatics.

[68] Quinlan AR, Hall IM (2010). BEDTools: a flexible suite of utilities for comparing genomic features. Bioinformatics..

[69] Loman NJ, Quick J, Simpson JT (2015). A complete bacterial genome assembled de novo using only nanopore sequencing data. Nat Methods.

[70] Artesi M, Hahaut V, Cole B, Lambrechts L, Ashrafi F, Marçais A, Hermine O, Griebel P, Arsic N, van der Meer F, Burny A, Bron D, Bianchi E, Delvenne P, Bours V, Charlier C, Georges M, Vandekerckhove L, Van den Broeke A, Durkin K. PCIP-seq: simultaneous sequencing of integrated viral genomes and their insertion sites with long reads. ENA EMBL. 2021. https://www.ebi.ac.uk/ena/browser/view/PRJEB34495. Accessed 25 Feb 2021. 10.1186/s13059-021-02307-0 PMC8025556 33823910

[71] Artesi M, Hahaut V, Cole B, Lambrechts L, Ashrafi F, Marçais A, Hermine O, Griebel P, Arsic N, van der Meer F, Burny A, Bron D, Bianchi E, Delvenne P, Bours V, Charlier C, Georges M, Vandekerckhove L, Van den Broeke A, Durkin K. PCIP-seq: simultaneous sequencing of integrated viral genomes and their insertion sites with long reads (version v1.0.0). Github. 2021. doi: 10.5281/zenodo.4543265. 10.1186/s13059-021-02307-0 PMC8025556 33823910

