# Supplementary material for: PCIP-seq: simultaneous sequencing of integrated viral genomes and their insertion sites with long reads
Source: Genome Biol. 2021 Apr 6;22:97. doi: 10.1186/s13059-021-02307-0 (PMC8025556; doi:10.1186/s13059-021-02307-0)
Supplement: Supplementary file 1 — Additional file 1: Figure S1. Clonality pie charts in sheep and cattle. Figure S2. SNP validation by clone specific PCR. Figure S3. Distinguishing between real SNPs and technical artifacts. Figure S4. Hypermutation in BLV proviruses. Figure S5. Validation of BLV structural variants by clone specific PCR. Figure S6. SNPs and recombination in the HIV-1 cell line U1. Figure S7. Estimation of the efficiency of PCIP-seq. Figure S8. Examples of HIV-1 proviruses from patient 02006. Figure S9. Insertion site of ERV causing cholesterol deficiency in Holstein cattle. Figure S10. Validated BERVK2 identified via PCIP-seq. Figure S11. ERV BTA3_115.3 LTRs match APOB (BTA11_77.9) ERV. Figure S12. Validated enJSRV. Table S1. Comparing PCIP-seq to ligation mediated PCR and Illumina sequencing. Table S2. SNPs identified in each sample. Table S3. BLV structural variants identified by PCIP-seq. Table S4. HIV-1 patients’ clinical information. Table S5. BERVK2s identified in cattle by PCIP-seq. Table S6. enJSRVs identified in sheep by PCIP-seq. Table S7. HPV integration sites identified in patients HPV18_PX and HPV18_PY. Table S8. PCIP-seq efficiency estimation in BLV. Supplementary note 1. Rationale behind the use of CRISPR-cas9 to cleave circular DNA. Supplementary note 2. Effect of coverage on SNP calling. Supplementary Methods. Supplementary References. [file 13059_2021_2307_MOESM1_ESM.pdf]

Supplementary Information for:

PCIP-seq: simultaneous sequencing of integrated viral genomes and their insertion sites with long reads

Maria Artesi, Vincent Hahaut, Basiel Cole, Laurens Lambrechts, Fereshteh Ashrafi, Ambroise Marçais, Olivier Hermine, Philip Griebel, Natasa Arsic, Frank van der Meer, Arsène Burny, Dominique Bron, Elettra Bianchi, Philippe Delvenne, Vincent Bours, Carole Charlier, Michel Georges, Linos Vandekerckhove, Anne Van den Broeke, Keith Durkin

Corresponding author
Anne Van den Broeke
anne.vandenbroeke@bordet.be

This PDF file includes:

Figures S1 to S12
Tables S1 to S8
Supplementary text:
- Supplementary note 1: Rationale behind the use of CRISPR-cas9 to cleave circular DNA
- Supplementary note 2: Effect of coverage on SNP calling
- Supplementary Methods
- Supplementary References

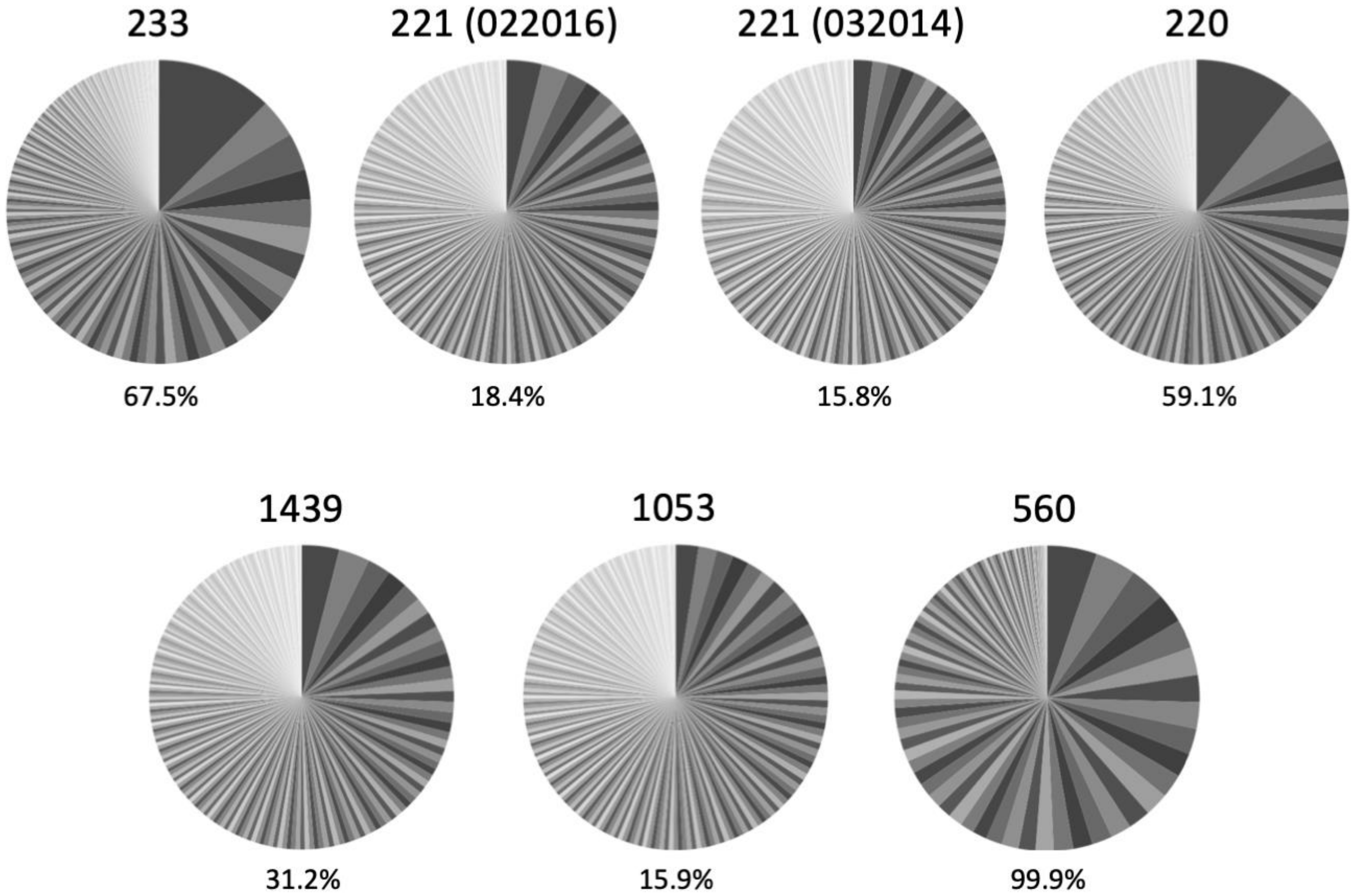

Fig. S1 Pie charts showing the relative abundance of the 200 largest clones in the four sheep (top) and three cattle (bottom) infected with BLV, each slice of the pie represents a single insertion site, the % below indicated what fraction of the overall reads these 200 clones represent.

Ovine 221 (022016) & 221 (032014)
BLV SNPs validated via clone specific PCR

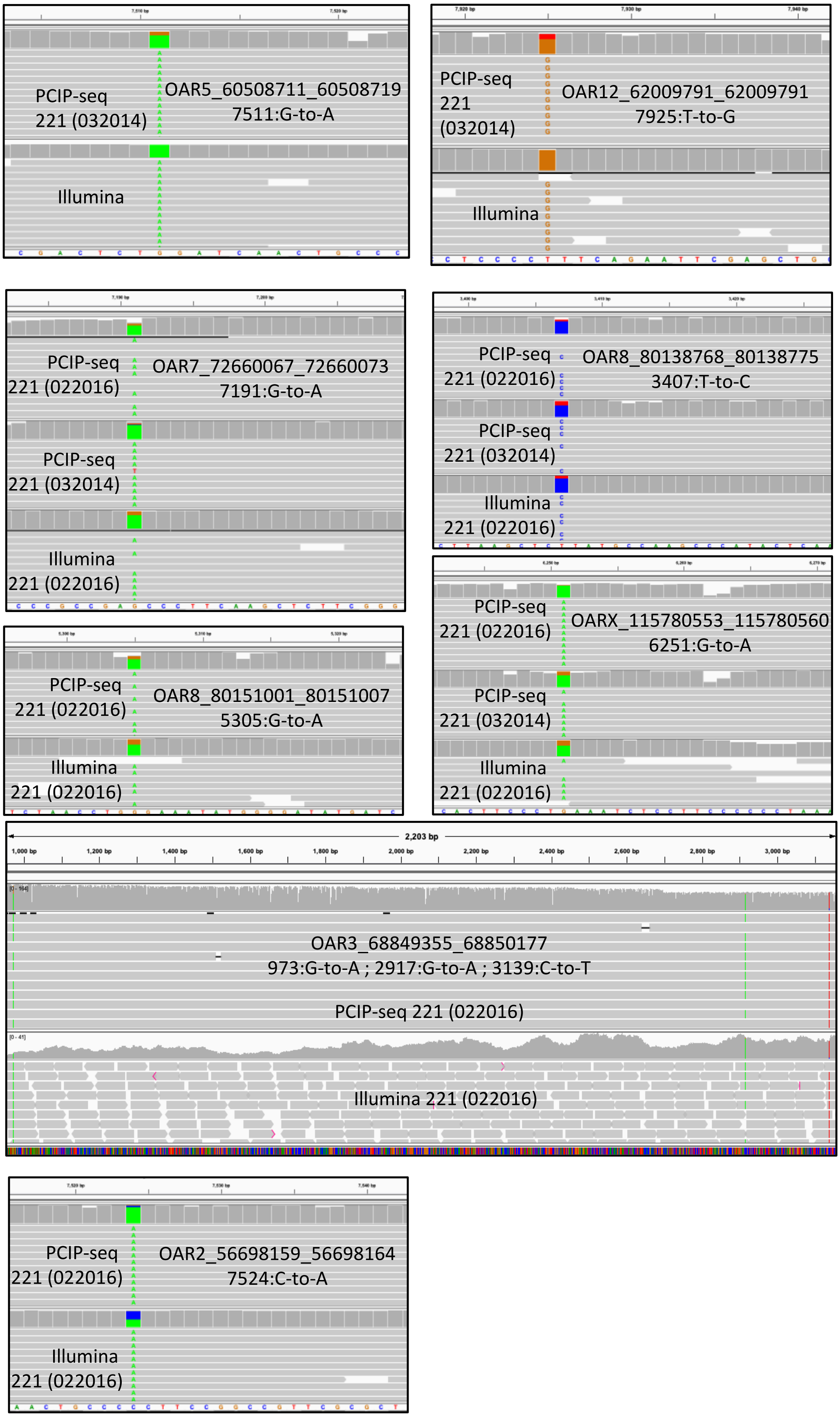

Fig. S2 SNPs identified by PCIP-seq in BLV validated by clone specific PCR. These SNPs came from eighteen proviruses, 10 from cattle, 8 from sheep.

Bovine 1439 BLV SNPs validated via clone specific PCR

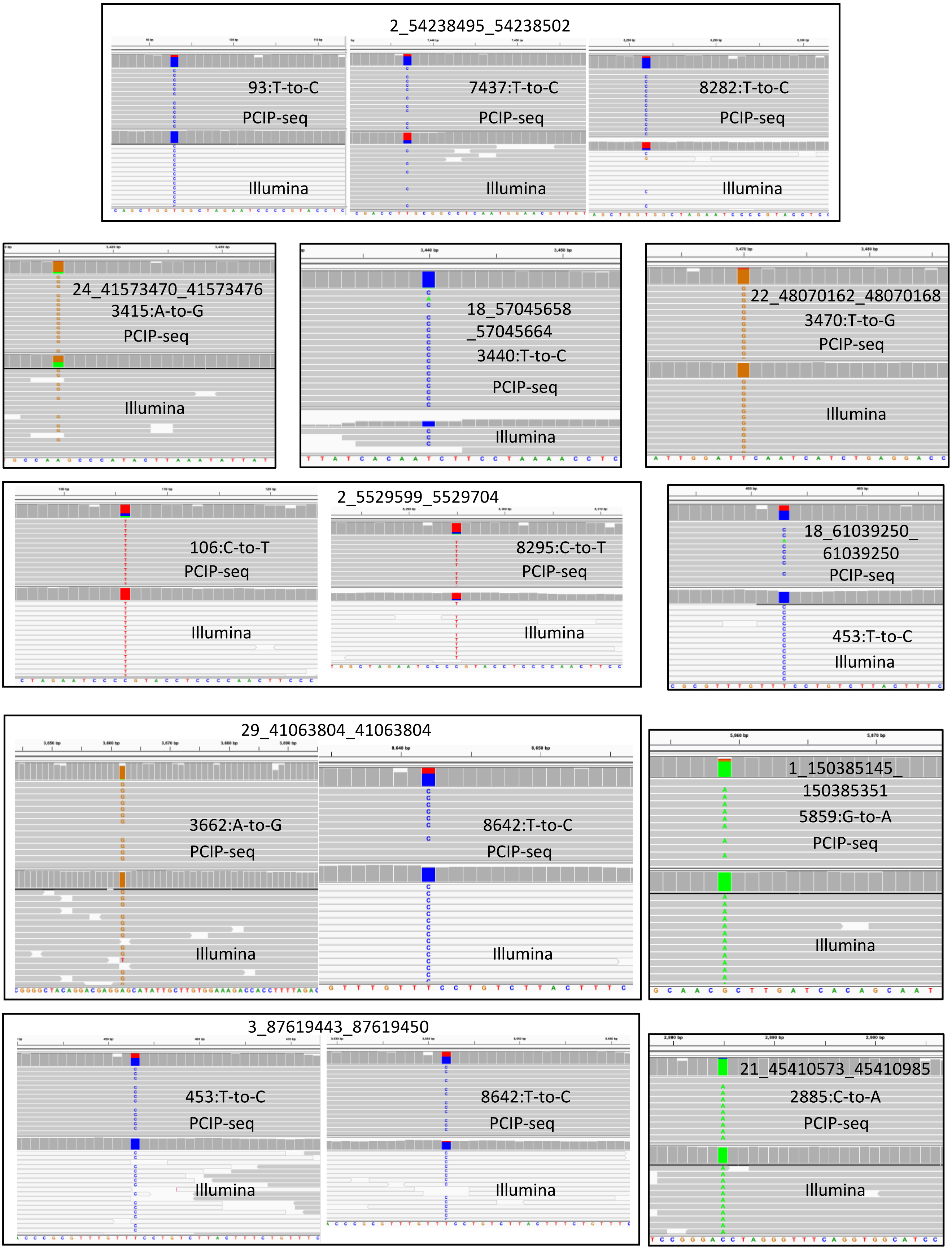

Fig. S2 continued

a

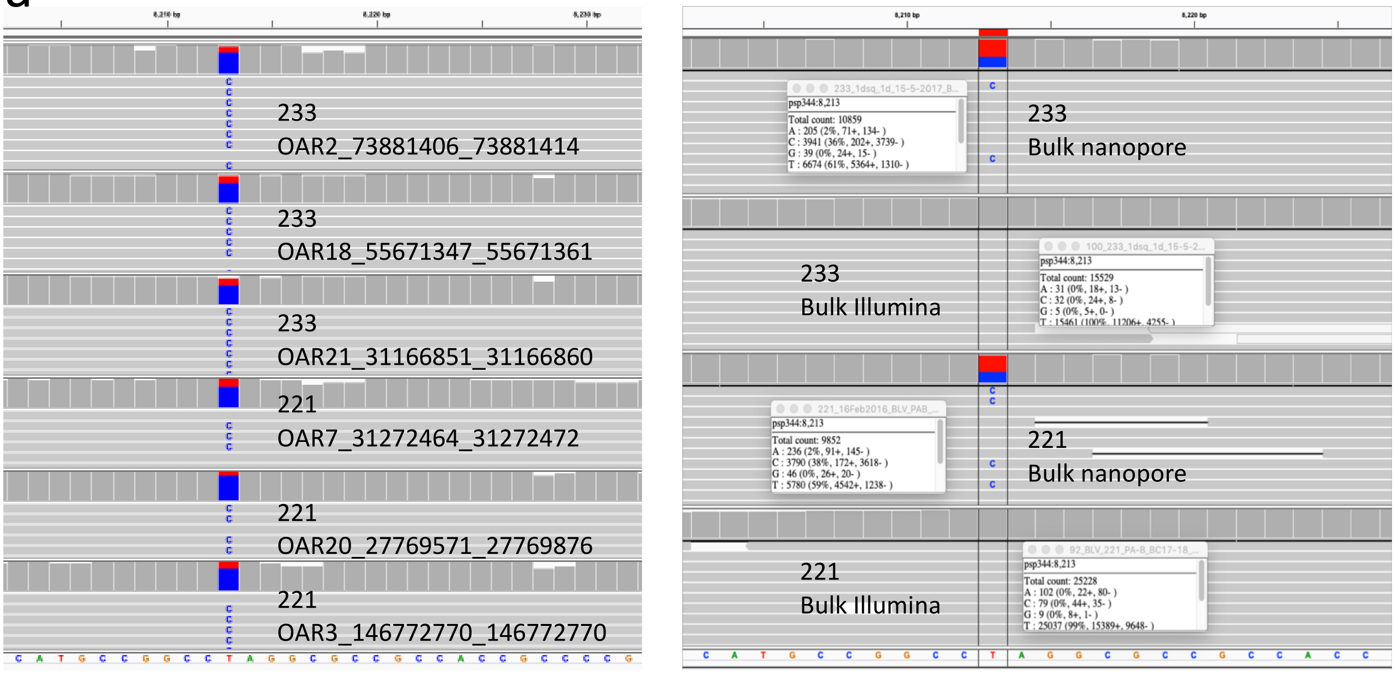

b Ovine 233

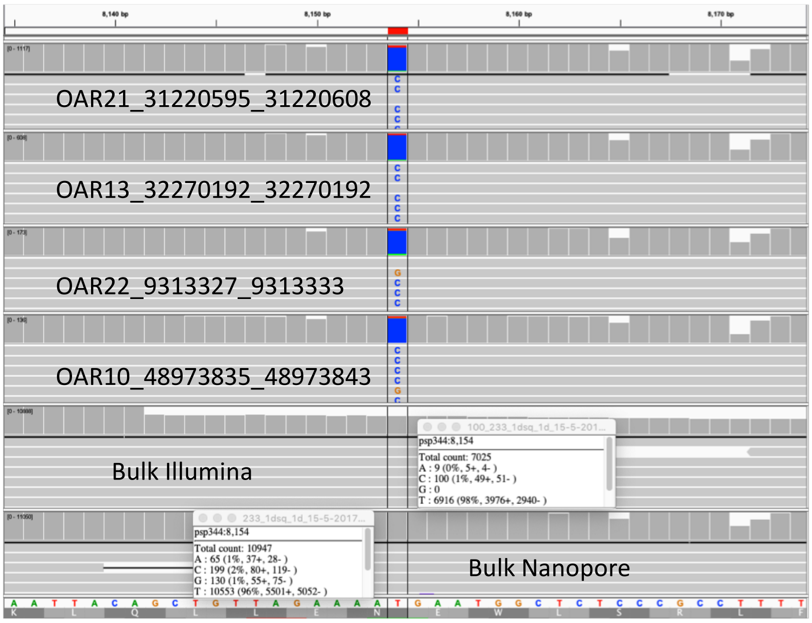

Fig. S3 Distinguishing between real SNPs and technical artifacts **(a)** We observed a number of BLV proviruses in all the samples that had an apparent SNP at position 8213. Shown are three examples from sheep 233 and 211. When we looked at this position in reads mapped to the provirus without first sorting based on insertion site (referred to as bulk) we saw a C called 36 and 38% of the time respectively in the Nanopore data. In the bulk Illumina data, generated from the same sample, we saw the C is called 0% of the time indicating a technical artifact. As a consequence, SNPs from this position were excluded. **(b)** In animal 233 we found 16 proviruses (provirus inclusion was based on the less stringent criteria of >10 reads covering the position, not filtered for PCR duplicates) carrying a T-to-C transition within the Tax ORF at position 8154, this variant does not change the amino acid. Shown are screen captures for 4 of the proviruses carrying the SNP, Illumina and Nanopore bulk sequencing from the same sample show C is called at a 2% frequency in Nanopore, while with Illumina C is called at a 1% frequency. This indicates that the SNPs observed in these proviruses are not a technical artifact.

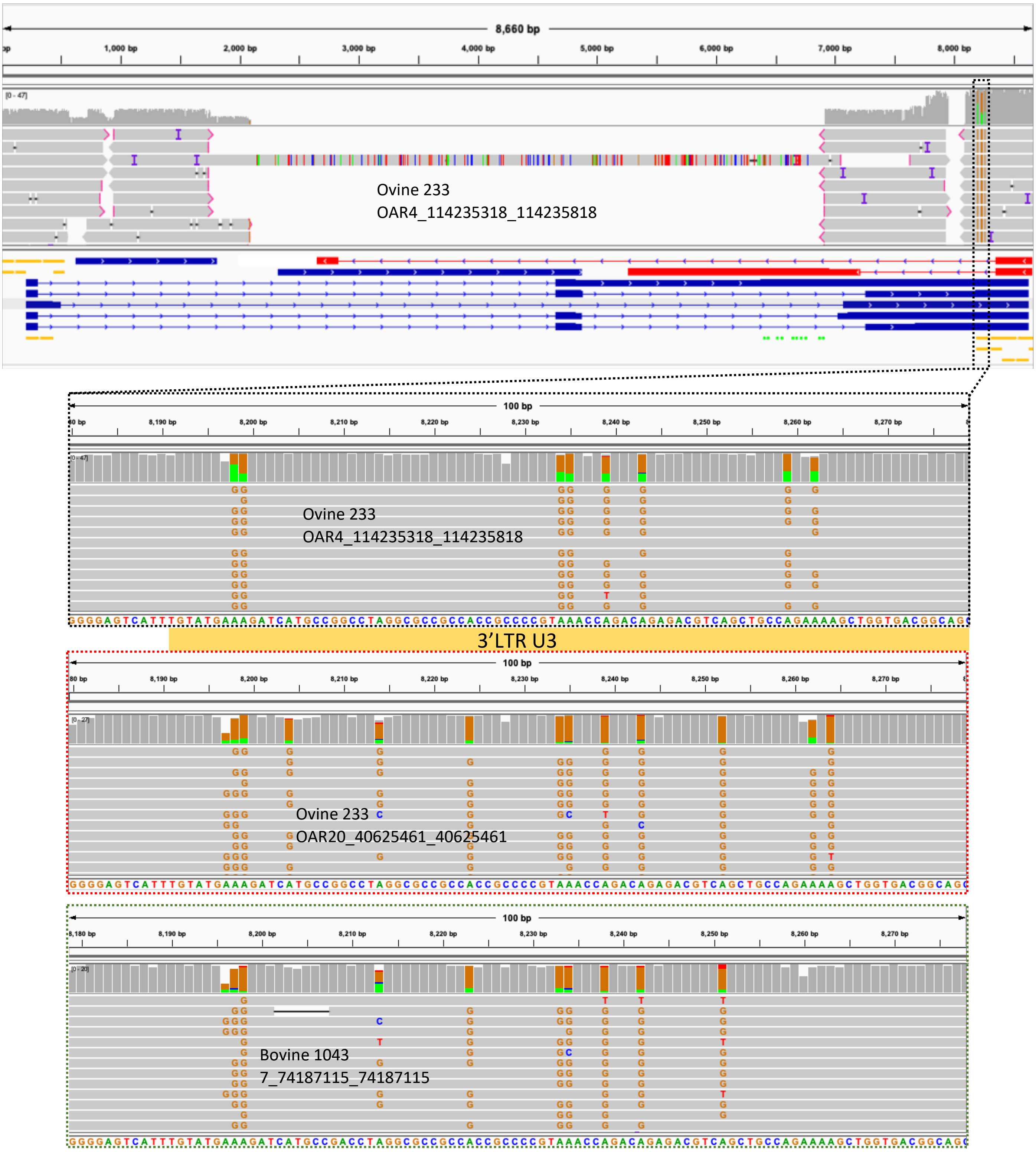

Fig. S4 Hypermutation of a ~70bp region in U3 of the 3’LTR of BLV proviruses.

Ovine 221 (022016) & 221 (032014)
BLV SVs validated by clone specific PCR

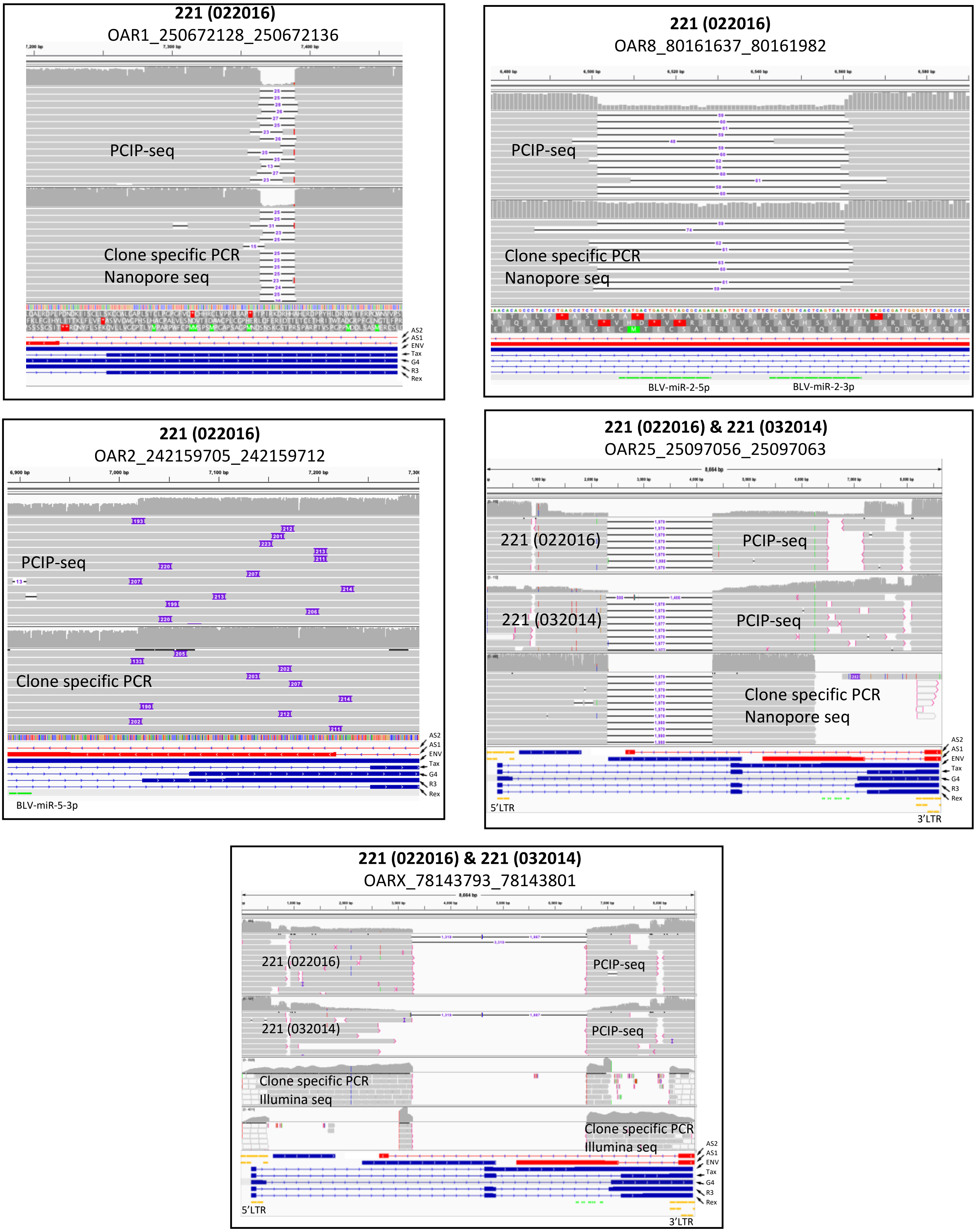

Fig. S5 Clone specific PCR to validate BLV structural variants. These CNVs are from fourteen proviruses, 7 from cattle, 7 from sheep. One of the sheep proviruses was also validated for a SNP.

Ovine 233
BLV SVs validated by clone specific PCR

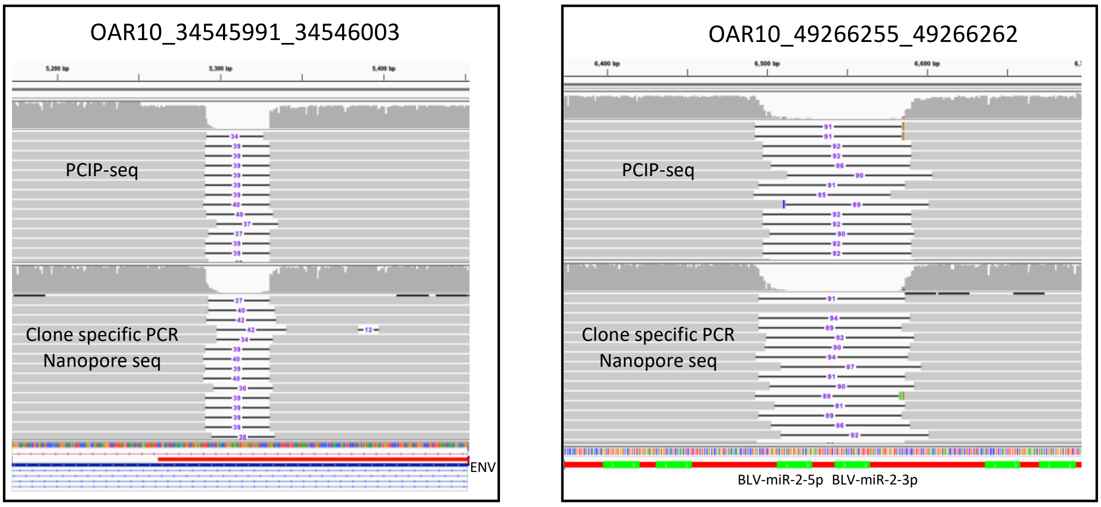

Bovine 1439
BLV SVs validated by clone specific PCR

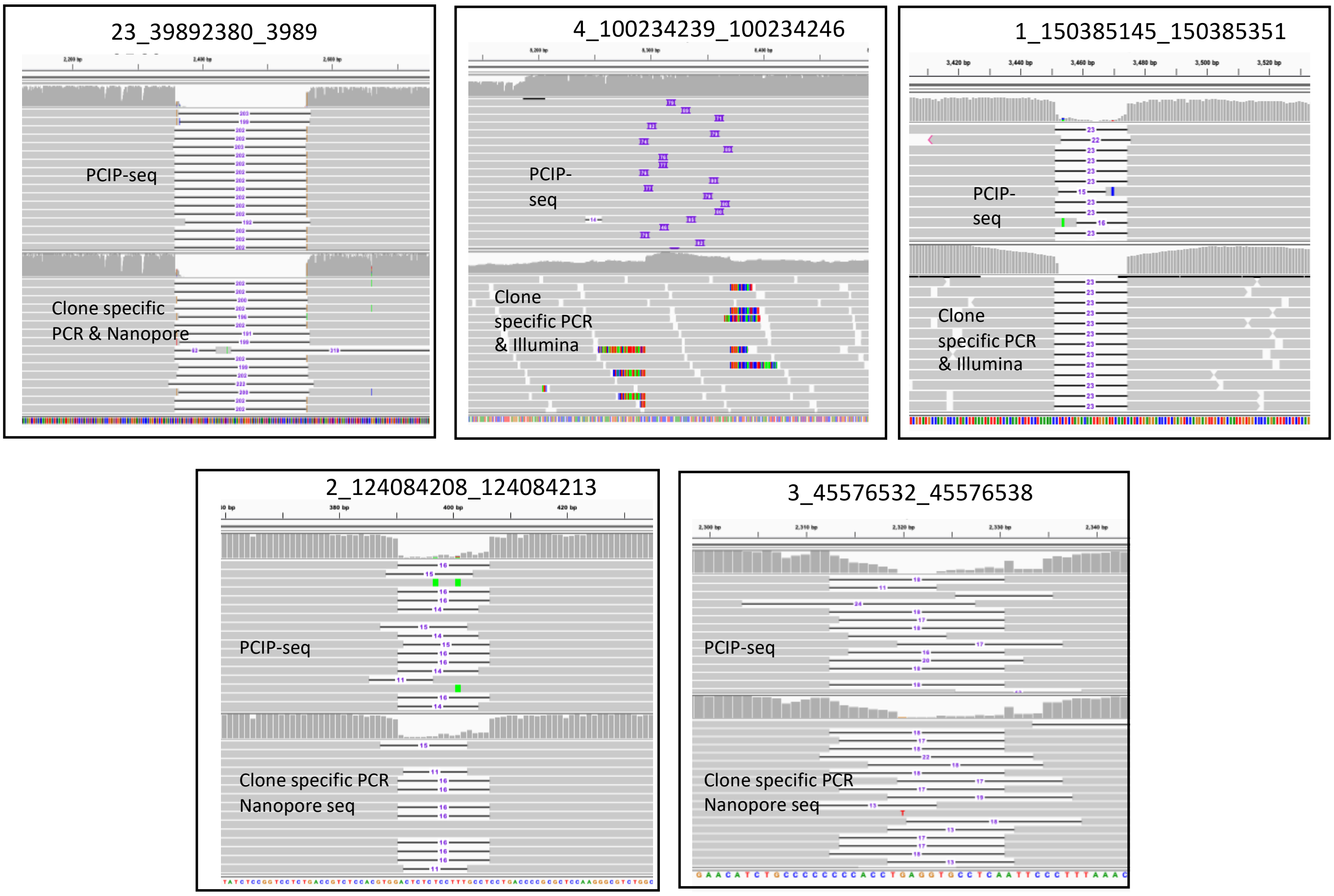

Fig. S5 continued

Bovine 1439
BLV 5' deletions validated by clone specific PCR

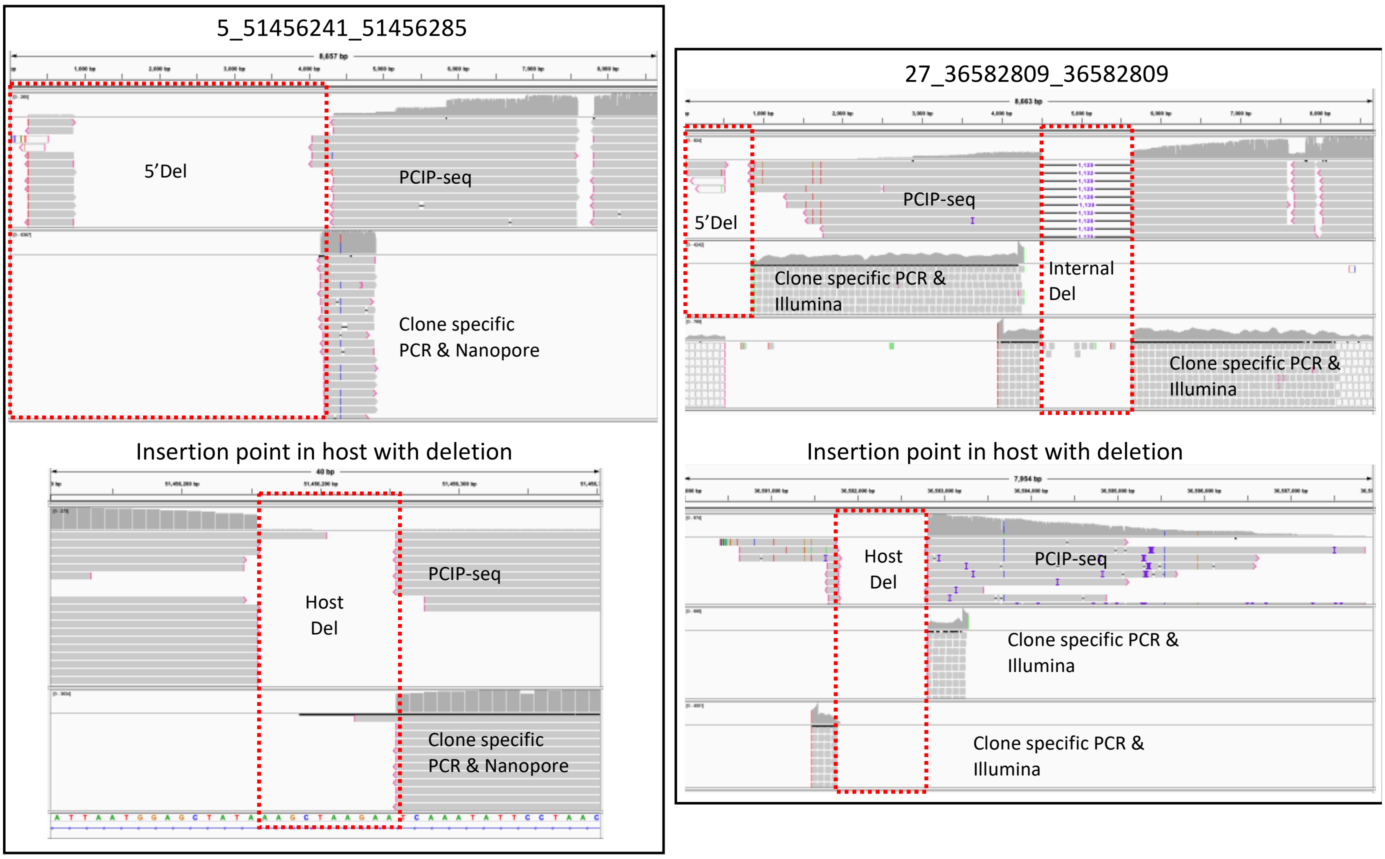

Fig. S5 continued

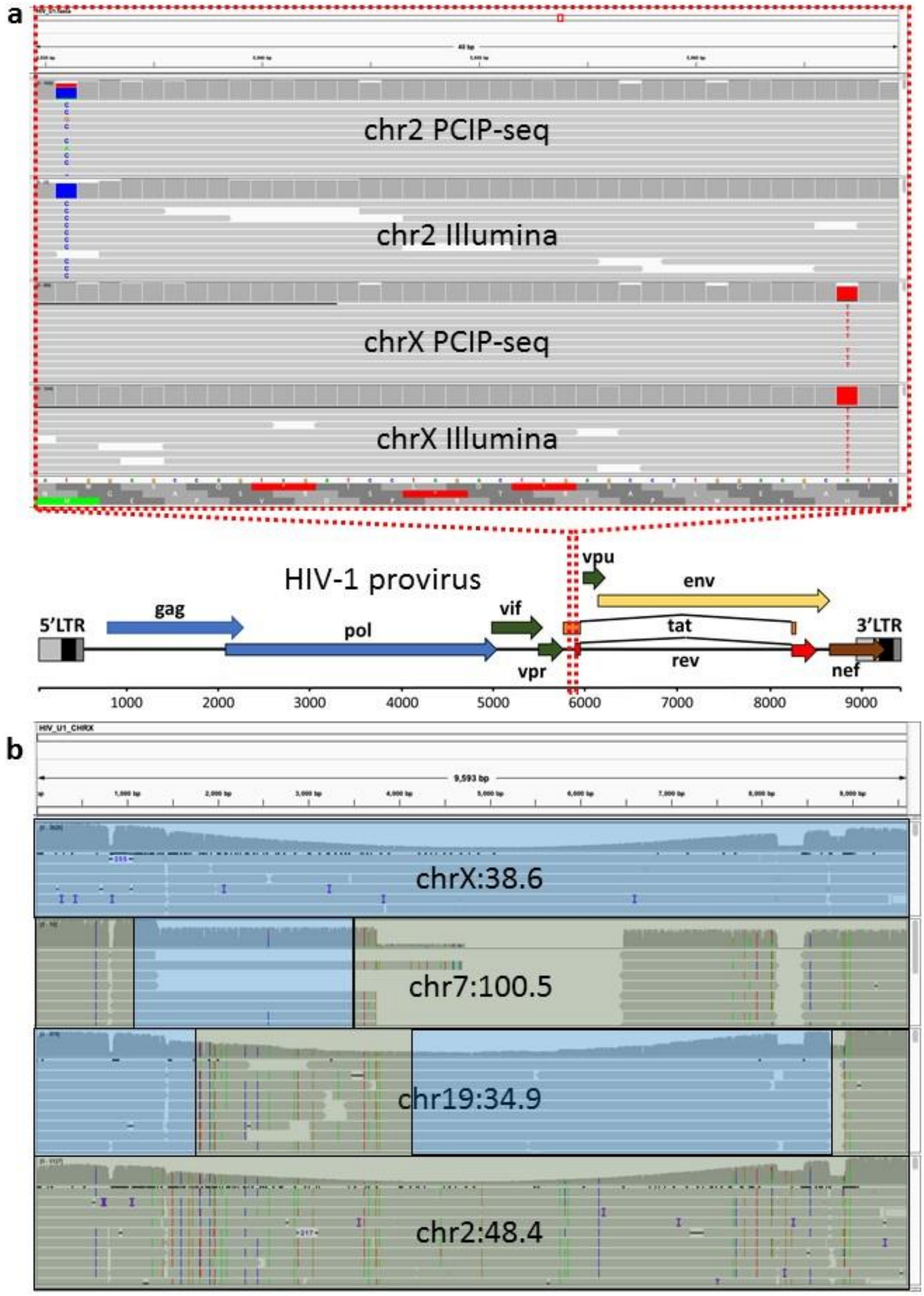

Fig. S6 SNPs and recombination observed in the HIV-1 cell line U1 (**a**) Screen shot from IGV, representative PCIP-seq reads and clone specific PCR products sequenced on Illumina. This region corresponds to the first 13 amino acids of the Tat protein. In the chr2 provirus a T-to-C changes ATG to ACG and the first methionine to a threonine. In the chrX provirus an A-to-T changes CAT to CTT replacing a histidine at position 13 with a leucine. (**b**) Two proviruses, chr7:100.5 & chr19:34.9 identified as the products of recombination between major chrX and chr2 proviruses. IGV screen shot shows proviral reads from all four proviruses mapped to a full length proviral genome (the sequence of the chrX provirus was used as the reference). The colored vertical lines indicate SNPs and identify sequences derived from the chr2 provirus, highlighted in green. Sequences originating from the chrX provirus is highlighted in blue. Sequences of the provirus from chr19 and chr7 that match either chrX or chr2 provirus are highlighted in the appropriate color.

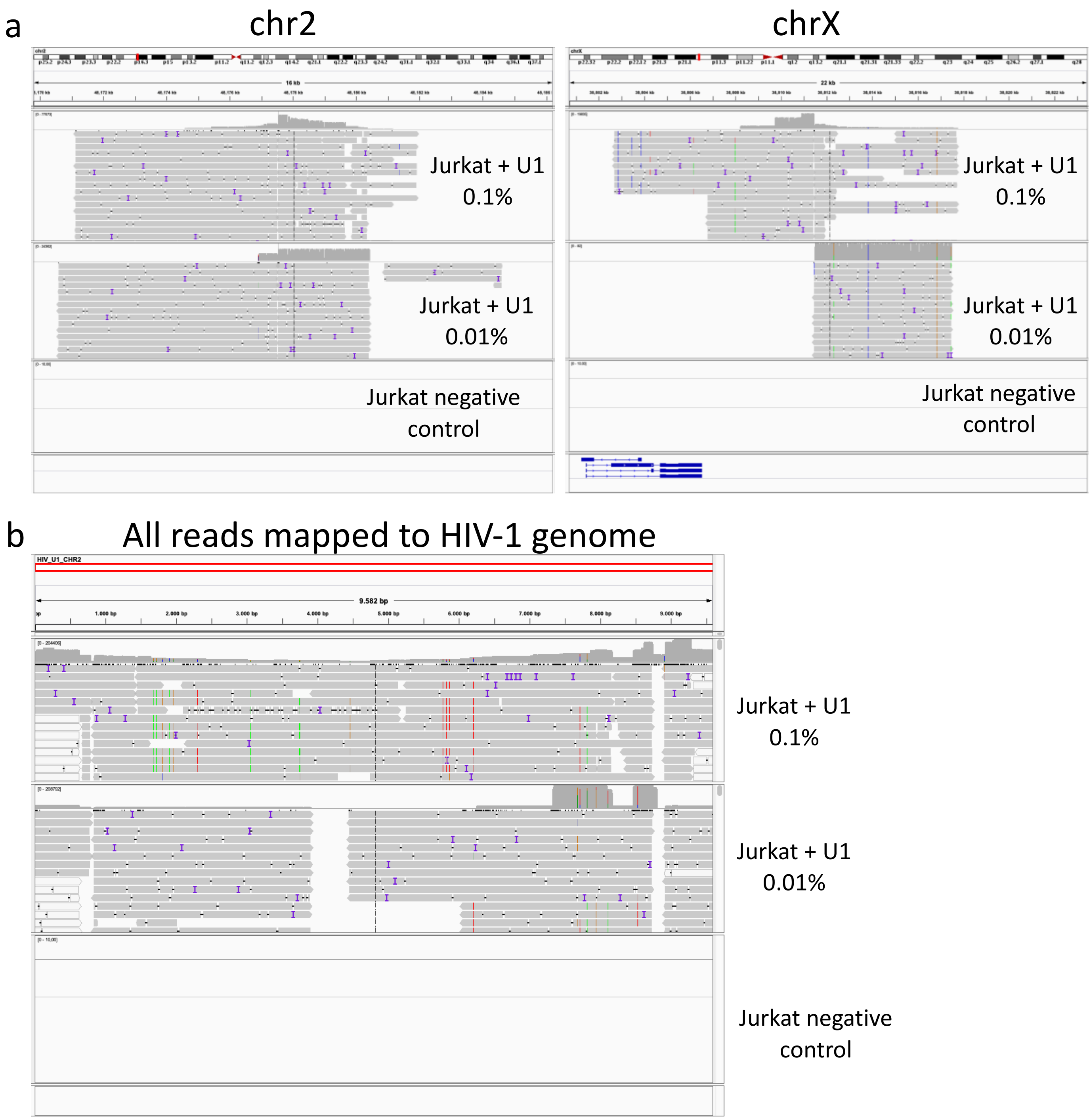

Fig. S7 Three PCIP-seq libraries were prepared in parallel using 5 µg of template DNA, all used the same guides and primers. Following sequencing and demultiplexing the Jurkat negative control produced 12,137 reads, Jurkat + U1 0.01% produced 234,421 reads and Jurkat + U1 0.1% 252,913 reads. **(a)** The resultant reads were mapped to the human genome, the major integration sites observed in U1 on chr2 and chrX are shown. **(b)** The reads were also mapped the HIV-1 genome. No reads of pure HIV-1 or chimeric HIV-1/host reads were observed in the Jurkat negative control. In Jurkat + U1 0.01% samples 12.6% of the reads were chimeric HIV-1/host, in Jurkat + U1 0.1% this rose to 43.2%. Red box at top indicates level of zoom on provirus.

This experiment gives us a rough estimation of the efficiency of PCIP-seq. In the dilution experiment, we started with 5ug of DNA. Assuming a diploid human genome size of 6.51 picograms this should equate to the DNA of approximately ~768,000 cells. At 0.01% the DNA from approximately ~77 U1 cells is present. This corresponds to ~154 proviruses as U1 contains two proviruses per cell (these numbers are probably inflated as cell lines often display extensive aneuploidy). Following circularization, the CRISPR cut and reaction clean-up, we are left with approximately ~12% of the DNA we started with, dropping the number of U1 genomes represented in the DNA to ~9, or ~18 proviruses. As can be seen from the resultant library we are able to identify

both the proviruses in U1 and in the case of the provirus on chr2 we observe amplification from 4 molecules based on observing different shear sites. This means we captured 5 proviruses, this equates to an efficiency of 3.2%.

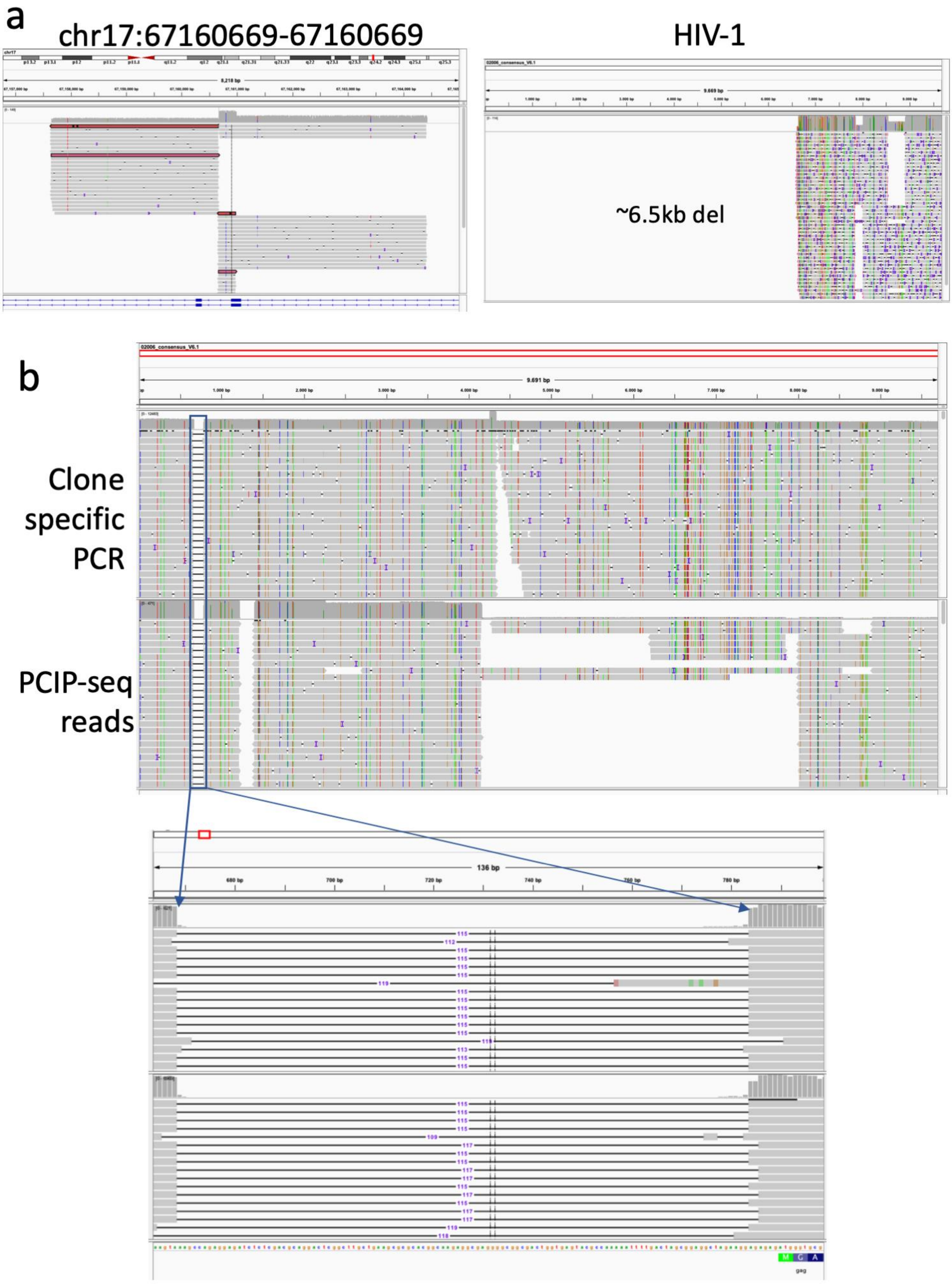
a
chr17:67160669-67160669
HIV-1
~6.5kb del
b
Clone specific PCR
PCIP-seq reads
136 bp
gag

C

Provirus with 5’ deletion
chr10:119577391-119577407

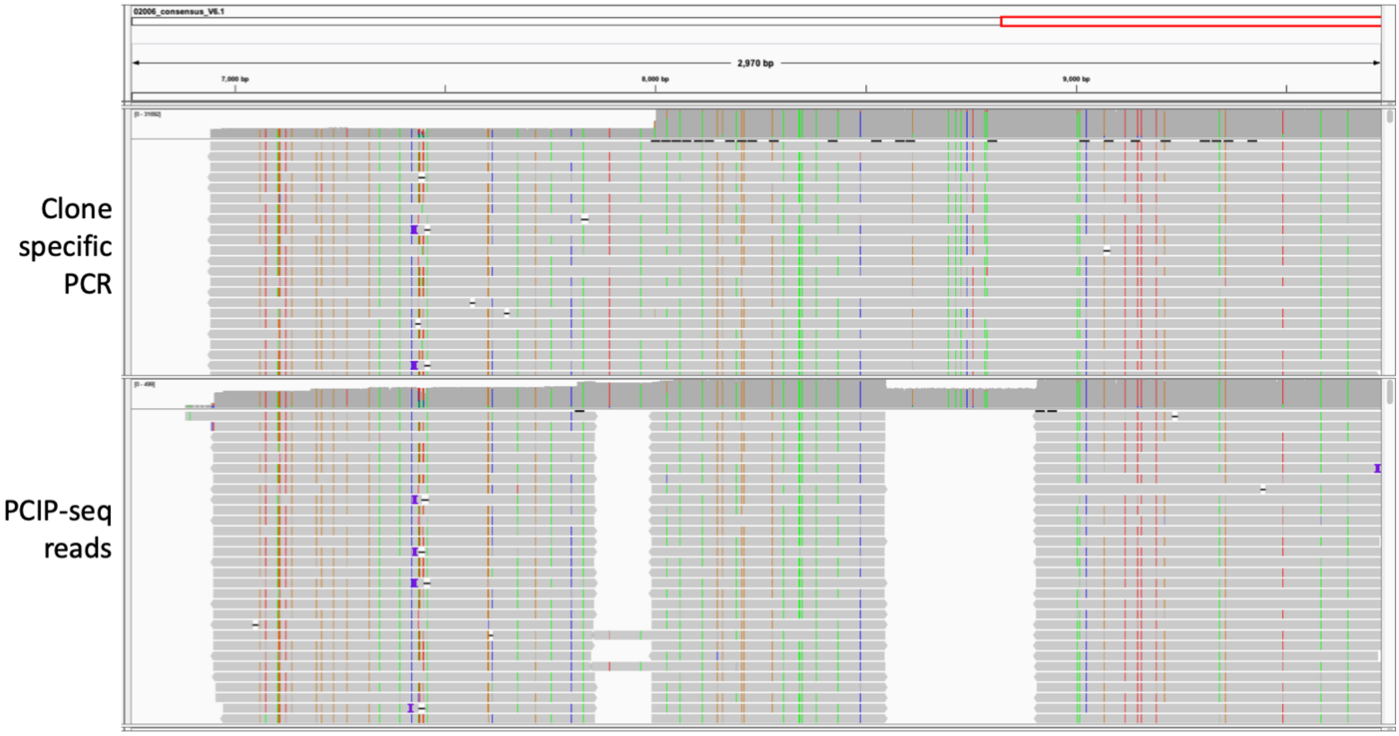

Provirus with 3’ deletion
chr11:128226471-128226471

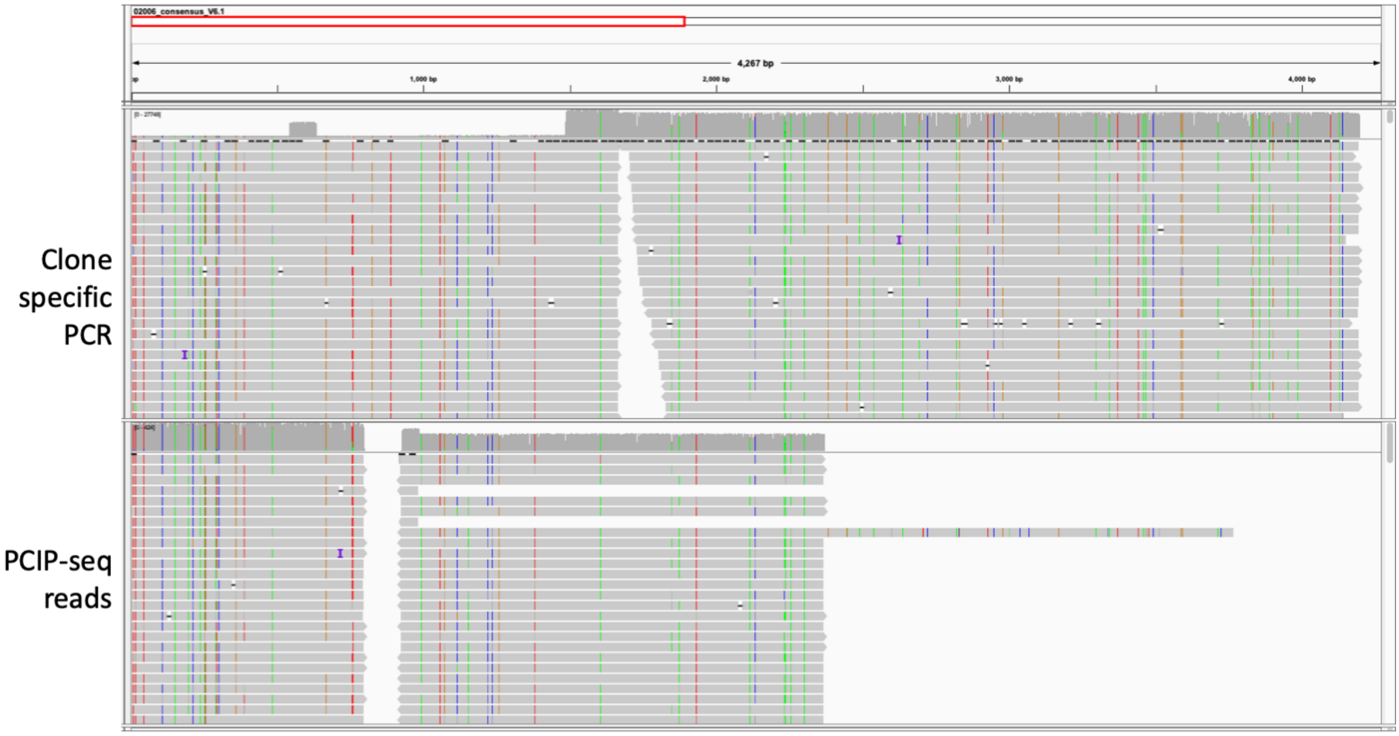

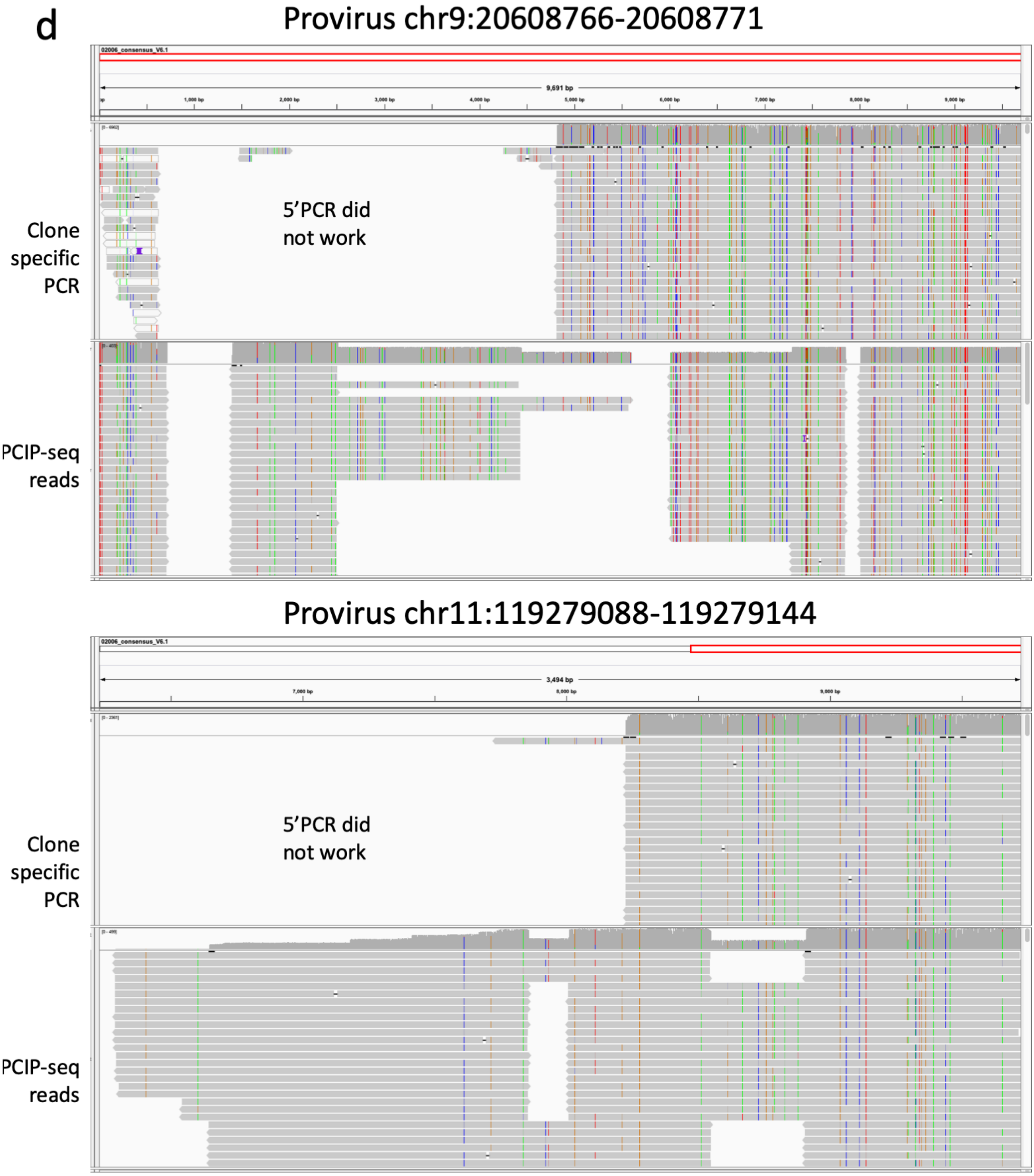

Fig. S8 Examples of HIV-1 proviruses from patient 02006 **(a)** Screen shot from IGV shows a proviral integration site in the host genome as well as the associated provirus sequence. Two reads have been highlighted (out of many) that are found both upstream and downstream of the provirus integration site. With a full-length provirus this would not be possible, however, with a provirus carrying a large deletion including the 5' or 3' regions targeted by the guides a single read can encompass the truncated provirus as well as host DNA from both upstream and downstream of the integration site. The provirus associated with this integration site has a 5' deletion that removes ~6.5kb. **(b)** Reads from the provirus inserted in chr16:29362672-29362679 mapped to the 02006 HIV-1 consensus sequence. Red box at top indicates level of zoom on provirus. This provirus has a ~115 bp deletion affecting the region containing the packaging signal (Ψ). This provirus was also

amplified via clone specific PCR. In addition to the ~115 bp deletion the SNPs in the clone specific PCR mirror those observed from the PCIP-seq library. The elevated coverage towards the middle is where the two PCR products overlap. **(c)**. Large deletions affecting both the 5' and 3' end of the provirus were frequently observed. Shown are reads from both PCIP-seq and clone specific nested PCR mapped to the 02006 HIV-1 consensus sequence. The pattern of SNPs in the clone specific PCR mirrors those observed from the PCIP-seq library **(d)** Partial conformation of two proviruses. In both cases the nested PCR for the 5' end did not work. The provirus at chr9:20608766-20608771 appears full length, the provirus at chr11:119279088-119279144 appears to have a 5' deletion. Pattern of SNPs in the clone specific PCR again mirrors those observed from the PCIP-seq library.

ERV insertion in the APOB gene

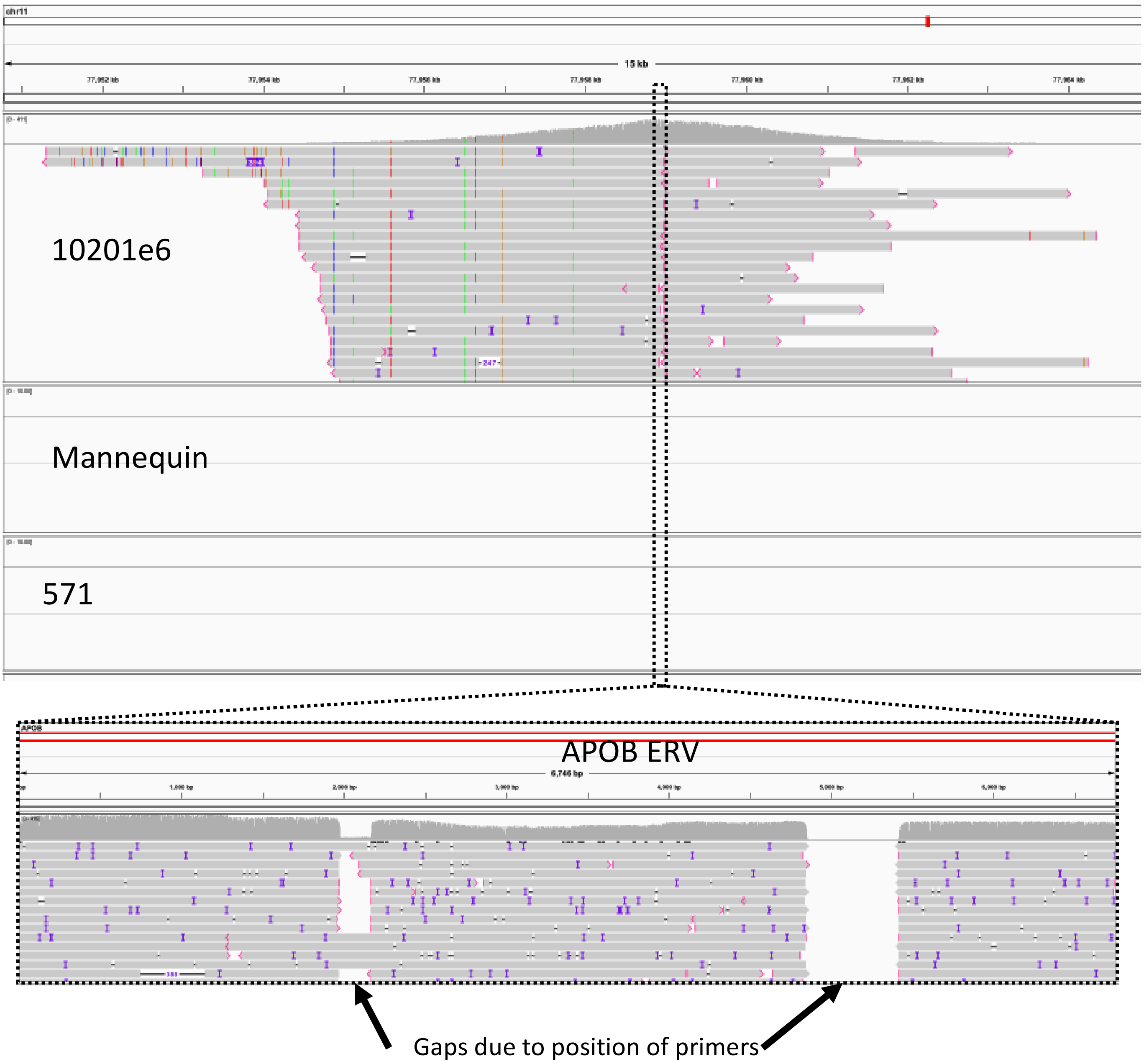

Fig. S9 Screen capture from IGV: PCIP-seq identified the insertion site of the ERV responsible for cholesterol deficiency in Holstein cattle. No reads are seen mapping to this position in libraries from the other two cattle (Mannequin & 571). Below is shown the partial sequence of the provirus.

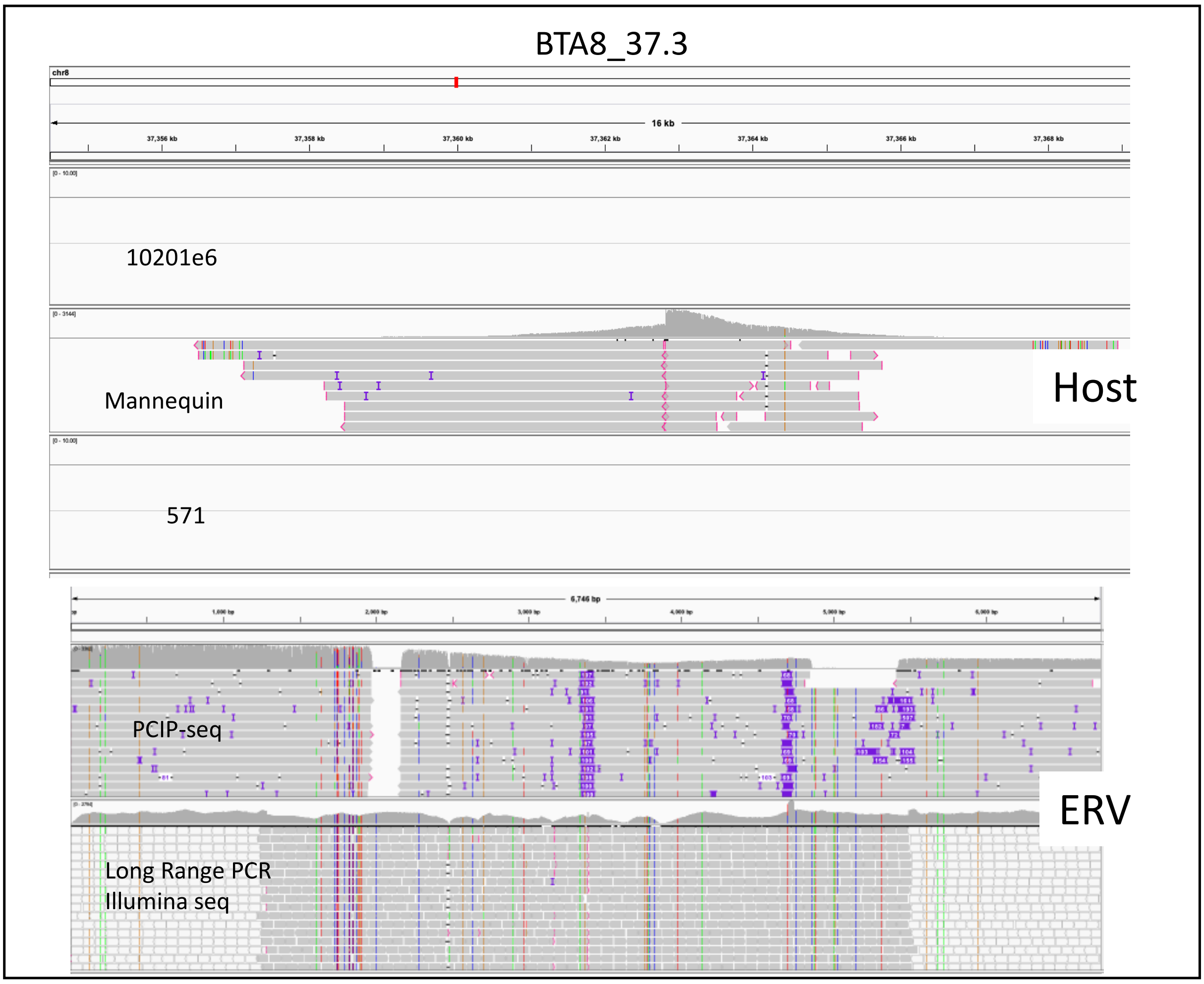

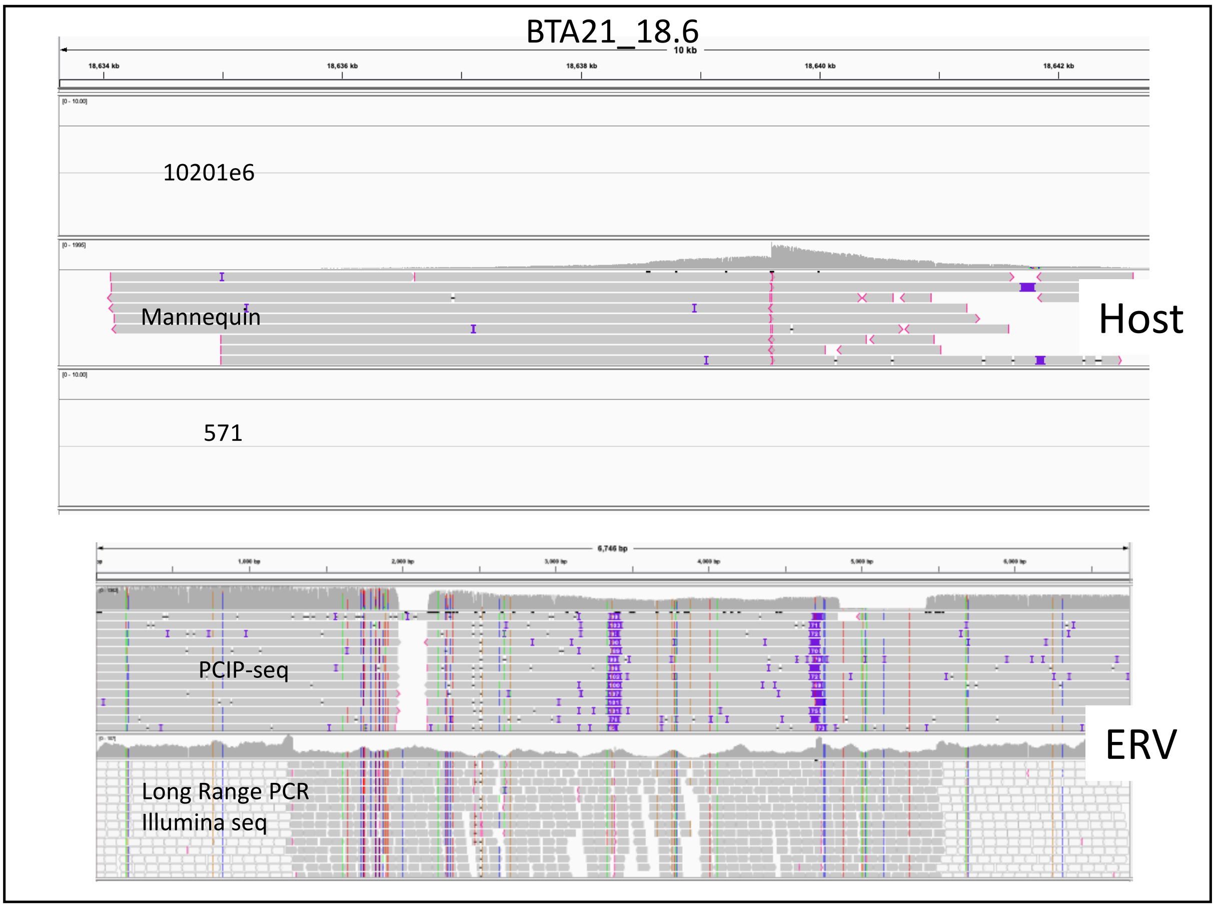

Fig. S10 Validated, Bovine endogenous retrovirus (BERVK2) identified via PCIP-seq.

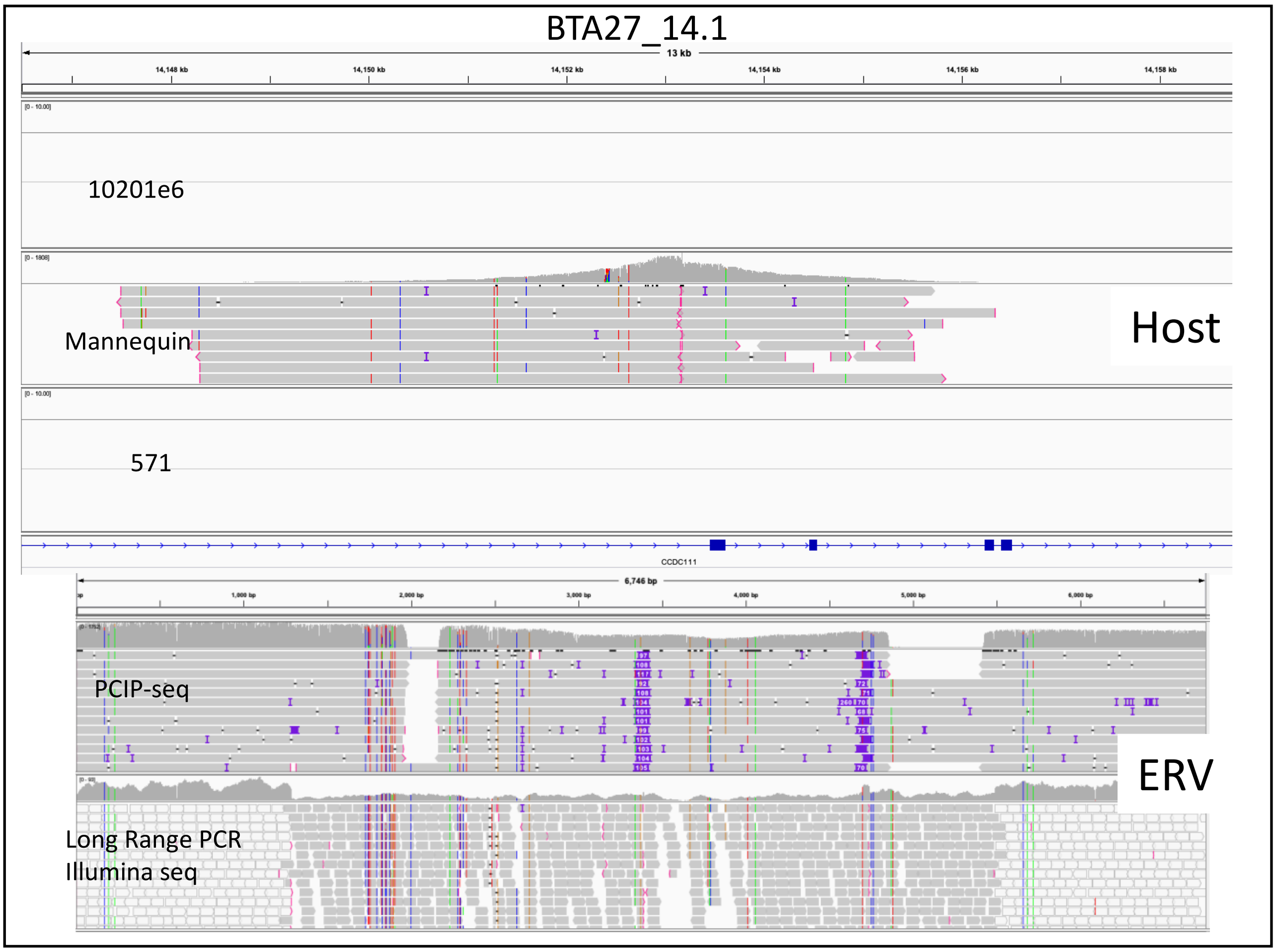

Fig. S10 continued

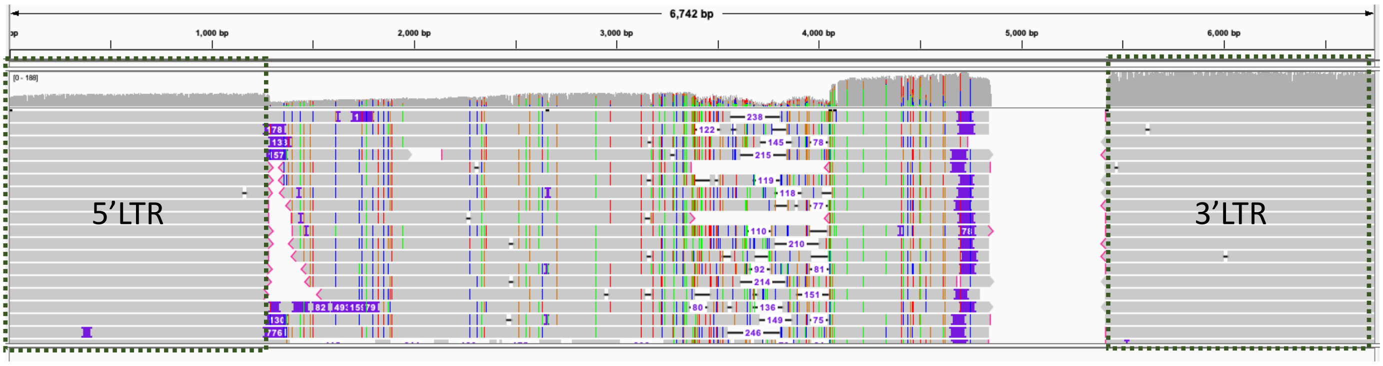

Fig. S11 ERV BTA3_115.3 LTRs match APOB (BTA11_77.9) ERV.

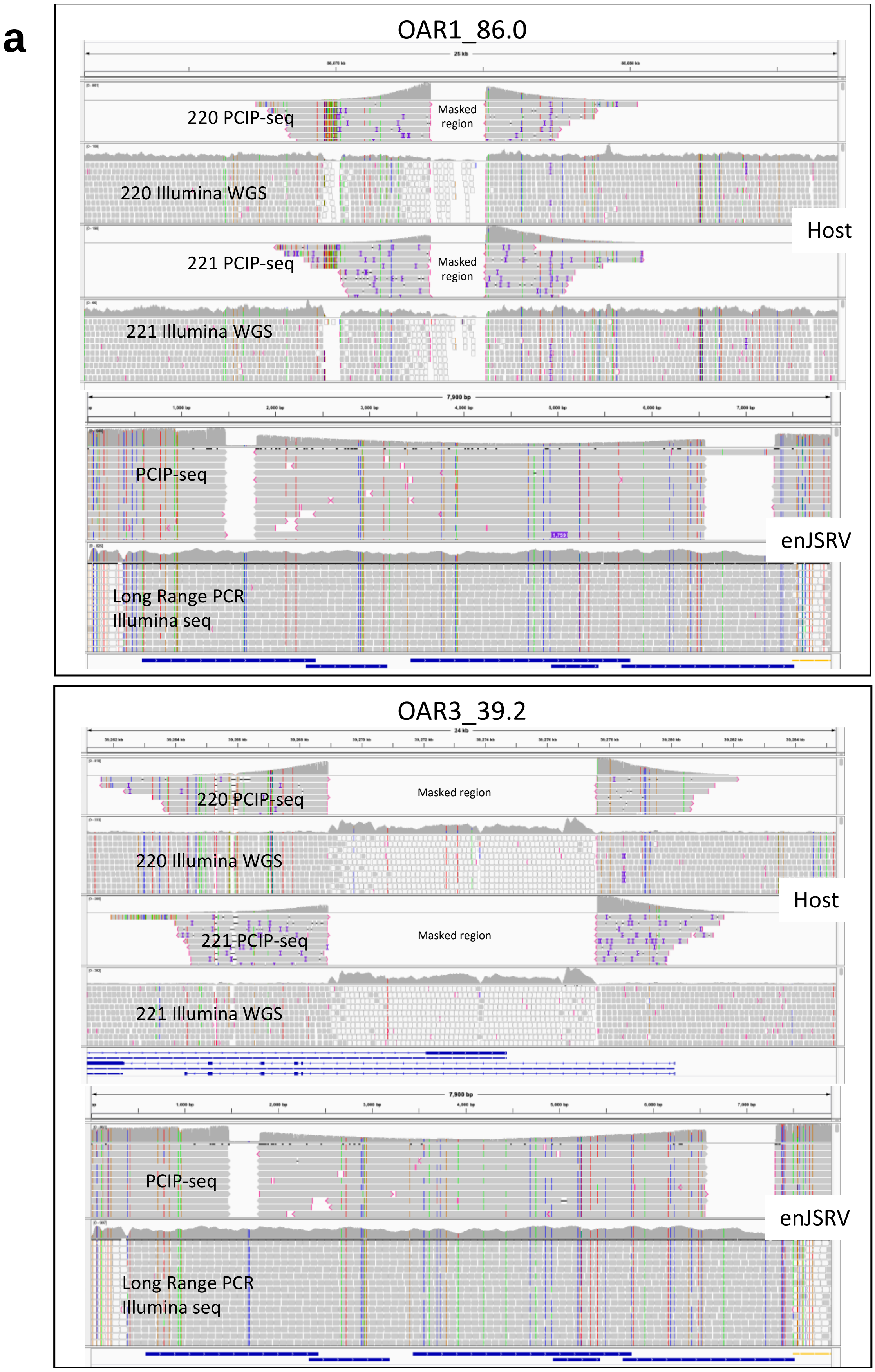

Fig. S12 Validated, enJSRV **(a)** The PCIP-seq reads were mapped to the reference genome (OAR3) where sequences matching enJSRV had been masked out, this preventing reads from multiple proviruses mapping to these positions. Hybrid reads in the unique flanking sequence allowed us to determine the sequence of the proviruses present at these locations (WGS = Whole genome sequencing). **(b)** Evidence of enJSRV insertion was also observed in Illumina WGS data from both animals. Colors, flag reads where one end is mapping to another region in the genome, pointing to an insertion at that position.

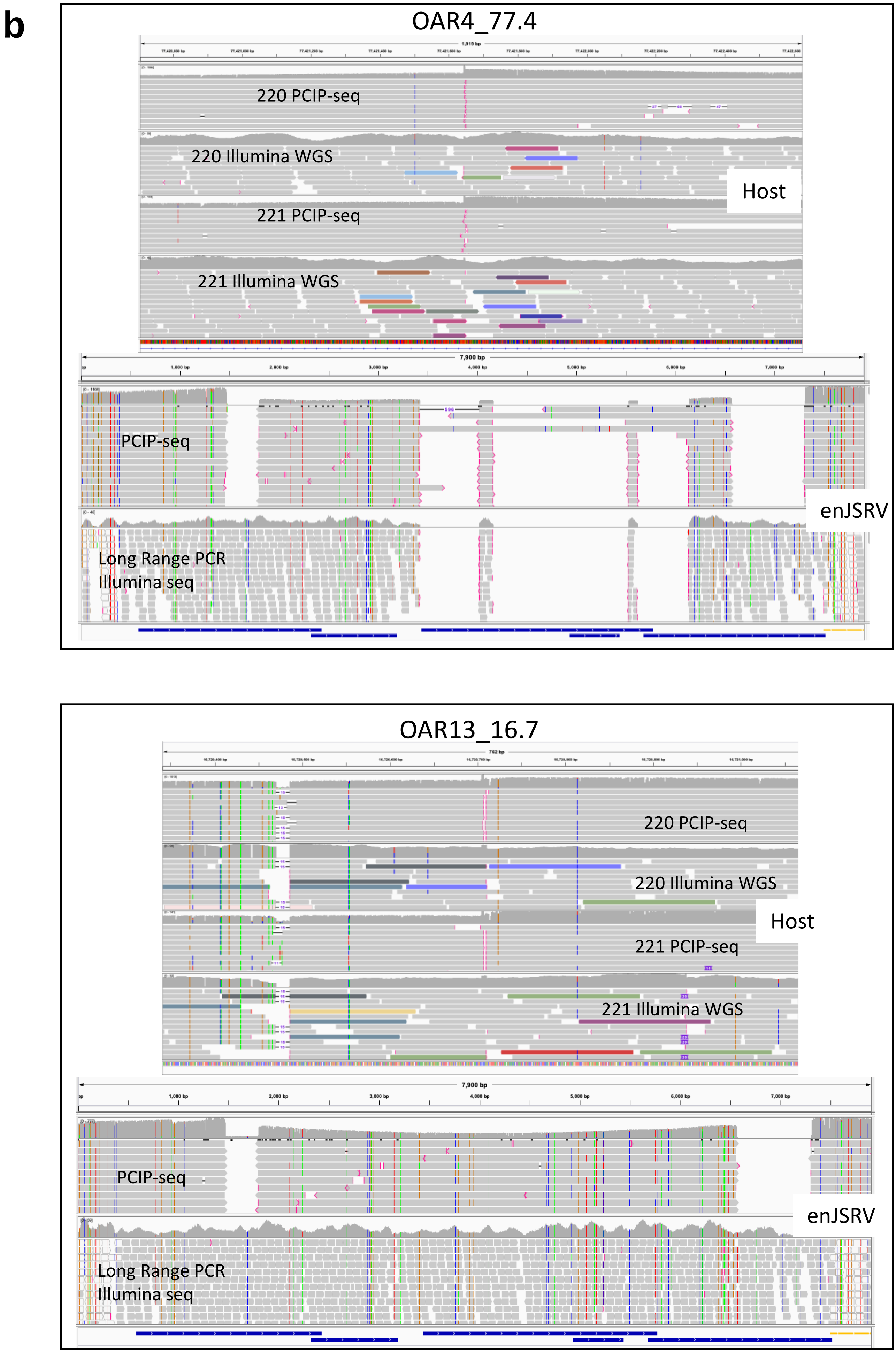

Fig. S12 continued

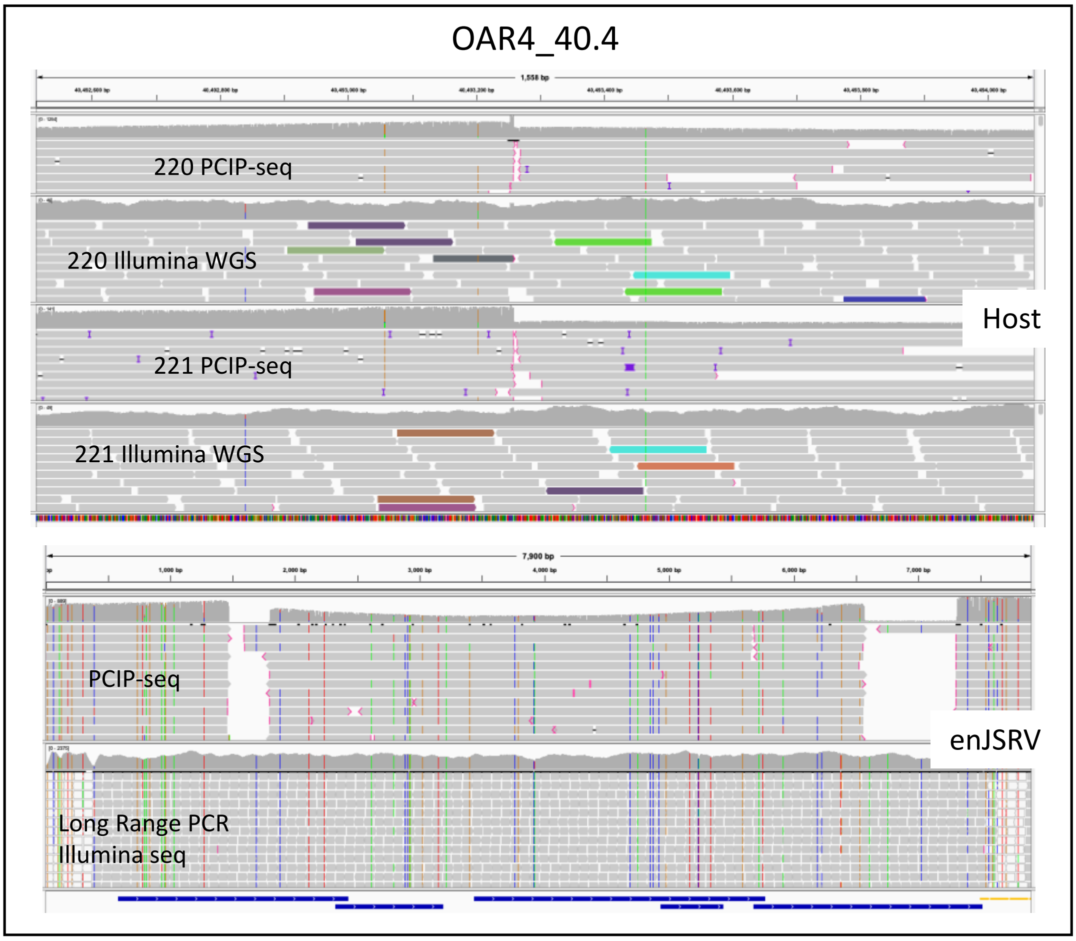

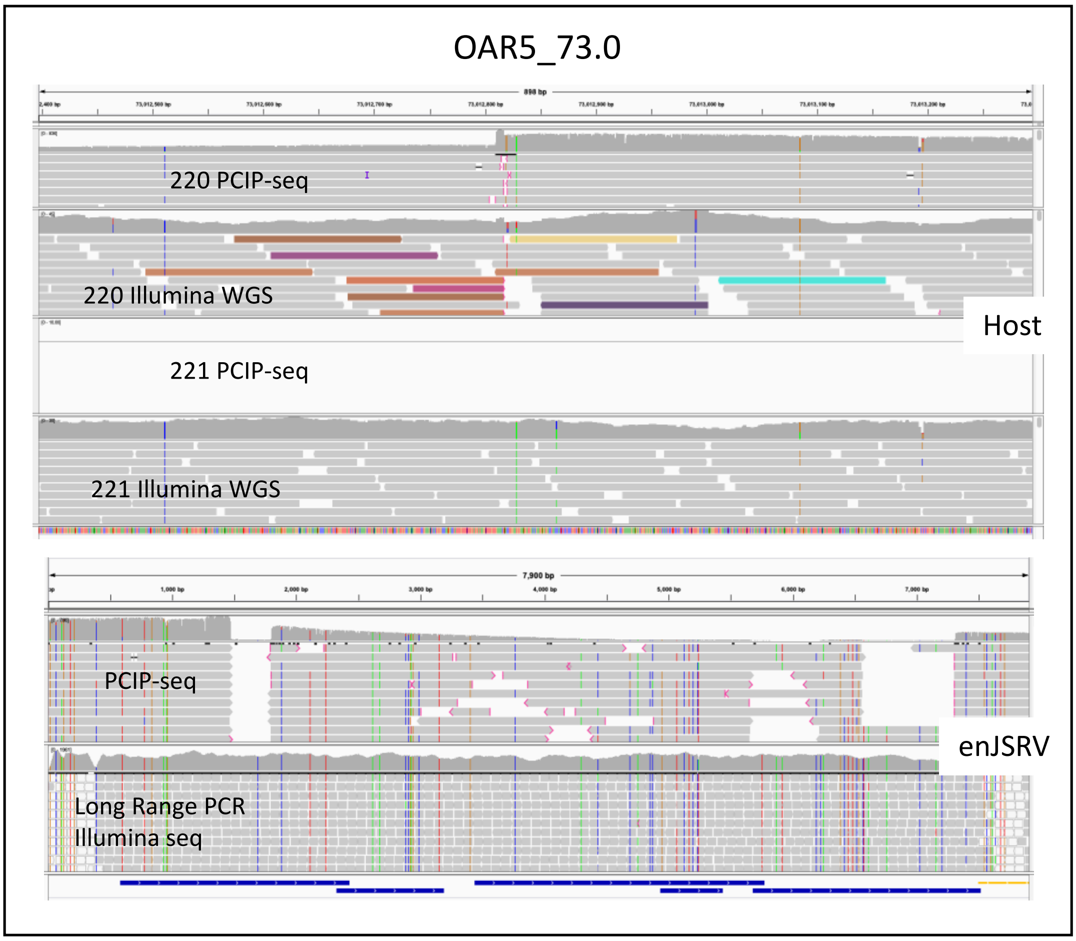

Fig. S12 continued

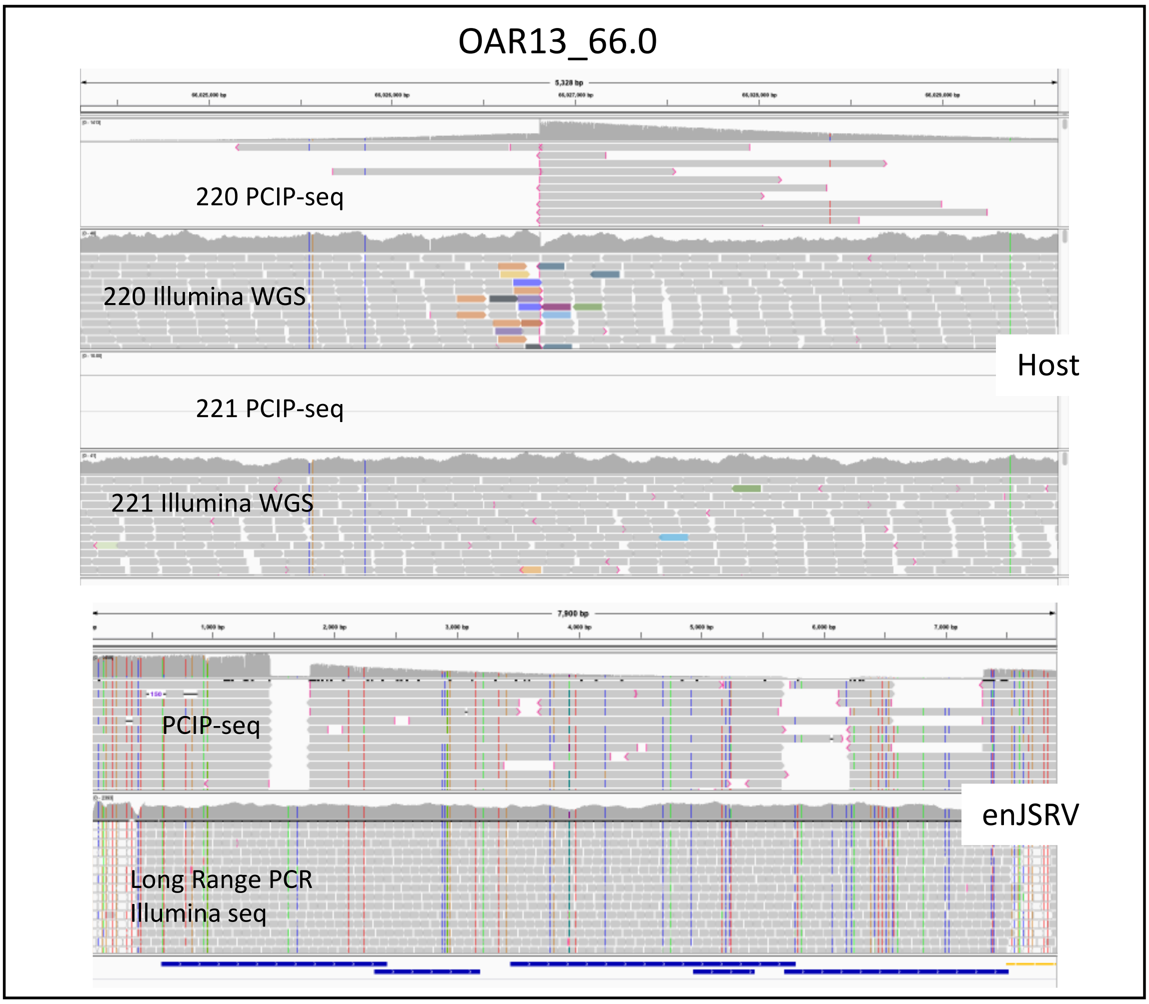

Fig. S12 continued

Table S1

Sample	Insertion sites ILLUMINA	Insertion sites PCIP-seq	U-IS ILL. in PCIP (%)	Pearson Correlation	U-IS PCIP in ILL. (%)	Insertion sites ILLUMINA (>3)	Insertion sites PCIP-seq (>3)	U-IS ILL. in PCIP (%) (>3)	U-IS PCIP in ILL. (%) (>3)	Raw PCIP-seq reads	Raw Illumina reads
233	1110	5311	81.2	0.950	12.1	448	2302	85.9	7.7	524698	173196
221 (022016)	1122	8023	40.4	0.512	4.8	74	3546	50	0.4	180276	9579
221 (032014)	4473	5374	44.4	0.526	40.5	1555	1524	34.9	14.5	32266	391478
220	915	1352	36.1	0.895	27.9	401	664	47.6	16.3	44876	299554
1439	5784	5773	47.7	0.895	44.5	1449	3053	63.9	15.0	181055	216525
560	379	172	15.8	0.617	14.6	81	77	33.3	11.1	6802	192170
1053	8496	17903	62.0	0.811	29.1	2196	7777	68.5	7.3	367454	219461

Comparing PCIP-seq to ligation mediated PCR and Illumina sequencing.
For the Illumina libraries the template DNA used was 4 µg. For the PCIP-seq it varied between libraries (233=7µg, 221(022016)=4µg, 221(032014)=4µg, 220=2µg, 1439=3µg, 560=1µg, 1053=6µg). >3 signifies insertion sites supported by more than 3 reads after PCR duplicate removal. ILLUMINA = Ligation mediated PCR with Illumina sequencing. U-IS ILL. in PCIP = Unique insertion sites (%) identified in ILLUMINA and also found in PCIP-seq. U-IS PCIP in ILL = Unique insertion sites (%) identified in PCIP-seq and also found in ILLUMINA. Pearson's correlation Abundance = correlation of abundances from proviruses detected in both Illumina and PCIP-seq.

Table S2

Sample name	Species	PVL	# Insertion sites	# Proviruses examined for SNPs	# Variants detected (AF > 0.6)	# Proviruses with variant (AF > 0.6)	# Positions within proviruses with variant (AF > 0.6)
233	OAR	78.3	5311	789	233	168	136
221 (022016)	OAR	63.0	8023	408	93	79	86
221 (032014)	OAR	16.0	5374	70	6	6	6
220	OAR	3.8	1352	130	50	42	36
1439	BosT	45.0	5773	587	311	211	137
1053	BosT	23.5	17903	1243	241	182	169

Numbers of SNPs identified in each sample.

Table S3

1053

Provirus	Type	Region in BLV	Approx size	Clone specific PCR
1_120275095_120275095	DEL	230-252	22	no
1_147862114_147862122	DEL	2241-2275	34	no
2_106933456_106933462	DEL	7674-7708	34	no
3_6970332_6970339	DEL	5109-6728	1619	no
3_90671155_90671163	DEL	2608-2919	311	no
4_114867583_114867589	DEL	4574-4637	63	no
5_25818093_25818100	DEL	4482-4526	44	no
6_95273607_95273614	DEL	4487-5537	1050	no
6_112133285_112133291	DEL	5217-5368	151	no
10_101509344_101509352	DEL	7324-7425	101	no
12_36183673_36183673	DEL	1808-1835	27	no
13_35328779_35328785	DEL	3679-4603	924	no
15_24605050_24605054	DEL	8136-8162	26	no
16_28380797_28380803	DEL	2984-3895	911	no
17_64277037_64277043	DEL	5418-5636	218	no
20_7882911_7882911	DEL	8111-8137	26	no
20_7882911_7882911	DEL	8230-8340	110	no
21_53434814_53434824	DEL	6854-7130	276	no
21_53434814_53434824	DEL	7202-7246	44	no
22_40343810_40343823	DEL	4629-4838	209	no
22_48239823_48239830	DEL	2271-2799	528	no
23_41760533_41760533	DEL	8100-8201	101	no
24_22643966_22643974	DEL	6857-7165	308	no
25_33749737_33749744	DEL	4225-4264	39	no
28_28470239_28470248	DEL	4496-5191	695	no
29_25146501_25146508	DEL	3901-5251	1350	no
X_33071616_33071616	DEL	3322-3969	647	no
X_61600607_61600612	DEL	6193-6783	590	no

1439

Provirus	Type	Region in BLV	Approx size	Clone specific PCR
10_65013091_65013093	DEL	2164-3192	1028	no
1_150385145_150385351	DEL	3451-3474	23	yes
2_121703720_121703726	DEL	5350-5399	49	no
23_39892380_39892560	DEL	2364-2560	196	yes
2_4188067_4188067	DEL	2176-2570	394	no
24_3748146_3748155	DEL	5419-5497	78	no
27_36582809_36582809	DEL	4522-5636	1114	yes
27_36582809_36582809	DEL	1-852	852	yes
4_100234239_100234246	INS	8296-8370	75	yes
5_51456241_51456285	DEL	1-4152	4152	yes
2_124084208_124084213	DEL	391-406	15	yes
3_45576532_45576538	DEL	2316-2336	20	yes
5_95348339_95348346	DEL	8167-8200	33	no
8_112613917_112613964	DEL	4225-6244	2019	no
5_6307451_6307451	INS	3251-3590	338	no

221 (022016 & 032014)

Provirus	Type	Region in BLV	Approx size	Clone specific PCR
OAR3_128671913_128671921	DEL	4591-4620	30	no
OAR18_26694984_26694991	DEL	5287-5508	222	no
OAR25_25097056_25097063	DEL	2325-4303	1979	yes
OARX_110727773_110727797	DEL	2858-2970	113	no
OARX_78143793_78143801	DEL	3284-6602	3298	yes

221 (032014)

Provirus	Type	Region in BLV	Approx size	Clone specific PCR
OAR14_25755878_25755884	DEL	5846-6486	640	no

221 (022016)

Provirus	Type	Region in BLV	Approx size	Clone specific PCR
OAR1_25125478_25125485	DEL	6237-6255	19	no
OAR1_250672128_250672136	DEL	7365-7389	25	yes
OAR2_73878244_73878251	DEL	237-264	28	no
OAR3_149619110_149619110	DEL	7610-7726	117	no
OAR3_211678275_211678275	DEL	6228-6285	58	no
OAR8_80161637_80161982	DEL	6502-6561	60	yes
OAR13_10090846_10090865	DEL	6484-6561	78	no
OAR16_10037623_10037623	DEL	1287-1396	110	no
OAR21_31148897_31148902	DEL	7292-7544	253	no
OAR24_28280610_28280610	DEL	6807-6828	22	no
OAR2_242159705_242159712	INS	7017-7232	215	yes

233

Provirus	Type	Region in BLV	Approx size	Clone specific PCR
OAR10_34545991_34546003	DEL	5298-5330	32	yes
OAR10_49266255_49266262	DEL	6512-6586	74	yes
OAR14_42146250_42146256	DEL	1658-1724	66	no
OAR16_3998022_3998027	DEL	4479-4706	227	no
OAR19_37466567_37466573	DEL	278-428	150	no
OAR23_14140808_14140814	DEL	3270-5878	2608	no
OAR3_184106381_184106391	DEL	5799-5874	75	no
OAR7_72584331_72584331	DEL	4574-5453	879	no
OAR7_72649090_72649098	DEL	539-629	90	no

BLV structural variants identified via PCIP-seq.

Table S4

	02006	06042
**Clinical characteristics**		
Age (years)	52	47
Gender	Male	Male
Time of diagnosis (year)	2002	2006
Viral load zenith (log10 HIV-1 c/ml)	5.45	5.65
CD4 count, nadir (cells/mm^3)	44	118
Start of ART (year)	2002	2009
Total ART duration (years)	15	8
Time of sampling (year)	2017	2017
CD4 count at sampling (cells/mm^3)	293	659
Viral load at sampling (HIV-1 c/ml)	<50	<50
CD4/CD8 ratio at sampling	0.33	0.56
**Virological markers**		
Total HIV-1 DNA (c/10^6 CD4 T-cells)	4644	5648

Clinical information for the HIV-1 patients

Table S5

#	Approximate location in genome (BTA6)	Provirus name	10201e6	Mannequin	571	Provirus
1	chr1:108,822,892-108,832,262	BTA1_108.8	no	no	YES	Full
2	chr1:140,473,236-140,486,732	BTA1_140.4	YES	no	YES	Full
3	chr2:7,341,443-7,349,776	BTA2_7.3	no	no	YES	Full
4	chr2:68,574,688-68,583,604	BTA2_68.5	YES	no	no	Partial
5	chr2:108,763,340-108,771,071	BTA2_108.7	no	YES	no	Full
6	chr2:136,856,893-136,860,100	BTA2_136.8	YES	no	no	Full
7	chr3:11,025,879-11,032,187	BTA3_11.0	no	YES	no	Full
8	chr3:21,243,379-21,247,173	BTA3_21.24	no	YES	no	Full
9	chr3:21,262,507-21,266,148	BTA3_21.26	no	YES	no	Full
10	chr3:115,305,677-115,313,191	BTA3_115.3	YES	no	no	**Full***
11	chr4:23,529,679-23,538,398	BTA4_23.5	YES	no	no	Partial
12	chr4:106,804,424-106,812,368	BTA4_106.8	no	no	YES	Full
13	chr5:76,505,040-76,518,833	BTA5_76.5	YES	YES	YES	Full
14	chr6:19,795,982-19,804,772	BTA6_19.7	YES	YES	YES	Full
15	chr6:33,664,998-33,674,349	BTA6_33.6	YES	no	no	Full
16	chr6:93,979,584-93,984,028	BTA6_93.9	YES	YES	YES	Partial
17	chr7:18,507,208-18,514,234	BTA7_18.5	no	YES	no	Partial
18	chr7:62,318,935-62,329,558	BTA7_62.3	YES	no	no	Full
19	chr7:109,501,965-109,512,061	BTA7_109.5	YES	no	YES	Full
20	chr8:16,410,224-16,424,259	BTA8_16.4	YES	no	YES	Full
21	chr8:37,357,029-37,369,016	BTA8_37.3	no	YES	no	Full
22	chr8:67,963,331-67,972,754	BTA8_67.9	no	YES	no	Full
23	chr8:81,237,785-81,244,766	BTA8_81.2	YES	YES	no	Full
24	chr9:15,412,806-15,418,477	BTA9_15.4	YES	no	no	Partial
25	chr9:83,082,008-83,092,749	BTA9_83.0	YES	no	no	Full
26	chr9:84,257,434-84,262,548	BTA9_84.2	YES	no	no	Full
27	chr9:101,949,614-101,957,434	BTA9_101.9	YES	YES	no	Full
28	chr10:71,920,524-71,928,975	BTA10_71.9	YES	no	no	Full
29	chr10:87,425,735-87,443,841	BTA10_87.4	YES	YES	YES	Partial
30	chr11:50,592,847-50,606,524	BTA11_50.5	YES	no	YES	Full
31	chr11:61,788,705-61,792,024	BTA11_61.7	no	YES	no	Full
32	chr11:77,955,413-77,963,724	BTA11_77.9	YES	no	no	**Full#**
33	chr12:72,978,039-72,985,406	BTA12_72.9	YES	YES	no	Full
34	chr12:74,723,248-74,731,915	BTA12_74.7	YES	YES	no	Partial
35	chr15:9,435,764-9,439,369	BTA15_9.4	YES	YES	YES	Full
36	chr16:10,720,162-10,727,571	BTA16_10.7	YES	no	no	Full
37	chr16:13,308,596-13,315,659	BTA16_13.3	YES	no	no	Partial
38	chr16:28,504,653-28,536,456	BTA16_28.5	YES	no	YES	Full
39	chr18:27,619,893-27,626,348	BTA18_27.6	YES	no	YES	Partial
40	chr18:27,715,161-27,722,285	BTA18_27.7	no	no	YES	Full
41	chr18:50,368,602-50,378,304	BTA18_50.3	YES	YES	YES	Full
42	chr18:60,211,168-60,220,590	BTA18_60.2	YES	YES	YES	Partial
43	chr18:61,691,367-61,697,347	BTA18_61.6	YES	no	YES	Full
44	chr19:5,180,841-5,189,334	BTA19_5.1	YES	no	no	Partial
45	chr19:22,014,748-22,025,138	BTA19_22.0	YES	no	no	Full
46	chr19:51,039,969-51,101,363	BTA19_51.0	no	YES	YES	Partial
47	chr20:15,283,426-15,290,599	BTA20_15.2	YES	no	no	Full
48	chr20:55,126,259-55,134,120	BTA20_55.1	no	YES	no	Full
49	chr21:1,241,740-1,256,399	BTA21_1.2	YES	YES	YES	Partial
50	chr21:2,303,211-2,307,834	BTA21_2.3	no	YES	no	Full
51	chr21:4,133,180-4,142,631	BTA21_4.1	no	no	no	Full
52	chr21:18,634,068-18,645,042	BTA21_18.6	no	YES	no	Full
53	chr22:160,456-166,792	BTA22_160.4	no	no	YES	Full
54	chr23:41,312,657-41,328,100	BTA23_41.3	YES	no	no	Full
55	chr23:52,329,640-52,337,577	BTA23_52.3	YES	no	no	Full
56	chr24:12,819,683-12,824,449	BTA24_12.6	YES	YES	no	Partial
57	chr24:53,067,680-53,078,844	BTA24_53.0	no	no	YES	Full
58	chr25:20,428,960-20,444,963	BTA25_20.4	no	no	no	Full
59	chr26:50,606,858-50,616,960	BTA26_50.6	YES	no	no	Full
60	chr27:14,146,146-14,156,627	BTA27_14.1	no	YES	no	Full
61	chr28:17,575,320-17,582,731	BTA28_17.5	YES	no	no	Full
62	chr29:39,631,808-39,639,476	BTA29_39.6	YES	no	no	Full
63	chrX:27,723,875-27,732,458	BTAX_27.7	no	YES	no	Full
64	chrX:30,183,463-30,187,122	BTAX_30.1	YES	no	no	Partial
65	chrX:36,260,818-36,264,888	BTAX_36.2	YES	no	no	Partial
66	chrX:43,949,278-43,960,449	BTAX_43.9	no	no	YES	Full
67	chrX:47,314,044-47,327,526	BTAX_47.3	no	no	YES	Full

Endogenous retroviruses (BERVK2) identified in cattle via PCIP-seq.
*****LTR matches APOB ERV (BTA11_77.9)
#ERV inserted into APOB
Full = Full length ERV.
Partial = ERV with large deletion.

Table S6

	Approximate location in genome (OAR3)	ERV name	**220**	**221**	provirus
1	chr1:57,132,178-57,139,903	OAR1_57.13	no	YES	Full
2	chr1:86,065,652-86,091,348	OAR1_86.0	YES	YES	Full
3	chr1:129,489,883-129,502,056	OAR1_129.4	no	YES	Full
4	chr1:220,250,002-220,258,800	OAR1_220.2	YES	YES	Full
5	chr1:240,077,458-240,092,905	OAR1_240.0	YES	YES	Partial
6	chr1:253,739,233-253,756,582	OAR1_253.7	YES	YES	Partial
7	chr2:196,585,537-196,593,010	OAR2_196.5	YES	no	Full
8	chr3:39,261,134-39,285,428	OAR3_39.2	YES	YES	Full
9	chr3:39653898-39656987	OAR3_39.6	YES	YES	Partial
10	chr3:151,767,643-151,783,037	OAR3_151.7	YES	YES	Partial
11	chr3:182,538,937-182,555,692	OAR3_182.5	YES	no	Full
12	chr4:40,485,410-40,504,790	OAR4_40.4	YES	YES	Full
13	chr4:77,416,611-77,428,510	OAR4_77.4	YES	YES	Partial
14	chr5:7,744,521-7,756,178	OAR5_7.74	YES	YES	Partial
15	chr5:64,916,815-64,926,920	OAR5_64.9	YES	no	Partial
16	chr5:73,009,027-73,018,771	OAR5_73.0	YES	no	Full
17	chr6:5,400,881-5,410,594	OAR6_5.4	no	YES	Full
18	chr6:6,789,991-6,858,767	OAR6_6.7	YES	YES	Partial
19	chr6:26,968,086-26,977,558	OAR6_26.9	no	YES	Full
20	chr8:2,974,531-2,988,179	OAR8_2.9	YES	YES	Partial
21	chr8:49,483,598-49,499,241	OAR8_49.4	YES	YES	Partial
22	chr9:48,096,442-48,105,912	OAR9_48.0	no	YES	Full
23	chr9:89,743,769-89,752,495	OAR9_89.7	no	YES	Partial
24	chr10:70,892,072-70,919,960	OAR10_70.8	YES	no	Partial
25	chr11:32,085,050-32,095,786	OAR11_32.0	YES	YES	Full
26	chr13:5,676,353-5,686,765	OAR13_5.6	no	YES	Full
27	chr13:16,714,529-16,726,069	OAR13_16.7	YES	YES	Full
28	chr13:37,514,438-37,529,955	OAR13_37.5	YES	YES	Full
29	chr13:66022872-66031772	OAR13_66.0	YES	no	Full
30	chr14:13,811,039-13,844,103	OAR14_13.8	YES	YES	Partial
31	chr14:15,011,370-15,043,076	OAR14_15.0	YES	YES	Partial
32	chr14:56,232,971-56,236,157	OAR14_56.2	YES	YES	Full
33	chr14:57,491,683-57,503,056	OAR14_57.4	no	YES	Partial
34	chr14:57,605,121-57,623,737	OAR14_57.6	YES	YES	Partial
35	chr15:10,864,017-10,870,430	OAR15_10.8	no	YES	Full
36	chr17:48,876,178-48,887,208	OAR17_48.8	no	YES	Full
37	chr18:1,738,143-1,751,356	OAR18_1.7	no	YES	Partial
38	chr18:67,778,281-67,799,930	OAR18_67.7	YES	YES	Full
39	chr19:52,665,989-52,689,785	OAR19_52.6	YES	YES	Partial
40	chr20:433,819-443,901	OAR20_0.4	YES	no	Full
41	chr20:1,237,366-1,250,699	OAR20_1.2	no	YES	Partial
42	chr20:27,598,593-27,615,677	OAR20_27.5	no	YES	Full
43	chr21:6,694,384-6,709,701	OAR21_6.6	YES	no	Partial
44	chr22:46,781,990-46,790,196	OAR22_46.7	no	YES	Full
45	chr26:8,253,764-8,265,010	OAR26_8.2	no	YES	Full
46	chrX:3,690,949-3,701,009	OARX_3.6	YES	no	Full
47	chrX:62,939,566-62,949,333	OARX_62.9	YES	YES	Partial
48	chrX:78,127,416-78,132,398	OARX_78.1	YES	no	Partial

Endogenous retroviruses (enJSRV) identified in sheep via PCIP-seq.

Full = Full length ERV.
Partial = ERV with large deletion.

Table S7

Patient	ID	Estimated read count	Overlapping Gene	geneID	Notes
HPV18_PX	chr1:201993711-201993711	1	RNPEP	ENSG00000176393	
HPV18_PX	chr1:54070808-54070808	1	TCEANC2	ENSG00000116205	
HPV18_PX	chr1:74339164-74339164	2	FPGT-TNNI3K	ENSG00000259030	
HPV18_PX	chr11:72988358-72988358	6	FCHSD2	ENSG00000137478	
HPV18_PX	chr12:124528897-124528897	5	NCOR2	ENSG00000196498	
HPV18_PX	chr12:62430096-62430096	3	NA	NA	
HPV18_PX	chr12:88750111-88750111	2	NA	NA	
HPV18_PX	chr13:32401471-32401471	1	N4BP2L1	ENSG00000139597	
HPV18_PX	chr13:59883976-59883976	1	DIAPH3	ENSG00000139734	
HPV18_PX	chr13:70017637-70017637	1	KLHL1	ENSG00000150361	
HPV18_PX	chr13:96145444-96145444	1	HS6ST3	ENSG00000185352	
HPV18_PX	chr16:35696743-35696743	4	NA	NA	
HPV18_PX	chr16:46391666-46391666	15	NA	NA	
HPV18_PX	chr16:60839237-60839237	3	NA	NA	
HPV18_PX	chr17:50736162-50736162	1	LUC7L3	ENSG00000108848	
HPV18_PX	chr17:71945217-71945217	1	NA	NA	
HPV18_PX	chr18:33256597-33256597	2	CCDC178	ENSG00000166960	
HPV18_PX	chr2:175176252-175176252	1	NA	NA	
HPV18_PX	chr2:184979785-184979785	1	NA	NA	
HPV18_PX	chr2:222973976-222973976	1	NA	NA	
HPV18_PX	chr20:26724089-27697774	1	NA	NA	Virus in satellite repeat
HPV18_PX	chr20:59882951-59882951	4	SYCP2	ENSG00000196074	
HPV18_PX	chr21:31443081-31443081	5	TIAM1	ENSG00000156299	
HPV18_PX	chr21:8210410-8210516	6	FP671120.3	ENSG00000280800	
HPV18_PX	chr21:8225927-8228889	9	FP671120.1	ENSG00000278996	
HPV18_PX	chr21:8393406-8393551	9	FP236383.2	ENSG00000280614	
HPV18_PX	chr21:8437761-8437761	9	FP236383.3	ENSG00000281181	
HPV18_PX	chr21:8453856-8454775	19	NA	NA	
HPV18_PX	chr3:141177260-141177260	1	NA	NA	
HPV18_PX	chr3:183646815-183646815	5	KLHL24	ENSG00000114796	
HPV18_PX	chr3:52477576-52477615	67	NISCH	ENSG00000010322	
HPV18_PX	chr3:52491989-52492028	67	NISCH	ENSG00000010322	
HPV18_PX	chr3:52564151-52564190	75	SMIM4	ENSG00000168273	
HPV18_PX	chr4:113196089-113196089	3	ANK2	ENSG00000145362	
HPV18_PX	chr4:118149173-118149173	2	NDST3	ENSG00000164100	
HPV18_PX	chr4:125160196-125160196	2	NA	NA	
HPV18_PX	chr4:8361851-8361851	1	NA	NA	
HPV18_PX	chr5:85159333-85159333	2	NA	NA	
HPV18_PX	chr6:12217019-12217019	1	NA	NA	
HPV18_PX	chr6:58604926-59721758	1	NA	NA	Virus in satellite repeat
HPV18_PX	chr6:60995120-60995120	4	NA	NA	

HPV18_PX	chr6:72218404-72218404	3	RIMS1	ENSG00000079841	
HPV18_PX	chr6:7655460-7655460	6	NA	NA	
HPV18_PX	chr7:55353950-55353950	10	NA	NA	
HPV18_PX	chr7:63798384-63798384	3	NA	NA	
HPV18_PX	chr7:7812181-7812181	4	AC007161.3	ENSG00000283549	
HPV18_PX	chr7:98111088-98111088	1	LMTK2	ENSG00000164715	
HPV18_PX	chr8:119801685-119801685	13	TAF2	ENSG00000064313	
HPV18_PX	chr8:2564068-2564068	1	NA	NA	
HPV18_PX	chr8:93515097-93515097	1	LINC00535	ENSG00000246662	
HPV18_PX	chr8:9886409-9886409	2	NA	NA	
HPV18_PX	chr9:12503146-12503146	1	NA	NA	
HPV18_PX	chr9:128458663-128458663	1	ODF2	ENSG00000136811	
HPV18_PX	chrX:19414286-19414286	1	MAP3K15	ENSG00000180815	
HPV18_PX	chrX:41675298-41675299	1	CASK	ENSG00000147044	

HPV18_PY	chr5:37774016-37774016	2	NA	NA	
HPV18_PY	chr7:64329003-64329003	2	ZNF736	ENSG00000234444	
HPV18_PY	chr4:184039889-184039889	2	NA	NA	
HPV18_PY	chr18:108534-108534	2	NA	NA	
HPV18_PY	chr3:59699600-59699600	1	NA	NA	
HPV18_PY	chr4:90546531-90546531	1	CCSER1	ENSG00000184305	
HPV18_PY	chr5:146985347-146985347	1	PPP2R2B	ENSG00000156475	
HPV18_PY	chr6:41200232-41200232	1	TREML2	ENSG00000112195	
HPV18_PY	chr6:113561576-113561576	1	NA	NA	
HPV18_PY	chr1:107169512-107169512	1	NTNG1	ENSG00000162631	
HPV18_PY	chr1:218361256-218361256	1	TGFB2	ENSG00000092969	
HPV18_PY	chr3:52563123-52563123	1	SMIM4	ENSG00000168273	
HPV18_PY	chr9:15686595-15686595	1	CCDC171	ENSG00000164989	
HPV18_PY	chr9:137787856-137787856	1	AL590627.1	ENSG00000255585	
HPV18_PY	chr10:6703026-6703026	1	AL158210.2	ENSG00000285743	
HPV18_PY	chr10:23788794-23788794	1	KIAA1217	ENSG00000120549	
HPV18_PY	chr10:91570894-91570894	1	NA	NA	
HPV18_PY	chr11:97096506-97096506	1	NA	NA	
HPV18_PY	chr19:35339090-35339090	1	CD22	ENSG00000012124	

HPV integration sites identified in patients HPV18_PX and HPV18_PY

Estimated read count refers to number of reads after PCR duplicates have been removed, see https://github.com/GIGA-AnimalGenomics-BLV/PCIP/blob/master/README.md

Table S8

PCIP-seq efficiency estimation in BLV

Sample name	Virus	Host	PVL	Template µg	Estimated number proviruses	Raw reads	Chimeric reads (%)	Pure Host / Pure Viral reads	Insertion sites	Largest clone (%)	Efficiency based on unique integration sites*
233	BLV	OAR	78.3	7	913500	524698	53.4	0.04 / 46.53	5311	5.22	0.6
221 (022016)	BLV	OAR	63	4	420000	180276	67.14	3.59 / 29.27	8023	0.625	1.9
221 (032014)	BLV	OAR	16	4	106667	32266	68.69	0.11 / 31.20	5374	0.279	5.0
220	BLV	OAR	3.8	2	12667	44876	67.38	0 / 32.62	1352	3.55	10.7
1439	BLV	BosT	45	3	225000	181055	70.52	0.19 / 29.29	5773	1.17	2.6
560	BLV	BosT	0.644	1	1073	6802	69.83	1.12 / 29.06	172	4.59	16.0
1053	BLV	BosT	23.5	6	235000	367454	72.13	0.04 / 27.83	17903	0.353	7.6

PCIP-seq efficiency estimation in HIV-1

Sample name	Virus	Host	PVL	Templ ate µg	Estimated number proviruses	Raw reads	Chimeric reads (%)	Pure Host / Pure Viral reads	Inserti on sites	Largest clone (%)	Efficiency based on unique integration sites*	Efficiency based on unique shear sites*	Average coverage per integration site	Range of coverage at integration site	Base pairs of host DNA sequenced	Base pairs of provirus sequence d with coverage >20
02006	HIV-1	HSA	0.46	12	9200	240641	51.63	1.10 / 47.27	158	7.82	1.7	2.95	700	1-11114	467929	391036
06042	HIV-1	HSA	0.56	8	7467	226685	21.18	0.41 / 78.41	73	4.77	1.0	1.0	645	2-4449	155945	166914

These efficiency estimations are based on unique integration sites observed and do not account for clonal expansion. As PCIP-seq will frequently capture the same integration site from multiple copies of the same clone this will underestimate the efficiency of the method. In the case of HIV-1 a manual count of shear sites allowed us to more accurately estimate the efficiency observed in these samples.
Chimeric reads (%): percentage of PCIP-seq reads that contain both host and viral sequences. These reads cover the integration site.

Supplementary Information Text

Supplementary Note 1
Rationale behind the use of CRISPR-cas9 to cleave circular DNA

It is established practice to linearize plasmids (generally via cutting with a restriction enzyme) prior to their use as template in PCR. It is believed that this avoids supercoiling and thereby increases PCR efficiency[1]. Following the same logic, we speculated that linearizing our circularized DNA could also increase PCR efficiency.

As we wanted to cut specific sequences we used the CRISPR-cas9 system. In many cases CRISPR-cas9 cleavage is not 100% effective. As can be seen in the gel below, for G2 a fraction of the target DNA remains uncut. In order to increase efficiency of cutting while also including redundancy against any SNPs in the targeted regions we pooled ~3 guides that targeted a relatively small region (generally a few hundred bases). We found that by pooling guides all the target DNA was successfully cut.

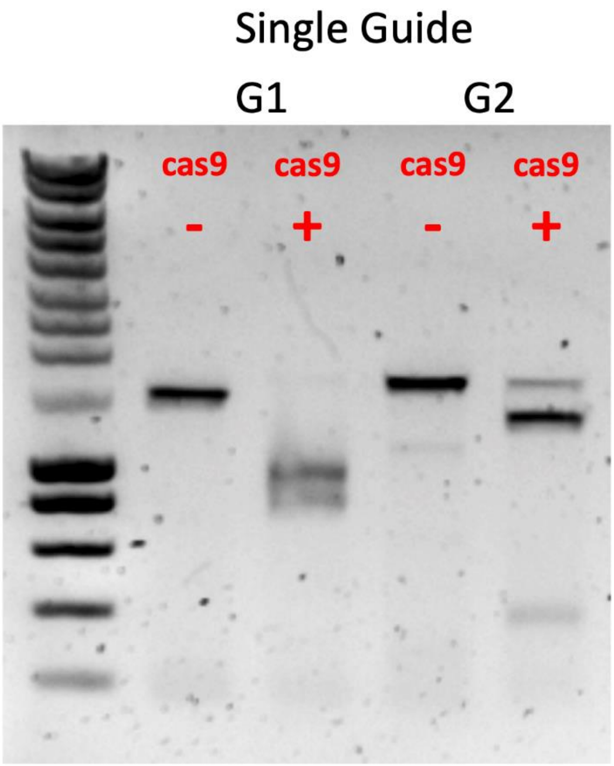

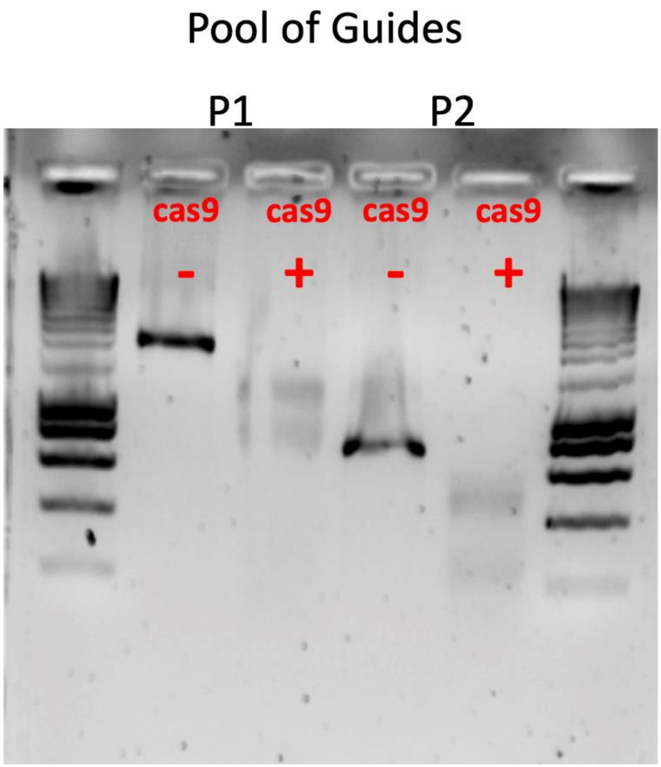

We next applied these pools to circular DNA. The gel below shows an experiment carried out using 8ug of DNA from a BLV infected sheep with a proviral load of 82.6%. The DNA was circularized and linear DNA was eliminated (to prevent PCR amplification/recombination involving the remaining linear fragments) using plasmid safe DNase (see methods for a complete description). One quarter of the resultant DNA was subject to CRISPR-cas9 cleavage using the Pool A guides, the second quarter was cleaved using the Pool B guides, the remaining half was kept aside. The linearized DNA was cleaned and used as template in 2x 50ul PCR reactions using the appropriate primer pairs for Pool A (PA) or Pool B (PB). For the uncut DNA half was used as template for 2x 50ul PCR reactions using the PA primers and the other half was used for 2x 50ul PCR reactions using the PB primers. Following 25 PCR cycles, 10ul of each reaction were loaded on a 1% agarose gel. As can be seen in the gel below, the band intensity for the CRISPR-cas9 cut samples is higher. It should be noted that in lane 3 the PCR smear is shifted down, we generally discard these types of products as the fraction of host-virus fragments is low. (A=unshared genomic DNA, B=genomic DNA sheared to 8kb)

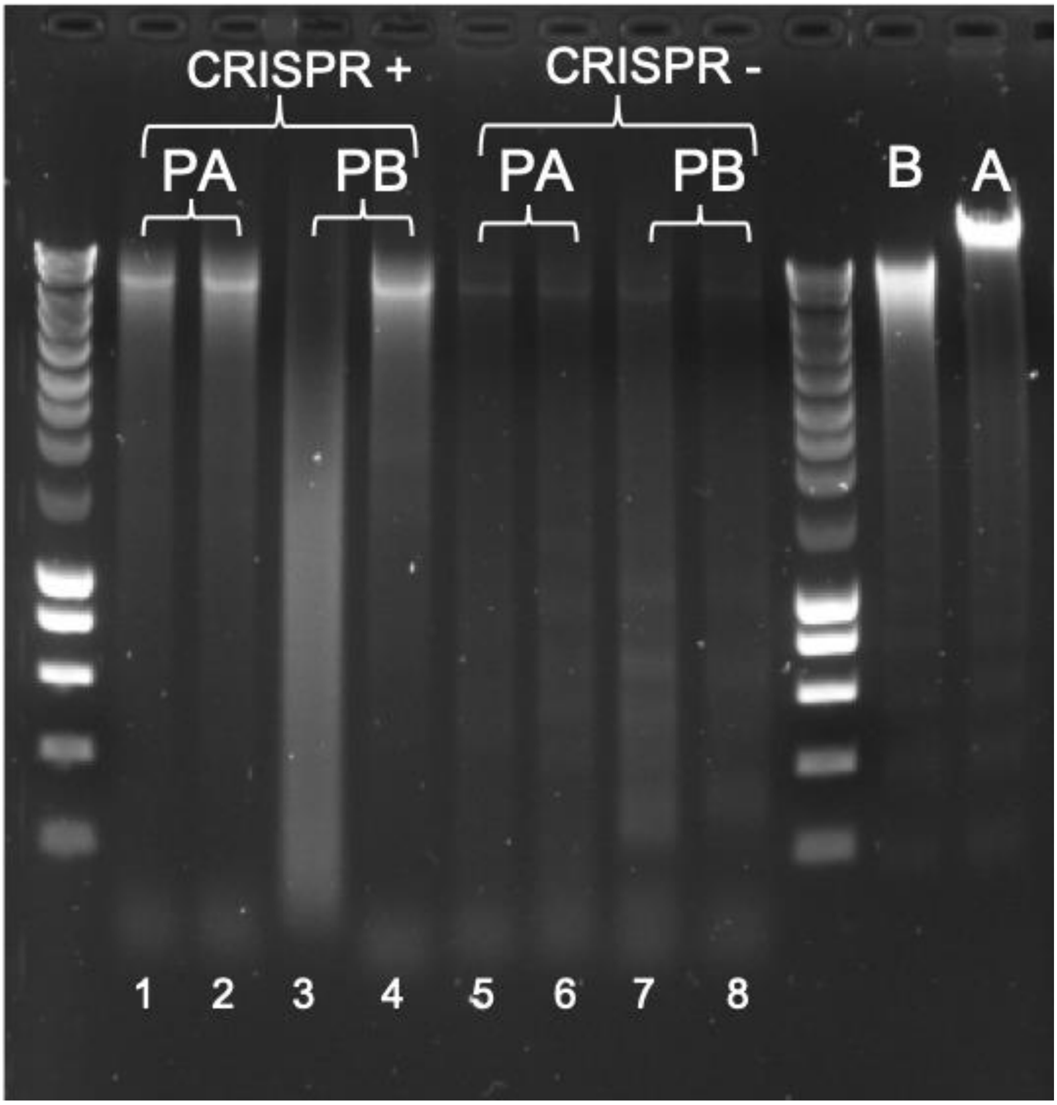

Following clean up and elution in ~40 ul of H2O we took an equal volume (3ul) of each library and indexed them via PCR, in a 50 ul reaction volume and using 8 cycles. Again, following clean up, an equal volume of library was pooled and a nanopore library (LSK-109) was prepared and sequenced on a r9.4 flow cell. Base calling and demultiplexing was carried out as described in the methods. The results are outlined in the table below. In addition the coverage of the resultant reads is shown.

Lib	Treatment	DNA concentration PCR 1 (ng/ul)	DNA concentration PCR 2 (ng/ul)	Raw reads	Chimeric reads %	Pure Host / Pure Viral reads (%)	Mean Length	N50	Median Length	Insertion sites PCIP	Largest clone PCIP (%)	Insertion sites Illumina	Largest clone Illumina (%)
1	PA-Cut BC31	22.52	69.48	113,485	55.6	0.25 / 44.2	2880.6	3855.0	2217.0	2122	25.8	1700	30.849
2	PA-Cut BC32	26.18	72.06	137,109	54.1	0.47 / 45.4	2770.6	3710.0	2141.0	2216	24.7	“	“
3	PB-Cut BC33	71.85	63.7	6,844	1.01	98.5 / 0.51	263.8	277.0	195.5	2	50	“	“
4	PB-Cut BC34	34.17	86.65	126,655	49.4	0.19 / 50.4	2616.2	3395.0	2010.0	2281	24.5	“	“
5	PA-UnCut BC35	13.4	33.32	42,795	22.5	0.19 / 77.3	1759.8	2670.0	1227.0	660	30.9	“	“
6	PA-UnCut BC36	17.26	42.53	66,602	19.7	0.19 /80.2	1549.1	2381.0	1056.0	713	30.4	“	“
7	PB-UnCut BC37	22.27	48.24	114,967	10.4	0.16 / 89.4	917.9	1579.0	497.0	690	29.5	“	“
8	PB-UnCut BC38	14.71	35.92	64,789	18.1	0.19 / 81.7	1461.4	2111.0	992.0	736	30.4	“	“

The table shows that libraries prepared with the CRISPR cut generally produced more raw reads and a much larger fraction of them is composed of the desired chimeric reads containing proviral and host DNA. The CRISPR cut libraries also identified a large number of integration sites. The comparison with an Illumina based library prepared from the same timepoint, using ~4ug of template, shows that PCIP can identify more integration sites. This experiment also shows that only libraries with a size distribution that mirrors that observed in the sheared DNA should be sequenced. Libraries with a preponderance of shorter fragments mainly represent nonspecific amplification.

Coverage of the pure viral reads as well as the chimeric reads on the BLV proviral genome
(BC refers to the barcode used for each library)

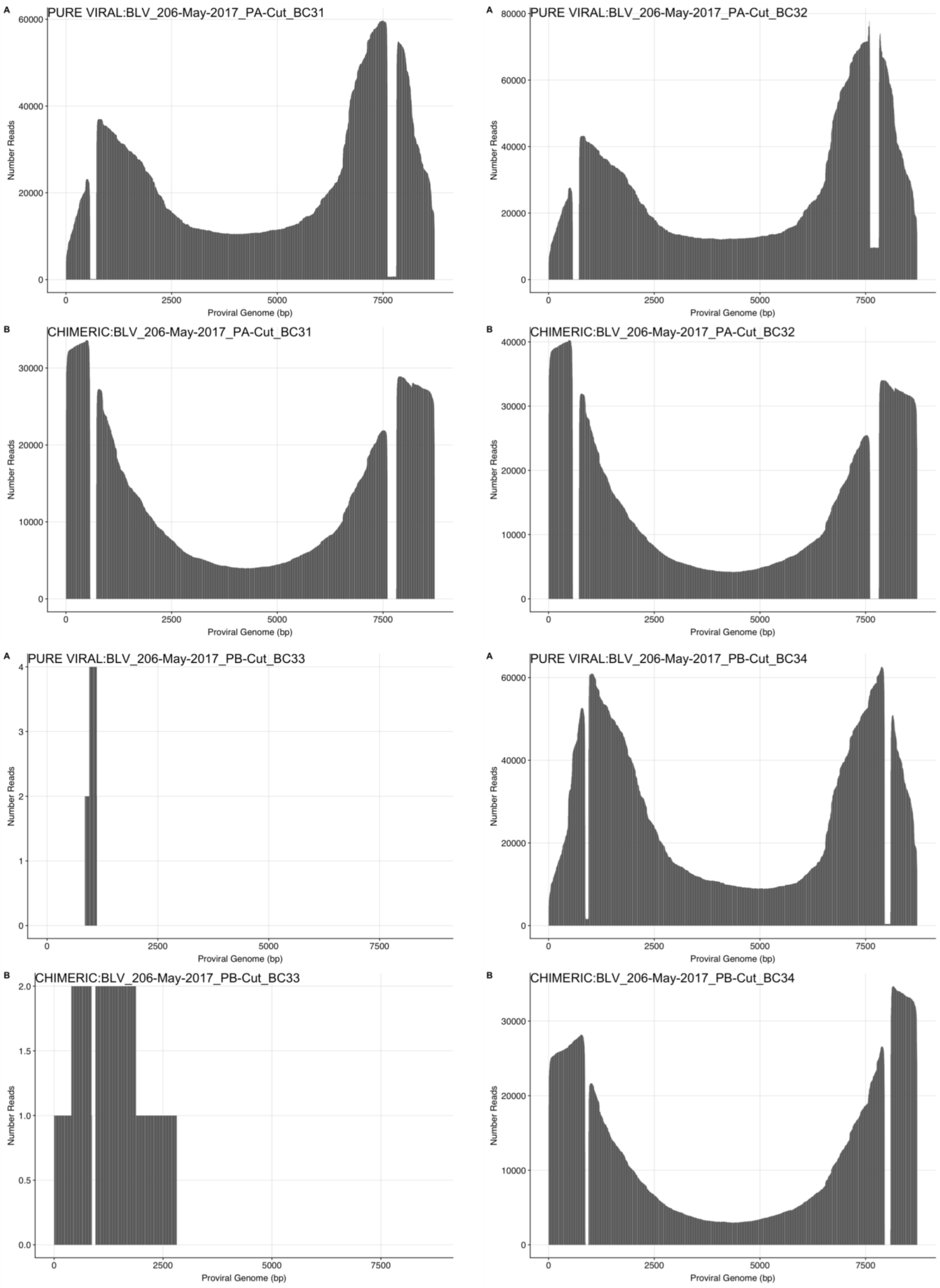

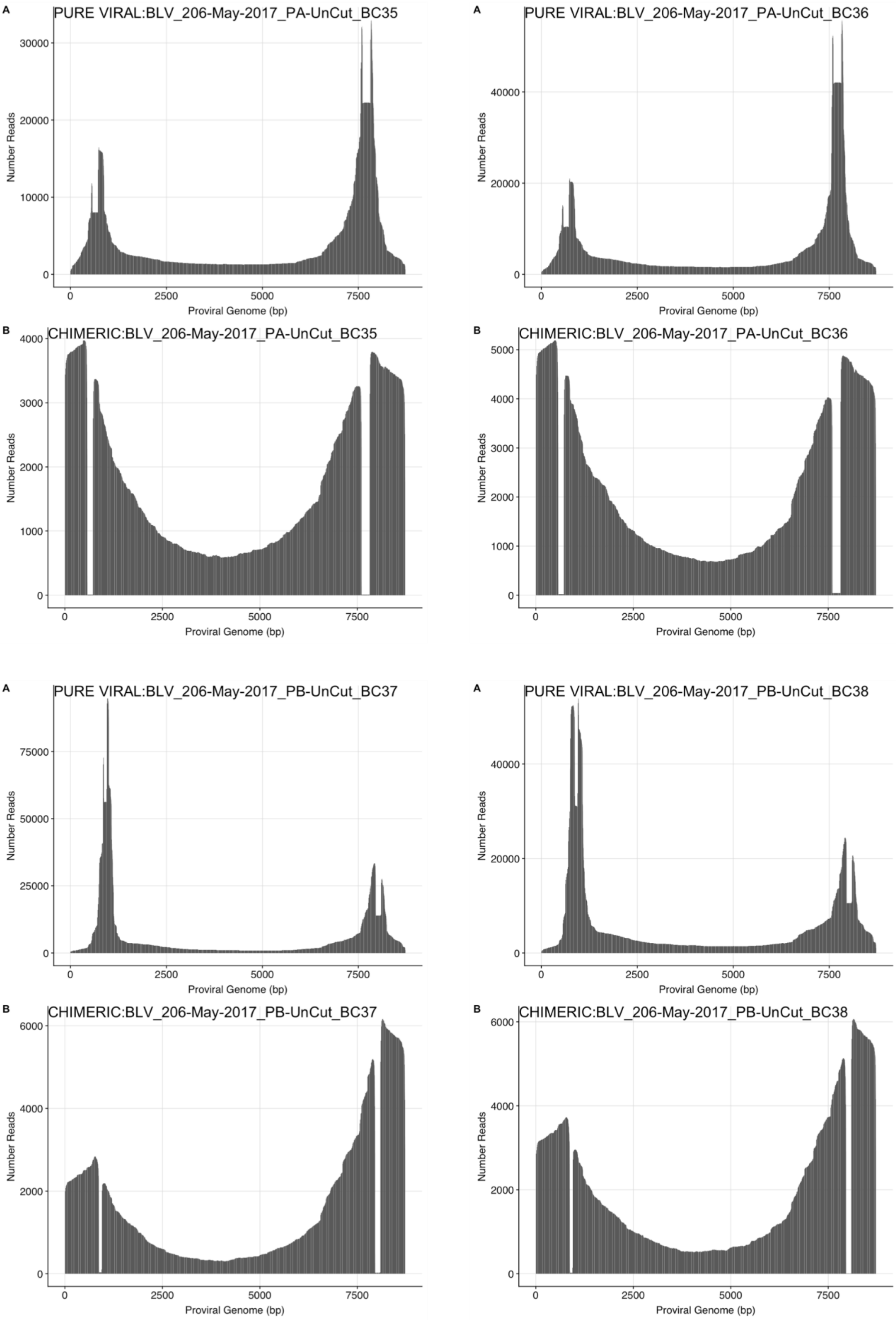
A
PURE VIRAL:BLV_206-May-2017_PA-UnCut_BC35
A
PURE VIRAL:BLV_206-May-2017_PA-UnCut_BC36
B
CHIMERIC:BLV_206-May-2017_PA-UnCut_BC35
B
CHIMERIC:BLV_206-May-2017_PA-UnCut_BC36
A
PURE VIRAL:BLV_206-May-2017_PB-UnCut_BC37
A
PURE VIRAL:BLV_206-May-2017_PB-UnCut_BC38
B
CHIMERIC:BLV_206-May-2017_PB-UnCut_BC37
B
CHIMERIC:BLV_206-May-2017_PB-UnCut_BC38
Number Reads
Proviral Genome (bp)
0
2500
5000
7500

1.

Supplementary Note 2
Effect of coverage on SNP calling

One of the issues often raised regarding Nanopore sequencing is the error rate and the effect sequencing depth has on SNP calls. In this manuscript we have chosen to be quite conservative by only calling SNPs in proviruses with more than 10 reads after PCR duplicate removal. As base calling and variant calling algorithms continue to improve, such a cutoff is likely to be overly conservative. Without carrying out excessive and expensive validation via clone specific PCR it is difficult to decide on an optimal coverage cutoff for calling SNPs in a provirus due to the difficulty of distinguishing between false positives/negatives in the provirus as one adjust thresholds.

In order to get an estimation of the effect coverage has on variant calling we instead decided to look at the part of the host genome captured by PCIP-seq. We sequenced the two HIV-1 patient PCIP-seq libraries used to generate the Nanopore data with Illumina short reads on a MiSeq instrument. This produced ~2.3 million paired-end reads for each library. The shearing necessary to generate the Illumina library prevents us linking viral reads to a specific provirus/integration site, precluding a comparison of variants within the provirus using the two technologies and at different depths. Instead we compared variants found in the DNA flanking the integration sites sequenced by both technologies.

We concentrated on proviruses that were not clonally expanded. We did this for two reasons. Firstly, in the two HIV-1 samples used in this study the majority of proviruses fall into this category. Second, in such cases all the reads are PCR duplicates and cover the same part of the host genomes, at an even coverage, making the downsampling more consistent. We selected proviruses/integrations where the Illumina coverage was on average greater to or equal to ~50X to insure accurate variant calling. This left us with 118 sites in total, 77 sites for patient 02006 and 41 for patient 06042 (both represent 59% of the non-clonally expanded sites in the patients). The flanking host DNA associated with these proviruses covers ~202kb, while the median coverage from the Illumina sequencing was 274X.

We first called SNPs in the Illumina data using lowfreq. The resultant VCF was filtered with SnpSift to retain SNPs with an allele frequency in the reads of >0.6, leaving 157 SNPs. We compared these SNPs to a set of common human variants (ftp://ftp.ncbi.nih.gov/snp/organisms/human_9606/VCF/) and found that 141 (90%) represent common SNPs segregating in the human population. Of the remaining 10% (16 SNPs), 13 had a high allele frequency in the reads (average AF= 0.98), suggesting that these are true SNPs at a low frequency in the population. (This assumes that the original Watson and Crick strands both generally contribute to the amplified pool of products and having the same base substitution on both strands in the very early rounds of amplification is rare). For the remaining 3 SNPs, the allele frequency average was ~0.65, suggesting PCR errors that had occurred in the first round of amplification on either the Watson and Crick strand, that had come to dominate the pool of products, lifting it over our 0.6 cutoff.

We next called SNPs in the Nanopore data from the same 118 regions (median coverage 547X). After filtering for an allele frequency in the reads of >0.6 we called 142 SNPs. All overlapped with the SNPs called in the Illumina data. This left 15 SNPs called in the Illumina data but absent from the Nanopore calls (9.6% false negatives). Of these, 10 had been called as SNPs by lowfreq in the Nanopore data but the allele frequency (average AF= 0.49) did not exceed the 0.6 cutoff. (Two of the potential false positives SNPs called in the Illumina data, with AF=~0.65, were not called as SNPs in the Nanopore data, the third false positive was also called in the Nanopore data.) For the remaining 5 SNPs, where lowfreq did not produce calls, examination of the reads in IGV showed them to be imbedded within a homopolymer (a known weakness of the R9.4 Nanopore data), although their presence could be deduced by examining the raw reads in IGV. Rebase calling the data with the latest high accuracy Nanopore base callers or using the R10.3 flow cell would help with these homopolymer regions. Alternative technologies like Pacbio Hi-Fi reads could also be employed if high single molecule accuracy is desired.

To address the effect of coverage on variant calling we then downsampled every region to an average coverage of ~100X, ~50X and ~20X and again called SNPs from the remaining Nanopore reads. Downsampling did not result in any false positives. Instead, reducing read number had the effect of increasing false negatives, with false negative rates of 13.4%, 15.3% and 19.1% observed in the ~100X, ~50X and ~20X downsampled reads respectively. These numbers are summarized in the table below.

	Illumina	Nanopore	Nanopore 100X	Nanopore 50X	Nanopore 20X
Sample 02006	119	107	103	101	98
Sample 06042	38	35	33	32	29
Total	157	142	136	133	127
% SNPs called in Nanopore also called in Illumina		90.4	86.6	84.7	80.9

Calling SNPs in the host DNA flanking HIV-1 proviruses with both Nanopore and Illumina technologies and examining the effect of coverage on number of SNPs called.

As a consequence, we can conclude that higher coverage helps reduce false negatives, but even with relatively modest coverage false positives are not a major issue, instead we are more likely to get false negatives as the coverage goes down.

Supplementary Methods

PCR validation and Illumina sequencing

Clone specific PCR products as well as the PCIP-seq libraries generated from the HIV-1 patients were sheared to ~400bp using the Bioruptor Pico (Diagenode) and Nextera XT indexes added as previously described[2]. Illumina PCIP-seq libraries were generated in the same manner. Sequencing was carried out on either an Illumina MiSeq or NextSeq 500. Clone specific PCR products sequenced on Nanopore were indexed by PCR, multiplexed and libraries prepared using the Ligation Sequencing Kit 1D (SQK-LSK108) and sequenced on a MinION R9.4 flow cell. Oligos used can be found in Supplementary Dataset 3.

Measure of HIV-1 DNA content in CD4 T-cell DNA isolates by digital droplet PCR (ddPCR)

The DNA was subjected to a restriction digest with EcoRI (Promega, Leiden, The Netherlands) for one hour, and diluted 1:2 in nuclease free water. HIV-1 DNA was measured in triplicate using 4 µL of the diluted DNA as input into a 20µL reaction, while the RPP30 reference gene was measured in duplicate using 1 µL as input. Primers and probes are summarized in Supplementary Dataset 3. Thermocycling conditions were as follows: 95˚C for 10 min, followed by 40 cycles of 95˚C for 30 s and 56°C for 60 s, followed by 98°C for 10 min.

Detailed PCIP-seq protocol

Before starting library preparation

gRNA designed with CHOP CHOP (https://chopchop.cbu.uib.no/)
Oligo sequences generated via (http://nebiocalculator.neb.com/#!/sgrna)
Oligos ordered from IDT (www.idtdna.com)

In the case of HIV-1 due to the large amount of variation generally seen within proviruses we found it necessary to generate guides and primers tailored to the patient. We first carried out nested PCR using 250 ng of template DNA to amplify the regions upstream and downstream of the 5' and 3' LTR. The resultant PCR products were then sequenced via Nanopore (Illumina could equally be used). A consensus of the resultant reads was then used to select amongst the existing HIV-1 guides and primers we had already generated or when necessary to design new ones using CHOP CHOP and Primer 3.

Primers used in HIV-1

A1mod2	1st	U5-623F	AAATCTCTAGCAGTGGCGCCCGAACAG
		NE1	CCACTAACTTCTGTATGTCATTGACAGTCCAGCT
	2nd	U5-638F	GCGCCCGAACAGGGACYTGAAARCGAAAG
		ProC-	GAGTATTGTATGGATTTTCAGGCCCAAT
B2	1st	GP41Fo	TTCAGACCTGGAGGAGGAGATAT
		3LTRi	TCAAGGCAAGCTTTATTGAGGCTTAA
	2nd	GP41Fi	GGACAATTGGAGAAGTGAATTAT
		3UTRi	AGGCTTAAGCAGTGGGTTCCCTAG

gRNAs generated using EnGen sgRNA Synthesis kit, S. pyogenes, New England Biolabs #E3322S

Following the manufacturer's recommendations assemble reaction at RT, in the order listed. For preparation of a pool of guide RNAs, take 5 µL of each guide oligo and pool. Then dilute 5 µL of the pool in 495 µl of H20 to make a 1 µM solution. Use this for the following steps.

H20	2 µL
Engen 2X reaction mix	10 µL
Guide Oligo (1 µM)	5 µL
DTT (0.1M)	1 µL
Engen sgRNA enzyme mix	2 µL

Total volume 20 µL

Mix thoroughly - Spin down – incubate at 37°C for 30 minutes
Transfer on ice
Add 30 µL of H20 (volume up to 50)
Add 2 µL of DNAse I (RNAse free, provided)
Mix – incubate at 37°C for 15 minutes
Purify with 2X **RNAClean XP kit, Beckman Coulter, #A63987**
Quantify the purified guide RNAs on a nanodrop spectrophotometer and keep them on ice

Library preparation Day 1

1) **Shear DNA in 8KB fragments using covaris g-tubes**
Ref - http://covaris.com/products/g-tube/#tab-id-1

Optional. Remove small DNA fragments using 0.8X AMPure XP beads, Beckman Coulter, #A63881.

Dilute DNA in 75 µL (typically 5 µg but can be scaled up or down) and transfer in covaris tubes.
Centrifuge the covaris tube at 7200 RPM for 1 min placing the tube straight and another minute placing it upside down in the centrifuge. Sheared material will be collected in the covaris tube cap.

Note: Check quality and size of the starting DNA and of the sheared DNA on a 1% agarose gel, loading an aliquot of sheared and unsheared DNA. Shown below in ***A*** *is appropriate high molecular weight DNA to use as starting material,* ***B*** *the same DNA following shearing*

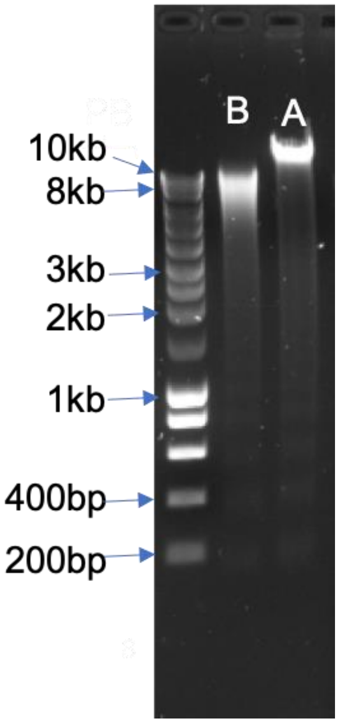

2) **End Repair the DNA fragments NEBNext EndRepair Module, New England Biolabs, #E6050**
For end repair reactions of 75 µL of DNA each, each reaction can include a maximum of 5 µg of DNA. If more than 5 µg of samples are being processed, volumes need to be scaled up.

Buffer	9.75 µL
Enzyme	4.5 µL
DNA	75 µL

Incubate at 20°C for 30 minutes.

3) **Purify DNA using AMPure XP beads 1X (90 µL) and resuspend in 30 µL of H20.**

4) **Fragment circularization via Ligation reaction, T4 DNA Ligase (New Enland Biolabs).**
Example shows reaction volumes for intramolecular circularization of 2 µg of DNA. If more input DNA is used, volumes need to be scaled up (can also be scaled down).

Blunt end repaired DNA	2 µg of DNA eluted in 260 µL
T4 Buffer	30 µL
Ligase (400.000 units/ml)	15 µL

Mix gently and incubate at 16°C overnight.

Library preparation Day 2

1) **Linear DNA digestion using Plasmid-Safe-ATP-Dependent DNAse (Epicentre, Madison WI)**
Add the enzyme directly (1 µL for 2 µg of DNA, scale up or down accordingly).
Note: the buffer of the plasmid-safe-ATP-dependent- DNAse is compatible with the T4 Ligase buffer.
Incubate at 37°C for 1 hour, then at 70°C for 30 min (heat inactivation step). Let the DNA cool down at room temperature.

2) **Purify DNA using AMPure beads 1X and resuspend in 26 µL H20.**
NOTE: nanodrop spectrophotometer does not give accurate quantification of the DNA at this point.

3) **Crispr-Cas9 Digestion of circularized DNA using NEB kit # M0386**
Each sample will be split in two separate reactions of 1 µg each, to be digested with the appropriate pools of RNA guides (pool A and pool B) targeting different portions of the target virus.

	each sample split in two	
H20	9 µL	9 µL
10x Cas9 buffer	3 µL	3 µL
Guide RNA 300 nM (10 ng/µL for our RNAs, these are about 100bp long)	4.5 µL pool A	4.5 µL pool B
1uM of Cas9 Nuclease enzyme	1.5 µL	1.5 µL
Incubate at 25°C for 10 min		
Add circularized DNA	12 µL	12 µL
Total volume	30 µL	30 µL

Mix- Spin- incubate at 37°C for 1 hour
Add 1 µL of Proteinase K
Mix- Spin – incubate at room temperature for 10 min

4) **Purify DNA using AMPure beads 1X (30 µL), resuspend in 26 µL of H20 each.**
***Note**: avoid allowing beads to dry too much, this could shear long DNA fragments.*
Aliquot 1.5 µL of sample and quantify via nanodrop spectrophotometer.
Typically ~10-15% of the starting material is recovered at this point.

Note: For following PCR reactions, the appropriate primers that flank the sites of the Guide RNAs Pool should be used.

5) **Long range PCR1 overnight using primer pairs designed to flank the specific Crispr Cas9 digestion site. LongAmp® Hot Start Taq 2X Master Mix (New England Biolabs) # M0533**

Primer working solution prepared by taking 2.5 ul of the 100 uM stock solution for each of the four primers in the pool and diluting in 190 µl H20.

Take 25 µL of LongAmp Hot Start master mix + 2 µL of primer working solution + 23 µL of sample.
(Final concentration for each primer 0.2 µM)

Program

95 °C - 00:30

95 °C – 00:30
62 °C – 00:30 } 30 cycles*
65 °C – 10:00

65 °C – 06:00
4 °C – pause

*Number of cycles can be increased or decreased, for low proviral load samples such as HIV-1 35 cycles are often required. Samples with large numbers of copies of the target virus (eg ERVs) can reduce the number of cycles to 25.

Library preparation Day 3

1) Load 10 µL of Long range PCR1 products on a 1% agarose gel.

Shown below is the part of the gel from the Supplementary Note showing 10 ul of PCR1 loaded on a 1% agarose gel. Lanes 1,2 and 4 show PCRs that produced a high molecular weight band at about ~8 kb, when sequenced this will produce a good quality library. Lane 3 shows a PCR that has a preponderance of shorter fragments, sequencing of such PCR product is not advised as these molecules are mainly derived from the host and yield very few integration sites (see the Supplementary Note table). It should be noted that lanes 3 and 4 used identical input DNA and PCR mix, about ~10% of PCR reactions (in all viruses targeted) generate these patterns of short fragment stochastically, probably due to nonspecific amplification. PCRs that produce such a pattern should be repeated. The Supplementary Note details the DNA concentrations obtained for these libraries, the number of virus/host chimeric reads and number of integration sites captured. (The products of PCR2 when run on a 1% agarose gel should produce the same pattern.)

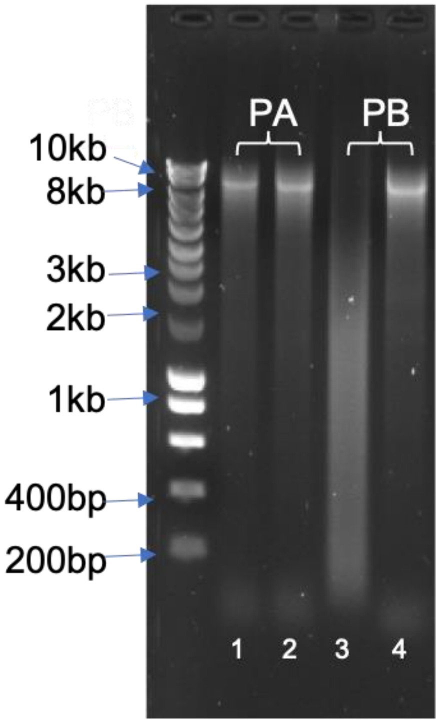

2) Purify the remaining DNA using AMPure beads 1X, resuspend in 35 µL H20.
Quantify via nanodrop spectrophotometer and calculate the volume required for 50 ng in 23 µL H20
Save PCR1 as a backup.

3) Long range indexing by PCR, LongAmp Taq DNA Polymerase (New England Biolabs) # m0323

Options: Indexing of the samples can be done via a second PCR using the PCR Barcoding Expansion KIT 1-96 (EXP-PBC096) from Oxford Nanopore or via ligation of barcodes using the Native Barcoding Expansion 1-12 (EXP-NBD104) Oxford Nanopore.

Take 25 µL of LongAmp Hot Start master mix + 2 µL of Nanopore Barcode primers at 10 µM each + 23 µL of sample containing 50ng of PCR1 product.

Program

94 °C - 00:30

94 °C – 00:20
58 °C – 00:30 } 6 cycles
65 °C – 10:00

65 °C – 07:00
4 °C – pause

4) **Load 10 µL of Long range PCR1 products on a 1% agarose gel.**

5) **Purify the remaining DNA using Ampure beads 0.8X, resuspend in 35 µL H20 each.**
Quantify via nanodrop spectrophotometer

Optional: Pool equal amont of the libraries to be sequenced and clean with using 0.4X Ampure beads to remove small fragments, then proceed to library perp

6) **Oxford Nanopore library preparation using the Ligation sequencing KIT LSK109, according to the manufacturer's instructions**

Supplementary References

1. Chen, J., Kadlubar, F. F. & Chen, J. Z. DNA supercoiling suppresses real-time PCR: a new approach to the quantification of mitochondrial DNA damage and repair. Nucleic Acids Res. 2007; 35:1377–1388

2. Durkin K, Rosewick N, Artesi M, Hahaut V, Griebel P, Arsic N, Burny A, Georges M, Van den Broeke Anne. Characterization of novel Bovine Leukemia Virus (BLV) antisense transcripts by deep sequencing reveals constitutive expression in tumors and transcriptional interaction with viral microRNAs. Retrovirology 2016; 13:1–16.
