# Supplementary material for: PCIP-seq: simultaneous sequencing of integrated viral genomes and their insertion sites with long reads
Source: Genome Biol. 2021 Apr 6;22:97. doi: 10.1186/s13059-021-02307-0 (PMC8025556; doi:10.1186/s13059-021-02307-0)
Supplement: Supplementary file 3 — Additional file 3: Dataset S2. Provirus consensus sequences. [file 13059_2021_2307_MOESM3_ESM.pdf]

Supplementary Dataset 2

Proviral sequences generated as part of the study using the medaka_consensus program (https://nanoporetech.github.io/medaka/index.html) with manual correction guided by mapping reads to consensus and inspection in IGV

pBLV344
Bos-1053_BLV_consensus
Bos-1439_BLV_consensus
HIV_U1_CHRX
HIV_U1_CHR2
02006_consensus, consensus sequence of the proviruses from patient 02006
02006_chr10_sc, sequence of the provirus with ID chr10:41845251-42110306*
02006_cen, sequence of the provirus with ID chr13:16283554-17732159*
BosT_ERV_chr11_APOB

*these two consensus sequences were generated using a limited number of Nanopore reads and as a consequence there is a higher change that they may contain errors.

>pBLV344 8721 bp

TGTATGAAAGATCATGCCGGCCTAGGCGCCGCCACCGCCCCGTAAACCAGACAGAGACGTCAGCT
GCCAGAAAAGCTGGTGACGGCAGCT

GGTGGCTAGAATCCCCGTACCTCCCCAACTTCCCCTTTCCCGAAAAATCCACACCCCGAGCTGCTG
ACCTCACCTGCTGATAAAACAATA

AAATGCCGGCCCTGTCGAGTTAGCGGCACCAGAAGCGTTCTCCTCCTGAGACCCTCGTGCTCAGC
TCTCGGTCCTGAGCTCTCTTGCTCC

CGAGACCTTCTGGTCGGCTATCCGGCAGCGGTCAGGTAAGGCAAACCACGGTTTGGAGGGTGGTT
CTCGGCTGAGACCACCGCGAGCTCT

ATCTCCGGTCCTCTGACCGTCTCCACGTGGACTCTCTCTCTTGCCTCCTGACCCCGCGCTCCAAGG
GCGTCTGGCTTGCACCCGCGCTTG

TTTCCTGTCTTACTTTCTGTTTCTCGCGGCCCGCGCTCTCTCCTTCGGCGCCCTCTAGCGGCCAGG
AGAGACCGGCAAACAATTGGGGGC

TCGTCCGGGATTGATCACCCCGGAACCCTAACAATCCTCTGGACCCACCCCCTCGGCGGCGTTTT
GGGTCTTTCCTTTAAATTATATCAT

GGGAAATTCCCCCTCCTATAACCCCCCCGCTGGTATCTCCCCCTCAGACTGGCTCAACCTTTTGCA
AAGCGCGCAAAGGCTCAATCCGCG

ACCCTCTCCTAGCGATTTTACCGATTTAAAAAATTACATCCATTGGTTTCATAAGACCCAGAAAAAAC
CATGGACTTTCACTTCTGGTGG

CCCCGCCTCATGCCCACCCGGGAAATTCGGCCGGGTTCCCCTTGTCTTGGCCACCCTAAACGAAG
TGCTCTCAAACGATGAGGGCGCCCC

GGGTGCATCGGCCCCAGAAGAACAACCCCCCCCTTATGACCCCCCCGCCGTTTTGCCAATCATAT
CTGAAGGGAATCGCAACCGCCATCG

CGCTTGGGCACTCCGAGAATTACAAGATATTAAAAAAGAAATTGAAAATAAGGCACCGGGTTCGCA
AGTATGGATACAAACACTACGACT

TGCAATCTTGCAGGCCGACCCTACTCCTGCTGACCTAGAACAACTTTGCCAATATATTGCTTCCCCG
GTCGATCAAACGGCCCACATGAC

CAGCCTAACGGCAGCAATAGCAGCCGCTGAAGCGGCCAACACCCTTCAGGGTTTTAATCCCCAAA
ACGGGACCCTGACCCAACAATCAGC

TCAGCCCAACGCCGGGGATCTTAGAAGTCAATATCAAAACCTTTGGCTTCAGGCCTGGAAAAATCT
CCCTACTCGTCCTTCAGTACAACC

CTGGTCCACCATCGTCCAAGGCCCCGCCGAGAGCTATGTAGAGTTTGTCAACCGGTTACAAATTTC
ATTAGCTGACAACCTTCCCGACGG

AGTCCCTAAAGAACCCATTATTGACTCCCTTAGCTATGCTAATGCTAACAAAGAATGCCAACAAATTT
TGCAGGGGCGGGGCCTAGTGGC

CGCCCCGGTGGGACAAAAACTGCAGGCTTGTGCACATTGGGCCCCCAAGATTAAACAGCCTGCAA
TCCTCGTCCACACCCCAGGGCCCAA

GATGCCCGGGCCTCGGCAACCGGCCCCCAAAAGGCCCCCCCCGGGACCATGCTATCGATGCCTC
AAAGAAGGCCATTGGGCCCGGGACTG

TCCCACCAAGACCACCGGCCCCCCTCCGGGACCTTGTCCCATATGCAAAGATCCTTCCCATTGGAA
ACGAGACTGTCCAACCCTCAAATC

AAAAAACTAATAGAGGGGGGACTTAGCGCCCCCCAAACCGTAACCCCTATAACAGATCCTCTTAGT
GAGGCTGAATTGGAATGCTTACTT

TCTATTCCTCTGGCTCGCAGCCGTCCCTCCGTGGCTGTATACCTGTCTGGCCCCTGGCTGCAGCC
CTCTCAGAATCAAGCCCTTATGCTC

GTGGACACCGGGGCTGAAAATACGGTCCTCCCACAAAATTGGCTGGTTCGAGATTACCCACGGAT
CCCCGCCGCAGTGCTCGGAGCGGGG

GGAGTCTCCCGGAACAGATACAATTGGCTACAAGGCCCTCTGACCCTGGCTCTAAAACCAGAGGG
TCCCTTTATCACCATCCCAAAAATT

TTAGTTGACACTTTCGATAAATGGCAAATTTTAGGACGGGACGTCCTCTCCCGCCTACAGGCCTCTA
TCTCCATACCTGAGGAGGTACGC

CCCCCCATGGTAGGCGTCCTAGATGCCCCCCCGAGCCACATTGGATTAGAACATTTGCCCGCCCC
ACCTGAGGTACCTCAATTCCCTTTA

AACTAGAACGCCTCCAGGCCCTTCAAGACCTGGTCCATCGCTCTCTGGAGGCAGGTTATATCTCCC
CCTGGGACGGGCCAGGCAATAATC

CAGTATTCCCGGTACGGAAACCAAATGGCACCTGGAGGTTTGTGCATGATCTACGAGCTACAAATG
CTCTTACAAAGCCCATCCCGGCGC

TCTCCCCCGGACCGCCAGACCTTACCGCTATCCCTACACACCTTCCACATATCATTTGCCTAGATCT
CAAAGATGCCTTCTTCCAGATTC

CAGTCGAAGACCGCTTCCGCTCCTATTTTGCTTTTACCCTCCCTACCCCCGGGGGACTCCAACCTC
ATAGACGCTTTGCCTGGCGGGTCC

TACCTCAAGGCTTCATTAATAGCCCGGCTCTTTTCGAACGGGCACTACAGGAACCCCTTCGCCAAG
TTTCCGCCGCCTTCTCCCAGTCTC

TTCTGGTGTCCTATATGGACGATATCCTTATCGCTTCGCCTACAGAAGAACAACGGTCACAATGTTA
TCAAGCCCTGGCTGCCCGCCTCC

GGGACCTAGGGTTTCAGGTGGCGTCTGAAAAGACTCGCCAGACGCCTTCGCCCGTCCCCTTCCTG
GGACAAATGGTCCATGACCAGATTG

TCACCTATCAGTCCCTACCTACCTTGCAGATCTCATCCCCAATTTCTCTTCACCAATTACAGGCGGT
CTTGGGAGACCTCCAGTGGGTCT

CCAGGGGCACACCTACTACCCGCCGACCCCTGCAACTTCTCTACTCTTCCCTTAAAGGCATCGATG
ACCCTAGGGCCACCATCCAGCTTT

CCCCGGAACAGCTACAAGGCATTGCAGAGCTTCGACAAGCCCTGTCCCATAACGCAAGATCTAGAT
ATAACGAGCAAGAACCCCTGCTGG

CCTACATACACCTAACCCGGGCGGGGTCCACCCTGGTACTCTTCCAAAAGGGCGCTCAATTTCCCC
TGGCCTACTTTCAGACCCCCTTGA

CTGACAACCAAGCCTCACCTTGGGGCCTCCTTCTCCTGCTGGGATGCCAATACCTGCAGACTCAG
GCCTTAAGCTCTTATGCCAAGCCCA

TACTCAAATACTATCACAATCTTCCTAAAACCTCTCTCGACAATTGGATTCAATCATCTGAGGACCCT
CGAGTTCAGGAGTTGTTGCGAT

TGTGGCCCCAGATTTCCTCTCAGGGAATACAGCCCCCGGGCCCCTGGAAGACCTTGATCACCAGG
GCAGAGGTTTTTTTGACGCCCCAGT

TCTCTCCTGAACCGATTCCTGCGGCCCTTTGCCTCTTTAGTGACGGGGCTACAGGACGAGGAGCAT
ATTGCCTGTGGAAAGACCACCTTT

TGGACTTTCAGGCCGTTCCGGCTCCAGAGTCCGCCCAAAAGGGAGAACTAGCAGGTCTCTTGGCG
GGCTTAGCAGCCGCCCCGCCTGAAC

CTTTAAATATATGGGTAGATTCCAAATACCTATACTCCTTGCTCAGAACCCTAGTTCTGGGAGCTTG
GCTTCAACCTGACCCCGTACCCT

CCTATGCCCTCCTATACAAAAGCCTCCTCCGACATCCAGCAATCTTTGTTGGTCATGTCCGGAGCC
ACTCCTCAGCATCCCACCCTATTG

CTTCCCTGAACAATTATGTAGATCAACTGCTCCCCTTAGAAACTCCAGAGCAATGGCATAAGCTCAC
CCACTGCAACTCTCGGGCCTTGT

CTCGATGGCCGAACCCACGTATTTCGGCCTGGGATCCCCGTTCCCCCGCTACGCTATGTGAAACC
TGTCAAAAGCTCAATCCAACTGGAG

GTGGAAAGATGCGAACTATTCAGAGAGGGTGGGCCCCGAATCATATTTGGCAGGCCGATATAACC
CATTATAAATACAAACAGTTCACCT

ACGCTTTGCACGTGTTTGTAGATACTTACTCTGGAGCTACTCATGCCTCAGCAAAGCGAGGGCTCA
CCACTCAAATGACCATTGAGGGCC

TTCTGGAGGCCATAGTACATCTGGGTCGTCCAAAAAAGCTAAACACTGACCAAGGCGCAAACTACA
CCTCCAAAACCTTTGTCAGGTTTT

GCCAGCAGTTCGGAGTTTCCCTTTCTCATCACGTTCCCTACAACCCCACAAGTTCAGGGTTGGTAG
AACGGACAAATGGACTGCTCAAAC

TTCTTTTGTCTAAATATCACCTAGACGAACCCCACCTTCCCATGACTCAGGCCCTTTCTCGAGCCCT
CTGGACTCACAATCAGATTAACC

TCCTGCCAATTCTAAAGACCAGATGGGAGTTACACCATTCACCCCTACTTGCTGTCATTTCAGAGGG
CGGAGAAACACCCAAGGGCTCTG

ATAAACTCTTTTTGTACAAGCTCCCCGGGCAAAACAATCGTCGGTGGCTAGGACCACTCCCGGCCC
TAGTCGAAGCCTCGGGAGGCGCCC

TCCTGGCTACTAACCCCCCCGTGTGGGTTCCCTGGCGTTTGCTAAAAGCCTTCAAATGCCCAAAGA
ACGACGGTCCCGAAGACGCCCACA

ACCGATCATCAGATGGGTAAGTCTCACTCTTACTCTCCTCGCTCTCTGTCAGCCCATCCAGACTTG
GAGATGCTCCCTGTCCCTAGGAAA

TCAACAATGGATGACAACATATAACCAAGAGGCAAAATTTTCCATCGCCATTGACCAAATACTAGAG
GCTCATAATCAATCGCCTTTCTG

TCCCAGGTCTCCCAGATACACCTTGGACTTTGTAAATGGTTATCCTAAGATCTATTGGCCCCCCCCA
CAAGGGCGACGCCGGTTTGGAGC

CAGGGCCATGGTCACATATGATTGCGAGCCCCGATGCCCTTATGTGGGGGCAGATCACTTCGACT
GCCCCCACTGGGACAATGCTTCCCA

GGCCGATCAAGGGTCCTTTTATGTCAATCATCAGATTTTATTCCTGCATCTCAAACAATGTCATGGA
ATTTTCACTCTAACCTGGGAAAT

ATGGGGATATGATCCCCTGATCACCTTTTCTTTACATAAAATCCCTGATCCCCCTCAACCCGACTTC
CCTCAGCTGAACAGTGACTGGGT

TCCCTCTGTCAGGTCATGGGCCCTGCTTTTAAATCAAACGGCACGGGCCTTCCCAGACTGTGCTAT
ATGTTGGGAACCTTCCCCTCCCTG

GGCTCCCGAAATATTAGTATATAACAAAACCATCTCCAACTCTGGACCCGGTCTCGCCCTCCCGGA
CGCCCAAATCTTCTGGGTCAACAC

GTCCTTGTTTAACACCACCCAAGGATGGCACCACCCTTCCCAGAGGTTGTTGTTCAACGTTTCTCAA
GGCAACGCCTTATTATTACCCCC

TATCTCCCTGGTTAATCTCTCTACGGCTTCCTCCGCCCCTCCTACCCGGGTCAGACGCAGTCCTGC
CGCAGCCCTGACCTTGGGCCTAGC

CCTGTCAGTGGGGCTCACTGGAATTAATGTAGCCGTGTCCGCCCTTAGCCATCAGAGACTCACCTC
CCTGATCCACGTTCTGGAGCAAGA

TCAGCAACGCTTGATCACAGCAATTAACCAGACCCACTATAATTTGCTTAATGTGGCCTCTGTGGTC
GCCCAGAACCGACGGGGGCTAGA

TTGGTTGTACATCCGGCTGGGTTTTCAAAGCCTATGTCCCACGATCAATGAACCTTGCTGTTTCCTG
CGCATTCAAAATGACTCCATTAT

CCGCCTCGGTGATCTCCAGCCTCTCTCGCAAAGAGTCTCTACAGACTGGCAATGGCCCTGGAATTG
GGATCTGGGGCTCACCGCCTGGGT

GCGAGAAACCATTCATTCTGTTCTAAGCCTATTCCTATTAGCCCTTTTTTTGCTCTTCTTGGCCCCCT
GCCTGATAAAATGCTTGACCTC

TCGCCTTTTAAAACTCCTCCGGCAGGCTCCCCACTTCCCTGAAATCTCCTTCCCCCCTAAACCCGAT
TCTGATTATCAGGCCTTGCTACC

ATCCGCGCCAGAGATCTACTCTCACCTCTCCCCCACCAAACCCGATTACATCAACCTTCGACCCTG
CCCTTGACACCCCCATGTTTCACG

CACCCTCAGGCTGTGGTGGGGCACTGGCTTAGTGGAATAGTCAGTGTACCATCACAAGCCTCTTCT
TGCTGCCAGCGCCGAGTTCGAACA

CAGCCCTACCCTGAGCCTCTCTGAGTGCATGACTGAGTGTAGCGCAGAGAGATTGTCGCTTCTGC
GTGTCACTCAGTCATTTTTTATAGC

CGATTGGGGTTCGCGCCCTCCCGTTGCCTGTGACACGGTTAAGACCTCTCTCACTTCTGCTTCACC
ATCCCCCTGCCAGCGTTGGTCTAG

TGGAAAGAACTAACGCTGACGGGGGCGATTTCTTGCAGCTGTGCTAAGCGAGAGGCTCTGGTGCT
GGGGATAAGATGCGGCCCCTAGCAC

CACAGTCTCTGCGCCTTTTGGGTTCGAATCTTCCCCATGCAGCTTCCGCTTTTTACGCCCTGTTGCA
CACCCTTTCTAGAGATACCTGAA

AATCTCAGCTCGCACCCCAAGGAAGGTTGTGGCTCAGAGGTTAAAATAGCTCGGACCGCAACCTC
CCTTTCTTTTTATTCCACCCTCGCA

AGGCCCCGGGTTCTAGACCCCCTAACGGAGGTTCAAAATTTCCTCTACTAGGGGGTGCTCAGGTC
CAAGTGTGCACAACATCTCTTCCAA

AAGGTCCTGATGAACATCTTCCCATGTAACAAGCCCCAGCAGAGACATTCCAGCCACATCCAGCAG
CATTTGGGCCGCCTTCTCTAACAG

TGCCCATAAAGTCCCTTCTGTTTCCACAACGGCTGCCTCTGCATCTTCTATTTCCACCTCGGCACCG
ACTCCCCCGCCGAGCCCTTCAAG

CTCTTCGGGATCCATTACCTGATAACGACAAAATTATTTCTTGTCTTTTAAGCAAGTGTTGTTGGTTG
GGGGCCCCACTCTCTACATGCC

TGCCCGGCCCTGGTTTTGTCCAATGATGTCACCATCGATGCCTGGTGCCCCCTCTGCGGGCCCCA
TGAACGACTCCAATTCGAAAGGATC

GACACCACGCTCACCTGCGAGACCCACCGTATCACCTGGACCGCCGATGGACGACCTTTCGGCCT
CAATGGAACGTTGTTCCCTCGACTG

CATGTCTCCGAGACCCGCCCCCAAGGGCCCCGACGACTCTGGATCAACTGCCCCCTTCCGGCCGT
TCGCGCTCAGCCCGGCCCGGTTTCA

CTTTCCCCCTTCGAGCAGTCCCCCTTCCAGCCCTACCAATGCCAATTGCCCTCGGCCTCTAGCGAT
GGTTGCCCCATCATCGGGCACGGC

CTTCTTCCCTGGAACAGCTTAGTAACGCATCCTGTCCTCGGAAAAGTCCTTACATTAAATCAAATGG
CCAATTTTTCCTTACTCCCCCCC

TTCGATACCCTCCTTGTGGACCCCCTCCGGCTGTCCGTCTTTGCCCCGGACACTAGGGGAGCCAT
ACGTTATCTCTCCACCCTTTTGACG

CTATGCCCAGCTACTTGTATTCTACCCCTAGGCGAGCCCTTCTCTCCTAATGTCCCCATATGCCGCT
TTCCCCGGGACACCAATGAACCT

CCCCTTTCAGAATTCGAGCTGCCCCTTATCCAAACGCCCGGCCTGTCTTGGTCTGTCCCCGCGATC
GACCTATTCCTAACCGGTCCCCCT

TCCCCATGCGACCGGTTACACGTGTGGTCCAGTCCTCAGGCCTTACAACGCTTCCTCCATGACCCC
ACGCTCACCTGGTCAGAATTGGTT

GCTAGCGGGAAACTAAGACTTGATTCACCCTTAAAATTACAGCTGTTAGAAAATGAATGGCTCTCCC
GCCTTTTTTGAGGGGGAGTCATT

TGTATGAAAGATCATGCCGGCCTAGGCGCCGCCACCGCCCCGTAAACCAGACAGAGACGTCAGCT
GCCAGAAAAGCTGGTGACGGCAGCT

GGTGGCTAGAATCCCCGTACCTCCCCAACTTCCCCTTTCCCGAAAAATCCACACCCCGAGCTGCTG
ACCTCACCTGCTGATAAAACAATA

AAATGCCGGCCCTGTCGAGTTAGCGGCACCAGAAGCGTTCTCCTCCTGAGACCCTCGTGCTCAGC
TCTCGGTCCTGAGCTCTCTTGCTCC

CGAGACCTTCTGGTCGGCTATCCGGCAGCGGTCAGGTAAGGCAAACCACGGTTTGGAGGGTGGTT
CTCGGCTGAGACCACCGCGAGCTCT

ATCTCCGGTCCTCTGACCGTCTCCACGTGGACTCTCTCTCTTGCCTCCTGACCCCGCGCTCCAAGG
GCGTCTGGCTTGCACCCGCGCTTG

TTTCCTGTCTTACTTTCTGTTTCTCGCGGCCCGCGCTCTCTCCTTCGGCGCCCTCTAGCGGCCAGG
AGAGACCGGCAAACA

>Bos-1053_BLV_consensus 8720 bp

TGTATGAAAGATCATGCCGACCTAGGCGCCGCCACCGCCCCGTAAACCAGACAGAGACGTCAGCT
GCCAGAAAAGCTGGTGACGGCAGCT

GGTGGCTAGAATCCCCGTACCTCCCCAACTTCCCCTTTCCCGAAAAATCCACACCCTGAGCTGCTG
ACCTCACCTGCTGATAAACTAATA

AAATGCCGGCCCTGTCGAGTTAGCGGCACCAGAAGCGTTCTTCTCCTGAGACCCTCGTGCTCAGC
TCTCGGTCCTGAGCTCTCTTGCTCC

CGAGACCTTCTGGTCGGCTATCCGGCAGCGGTCAGGTAAGGCAAACCACGGTTTGGAGGGTGGTT
CTCGGCTGAGACCACCGCGAGCTCT

ATCTCCGGTCCTCTGACCGTCTCCACGTGGACTCTCTCCTTTGCCTCCTGACCCCGCGCTCCAAGG
GCGTCTGGCTTGCACCCGCGTTTG

TTTCCTGTCTTACTTTCTGTTTCTCGCGGCCCGCGCTCTCTCCTTCGGCGCCCTCTAGCGGCCAGG
AGAGACCGGCAAACAATTGGGGGC

TCGTCCGGGATTGATCACCCCGGAACCCTAACAACTCTCTGGACCCACCCCCTCGGCGGCATTTT
GGGTCTCTCCTTCAAATTATATCAT

GGGAAATTCCCCCTCCTATAACCCCCCCGCTGGTATCTCCCCCTCAGACTGGCTCAACCTTCTGCA
AAGCGCGCAAAGGCTCAATCCGCG

ACCCTCTCCTAGCGATTTTACCGATTTAAAGAATTACATCCATTGGTTTCATAAGACCCAGAAAAAAC
CATGGACTTTCACTTCTGGTGG

CCCCACCTCATGTCCACCCGGGAGATTCGGCCGGGTTCCCCTTGTCTTGGCCACCCTAAACGAAG
TGCTCTCAAACGAAGGGGGCGCCCC

GGGTGCATCGGCCCCAGAAGAACAACCCCCCCCTTATGACCCCCCCGCCGTTTTGCCAATCATAT
CTGAAGGGAATCGCAACCGCCATCG

TGCTTGGGCACTCCGAGAATTACAAGATATCAAAAAGGAAATTGAAAATAAGGCACCGGGTTCGCA
AGTATGGATACAAACACTACGACT

TGCAATCCTGCAGGCCGACCCTACTCCGGCTGACTTAGAACAACTTTGCCAATATATTGCTTCCCC
GGTCGACCAAACGGCCCATATGAC

CAGCCTAACGGCAGCAATTGCCGCCGCTGAAGCGGCCAACACCCTCCAGGGTTTTAACCCCCAAA
ACGGGACCCTAACCCAACAATCAGC

TCAGCCCAACGCCGGGGATCTTAGAAGTCAATATCAAAACCTCTGGCTTCAGGCCTGGAAAAATCT
CCCTACTCGTCCTTCAGTACAACC

TTGGTCCACCATCGTCCAAGGCCCCGCCGAAAGCTATGTAGAGTTTGTCAACCGGTTACAAATTTC
ATTAGCTGACAACCTTCCCGACGG

AGTCCCTAAGGAACCCATTATTGACTCCCTTAGTTATGCAAATGCTAACAAAGAGTGTCAGCAAATT
TTGCAAGGGCGAGGCCTAGTGGC

CGCCCCGGTGGGGCAAAAGCTGCAGGCTTGCGCACATTGGGCCCCCAAGATGAAACAGCCTGCA
ATTCTCGTCCACACCCCAGGGCCCAA

GATGCCCGGGCCTCGGCAACCGGCCCCCAAAAGGCCTCCCCCAGGACCATGCTATCGATGCCTC
AAAGAAGGCCATTGGGCCCGGGATTG

TCCTACCAAGGCCACCGGCCCCCCTCCGGGACCTTGCCCCATATGTAAAGATCCTTCCCATTGGAA
ACGAGACTGTCCAACCCTCAAATC

AAAAAACTAATAGAGGGGGGACTTAGCGCCCCCCAAACCATAACACCCATAACAGATTCTCTTAGT
GAGGCCGAATTAGAATGCTTACTT

TCTATTCCTCTGGCTCGCAGCCGTCCCTCCGTGGCTGTATACCTGTCTGGCCCCTGGCTGCAGCC
CTCTCAGAATCAAGCCCTCATGCTT

GTGGACACCGGGGCTGAAAATACGGTTCTCCCACAAAATTGGCTGGTTCGGGATTACCCACGGAT
CCCCGCCGCAGTGCTCGGAGCAGGG

GGAGTCTCCCGGAACAGATACAATTGGCTACAAGGCCCTCTGACCCTGGCTCTAAAACCAGAGGG
TCCCTTTATCACCATCCCAAAAATT

TTAGTTGACACTTTCGACAAATGGCAAATTTTAGGACGGGACGTCCTCTCCCGCCTACAGGCCTCT
ATCTCCATACCTGAGGAAGTACGC

CCCCCTGTGGTAGGCGTCTTGGATGCCCCCCCGAGCCACATTGGATTAGAACATCTGCCCCCTCC
ACCTGAGGTGCCTCAATTCCCTTTA

AACTAGAACGCCTCCAGGCCCTTCAAGACCTGGTCCATCGCTCTCTGGAGGCAGGTTATATCTCCC
CCTGGGACGGGCCAGGCAATAATC

CAGTCTTCCCGGTACGGAAACCAAATGGCGCCTGGAGGTTTGTGCATGACCTACGAGCTACAAATG
CTCTTACAAAGCCCATTCCGGCAC

TCTCCCCCGGACCGCCAGACCTTACCGCTATCCCTACACACCTTCCACATATCATTTGCCTAGATCT
CAAAGACGCCTTCTTCCAGATTC

CAGTCGAAGACCGCTTCCGCTCCTACTTTGCTTTTACCCTCCCTACCCCCGGGGGACTCCAACCTC
ATAGACGCTTTGCCTGGCGGGTCC

TACCTCAAGGCTTCATTAACAGCCCAGCTCTTTTCGAACGAGCACTACAGGAACCTCTTCGCCAAG
TTTCCGCCGCCTTTTCCCAGTCTC

TTCTGGTGTCCTATATGGACGATATCCTTATCGCTTCGCCTACAGAAGAACAGCGGTCACAATGTTA
TCAAGCCCTGGCTGCCCGCCTCC

GGGACCTAGGGTTTCAGGTGGCATCCGAAAAGACTCGCCAGACGCCTTCGCCCGTCCCCTTCTTG
GGACAAATGGTCCATGAGCAGATTG

TCACCTACCAGTCCCTACCTACCTTGCAGATCTCATCCCCAATTTCTCTTCACCAATTACAGGCGGT
CTTAGGAGACCTCCAATGGGTCT

CTAGGGGCACACCCACTACCCGCCGGCCCCTGCAACTTCTCTACTCTTCCCTTAAAGGCATCGATG
ACCCTAGGGCCATCATCCAGCTTT

CCCCGGAACAGCTGCAAGGCATTGCAGAGCTTCGACAAGCCCTGTCCCATAACGCAAGATCTAGA
TATAACGAGCAAGAACCCCTGCTAG

CCTACGTACACCTAACCCGGGCGGGGTCCACCCTGGTACTCTTCCAAAAGGGCGCTCAATTTCCC
CTGGCCTACTTCCAGACCCCCTTGA

CTGACAACCAAGCCTCACCTTGGGGCCTCCTTCTCCTGCTGGGATGCCAATACCTGCAGACTCAG
GCCTTAAGCTCGTATGCCAAGCCCA

TACTTAAATATTATCACAATCTTCCTAAAACTTCTCTAGACAATTGGATTCAATCATCTGAGGACCCT
CGAGTCCAGGAGTTGCTGCAAT

TGTGGCCCCAGATTTCCTCTCAGGGAATACAGCCCCCGGGCCCTTGGAAGACCTTAATCACCAGG
GCAGAGGTTTTTTTGACGCCCCAGT

TCTCCCCTGATCCGATTCCTGCGGCCCTTTGCCTCTTTAGTGACGGGGCTAAAGGACGAGGAGCAT
ATTGCTTGTGGAAAGACCACCTTT

TAGACTTTCAGGCCGTTCCGGCTCCAGAATCCGCTCAAAAGGGAGAACTAGCAGGTCTCTTGGCG
GGCTTGGCAGCCGCCCCGCCTGAAC

CTGTAAATATATGGGTAGATTCCAAATACCTGTACTCTTTGCTCAGAACCCTAGTTCTGGGAGCTTG
GCTTCAACCTGACCCCGTACCCT

CCTACGCCCTCCTATATAAAAGCCTCCTCCGACATCCAGCAATCTTTGTTGGCCATGTCCGGAGCC
ACTCTTCAGCATCCCACCCTATTG

CTTCCCTGAACAATTATGTAGATCAACTGCTTCCCTTAGAAACTCCAGAGCAATGGCATAAGCTCAC
CCACTGCAACTCTCGGGCCTTGT

CTCGATGGCCGAACCCACGTATCTCTGCCTGGGACCCCCGTTCCCCCGCTACGCTGTGTGAAACC
TGTCAAAAGCTTAATCCAACTGGAG

GAGGAAAGATGCGAACTATTCAGAGAGGGTGGGCCCCGAACCATATTTGGCAGGCCGATATAACC
CATTATAAATACAAACAGTTCACCT

ACGCTCTGCATGTGTTTGTAGATACTTACTCTGGAGCTACTCATGCCTCGGCGAAGCGTGGGCTCA
CCACTCAAATGACCATTGAGGGCC

TTCTTGAGGCCATAGTGCATCTGGGTCGTCCAAAAAGGCTAAACACTGACCAAGGTGCAAACTACA
CCTCCAAAACCTTTGTCAGGTTTT

GCCAGCAGTTCGGAGTTTCCCTTTCTCATCATGTTCCCTACAACCCCACAAGTTCGGGGTTAGTAG
AACGGACAAATGGACTGCTCAAAC

TTCTTCTATCTAAATATCACCTAGACGAACCCCACCTTCCCATGACTCAGGCCCTTTCTCGAGCCCT
CTGGACTCACAATCAGATTAACC

TCCTACCAATTCTAAAGACCAGATGGGAGCTACACCATTCACCCCCACTTGCTGTCATTTCAGAGG
GCGGAGAAACACCCAAGGGCTCTG

ATAAACTCTTTTTGTACAAGCTCCCCGGGCAAAACAATCGTCGGTGGCTAGGACCACTCCCGGCCC
TAGTCGAAGCCTCGGGAGGCGCTC

TCCTGGCTACTGACCCCCCCGTGTGGGTTCCCTGGCGTTTGCTGAAAGCCTTCAAATGCCTAAAGA
ACGACGGTCCCGAAGACGCCCACA

ACCGATCATCAGATGGGTAAGTCTCACTCTCACTCTCCTCGCTCTCTCTCGGCCCATCCAGACTTG
GAGATGCTCCCTGTCCCTAGGAAA

CCAACAATGGATGACAGCATATAACCAAGAGGCAGAATTTTCCATCTCCATTGACCAAATACTAGAG
GCTCATAATCAGTCACCTTTCTG

TGCCAAGTCTCCCAGATACACCTTGGACTCTGTAAATGGCTATCCTAAGATCTACTGGCCCCCCCC
ACAAGGGCGGCGCCGGTTTGGAGC

CAGGGCCATGGTCACATATGATTGCGAGCCCCGATGCCCTTATGTGGGGGCAGATCGCTTCGACT
GCCCCCACTGGGACAATGCCTCCCA

GGCCGATCAAGGATCCTTTTATGTCAATCATCAGATTTTATTCCTGCATCTCAAACAATGTCATGGAA
TTTTCACTCTAACCTGGGAGAT

ATGGGGATATGATCCCCTGATCACCTTTTCTTTACATAAGATCCCTGATCCCCCTCAACCCGACTTT
CCCCAGTTGAACAGTGACTGGGT

TCCCTCTGTCAGATCATGGGCCCTGCTTTTAAATCAAACAGCACGGGCCTTCCCAGACTGTGCTAT
ATGTTGGGAACCTTCCCCTCCCTG

GGCTCCCGAAATATTAGTATATAACAAAACCATCTCCAGCTCTGGACCCGGCCTCGCCCTCCCGGA
CGCCCAAATCTTCTGGGTCAACGC

GTCCTCGTTTAACACCACCCAAGGATGGCACCACCCTTCCCAGAGGTTGTTGTTCAATGTTTCTCAA
GGCAACGCCTTGTTATTACCTCC

TATCTCCCTGGTTAATCTCTCTACGGCTTCCTCCGCCCCTCCTACCCGGGTCAGACGTAGTCCCGT
CGCAGCCCTGACCTTAGGCCTAGC

CCTGTCAGTGGGGCTCACTGGAATTAATGTGGCCGTGTCTGCCCTTAGCCATCAGAGACTCACCTC
CCTGATCCACGTTCTGGAGCAAGA

TCAGCAACGCTTGATCACAGCAATTAACCAGACCCACTATAATTTGCTTAATGTGGCCTCTGTGGTT
GCCCAGAACCGACGGGGGCTTGA

TTGGTTATACATCCGGCTGGGTTTTCAAAGCCTATGTCCCACAATTAATGAGCCTTGCTGTTTCCTG
CGCATTCAAAATGACTCCATTAT

CCGCCTCGGTGATCTCCAGCCTCTCTCGCAAAGAGTCTCTACAGACTGGCAGTGGCCCTGGAATT
GGGATCTGGGGCTCACTGCCTGGGT

GCGAGAAACCATTCATTCTGTTCTAAGCCTGTTCCTATTAGCCCTTTTTTTGCTCTTCCTGGCCCCC
TGCCTGATAAAATGCTTGACCTC

TCGCCTTTTAAAGCTCCTCCGGCAGGCTCCCCACTTCCCTGAAATCTCCTTAACCCCTAAACCCGAT
TCTGATTATCAGGCCTTGCTACC

ATCTGCACCAGAGATCTACTCTCACCTCTCCCCCGTCAAACCCGATTACATCAACCTCCGACCCTG
CCCTTGATACCCCCGCGTTTCACG

CACCCCCAGGCTGTGGTGGTGCACTGGCTTAGTGGAATAGTCAGTGTACCATCACAAGCCTCTTCT
TGCTGCCGGCACCGAGTTCGAACA

CAGCCCTACCCTGAACCTCTCTGAGTGCATGACTGAGTGTAGCGCAGAGAGATTGTCGCTTCTGCG
TGTCGCTCAGTCATTTTTTATAGC

CGATTGGGGTTCGCGCCCTTCCGTTGCCTGTGACACAGATAAGACCTCTCTCACTTCTGCTTCACC
ATCCCCCTGCCAGCGTTGGTCTAG

TGGAAAGAACTAACGCTGACGGGGGCGATTTCTTGCAGCTGTGCTAGCGGGAGGCTCTGGTGCTG
GGGATAAGATGTGGCCCTTAGCACC

ACAGTCTCTGCGCCTTTTGGGTTCGAATCTTCCCCACGCAGCTTCCGCTTTTTACGCCCTGTTGCA
CACCCTTTCTAGAGATACCTGAAA

ATCTCAGCTCGCACCCTGAGGAAGGTTGTGGCTCAGAGGTTAAAATAGCTCGAGCCGCAACCTCC
CTTTCTTTTTATTCCACCCTCGCAA

GGCCCCGGGTTCTGAGCCCCCTAACGGAGGTTCAAAATTTCCTCTACTAGGGGATGCTCGGGTCC
AAGTGTGCACAATATCTCTTCCAAA

AGGTCCTGATGAACGTCTTCCCATGTAACAAGCCCCAGCAGAGACATTCCAGCCACATCCAGCAGC
ATTTGGGCCGCCTTTTCTAACAGT

GCCCATAAAGTCCCTTCCGTTTCCACAACGGCTGCCTCTGCTTCTTCTATTTCCACCTCGGCACCG
ACTCCCCCGCCGAGCCCTTCGAGC

TCTTCGGGATCCATTACCTGATAACGACAAAATTATTTCTTGTCTTTTAAGCAAGTGTTGTTGGTTGG
GGGCCCCACTCTCTACATGCCT

GCCCGGCCCTGGTTTTGTCCAATGATGTCACCATCGATGCCTGGTGCCCCCTCTGCGGGCCCCAT
AAGCGACTCCAATTCGAAAGGATCG

ACACCACGCTCACCTGCGAGACCCACCGTATCAACTGGACCGCCGATGGACGACCTTGCGGCCTC
AATGGAACGTTGTTCCCTCGACTAC

ATGTCTCCGAGACCCGCCCCCAAGGGCCCCGACGACTCTGGATCAACTGCCCCCTTCCGGCCGTT
CGCGCTCAGCCCGGCCCGGTTTCAC

TTTCCCCCTTCGAGCGGTCCCCCTTCCAGCCCTACCAATGCCAATTGCCCTCGGCCTCTAGCGACG
GTTGCCCCATTATCGGGCACGGCC

TTCTTCCCTGGAACAACTTAGTAACGCATCCTGTCCTCGGAAAAGTCCTTATATTAAATCAAATGGC
CAATTTTTCCTTACTCCCCTCCT

TCGATACCCTCCTTGTGGACCCCCTCCGGCTGTCCGTCTTTGCCCCAGACACCAGGGGAGCCATA
CGTTATCTCTCCACCCTTTTGACGC

TATGCCCAGCTACTTGTATTCTACCCCTAGGCGAGCCCTTCTCTCCTAATGTCCCCATATGCCGCTT
TCCCCGGGACTCCAATGAACCCC

CCCTTTCAGAATTCGAGCTGCCCCTTATCCAAACGCCCGGCCTGTCCTGGTCTGTCCCCGCGATCG
ACCTATTTCTAACCGGTCCCCCTT

CCCCATGCGACCGGTTACACGTATGGTCCAGTCCTCAGGCCTTACAGCGCTTCCTTCATGACCCTA
CGCTAACCTGGTCCGAATTGGTTG

CTAGCAGAAAGATAAGACTTGATTCCCCCTTAAAATTACAACTGCTAGAAAATGAATGGCTCTCCCG
CCTTTTTTGAGGGGGAGTCATTT

GTATGAAAGATCATGCCGACCTAGGCGCCGCCACCGCCCCGTAAACCAGACAGAGACGTCAGCTG
CCAGAAAAGCTGGTGACGGCAGCTG

GTGGCTAGAATCCCCGTACCTCCCCAACTTCCCCTTTCCCGAAAAATCCACACCCTGAGCTGCTGA
CCTCACCTGCTGATAAACTAATAA

AATGCCGGCCCTGTCGAGTTAGCGGCACCAGAAGCGTTCTTCTCCTGAGACCCTCGTGCTCAGCT
CTCGGTCCTGAGCTCTCTTGCTCCC

GAGACCTTCTGGTCGGCTATCCGGCAGCGGTCAGGTAAGGCAAACCACGGTTTGGAGGGTGGTTC TCGGCTGAGACCACCGCGAGCTCTA

TCTCCGGTCCTCTGACCGTCTCCACGTGGACTCTCTCCTTTGCCTCCTGACCCCGCGCTCCAAGG GCGTCTGGCTTGCACCCGCGTTTGT

TTCCTGTCTTACTTTCTGTTTCTCGCGGCCCGCGCTCTCTCCTTCGGCGCCCTCTAGCGGCCAGGA GAGACCGGCAAACA

>Bos-1439_BLV_consensus 8720 bp

TGTATGAAAGATCATGCCGACCTAGGCGCCGCCACCGCCCCGTAAACCAGACAGAGACGTCAGCT GCCAGAAAAGCTGGTGACGGCAGCT

GGTGGCTAGAATCCCCGTACCTCCCCAACTTCCCCTTTCCCGAAAAATCCACACCCTGAGCTGCTG ACCTCACCTGCTGATAAATTAATA

AAATGCCGGCCCTGTCGAGCTAGCGGCACCAGAAGCGTTCTTCTCCTGAGACCCTCGTGCTCAGC TCTCGGTCCTGAGCTCTCTTGCTCC

CGAGACCTTCTGGTCGGCTATCCGGCAGCGGTCAGGTAAGGCAAACCACGGTTTGGAGGGTGGTT CTCGGCTGAGACCACCGCGAGCTCT

ATCTCCGGTCCTCTGACCGTCTCCACGTGGACTCTCTCCTTTGCCTCCTGACCCCGCGCTCCAAGG GCGTCTGGCTTGCACCCGCGTTTG

TTTCCTGTCTTACTTTCTGTTTCTCGCGGCCCGCGCTCTCTCCTTCGGCGCCCTCTAGCGGCCAGG AGAGACCGGCAAACAATTGGGGGC

TCGTCCGGGATTGATCACCCCGGAACCCTAACAACTCTCTGGACCCACCCCCTCGGCGGCATTTT GGGTCTCTCCTTCAAATTATATCAT

GGGAAATTCCCCCTCCTATAACCCCCCCGCTGGTATCTCCCCCTCAGACTGGCTCAACCTTCTGCA AAGCGCGCAAAGGCTCAATCCGCG

ACCCTCTCCTAGCGATTTTACCGATTTAAAGAATTACATCCATTGGTTTCATAAGACCCAGAAAAAAC CATGGACTTTCACTTCTGGTGG

CCCCACCTCATGTCCACCCGGGAGATTCGGCCGGGTTCCCCTTGTCTTGGCCACCCTAAACGAAG TGCTCTCAAACGAAGGGGGCGCCCC

GGGTGCATCGGCCCCAGAAGAACAACCCCCCCCTTATGACCCCCCCGCCGTTTTGCCAATCATAT CTGAAGGGAATCGCAACCGCCATCG

TGCTTGGGCACTCCGAGAATTACAAGATATCAAAAAGGAAATTGAAAATAAGGCACCGGGTTCGCA AGTATGGATACAAACACTACGACT

TGCAATCCTGCAGGCCGACCCTACTCCGGCTGACCTAGAACAACTTTGCCAATATATTGCTTCCCC GGTCGACCAAACGGCCCATATGAC

CAGCCTAACGGCAGCAATAGCCGCCGCTGAAGCGGCCAACACCCTCCAGGGTTTTAACCCCCAAA ACGGGACCCTAACCCAACAATCCGC

TCAGCCCAACGCCGGGGATCTTAGAAGTCAATATCAAAACCTCTGGCTTCAGGCCTGGAAAAATCT CCCTACTCGTCCTTCAGTACAACC

TTGGTCCACCATCGTCCAAGGCCCCGCCGAAAGCTATGTAGAGTTTGTCAACCGGTTACAAATTTC ATTAGCTGACAACCTTCCCGACGG

AGTCCCTAAGGAACCCATTATTGACTCCCTTAGTTATGCAAATGCTAACAAAGAGTGTCAGCAAATT
TTGCAGGGGCGAGGCCTAGTGGC

CGCCCCGGTGGGGCAAAAACTGCAGGCTTGCGCACATTGGGCCCCCAAGATGAAACAGCCTGCAA
TTCTCGTCCACACCCCAGGGCCCAA

GATGCCCGGGCCTCGGCAACCGGCCCCCAAAAGGCCTCCCCCAGGACCATGCTATCGATGCCTC
AAAGAAGGCCATTGGGCCCGGGATTG

TCCTACCAAGGCCACCGGCCCCCCTCCGGGACCTTGCCCCATATGTAAAGATCCTTCCCATTGGAA
ACGAGACTGTCCAACCCTCAAATC

AAAAAACTAATAGAGGGGGGACTTAGCGCCCCCCAAACCATAACACCTATAACAGATTCTCTTAGT
GAGGCCGAATTAGAATGCTTACTT

TCTATTCCTCTGGCTCGCAGCCGTCCCTCCGTGGCTGTATACCTGTCTGGCCCCTGGCTGCAGCC
CTCTCAGAATCAAGCCCTCATGCTT

GTGGACACCGGGGCTGAAAATACGGTTCTCCCACAAAATTGGCTGGTTCGAGATTACCCACGGATC
CCCGCCGCAGTGCTCGGAGCAGGG

GGAGTCTCCCGGAACAGATACAATTGGCTACAAGGCCCTCTGACCCTGGCTCTAAAACCAGAGGG
TCCCTTTATCACCATCCCAAAAATT

TTAGTTGACACTTTCGACAAATGGCAAATTTTAGGACGGGACGTTCTCTCCCGCCTACAGGCCTCTA
TCTCCATACCTGAGGAAGTACGC

CCCCCTGTGGTAGGCGTCTTGGATGCCCCCCCGAGCCACATTGGATTAGAACATCTGCCCCCCCC
ACCTGAGGTGCCTCAATTCCCTTTA

AACTAGAACGCCTCCAGGCCCTTCAAGACCTGGTCCATCGCTCTCTGGAGGCAGGTTATATCTCCC
CCTGGGACGGGCCAGGCAATAATC

CAGTCTTCCCGGTACGGAAACCAAATGGCGCCTGGAGGTTTGTGCATGACCTACGAGCTACAAATG
CTCTTACAAAGCCCATTCCGGCAC

TCTCCCCCGGACCGCCAGACCTTACCGCTATCCCTACACACCTTCCACATATCATTTGCCTAGATCT
CAAAGATGCCTTCTTCCAGGTTC

CAGTCGAAGACCGCTTCCGCTCCTACTTTGCTTTCACCCTCCCTACCCCCGGGGGACTCCAACCTC
ATAGACGCTTTGCCTGGCGGGTCC

TACCTCAAGGCTTCATTAACAGCCCAGCTCTTTTCGAACGAGCACTACAGGAACCTCTTCGCCAAG
TTTCCGCCGCCTTTTCCCAGTCTC

TTCTGGTGTCCTATATGGACGATATCCTTATCGCTTCGCCTACAGAAGAACAGCGGTCACAATGTTA
TCAAGCCCTGGCTGCCCGCCTCC

GGGACCTAGGGTTTCAGGTGGCATCCGAAAAGACTCGCCAGACGCCTTCGCCCGTCCCCTTCTTG
GGACAAATGGTCCATGAGCAGATTG

TCACCTACCAGTCCCTACCTACCTTGCAGATCTCATCCCCAATTTCTCTTCACCAATTACAGGCGGT
CTTAGGAGACCTCCAATGGGTCT

CTAGGGGCACACCCACTACCCGCCGGCCCCTGCAACTTCTCTACTCTTCCCTTAAAGGCATCGATG
ACCCTAGGGCCATCATCCAGCTTT

CCCCGGAACAGCTGCAAGGCATTGCAGAGCTTCGACAAGCCCTGTCCCATAACGCAAGATCTAGA
TATAACGAGCAAGAACCCCTGCTAG

CCTACGTACACCTAACCCGGGCGGGGTCCACCCTGGTACTCTTCCAAAAGGGCGCTCAATTTCCC
CTGGCCTACTTTCAGACCCCCTTGA

CTGACAACCAAGCCTCACCTTGGGGCCTCCTTCTCCTGCTGGGATGCCAATACCTGCAGACTCAG
GCCTTAAGCTCGTATGCCAAGCCCA

TACTTAAATATTATCACAATCTTCCTAAAACCTCTCTAGACAATTGGATTCAATCATCTGAGGACCCT
CGAGTCCAGGAGTTGCTGCAAT

TGTGGCCCCAGATTTCCTCTCAGGGAATACAGCCCCCGGGCCCTTGGAAGACCTTAATCACCAGG
GCAGAGGTTTTTTTGACGCCCCAGT

TCTCCCCTGATCCGATTCCTGCGGCCCTTTGCCTCTTTAGTGACGGGGCTACAGGACGAGGAGCA
TATTGCTTGTGGAAAGACCACCTTT

TAGACTTTCAGGCCGTTCCGGCTCCAGAATCCGCTCAAAAGGGAGAACTAGCAGGTCTCTTGGCG
GGCTTAGCAGCCGCCCCGCCTGAAC

CTGTAAATATATGGGTAGATTCCAAATACCTGTACTCTTTGCTCAGAACCCTAGTTCTGGGAGCTTG
GCTTCAACCTGACCCCGTACCCT

CCTACGCCCTCCTATATAAAAGCCTCCTCCGACATCCAGCAATCTTTGTTGGTCATGTCCGGAGCC
ACTCTTCAGCATCCCACCCTATTG

CTTCCCTGAACAATTATGTAGATCAACTGCTTCCCTTAGAAACTCCAGAGCAATGGCATAAGCTCAC
CCACTGCAACTCGCGGGCCTTAT

CTCGATGGCCGAACCCACGTATCTCTGCCTGGGACCCCCGTTCCCCCGCTACGCTGTGTGAAACC
TGTCAAAAGCTTAATCCAACTGGAG

GAGGAAAGATACGAACTATTCAGAGAGGGTGGGCCCCGAATCATATTTGGCAGGCCGATATAACC
CATTATAAATACAAACAGTTCACCT

ACGCTCTGCATGTGTTTGTAGATACTTACTCTGGAGCTACTCATGCCTCGGCGAAGCGTGGGCTCA
CCACTCAAATGACCATTGAGGGCC

TTCTTGAGGCCATAGTGCATCTGGGTCGTCCAAAAAAGCTAAACACTGACCAAGGTGCAAACTACA
CCTCCAAAACCTTTGTCAGGTTTT

GCCAGCAGTTCGGAGTTTCCCTTTCTCATCATGTTCCCTACAACCCCACAAGTTCGGGGTTAGTAG
AACGGACAAATGGACTGCTCAAAC

TTCTTCTATCTAAATATCACCTAGACGAACCCCACCTTCCCATGACTCAGGCCCTTTCTCGAGCCCT
CTGGACTCACAATCAGATTAACC

TCCTACCAATTCTAAAGACCAGATGGGAGCTACACCATTCACCCCCACTTGCTGTCATTTCAGAGG
GCGGAGAAACACCCAAGGGCTCTG

ATAAACTCTTTTTGTACAAGCTCCCCGGGCAAAACAATCGTCGGTGGCTAGGACCACTCCCGGCCC
TAGTTGAAGCCTCGGGAGGCGCTC

TCCTGGCTACTGACCCCCCCGTGTGGGTTCCCTGGCGTTTGCTGAAAGCCTTCAAATGCCTAAAGA
ACGACGGTCCCGAAGACGCCCACA

ACCGATCGTCAGATGGGTAAGTCTCACTCTCACTCTCCTCGCTCTCTGTCGGCCCATCCAGACTTG
GAGATGCTCCCTGTCCCTAGGAAA

CCAACAATGGATGACAGCATATAACCAAGAGGCAAAATTTTCCATCTCCATTGACCAAATACTAGAG
GCTCATAATCAGTCACCTTTCTG

TGCCAAGTCTCCCAGATACACCTTGGACTCTGTAAATGGCTATCCTAAGATCTACTGGCCCCCCCC
ACAAGGGCGGCGCCGGTTTGGAGC

CAGGGCCATGGTCACATATGATTGCGAGCCCCGATGCCCTTATGTGGGGGCAGATCGCTTCGACT
GCCCCCACTGGGACAATGCCTCCCA

GGCCGATCAAGGGTCCTTTTATGTCAATCATCAGATTTTATTCCTGCATCTCAAACAATGTCATGGA
ATTTTCACTCTAACCTGGGAGAT

ATGGGGATATGATCCCCTGATCACCTTTTCTTTACATAGGATCCCTGATCCCCCTCAACCCGACTTT
CCCCGGTTGAGCAGTGACTGGGT

TCCCTCTGTCAGATCATGGGCCCTGCTTTTAAATCAAACAGCACGGGCCTTCCCAGACTGTGCTAT
ATGTTGGGAACCTTCCCCTCCCTG

GGCTCCCGAAATATTAGTATATAACAAAACCATCTCCAGCTCTGGACCCGGCCTCGCCCTCCCGGA
CGCCCAAATCTTCTGGGTCAACAC

GTCCTCGTTTAACACCACCCAAGGATGGCACCACCCTTCCCAGAGGTTGTTGTTCAATGTTTCTCAA
GGCAACGCCTTGTTATTACCTCC

TATCTCCCTGGTTAATCTCTCTACGGCTTCCTCCGCCCCTCCTACCCGGGTCAGACGTAGTCCCGT
CGCAGCCCTGACCTTAGGCCTAGC

CCTGTCAGTGGGGCTCACTGGAATTAATGTGGCCGTGTCTGCCCTTAGCCATCAGAGACTCACCTC
CCTGATCCACGTTCTGGAGCAAGA

TCAGCAACGCTTGATCACAGCAATTAACCAGACCCACTATAATTTGCTTAATGTGGCCTCTGTGGTT
GCCCAGAACCGACGGGGGCTTGA

TTGGTTGTACATCCGGCTGGGTTTTCAAAGCCTATGTCCCACAATTAATGAGCCTTGCTGTTTCCTG
CGCATTCAAAATGACTCCATTAT

CCGCCTTGGTGATCTCCAGCCTCTCTCGCAAAGAGTCTCTACAGACTGGCAGTGGCCCTGGAATTG
GGATCTGGGGCTCACTGCCTGGGT

GCGAGAAACCATTCATTCTGTTCTAAGCCTGTTCCTATTAGCCCTTTTTTTGCTCTTCCTGGCCCCC
TGCCTGATAAAATGCTTGACCTC

TCGCCTTTTAAAGCTCCTCCGGCAGGCTCCCCACTTCCCTGAAATCTCCTTAACCCCTAAACCCGAT
TCTGATTATCAGGCCTTGCTACC

ATCTGCACCAGAGATCTACTCTCACCTCTCCCCCGTCAAACCCGATTACATCAACCTCCGACCCTG
CCCTTGATACCCCCGCGTTTCACG

CACCCCCAGGCTGTGGTGGTGCACTGGCTTAGTGGAATAGTCAGTGTACCATCACAAGCCTCTTCT
TGCTGCCAGCACCGAGTTCGAACA

CAGCTCTACCCTGAGCCTCTCTGAGTGCATGACTGAGTGTAGCGCAGAGAGATTGTCGCTTCTGCG
TGTCGCTCAGTCATTTTTTATAGC

CGATTGGGGTTCGCGCCCTTCCGTTGCCTGTGACACAGATAAGACCTCTCTCACTTCTGCTTCACC
ATCCCCCTGCCAGCGTTGGTCTAG

TGGAAAGAACTAACGCTGACGGGGGCGATTTCTTGCAGCTGTGCTAGCGGGAGGCTCTGGTGCTG
GGGATAAGATGTGGCCCTTAGCACC

ACAGTCTCTGCGCCTTTTGGGTTCGAATCTTCCCCACGCAGCTTCCGCTTTTTACGCCCTGTTGCA
CACCCTTTCTAGAGATACCTGAAA

ATCTCAGCTCGCACCCTGAGGAAGGTTGTGGCTCAGAGGTTAAAATAGCTCGAGCCGCAACCTCC
CTTTCTTTTTATTCCACCCTCGCAA

GGCCCCGGGTTCTGAGCCCCCTAACGGAGGTTCAAAATTTCCTCTACTAGGGGATGCTCGGGTCC
AAGTGTGCACAATATCTCTTCCAAA

AGGTCCTGATGAACGTCTTCCCATGTAACAAGCCCCAGCAGAGACATTCCAGCCACATCCAGCAGC
ATTTGGGCCGCCTTTTCTAACAGT

GCCCATAAAGTCCCTTCCGTTTCCACAACGGCTGCCTCTGCATCTTCTATTTCCACCTCGGCACCG
ACTCCCCCGCCGAGCCCTTCGAGC

TCTTCGGGATCCATTACCTGATAACGACAAAATTATTTCTTGTCTTTTAAGCAAGTGTTGTTGGTTGG
GGGCCCCACTCTCTACATGCCT

GCCCGGCCCTGGTTTTGTCCAATGATGTCACCATCGATGCCTGGTGCCCCCTCTGCGGGCCCCAT
GAGCGACTCCAATTCGAAAGGATCG

ACACCACGCTCACCTGCGAGACCCACCGTATCAACTGGACCGCCGATGGACGACCTTGCGGCCTC
AATGGAACGTTGTTCCCTCGACTGC

ATGTCTCCGAGACCCGCCCCCAAGGGCCCCGACGACTCTGGATCAACTGCCCCCTTCCGGCCGTT
CGCGCTCAGCCCGGCCCGGTTTCAC

TTTCCCCCTTCGAGCGGTCCCCCTTCCAGCCCTACCAATGCCAATTGCCCTCGGCCTCTAGTGACG
GTTGCCCCATTATCGGGCACGGCC

TTCTTCCCTGGAACAACTTAGTAACGCATCCTGTCCTCGGAAAAGTCCTTATATTAAATCAAATGGC
CAATTTTTCCTTACTCCCCTCCT

TCGATACCCTCCTTGTGGACCCCCTCCGGCTGTCCGTCTTTGCCCCAGACACCAGGGGAGCCATA
CGTTATCTCTCCACCCTTTTGACGC

TATGCCCAGCTACTTGTATTCTACCCCTAGGCGAGCCCTTCTCTCCTAAGGTCCCCATATGCCGCTT
TCCCCGGGACTCCAATGAACCCC

CCCTTTCAGAATTCGAGCTGCCCCTTATCCAAACGCCCGGCCTGTCTTGGTCTGTCCCCGCGATCG
ACCTATTCCTAACCGGTCCCCCTT

CCCCATGCGACCGGTTACACGTATGGTCCAGTCCTCAGGCCTTACAGCGCTTCCTTCATGACCCTA
CGCTAACCTGGTCCGAATTGGTTG

CTAGCAGAAAAATAAGACTTGATTCCCCCTTAAAATTACAACTGCTAGAAAATGAATGGCTCTCCCG
CCTTTTTTGAGGGGGAGTCATTT

GTATGAAAGATCATGCCGACCTAGGCGCCGCCACCGCCCCGTAAACCAGACAGAGACGTCAGCTG
CCAGAAAAGCTGGTGACGGCAGCTG

GTGGCTAGAATCCCCGTACCTCCCCAACTTCCCCTTTCCCGAAAAATCCACACCCTGAGCTGCTGA
CCTCACCTGCTGATAAATTAATAA

AATGCCGGCCCTGTCGAGCTAGCGGCACCAGAAGCGTTCTTCTCCTGAGACCCTCGTGCTCAGCT
CTCGGTCCTGAGCTCTCTTGCTCCC

GAGACCTTCTGGTCGGCTATCCGGCAGCGGTCAGGTAAGGCAAACCACGGTTTGGAGGGTGGTTC
TCGGCTGAGACCACCGCGAGCTCTA

TCTCCGGTCCTCTGACCGTCTCCACGTGGACTCTCTCCTTTGCCTCCTGACCCCGCGCTCCAAGG
GCGTCTGGCTTGCACCCGCGTTTGT

TTCCTGTCTTACTTTCTGTTTCTCGCGGCCCGCGCTCTCTCCTTCGGCGCCCTCTAGCGGCCAGGA
GAGACCGGCAAACA

>HIV_U1_CHRX 9656 bp

TGGAAGGGCTAATTTGGTCCCAAAGAAGACAAGATATCCTTGATCTGTGGATCTACCACACACAAG
GCTACTTCCCTGATTGGCAGAACT

ACACACCAGGGCCAGGGATCAGATATCCACTGACCTTTGGATGGTGCTTCAAGTTAGTACCAGTTG
AGCCAGGGCAGGTAGAAGAGGCCA

ATGAAGGAGAGAACAACACCTTGTTACACCCTATGAGCCTGCATGGGATGGAGGACCCGGAGGGA
GAAGTATTAGTGTGGAAGTTTGACA

GCCTCCTAGCATTTCGTCACATGGCCCGAGAGCTGCATCCGGAGTACTACAAAGACTGCTGACATC
GAGTTTTCTACAAGGGACTTTCCG

CTGGGGACTTTCCAGGGAGGTGTGGCCTGGGCGGGACTGGGGAGTGGCGAGCCCTCAGATGCTG
CATATAAGCAGCTGCTTTTTGCCTGT

ACTGGGTCTCTCTGGTTAGACCAGATCTGAGCCTGGGAGCTCTCTGGCTAGCTAGGGAACCCACT
GCTTAAGCCTCAATAAAGCTTGCCT

TGAGTGCTACAAGTAGTGTGTGCCCGTCTGTTGTGTGACTCTGGTAACTAGAGATCCCTCAGACCC
TTTTAGTCAGTGTGGAAAATCTCT

AGCAGTGGCGCCCGAACAGGGACTTGAAAGCGAAAGTAAAGCCAGAGGAGATCTCTCGACGCAG
GACTCGGCTTGCTGAAGCGCGCACGG

CAAGAGGCGAGGGGCGGCGACTGGTGAGTACGCCAAAAATTTTGACTAGCGGAGGCTAGAAGGA
GAGAGATGGGTGCGAGAGCGTCGGTA

TTAAGCGGGGGAGAATTAGATAAATGGGAAAAAATTCGGTTAAGGCCAGGGGGAAAGAAACAATAT
AGACTAAAACATATAGTATGGGCA

AGCAGGGAACTAGAACGATTCGCAGTTAATCCTGGCCTTTTAGAGACATCAGAAGGCTGTAGACAA
ATACTGAGACAGCTACAACCATCC

CTTCAGACAGGATCAGAAGAACGTAGATCATTATTTAATACAGTAGCAGTCCTCTATTGTGTGCATC
AAAGGATAGATGTAAAAGACACC

AAGGAAGCCTTAGATAAGATAGAGGAAGAGCAAAACAAAAGTAAGAAAAAGGCACAGCAAGCAGCA
GCTGACACAGGAAACAGCAGCCAG

GTCAGCCAAAATTACCCTATAGTGCAGAACCTCCAGGGGCAAATGGTACATCAGGCCATATCACCT
AGAACTTTAAATGCATGGGTAAAA

GTAGTAGAAGAGAAGGCTTTCAGCCCAGAAGTAATACCCATGTTTTCAGCATTATCAGAAGGAGCC
ACCCCACAAGATTTAAATACCATG

CTAAACACAGTGGGGGGACATCAAGCAGCCATGCAAATGTTAAAAGAGACCATCAATGAGGAAGCT
GCAGAATGGGATAGATTGCATCCA

GTGCATGCAGGGCCTATTGCACCAGGCCAGATGAGAGAACCAAGGGGAAGTGACATAGCAGGAAC
TACTAGTACCCTTCAGGAACAAATA

GGATGGATGACACATAATCCACCTATCCCAGTAGGAGAAATCTATAAAAGATGGATAATCCTGGGAT
TAAATAAAATAGTAAGAATGTAT

AGCCCTACCAGCATTCTGGACATAAGACAAGGACCAAAGGAACCCTTTAGAGACTATGTAGACCGA
TTCTATAAAACTCTAAGAGCCGAG

CAAGCTTCACAAGAGGTAAAAAATTGGATGACAGAAACCTTGTTGGTCCAAAATGCGAACCCAGATT
GTAAGACTATTTTAAAAGCATTG

GGACCAGGAGCGACACTAGAAGAAATGATGACAGCATGTCAGGGAGTGGGGGGACCCGGCCATA
AAGCAAGAGTTTTGGCTGAAGCAATG

AGCCAAGTAACAAATCCAGCTAACATAATGATACAGAAAGGCAATTTTAGGAACCAAAGAAAGACTG
TTAAGTGTTTCAATTGTGGCAAA

GAAGGGCACATAGCCAAAAATTGCAGGGCCCCTAGGAAAAAGGGCTGTTGGAAATGTGGAAAGGA
AGGACACCAAATGAAAGATTGTACT

GAGAGACAGGCTAATTTTTTAGGGAAGATCTGGCCTTCCCACAAGGGAAGGCCAGGGAATTTTCTT
CAGAGCAGACCAGAGCCAACAGCC

CCACCAGAAGAGAGCTTCAGGTTTGGGGAAGAGACAACAACTCCCTCTCAGAAGCAGGAGACGAT
AGACAAGGAACTGTATCCTTTAGCT

TCCCTCAGATCACTCTTTGGCAGCGACCCCTCGTCACAATAAAGATAGGGGGGCAATTAAAGGAAG
CTCTATTAGATACAGGAGCAGATG

ATACAGTATTAGAAGAAATGAATTTGCCAGGAAGATGGAAACCAAAAATGATAGGGGGAATTGGAG
GTTTTATCAAAGTAAGACAGTATG

ATCAGATACTCATAGAAATCTGCGGACATAAAGCTATAGGTACAGTATTAGTAGGACCTACACCTGT
CAACATAATTGGAAGAAATCTGT

TGACTCAGATTGGCTGCACTTTAAATTTTCCCATTAGTCCTATTGAGACTGTACCAGTAAAATTAAAG
CCAGGAATGGATGGCCCAAAAG

TTAAACAATGGCCATTGACAGAAGAAAAAATAAAAGCATTAGTAGAAATTTGTACAGAAATGGAAAA
GGAAGGAAAAATTTCAAAAATTG

GGCCTGAAAATCCATACAATACTCCAGTATTTGCCATAAAGAAAAAAGACAGTACTAAATGGAGAAA
ATTAGTAGATTTCAGAGAACTTA

ATAAGAGAACTCAAGATTTCTGGGAAGTTCAATTAGGAATACCACATCCTGCAGGGTTAAAACAGAA
AAAATCAGTAACAGTACTGGATG

TGGGCGATGCATATTTTTCAGTTCCCTTAGATAAAGACTTCAGGAAGTATACTGCATTTACCATACCT
AGTATAAACAATGAGACACCAG

GGATTAGATATCAGTACAATGTGCTTCCACAGGGATGGAAAGGATCACCAGCAATATTCCAGTGTA
GCATGACAAAAATCTTAGAGCCTT

TTAGAAAACAAAATCCAGACATAGTCATCTATCAATACATGGATGATTTGTATGTAGGATCTGACTTA
GAAATAGGGCAGCATAGAACAA

AAATAGAGGAACTGAGACAACATCTGTTGAGGTGGGGATTTACCACACCAGACAAAAAACATCAGA
AAGAACCTCCATTCCTTTGGATGG

GTTATGAACTCCATCCTGATAAATGGACAGTACAGCCTATAGTGCTGCCAGAAAGGACAGCTGGA
CTGTCAATGACATACAGAAATTAG

TGGGAAAATTGAATTGGGCAAGTCAGATTTATGCAGGGATTAAAGTAAGGCAATTATGTAAACTTCT
TAGGGGAACCAAAGCACTAACAG

AAGTAGTACCACTAACAGAAGAAGCAGAGCTAGAACTGGCAGAAAACAGGGAGATTCTAAAAGAAC
CGGTACATGGAGTGTATTATGACC

CATCAAAAGACTTAATAGCAGAAATACAGAAGCAGGGGCAAGGCCAATGGACATATCAAATTTATCA
AGAGCCATTTAAAAATCTGAAAA

CAGGAAAGTATGCAAGAACGAAGGGTGCCCACACTAATGATGTGAAACAATTAACAGAGGCAGTGC
AAAAAATAGCCACAGAAAGCATAG

TAATATGGGGAAAGACTCCTAAATTTAAATTACCCATACAAAAGGAAACATGGGAAGCATGGTGGAC
AGAGTATTGGCAAGCCACCTGGA

TTCCTGAGTGGGAGTTTGTCAATACCCCTCCCTTAGTGAAGTTATGGTACCAGTTAGAAAAAGAACC
CATAGTAGGAGCAGAAACTTTCT

ATGTAGATGGGGCAGCCAATAGGGAAACTAAATTAGGAAAAGCAGGATATGTAACTGACAGAGGAA
GACAAAAAGTTGTCCCCCTAACGG

ACACAACAAATCAGAAGACTGAGTTACAAGCAATTCATCTAGCTTTGCAGGATTCAGGATTAGAAGT
AAACATAGTAACAGACTCACAAT

ATGCATTGGGAATCATTCAAGCACAACCAGATAAGAGTGAATCAGAGTTAGTCAGTCAAATAATAGA
GCAGTTAATAAAAAAGGAAAAAG

TCTACCTGGCATGGGTACCAGCACACAAAGGAATTGGAGGAAATGAACAAGTAGATAAATTGGTCA
GTGCTGGAATCAGGAAAGTACTAT

TTTTAGATGGAATAGATAAGGCCCAAGAAGAACATGAGAAATATCACAGTAATTGGAGAGCAATGG
CTAGTGATTTTAACCTGCCACCTG

TAGTAGCAAAAGAAATAGTAGCCAGCTGTGATAAATGTCAGCTAAAAGGAGAAGCCATGCATGGAC
AAGTAGACTGTAGCCCCGGAATAT

GGCAGCTAGATTGTACACATTTAGAAGGAAAAGTTATCTTGGTAGCAGTTCATGTGGCCAGTGGAT
ATATAGAAGCAGAAGTAATTCCAG

CAGAGACAGGGCAAGAAACAGCATACTTCCTCTTAAAATTAGCAGGAAGATGGCCAGTAAAAACAG
TACATACAGACAATGGCAGCAATT

TCACCAGTACTACAGTTAAAGCCGCCTGTTGGTGGGCGGGGATCAAGCAGGAATTTGGCATTCCCT
ACAATCCCCAAAGTCAAGGAGTAA

TAGAATCTATGAATAAAGAATTAAAGAAAATTATAGGACAGGTAAGAGATCAGGCTGAACATCTTAA
GACAGCAGTACAAATGGCAGTAT

TCATCCACAATTTTAAAAGAAAAGGGGGGATTGGGGGGTACAGTGCAGGGGAAAGAATAGTAGACA
TAATAGCAACAGACATACAAACTA

AAGAATTACAAAAACAAATTACAAAAATTCAAAATTTTCGGGTTTATTACAGAGACAGCAGAGATCCA
GTTTGGAAAGGACCAGCAAAGC

TCCTCTGGAAAGGTGAAGGGGCAGTAGTAATACAAGATAATAGTGACATAAAAGTAGTGCCAAGAA
GAAAAGCAAAGATCATCAGGGATT

ATGGAAAACAGATGGCAGGTGATGATTGTGTGGCAAGTAGACAGGATGAGGATTAACACATGGAAA
AGATTAGTAAAACACCATATGTAT

ATTTCAAGGAAAGCTAAGGACTGGTTTTATAGACATCACTATGAAAGTACTAATCCAAAAATAAGTTC
AGAAGTACACATCCCACTAGGG

GATGCTAAATTAGTAATAACAACATATTGGGGTCTGCATACAGGAGAAAGAGACTGGCATTTGGGT
CAGGGAGTCTCCATAGAATGGAGG

AAAAAGAGATATAGCACACAAGTAGACCCTGACCTAGCAGACCAACTAATTCATCTGCACTATTTTG
ATTGTTTTTCAGAATCTGCTATA

AGAAATACCATATTAGGACGTTTAGTTAGTCCTAGGTGTGAATATCAAGCAGGACATAACAAGGTAG
GATCTCTACAGTACTTGGCACTA

GCAGCATTAATAAAACCAAAACAGATAAGGCCACCTTTGCCTAGTGTTAGGAAACTGACAGAGGAC
AGATGGAACAAGCCCCAGAAGACC

AAGGGCCACAGAGGGAGCCATACAATGAATGGACACTAGAGCTTTTAGAGGAACTTAAGAGTGAAG
CTGTTAGACATTTTCCTAGGATAT

GGCTCCATAACTTAGGACAACATATCTATGAAACTTATGGGGATACTTGGGCAGGAGTGGAAGCCA
TAATAAGAATTCTGCAACAACTGC

TGTTTATTCATTTCAGTATTGGATGTCAACATAGCAGAATAGGCATTATTCGACAGAGGAGAGCAAG
GAATGGAGCCAGTAGATCCTAGA

CTAGAGCCCTGGAAGCTTCCAGGAAGTCAGCCTAAAACTGCTTGTACCAATTGCTATTGTAAAAAGT
GTTGCTTTCATTGCCAAGTTTGT

TTCACAACAAAAGCCTTAGGCATCTCCTATGGCAGGAAGAAGCGGAGACAGCGACGAAGAGCTCC
TCAAGACAGTGAGACTCATCAAGTT

TCTCTATCAAAGCAGTGAGTAGTACATGTAATGCTTTCTTTCAATAGTTGTGTGGACCATAGTGTTCA
TAGAATACAGGAAAGTATTAAG

ACAAAGAAAAGTAGACAGGATAATTGATAGAATAAGAGAAAGAGCAGAAGACAGTGGCAATGAGAG
CGAAGGGGACCAGGAAGAATTATT

AGCACTTGTGGAGATGGGGCACGATGCTCCTTGGGATGTTAATGATCTGTAGTGCTGCAGAACAAT
TGTGGGTCACAGTCTATTATGGGG

TACCTGTGTGGAAAGAAGCAACCACCACTCTATTTTGTGCCTCGGATGCTAAAGCATATGACACAG
AGGTACATAATGTTTGGGCCACAC

ATGCCTGTGTACCCACAGACCCTAACCCACAAGAAGTAGTATTGCAAAATGTGACAGAAAATTTTAA
CATGTGGAAAAATAACATGGTAG

AACAAATGCATGAGGATATAATCAGTTTATGGGATCAAAGCCTAAAACCATGTGTAAAATTAACCCC
ACTCTGTGTTACCTTAAATTGCA

CTGATTTGACGAATGCTACTTATGCCAATGGTAGTAGTGAGGAGAGAGGAGAAATAAGAAATTGCT
CTTTCAATGTCACCACAATCATAA

GAAATAAGATACAAAAAGAATATGCACTTTTTTATAGACTTGATATAGAACCAATAGATAAGGATAAT
ACAAGCTATACATTGATAAATT

GTAACACCTCAGTCATTACACAGGCTTGTCCAAAGGTATCCTTTGAACCAATTCCCATACATTATTGT
GCCCCGGCTGGTTTTGCGATTC

TAAAGTGTAACGATAAGAAATTCAATGGAACAGGACCATGTACAAATGTCAGTACAGTACAATGTAC
ACATGGAATTAGGCCAGTAGTGT

CAACTCATCTGCTATTAAATGGCAGTCTAGCAGAAGGAGAGGTAGTAATTAGGTCTGAAAATTTCAC GAACAATGTTAAAACCATAATAG

TACAGCTGAATGAATCTGTAGAAATTAATTGTACAAGACCCAACAACAATACAAGAAAAGGTATAGC TATAGGACCGGGGAGAACACTCT

ATGCAAGAGAAAAAATAATAGGAGATATAAGACAAGCACATTGTAACCTTAGTAGAGCAAAATGGAA TGACACTTTAAAACAGATAGTTA

CAAAATTAAAAGAACAATTTAGGAATAAAACAATAGTCTTTAATCAATCCTCAGGAGGGGACCCAGA AATTGTAATGCACAGTTTTAATT

GTGGAGGGGAATTTTTCTACTGTAAGACAACACAGCTGTTTAATAGTACTTGGCTGTTTAATAGTAC TTGGAATGATACTGAAAGGTCAG

ATAACAATGAAACTATCATACTCCCATGCAGAATAAAACAAATTATAAACAGGTGGCAGGAAGTAGG AAAAGCAATATATGCCCCTCCCA

TCAGTGGACAAATTAGATGCTCATCAAATATTACAGGGCTGCTATTAACAAGAGATGGTGGTGATAA AGAGAACAGTACCACCGAGATCT

TCAGACCTGGAGGAGGAGATATGAGGGACAATTGGAGAAGTGAATTATATAAATATAAAGTAGTAAA AATTGAGCCATTAGGAGTAGCAC

CCACCAAGGCAAAGAGAAGAGTGGTGCAGAGAGAAAAAAGAGCAGTGGGAGCGCTAGGAGCTCT GTTTCTTGGGTTCTTGGGAGCAGCAG

GAAGCACTATGGGCGCAGCGTCAATGGCGCTGACGGTACAGACCAGACAATTAATGTCTGGTATA GTGCAACAGCAAAACAATTTGCTGA

AGGCTATTGAGGCGCAACAGCATCTGTTGCAACTCACAGTCTGGGGCATCAAGCAGCTCCAGGCA AGAGTCCTGGCTGTGGAAAGATACC

TAAAGGATCAACAGCTCCTAGGGATGTGGGGTTGCTCTGGAAAACTCATCTGCACCACTACTGTGC CTTGGAATGCTAGTTGGAGTAATA

AATCTCTGGATAAGATTTGGGATAACATGACCTGGATGGAGTGGGAGAGAGAAATTGACAATTACA CAGGCTTAATATACACCTTAATTG

AAGAATCGCAGTACCAACAAGAAAAGAATGAACAAGAATTATTGGAATTGGATAAGTGGGCAAGTTT GTGGAATTGGTTTGACATAACAA

AGTGGCTGTGGTATATAAAAATATTCATAATGATAGTAGGAGGTTTAATAGGTTTAAGAATAGTTTTT ACTGTACTTTCTATAGTGAATA

GAGTTAGGCAGGGATACTCACCATTATCGTTCCAGACCCGCCTCCCAACCCAGAGGGGACCCGAC AGGCCCGAAGGAATCGAAGAAGAAG

GTGGAGAGAGAGACAGAGACAGATCCGGACCATTAGTGAATGGATTCTTAGCACTCATCTGGGTC GATCTGCGGAGCCTGTGCCTCTTCA

GCTACCACCGCTTGAGAGACTTACTCTTGATTGTAACGAGGATTGTGGAACTTCTGGGACGCAGGG GGTGGGAAGTCCTCAAATATTGTT

GGAATCTCCTGCAGTATTGGGGTCAGGAACTAAAGAATAGTGCTATTAGTTTGCTTAATGCCACAGC TGTAGCAGTAGCTGAGGGGACAG

ATAGGGTTATAGAAGTAGCACAAAGAATTGGTAGGGGTATCCTCCATATCCCTAGAAGAATAAGACA AGGCTTAGAAATTGCTTTGCTAT

AAGATGGGTGGCAAGTGGTCAAAACGTAGTATGGCTGGATGGTCTGCTGTAAGAGAAAGAATGAAA CGAGCTGAACCAGCAGCAGATGGG

GTGGGAGCAGTATCTCGAGACTTGGAAAAACATGGAGCAATCACAAGTAGCAATACAGCAGCTACT AATGCTGATTGTGCCTGGCTAGAA

GCACAAGAGGATGAGGAGGTGGGTTTTCCAGTCAGACCTCAGGTACCTTTAAGACCAATAACTCGC AGGGCAGCTATGGATCTTAGCCAC

TTTTTAAAAGAAGAGGGGGGACTGGAAGGGCTAATTTGGTCCCAAAGAAGACAAGATATCCTTGAT CTGTGGATCTACCACACACAAGGC

TACTTCCCTGATTGGCAGAACTACACACCAGGGCCAGGGATCAGATATCCACTGACCTTTGGATGG TGCTTCAAGTTAGTACCAGTTGAG

CCAGGGCAGGTAGAAGAGGCCAATGAAGGAGAGAACAACACCTTGTTACACCCTATGAGCCTGCA TGGGATGGAGGACCCGGAGGGAGAA

GTATTAGTGTGGAAGTTTGACAGCCTCCTAGCATTTCGTCACATGGCCCGAGAGCTGCATCCGGAG TACTACAAAGACTGCTGACATCGA

GTTTTCTACAAGGGACTTTCCGCTGGGGACTTTCCAGGGAGGTGTGGCCTGGGCGGGACTGGGGA GTGGCGAGCCCTCAGATGCTGCATA

TAAGCAGCTGCTTTTTGCCTGTACTGGGTCTCTCTGGTTAGACCAGATCTGAGCCTGGGAGCTCTC TGGCTAGCTAGGGAACCCACTGCT

TAAGCCTCAATAAAGCTTGCCTTGAGTGCTACAAGTAGTGTGTGCCCGTCTGTTGTGTGACTCTGG TAACTAGAGATCCCTCAGACCCTT

TTAGTCAGTGTGGAAAATCTCTAGCA

>HIV_U1_CHR2 9656 bp

TGGAAGGGCTAATTTGGTCCCAAAGAAGACAAGATATCCTTGATCTGTGGATCTACCACACACAAG GCTACTTCCCTGATTGGCAGAACT

ACACACCAGGGCCAGGGATCAGATATCCACTGACCTTTGGATGGTGCTTCAAGTTAGTACCAGTTG AGCCAGGGCAGGTAGAAGAGGCCA

ATGAAGGAGAGAACAACACCTTGTTACACCCTATGAGCCTGCATGGGATGGAGGACCCGGAGGGA GAAGTATTAGTGTGGAAGTTTGACA

GCCTCCTAGCATTTCGTCACATGGCCCGAGAGCTGCATCCGGAGTACTACAAAGACTGCTGACATC GAGTTTTCTACAAGGGACTTTCCG

CTGGGGACTTTCCAGGGAGGTGTGGCCTGGGCGGGACTGGGGAGTGGCGAGCCCTCAGATGCTG CATATAAGCAGCTGCTTTTTGCCTGT

ACTGGGTCTCTCTGGTTAGACCAGATCTGAGCCTGGGAGCTCTCTGGCTAGCTAGGGAACCCACT GCTTAAGCCTCAATAAAGCTTGCCT

TGAGTGCTACAAGTAGTGTGTGCCCGTCTGTTGTGTGACTCTGGTAACTAGAGATCCCTCAGACCC TTTTAGTCAGTGTGGAAAATCTCT

AGCAGTGGCGCCCGAACAGGGACCTGAAAGCGAAAGTAAAGCCAGAGGAGATCTCTCGACGCAG GACTCGGCTTGCTGAAGCGCGCACGG

CAAGAGGCGAGGGGCGGCGACTGGTGAGTACGCCAAAAATTTTGACTAGCGGAGGCTAGAAGGA
GAGAGATGGGTGCGAGAGCGTCGGTA

TTAAGCGGGGGAGAATTAGATAAATGGGAAAAAATTCGGTTAAGGCCAGGGGGAAAGAAACAATAT
AGACTAAAACATATAGTATGGGCA

AGCAGGGAACTAGAACGATTCGCAGTTAATCCTGGCCTTTTAGAGACATCAGAAGGCTGTAGACAA
ATACTGAGACAGCTACAACCATCC

CTTCAGACAGGATCAGAAGAACGTAGATCATTATTTAATACAGTAGCAGTCCTCTATTGTGTGCATC
AAAGGATAGATGTAAAAGACACC

AAGGAAGCCTTAGATAAGATAGAGGAAGAGCAAAACAAAAGTAAGAAAAAGGCACAGCAAGCAGCA
GCTGACACAGGAAACAGCAGCCAG

GTCAGCCAAAATTACCCTATAGTGCAGAACCTCCAGGGGCAAATGGTACATCAGGCCATATCACCT
AGAACTTTAAATGCATGGGTAAAA

GTAGTAGAAGAGAAGACTTTCAGCCCAGAAGTAATACCCATGTTTTCAGCATTATCAGAAGGAGCC
ACCCCACAAGATTTAAATACCATG

CTAAACACAGTGGGGGGACATCAAGCAGCCATGCAAATGTTAAAGGAGACCATCAATGAGGAAGCT
GCAGAATGGGATAGATTGCATCCA

GTGCATGCAGGGCCTATTGCACCAGGCCAGATGAGAGAACCAAGGGGAAGTGACATAGCAGGAAT
TACTAGTACCCTTCAGGAACAAATA

GGATGGATGACACATAATCCACCTATCCCAGTAGGAGAAATCTATAAAAGATGGATAATCCTGGGA
CTAAATAAAATAGTAAGAATGTAT

AGCCCTACCAGCATTCTGGACATAAGACAAGGACCAAAGGAACCCTTTAGAGACTATGTAGACCGG
TTCTATAAAACTCTAAGAGCCGAG

CAAGCTTCACAGGAGGTAAAAAATTGGATGACAGAAACCTTGTTGGTCCAAAATGCGAACCCAGAT
TGTAAGACTATTTTAAAAGCATTG

GGACCAGCAGCTACACTAGAAGAAATGATGACAGCATGTCAGGGAGTGGGGGGACCCGGCCATAA
AGCAAGAGTTTTGGCTGAAGCAATG

AGCCAAGTAACAAATCCAGCTACCATAATGATACAGAAAGGCAATTTTAGGAACCAAAGAAAGACTA
TTAAGTGTTTTAATTGTGGCAAA

GAAGGGCACATAGCCAAAAATTGCAGGGCCCCTAGGAAAAAGGGCTGTTGGAAATGTGGAAAAGA
AGGACACCAAATGAAAGATTGTACT

GAGAGACAGGCTAATTTTTTAGGGAAAATCTGGCCTTCCCACAAGGGAAGGCCAGGGAATTTTCTT
CAGAGCAGACCAGAGCCAACAGCC

CCACCAGAAGAGAGCTTCAGGTTTGGGGAAGAGACAACAACTCCCTCTCAGAAGCAGGAGACGAT
AGACAAGGAACTGTATCCTTTAGCT

TCCCTCAGATCACTCTTTGGCAGCGACCCCTCGTCACAATAAAGATAGGGGGGCAACTAAAGGAAG
CTCTATTAGATACAGGAGCAGATG

ATACAGTATTAGAAGAAATGAATTTGCCAGGAAGATGGAAACCAAAAATGATAGGGGGAATTGGAG
GTTTTATCAAAGTAAGACAGTATG

ATCAGATACCCATAGAAATCTGCGGACATAAAGCTATAGGTACAGTATTAGTAGGACCTACACCTGT
CAACATAATTGGAAGAAATCTGT

TGACTCAGATTGGCTGCACTTTAAATTTTCCCATTAGTCCTATTGAAACTGTACCAGTAAAATTAAAG
CCAGGAATGGATGGCCCAAAAG

TTAAACAATGGCCATTGACAGAAGAAAAAATAAAAGCATTAGTAGAAATTTGTACAGAAATGGAAAA
GGAAGGAAAAATTTCAAAAATTG

GGCCTGAAAATCCATACAATACTCCAGTATTTGCCATAAAGAAAAAAGACAGTACTAAATGGAGAAA
ATTAGTAGATTTCAGAGAACTTA

ATAAGAGAACTCAAGATTTCTGGGAAGTTCAATTAGGAATACCACATCCTGCAGGATTAAAACAGAA
AAAATCAGTAACAGTACTGGATG

TGGGTGATGCATATTTTTCAGTTCCCTTAGATAAAGACTTCAGGAAGTATACTGCATTTACCATACCT
AGTATAAACAATGAGACACCAG

GGATTAGATATCAGTACAATGTGCTTCCACAGGGATGGAAAGGATCACCAGCAATATTCCAGTGTA
GCATGACAAAAATCTTGGAGCCTT

TTAGAAAACAAAATCCAGACATAGTCATCTATCAATACATGGATGATTTGTATGTAGGATCTGACTTA
GAAATAGGGCAGCATAGAACAA

AAATAGAGGAACTGAGACAACATCTGTTGAGGTGGGGATTTACCACACCAGACAAAAAACATCAGA
AAGAACCTCCATTCCTTTGGATGG

GTTATGAACTCCATCCTGATAAATGGACAGTACAGCCTATAGTGCTGCCAGAAAAGGACAGCTGGA
CTGTCAATGACATACAGAAATTAG

TGGGAAAATTGAATTGGGCAAGTCAGATTTATGCAGGGATTAAAGTAAGGCAATTATGTAAACTTCT
TAGGGGAACCAAAGCACTAACAG

AAGTAGTACCACTAACAGAAGAAGCAGAGCTAGAACTGGCAGAAAACAGGGAGATTCTAAAAGAAC
CGGTACATGGAGTGTATTATGACC

CATCAAAAGACTTAATAGCAGAAATACAGAAGCAGGGGCAAGGCCAATGGACATATCAAATTTATCA
AGAGCCATTTAAAAATCTGAAAA

CAGGAAAGTATGCAAGAATGAAGGGTGCCCACACTAATGATGTGAAACAATTAACAGAGGCAGTAC
AAAAAATAGCCACAGAAAGCATAG

TAATATGGGGAAAGACTCCTAAATTTAAATTACCCATACAAAAGGAAACATGGGAAGCGTGGTGGA
CGGAGTATTGGCAAGCCACCTGGA

TTCCTGAATGGGAGTTTGTCAATACCCCTCCCTTAGTGAAGTTATGGTACCAGTTAGAAAAAGAACC
CATAGTAGGAGCAGAAACTTTCT

ATGTAGATGGGGCAGCCAATAGGGAAACTAAATTAGGAAAAGCAGGATATGTAACTGACAGAGGAA
GACAAAAAGTTGTCCCCCTAACGG

ACACAACAAATCAGAAGACTGAGTTACAAGCAATTCATCTAGCTTTGCAGGATTCAGGATTAGAAGT
AAACATAGTAACAGACTCACAAT

ATGCATTGGGAATCATTCAAGCACAACCAGATAAGAGTGAATCAGAGTTAGTCAGTCAAATAATAGA
GCAGTTAATAAAAAAGGAAAAAG

TCTACCTGGCATGGGTACCAGCACACAAAGGAATTGGAGGAAATGAACAAGTAGATAAATTGGTCA
GTGCTGGAATCAGGAAAGTACTAT

TTTTAGATGGAATAGATAAGGCCCAAGAAGAACATGAGAAATATCACAGTAATTGGAGAGCAATGG
CTAGTGATTTTAACCTGCCACCTG

TAGTAGCAAAAGAAATAGTAGCCAGCTGTGATAAATGTCAGCTAAAAGGAGAAGCCATGCATGGAC
AAGTAGACTGTAGCCCCGGAATAT

GGCAGCTAGATTGTACACATTTAGAAGGAAAAGTTATCTTGGTAGCAGTTCATGTAGCCAGTGGATA
TATAGAAGCAGAAGTAATTCCAG

CAGAGACAGGGCAAGAAACAGCATACTTCCTCTTAAAATTAGCAGGAAGATGGCCAGTAAAAACAG
TACATACAGACAATGGCAGCAATT

TCACCAGTACTACAGTTAAAGCCGCCTGTTGGTGGGCGGGGATCAAGCAGGAATTTGGCATTCCCT
ACAATCCCCAAAGTCAAGGAGTAA

TAGAATCTATGAATAAAGAATTAAAGAAAATTATAGGACAGGTAAGAGATCAGGCTGAACATCTTAA
GACAGCAGTACAAATGGCAGTAT

TCATCCACAATTTTAAAAGAAAAGGGGGGATTGGGGGGTACAGTGCAGGGGAAAGAATAGTAGACA
TAATAGCAACAGACATACAAACTA

AAGAATTACAAAAACAAATTACAAAAATTCAAAATTTTCGGGTTTATTACAGGGACAGCAGAGATCCA
GTTTGGAAAGGACCAGCAAAGC

TCCTCTGGAAAGGTGAAGGGGCAGTAGTAATACAAGATAATAGTGACATAAAAGTAGTGCCAAGAA
GAAAAGCAAAGATCATCAGGGATT

ATGGAAAACAGATGGCAGGTGATGATTGTGTGGCAAGTAGACAGGATGAGGATTAACACATGGAAA
AGATTAGTAAAACACCATATGTAT

ATTTCAAGGAAAGCTAAGGACTGGTTTTATAGACATCACTATGAAAGTACTAATCCAAAAATAAGTTC
AGAAGTACACATCCCACTAGGG

GATGCTAAATTAGTAATAACAACATATTGGGGTCTGCATACAGGAGAAAGAGACTGGCATTTGGGT
CAGGGAGTCTCCATAGAATGGAGG

AAAAAGAGATATAGCACACAAGTAGACCCTGACCTAGCAGACCAACTAATTCATCTGCACTATTTTG
ATTGTTTTTCAGAATCTGCTATA

AGAAATACCATATTAGGACGTTTAGTTAGTCCTAGGTGTGAATATCAAGCAGGACATAACAAGGTAG
GATCTCTACAGTACTTGGCACTA

GCAGCATTAATAAAACCAAAACAGATAAGGCCACCTTTGCCTAGTGTTAGGAAACTGACAGAGGAC
AGATGGAACAAGCCCCAGAAGACC

AAGGGCCACAGAGGGAGCCATACAATGAATGGACACTAGAGCTTTTAGAGGAACTTAAGAGTGAAG
CTGTTAGACATTTTCCTAGGATAT

GGCTCCATAACTTAGGACAACATATCTATGAAACTTATGGGGATACTTGGGCAGGAGTGGAAGCCA
TAATAAGAATTCTGCAACAACTGC

TGTTTATTCATTTCAGAATTGGATGTCAACATAGCAGAATAGGCATTATTCGACAGAGGAGAGCAAG
GAACGGAGCCAGTAGATCCTAGA

CTAGAGCCCTGGAAGCATCCAGGAAGTCAGCCTAAAACTGCTTGTACCAATTGCTATTGTAAAAAGT
GTTGCTTTCATTGCCAAGTTTGT

TTCACAACAAAAGCCTTAGGCATCTCCTATGGCAGGAAGAAGCGGAGACAGCGACGAAGAGCTCC
TCAAGACAGTGAGACTCATCAAGTT

TCTCTATCAAAGCAGTGAGTAGTACATGTAATGCTTTCTTTCAATAGTTGTGTGGACCATAGTGTTCA
TAGAATACAGGAAAGTATTAAG

ACAAAGAAAAGTAGACAGGATAATTGATAGAATAAGAGAAAGAGCAGAAGACAGTGGCAATGAGAG
CGAAGGGGACCAGGAAGAATTATC

AGCACTTGTGGAGATGGGGCACGATGCTCCTTGGGATGTTAATGATCTGTAGTGCTGCAGAACAAT
TGTGGGTCACAGTCTATTATGGGG

TACCTGTGTGGAAAGAAGCAACCACCACTCTATTTTGTGCCTCGGATGCTAAAGCATATGACACAG
AGGTACATAATGTTTGGGCCACAC

ATGCCTGTGTACCCACAGACCCTAACCCACAAGAAGTAGTATTGCAAAATGTGACAGAAAATTTTAA
CATGTGGAAAAATAACATGGTAG

AACAAATGCATGAGGATATAATCAGTTTATGGGATCAAAGCCTAAAACCATGTGTAAAATTAACCCC
ACTCTGTGTTACCTTAAATTGCA

CTGATTTGACGAATGCTACTTATGCCAATGGTAGTAGTGAGGAGAGAGGAGAAATAAGAAATTGCT
CTTTCAATGTCACCACAATCATAA

GAAATAAGATACAAAAAGAATATGCACTTTTTTATAGACTTGATATAGAACCAATAGATAAGGATAAT
ACAAGCTATACATTGATAAATT

GTAACACCTCAGTCATTACACAGGCTTGTCCAAAGGTATCCTTTGAACCAATTCCCATACATTATTGT
GCCCCGGCTGGTTTTGCGATTC

TAAAGTGTAACGATAAGAAATTCAATGGAACAGGACCATGTACAAATGTCAGTACAGTACAATGTAC
ACATGGAATTAGGCCAGTAGTGT

CAACTCATCTGCTATTAAATGGCAGTCTAGCAGAAGGAGAGGTAGTAATTAGGTCTGAAAATTTCAC
GAACAATGTTAAAACCATAATAG

TACAGCTGAATGAATCTGTAGAAATTAATTGTACAAGACCCAACAACAATACAAGAAAAGGTATAGC
TATAGGACCGGGGAGAACACTCT

ATGCAAGAGAAAAAATAATAGGAGATATAAGACAAGCACATTGTAACCTTAGTAGAGCAAAATGGAA
TGACACTTTAAAACAGATAGTTA

CAAAATTAAAAGAACAATTTAGGAATAAAACAATAGTCTTTAATCAATCCTCAGGAGGGGACCCAGA
AATTGTAATGCACAGTTTTAATT

GTGGAGGGGAATTTTTCTACTGTAAGACAACACAGCTGTTTAATAGTACTTGGCTGTTTAATAGTAC
TTGGAATGATACTGAAAGGTCAG

ATAACAATGAAACTATCATACTCCCATGCAGAATAAAACAAATTATAAACAGGTGGCAGGAAGTAGG
AAAAGCAATATATGCCCCTCCCA

TCAGTGGACAAATTAGATGCTCATCAAATATTACAGGGCTGCTATTAACAAGAGATGGTGGTGATAA
AGAGAACAGTACCACCGAGATCT

TCAGACCTGGAGGAGGAGATATGAGGGACAATTGGAGAAGTGAATTATATAAATATAAAGTAGTAAA
AATTGAGCCATTAGGAGTAGCAC

CCACCAAGGCAAAGAGAAGAGTGGTGCAGAGAAAAAAAAGAGCAGTGGGAGCGCTAGGAGCTCTG
TTCCTTGGGTTCTTGGGAGCAGCAG

GAAGCACTATGGGCGCAGCGTCAATGGCGCTGACGGTACAGACCAGACAATTAATGTCTGGTATA
GTGCAACAGCAGAACAATTTGCTGA

AGGCTATTGAGGCGCAACAGCATCTGTTGCAACTCACAGTCTGGGGCATCAAGCAGCTCCAGGCA
AGAGTCCTGGCTGTGGAAAGATACC

TAAAGGATCAACAGCTCCTAGGGATTTGGGGTTGCTCTGGAAAACTCATCTGCACCACTACTGTGC
CTTGGAATGCTAGTTGGAGTAATA

AATCTCTGGATAAGATTTGGGATAACATGACCTGGATGGAGTGGGAGAGAGAAATTGACAATTACA
CAGGCTTAATATACACCTTAATTG

AAGAATCGCAGATCCAACAAGAAAAGAATGAACAAGAATTATTGGAATTGGATAAGTGGGCAAGTTT
GTGGAATTGGTTTGACATAACAA

AGTGGCTGTGGTATATAAAAATATTCATAATGATAGTAGGAGGTTTAATAGGTTTAAGAATAGTTTTT
ACTGTACTGTCTATAGTGAATA

GAGTTAGGCAGGGATACTCACCATTATCGTTCCAGACCCGCCTCCCAGCCCAGAGGGGACCCGAC
AGGCCCGAAGGAATCGAAGAAGAAG

GTGGAGAGAGAGACAGAGACAGATCCGGACCATTAGTGAATGGATTCTTAGCACTCATCTGGGTC
GATCTGCGGAGCCTGTGCCTCTTCA

GCTACCACCGCTTGAGAGACTTACTCTTGATTGTAACGAGGATTGTGGAACTTCTGGGACGCAGGG
GGTGGGAAGCCCTCAAATATTGTT

GGAATCTCCTGCAGTATTGGGGTCAGGAACTAAAGAATAGTGCTATTAGTTTGCTTAATGCCACAGC
TGTAGCAGTAGCTGAGGGGACAG

ATAGGGTTATAGAAGTAGCACAAAGAATTGGTAGGGGTATCCTCCATATCCCTAGAAGAATAAGACA
AGGCTTAGAAATTGCTTTGCTAT

AAGATGGGTGGCAAGTGGTCAAAACGTAGTATGGCTGGATGGTCTGCTGTAAGAGAAAGAATGAAA
CGAGCTGAACCAGCAGCAGATGGG

GTGGGAGCAGTATCTCGAGACTTGGAAAAACATGGAGCAATCACAAGTAGCAATACAGCAGCTACT
AATGCTAATTGTGCCTGGCTAGAA

GCACACGAGGAGGAGGAGGTGGGTTTTCCAGTCAGACCTCAGGTACCTTTAAGACCAATAACTCG
CAAGGCAGCTATGGATCTTAGCCAC

TTTTTAAAAGAAGAGGGGGGACTGGAAGGGCTAATTTGGTCCCAAAGAAGACAAGATATCCTTGAT
CTGTGGATCTACCACACACAAGGC

TACTTCCCTGATTGGCAGAACTACACACCAGGGCCAGGGATCAGATATCCACTGACCTTTGGATGG
TGCTTCAAGTTAGTACCAGTTGAG

CCAGGGCAGGTAGAAGAGGCCAATGAAGGAGAGAACAACACCTTGTTACACCCTATGAGCCTGCA
TGGGATGGAGGACCCGGAGGGAGAA

GTATTAGTGTGGAAGTTTGACAGCCTCCTAGCATTTCGTCACATGGCCCGAGAGCTGCATCCGGAG
TACTACAAAGACTGCTGACATCGA

GTTTTCTACAAGGGACTTTCCGCTGGGGACTTTCCAGGGAGGTGTGGCCTGGGCGGGACTGGGGA
GTGGCGAGCCCTCAGATGCTGCATA

TAAGCAGCTGCTTTTTGCCTGTACTGGGTCTCTCTGGTTAGACCAGATCTGAGCCTGGGAGCTCTC
TGGCTAGCTAGGGAACCCACTGCT

TAAGCCTCAATAAAGCTTGCCTTGAGTGCTACAAGTAGTGTGTGCCCGTCTGTTGTGTGACTCTGG
TAACTAGAGATCCCTCAGACCCTT

TTAGTCAGTGTGGAAAATCTCTAGCA

>02006_consensus (9737 bp) Consensus sequence of the proviruses from patient 02006

TGGAAGGGATGATTTACTCCCAGAAAAGACAAGAAATCCTTGACCTGTGGGTCTACCACACACAAG
GCTACTTCCCTGATTGGCAGAACT

ACACACCAGGGCCAGGGATCAGATATCCACTGACCTTTGGGTGGTGCTTCAAGTTAGTACCAGTGG
ATCCAGATAAGGTAGAAGAGGCCA

CTGAAGGAGAAAACAACTGCTTGTTACACCCTATGAACCAGCACGGGATGGATGATCCGGAGAAG
GAAGTGTTACAGTGGAAGTTTGACA

GTCGCCTAGCATTTCACCATGTGGCCCGAGAGATACATCCGGAGTACTACAAGAACTGATGACACT
GAGCTTTCTACAAGGGACTTTCCA

CTGGGGACTTTCCAGGGGAGGTGTGGCCGGGGCGGGACTGGGGAGTGGCGAGCCCTCAGATGC
TGCATATAAGCAGCTGCTTTTTGCCTG

TACTGGGTCTCTCTGGTTAGACCAGATCTGAGCCTGGGAGCTCTCTGGCTGACTAGGGAACCCACT
GCTTAAGCCTCAATAAAGCTTGCC

TTGAGTGCTTCAAGTAGTGTGTGCCCGTCTGTTGTGTGACTCTGGTAACTAGAGATCCCTCAGACC
CATTTAGTCAGTGTGGAAAATCTC

TAGCAGTGGCGCCCGAACAGGGACTTGAAAGCGAAAGTAAAGCCAGAGGAGATCTCTCGACGCAG
GACTCGGCTTGCTGAAGCGCGCACA

GCAAGAGGCGAGGGGCGGCGACTGGTGAGTACGCCAAAAATTTTGACTAGCGGAGGCTAGAAGG
AGAGAGATGGGTGCGAGAGCGTCAAT

ATTAAGCGGGGGAAAATTAGATGCATGGGAAAAAATTCGGTTAAGGCCAGGAGGAAAGAAACAATA
TAAGCTAAAACATCTAGTATGGGC

AAGCAGGGAGTTAGAACGATTCGCGGTTAACCCTGGCCTGTTAGAAACATCAGAAGGCTGTAAACA
AATAATAGGACAGCTACAACCATC

CTTACAGACAGGATCAGAAGAACTTAAATCATTATTTAATACAGTAGCAGTACTCTATTGTGTACACC
AAAGGATAGAGGTAAAAGACAC

CAAGGAAGCTCTAGATAAGATAGAGGAAGAGCAAAATAAAAGTAAGAAAAAGGAACAGCAAGCAGC
AGCTGACGCAGGAAGCAGCAGCAA

GGTCAGCCAAAATTACCCTATAGTGCAGAACCTACAGGGACAAATGGTACATCAGCCTATATCACC
TAGAACTTTAAATGCATGGGTAAA

AGTAGTAGAAGAGAAGGCTTTCAGCCCAGAAGTAATACCCATGTTTACAGCATTATCAGAAGGAGC
CACCCCACAAGATTTAAACACCAT

GCTAAACACGGTGGGGGGACACCAAGCAGCCATGCAAATGTTAAAAGAGACCATCAATGAGGAAG
CTGCAGAATGGGATAGATTGCATCC

AGTGCAGGCAGGGCCTGTTGCACCAGGCCAAATAAGAGAACCAAGGGGAAGTGACATAGCAGGAA
CTACTAGTACCCTTCAGGAACAAAT

AGCATGGATGACACATAATCCACCTGTCCCAGTAGGAGAAATCTATAAAAGATGGATAATCCTGGG
ATTAAATAAGATAGTAAGAATGTA

TAGCCCTACCAGCATTCTGGACATAAGACAAGGACCAAAGGAACCCTTTAGAGACTATGTAGACCG
GTTCTATAAGACTCTAAGAGCTGA

GCAAGCTTCACAGGATGTAAAAAATTGGATGACAGAAACCTTGTTGGTCCAAAATTCAAACCCAGAT
TGTAAGACTATTTTAAAAGCATT

GGGACCAGGAGCTACACTAGAAGAAATGATGACAGCATGTCAGGGAGTGGGGGGACCCGGCCAC
AAAGCAAGAGTTTTGGCGGAAGCAAT

GAGCCAAGCAACAGGTGCAGCTACCATAATGATGCAGAAAGGCAATTTTAGGAACCAAAGAAAAGC
TGTTAAGTGTTTCAATTGTGGCAA

AGAAGGGCACATAGCCAGAAATTGCAGGGCCCCTAGGAAAAAGGGCTGTTGGAAATGTGGAAAGG
AAGGACACCAAATGAAAGATTGTAT

TGAGAGACAGGCTAATTTTTTAGGGAAAATCTGGCCTTCCCACAAGGGAAGGCCAGGGAATTTTCT
TCAGAGCAGACCAGAGCCAACAGC

CCCACCAGCCCCACCAGAAGAGAGCTTCAGGTTTGGGGAAGAGACAACAACTCCCCATCAGAAGC
CGGAGGCGATAGACAAGGAAGTGTA

TCCTTTGGCTTCCCTCAAATCACTCTTTGGCAGCGACCCCTCGTCACAATAAGGATAGGGGGGCAG
CTAAAGGAAGCTCTATTAGATACA

GGAGCAGATGACACAGTATTAGAAGAAATGAATTTGCCAGGAAAATGGAAACCAAAAATGATAGGG
GGAATTGGAGGTTTTATCAAAGTA

AGACAGTATGATCAGATACCCATAGAAATATGTGGACATAAAGCTATAGGTACAGTATTAGTAGGAC
CTACACCTGTCAACATAATTGGA

AGAAATCTGTTGACTCAGCTTGGCTGTACTTTAAATTTTCCCATTAGTCCTATTGAAACTGTACCAGT
AAAATTAAAGCCAGGAATGGAT

GGCCCAAAAGTTAAACAATGGCCATTGACAGAAGAAAAAATAAAAGCATTAGTAGAAATTTGTACAG
AAATGGAAAAGGAAGGGAAAATT

TCAAAAATTGGGCCTGAAAATCCATACAACACTCCAGTATTTGCCATAAAGAAAAAAGATAGTACTA
AATGGAGAAAATTAGTAGATTTC

AGAGAACTTAATAAGAGAACTCAAGACTTCTGGGAAGTTCAATTAGGAATACCACATCCTGCAGGGT
TAAAAAAGAAAAAATCAGTAACA

GTACTAGATGTGGGTGATGCATATTTTTCAGTTCCCTTAGATAAAGACTTCAGGAAGTATACTGCAT
TTACCATACCTAGTACAAACAAT

GAGACACCAGGGATTAGATATCAGTACAATGTGCTTCCACAGGGATGGAAAGGATCACCAGCAATA
TTCCAATGTAGCATGACAAAAATT

TTAGAGCCTTTTAGAAAACAACATCCAGATATAGTTATCTATCAATACATGGATGATTTGTATGTAGG
ATCTGACTTAGAAATAGGACAG

CATAGAACAAAAATAGAAGAACTGAGACAACACTTGTTGAAGTGGGGATTTACTACACCAGACAAAA
AACATCAGAAAGAACCTCCATTC

CTTTGGATGGGTTATGAACTCCATCCTGATAAATGGACAGTACAGCCTATAGTGCTGCCAGAAAAA
GACAGCTGGACTGTCAATGACATA

CAGAAGTTAGTGGGAAAATTGAATTGGGCAAGTCAGATTTACCCAGGGATTAAAGTAAGGCAATTAT
GTAAACTCCTTAGAGGAACCAAA

GCACTAACAGAAGTAATAACTCTAACAGCAGAAGCAGAGTTAGAACTGGCAGAAAACAGGGAAATT
CTAAAAGAACCAGTACATGGAGTG

TATTATGACCCATCAAAAGACTTAATAGCAGAAATACAGAAGCAGGAGCAAGGCCAATGGACATATC
AAATTTATCAAGAACCATTTAAA

AACCTGAAAACAGGAAAATATGCAAGAGTGAGGGGTGCCCACACTAATGATGTAAGACAATTAACA
GAGGCAGTGCAAAAAATAGCCACA

GAAAGCATAGTAATATGGGGAAAGACTCCTAAATTTAAACTACCCATACAAAAAGAAACATGGGAAG
CATGGTGGACAGAGTATTGGCAA

GCCACCTGGATTCCTGAATGGGAGTTTGTCAATACCCCTCCCCTAGTGAAGTTATGGTACCAGTTA
GAAAAAGAACCCATAGTAGGAGCA

GAAACTTTCTATGTAGATGGGGCAGCTAACAGAGAAACTAAATTAGGAAAAGCAGGATATGTTACTG
ACAGAGGAAGACAAAAGGTTGTC

CCCCTAACTGACACAACAAATCAGAAGACTGAGTTACAAGCAATTCAGCTAGCTTTGCAAGATTCGG
GATTAGAAGTAAACATAGTAACA

GACTCACAATATGCATTAGGAATCATTCAAGCACAACCAGATAAGAGCGAATCAGAGGTAGTCAGT
CAAATAATAGAGCAGTTAATAAAA

AAGGAAAAGGTCTACCTGGCCTGGGTGCCAGCACACAAAGGGATAGGAGGAAATGAACAGGTAGA
TAAATTAGTCAGTTCTGGAATCAGG

AAAATACTATTTTTAGATGGAATAGATAAGGCCCAAGAAGACCATGAGAAATATCACAGTAATTGGA
GAGCAATGGCTAGTGATTTTAAC

CTGCCACCTGTAATAGCAAAAGAAATAATAGCCAGCTGTGATAAATGTCAGCTAAAAGGAGAAGCC
ATGCATGGACAAGTAGACTGTAGT

CCAGGAATATGGCAACTAGATTGTACACACTTAGAAGGAAAAATTATCCTGGTAGCAGTTCATGTAG
CCAGTGGATACATAGAAGCAGAA

GTTATTCCAGCAGAGACAGGGCAAGAAACAGCATACTTTATCTTAAAATTAGCAGGAAGGTGGCCA
GTAAAAACAATACATACAGACAAT

GGCAGCAATTTCACCAGTACTACAGTTAAGGCCGCCTGCTGGTGGGCGGGGATCAAGCAGGAATT
TGGCATTCCCTACAATCCCCAAAGT

CAAGGAGTAGTAGAATCTATGAATAAAGAATTAAAGAAAATTATAGGGCAAGTAAGAGATCAGGCTG
AACATCTTAAGACAGCAGTACAA

ATGGCAGTATTCATCCACAATTTTAAAAGAAAAGGGGGGATTGGGGGGTACAGTGCAGGGGAAAG
AATAGTAGACATAATAGCAACAGAC

ATACAAACTAAAGAATTACAAAACCAAATTACAAAAATTCAAAAATTTCGGGTTTATTACAGGGACAG
CAGAGATCCACTTTGGAAAGGA

CCAGCAAAACTTCTCTGGAAAGGTGAAGGGGCAGTGGTAATACAAGATAATAGTGACATAAAAGTA
GTGCCAAGGAGAAAAGCAAAGATC

ATTAGGGATTATGGAAAACAGATGGCAGGTGATGATTGTGTGGCAAGTAGACAGGATGAGGATTAG
AACATGGAACAGTTTAGTAAAACA

CCATATGTATATTTCAAAGAAAGCTAGGGGATGGTCTTATAAACATCACTATGAAAACCCCCATCCA
AGAGTAGGTTCAGAAGTACATAT

CCCACTAGGGGATGCTAAATTGGTAGTAACAACATATTGGGGTCTGCATACAGGAGAAAGAGACTG
GCATATGGGTCAGGGAGTCTCCAT

AGAATGGAGGAAAAAGGAATATAGCACACAAGTAGACCCTAACACAGCAGACCGACTAATCCATCT
GTATTACTTTGATTGTTTTTCAGA

ATCTGCTATAAGAAAGGCCATATTAGGACATAGAGTTAGTCCTAGTTGTGAATATCAAGCAGGACAT
AACAAGGTAGGATCTCTACAGTA

CCTGGCACTAGTAGCATTAGTAAAACAAAAAAGGACAAAGCCACCTTTGCCTAGTATTACAAAGCTG
ACAGAGGATAGATGGAACAAGCC

CCAGAAGACCAAGGGCCACAGAGGGAGCCATACCATGAATGGACACTAGAGCTTTTAGAGGAGCT
TAAGCATGAAGCTGTCAGACATTTT

CCTAGGCCATGGCTCCATGGCTTAGGGCAACACATCTATGAAACTTATGGGGATACTTGGGCAGGA
GTGGAGGCCATAATAAGAATATTG

CAACAACTACTGTTTATTCATTTCAGAATTGGGTGTCGACATAGCAGAATAGGCATTACTCGACAGA
GGAGAGCAAGAAATGGAGCCAGT

AGATCCTAGACTAGAGCCCTGGAAGCATCCAGGAAGTCAGCCTAAAACTGCTTGTACCAATTGCTA
TTGTAAAAAGTGTTGCTTTCATTG

CCAAGTCTGTTTCACAAAAAAAGCCTTAGGCATCTCCTATGGCAGGAAGAAGCGGAGACAGCGACG
AAGAGCTCCTCAAGGCAATCAGAA

TAATCAAGTTCCTCTACCAGAACAGTAAGTAATATATGTAATGCAATCTGAAGTAATATTAGCAATAG
TAGGATTAGTAGTAGCAGCAAT

AATAGCAATAGTTGTGTGGACCATAGTATTCATAGAATATAGGAAAATATTAAGACAAAGAAAAATAG
ACAGGTTAATTGATAGAATAAG

AGATAGAGCAGAAGACAGTGGCAATGAGAGTGAAGGGGACCAGGAAGAATTGTCAGCACTTGTGG
AGATGGGGCATCATGCTCCTTGGGA

TGTTGATGATCTGTAGTGCTGCAGACAATTTGTGGGTCACAGTCTATTATGGGGTACCTGTGTGGA
AAGAAGCAACCACCACTCTATTTT

GTGCATCAGATGCTAAAGCATATGATGCAGAAGTACATAATGTTTGGGCTACACATGCCTGTGTACC
CACAGACCCCAGCCCACAAGAAA

TAAAATTGGAAAATGTGACAGAAAATTTTAATATGTGGAAAAATGGCATGGTAGAACAGATGCATGA
GGATATAATCAGTTTATGGGATC

AAAGCCTAAAGCCATGTGTAAAATTAACCCCACTCTGTGTTACTTTACATTGCCAAAAAGTAACGATA
GGCAATACCACTTATGTTAATA

ATAGTACAGAAGAAGAAATGAGAAACTGCTCTTTCAATACCACCACAAACATAAGAGATAAGATGCA
AAAAGAATATGCACTTTTTTATA

GTCTTGATTTAGTATCACTAGATAATAATAAGCATAATAATAGTAGTAATAGTACCAGATATAGATTG
ACAAGTTGTAATACCTCAGTTA

TCACACAGGCCTGCCCAAAGGTATCCTTTGAACCAATTCCCATACATTATTGTGCCCCGGCTGGTTT
TGCGCTTTTAAAGTGTAATGATA

AAAAGTTTAATGGAACAGGGCCATGCTCAAATGTCAGCACAGTACAATGTACACATGGAATTAAGCC
AGTAGTATCAACTCAATTGCTGT

TAAATGGCAGTTTAGCAGAAGAAGAGGTAGTAATTAGATCTAAAAATTTCACTGACAATGCTAAGAC
CATAATAGTACAGCTGAATAAAC

CTGTAGAAATTAACTGTACAAGACCCAACAACAATACAAGTAAAAGTATAACTATAGGACCAGGGAG
AGCATTTTATGCAACAGAAAGAA

TAATAGGAAATATAAGACAAGCACATTGTAACATTAATGGATCAAAATGGAATAACACTTTAAGACAG
ATAGTTACAAAATTAAGAGAAC

AATTTGGGAATAAAACAATAGTCTTTAATCAATCCTCAGGAGGGGACCCAGAAATAGTAATGCACAT
CTTTAATTGTAGAGGGGAATTTT

TCTACTGTAATACAGCAAAGCTGTTTAATAGTACTTGGCTGTTTAATAGTACTGAAAAGCCAAATAAC
ACCATCAATGGCACCATCACAC

TCCCATGCAGAATAAAACAAATTATAAACATGTGGCAGGAAGTAGGAAAAGCAATGTATGCCCCTCC
CATCCAAGGAAACATTAGTTGTT

CATCAAATATTACAGGGCTGTTATTAACAAGAGATGGTGGTAATAACAATAATGAGAGCACCCCAGA
GATCTTCAGACCTGGAGGAGGAA

ACATGAAGGACAATTGGAGAAGTGAATTATATAAATATAAAGTAGTAAAAATTGAACCATTAGGATTA
GCACCCACCAGGGCAAAGAGAA

GAGTGGTGCAGAGAGAAAAAAGAGCAGTAACACTAGGAGCTGTGTTCCTTGGGTTCTTGGGAGCA
GCAGGAAGCACTATGGGCGCAGCGT

CACTGACGCTGACGGTACAGGCCAGACAATTATTGTCTGGTATAGTGCAACAGCAGAACAACCTGC
TGAGGGCTATTGATGCGCAACAGC

ATCTGTTGCAGCTCACAGTCTGGGGCATCAAGCAGCTCCAGGCAAGAGTCCTGGCTGTAGAAAGA
TACCTACGGGATCAACAGCTCCTAG

GGATTTGGGGTTGCTCTGGAAAACTCATTTGCACCACTACTGTGCCTTGGAATACTAGTTGGAGTAA
TAAATCTCTGGATCACATTTGGG

GTAACATGACCTGGATGGAGTGGGAAAGAGAAATTGACAATTACACAAACTTAATATACAACTTAAT
TGAAGAATCACAGAACCAACAAG

AAAAGAATGAACAAGATTTATTAGAATTAGATAAATGGGCAAGTTTGTGGAGTTGGTTTGACATAAC
AAACTGGCTGTGGTATATAAAAA

TATTCATAATGATAGTAGGAGGCTTGATAGGTTTAAGAATAGTTTTTACTGTAATTTCTATAGTGAAT
AGAGTTAGGCAGGGATACTCAC

CATTATCGTTGCAGACCCGCTTCCCAGCCCCGGGGGGACCCGACAGGCCCGAAGGAATCGAAGG
AGAAGGTGGAGAGAGAGACAGAGAAC

GATCAACTCGATTAGTGCATGGATTCTTAGCACTCATCTGGGACGATCTACGGAGCCTGTGCCTCT
TCAGCTACCACCGCTTGAGAGACT

TACTCTTGATTGCAGCGAGGGTTGTGGAACTTCTGGGACGCAGGGGGTGGGAAGCCCTCAAATAT
TGGGGGAGTCTTCTGCAGTATTGGA

GTCAGGAGCTAAAGAATAGTGCTGTTAGTTTGCTTAATACCACAGCTATAGTAGTAGCAGAAGGAAC
AGATAGGGTTATAGAAGTAGTAC

AAAGAATTTGTAGAGCTATTCGCAACATACCTAGAAGAATAAGACAGGGCTTTGAAAGAGTTTTGCT
ATAAGATGGGTGGCAAGTGGTCA

AAACGTAGTGGGGGTGGATGGTCAGTTGTAAGGGAAAGAATGAGACGAGCTGAGCCAGCATCAGA
GCCAGCAGCAGAGGGGGTGGGAGCA

GCATCTCGAGACTTGGAAAAATATGGAGCAATCACAAGTAGCAATACAGCAGCTACCAATCCTGATT
GTGCCTGGCTAGAAGCACAAGAG

GAGGAGGAGGTGGGTTTTCCAGTCAGACCTCAGGTACCTTTAAGACCAATGACTTACAAGGGAGCT
CTAGATCTTAGCCACTTTTTAAAA

GAAAAGGGGGGACTGGAAGGGATGATTCACTCCCAGAAAAGACAAGAAATCCTTGACCTGTGGGT
CTACCACACACAAGGCTACTTCCCT

GATTGGCAGAACTACACACCAGGGCCAGGGATCAGATATCCACTGACCTTTGGGTGGTGCTTCAA
GTTAGTACCAGTGGATCCAGATAAG

GTAGAAGAGGCCACTGAAGGAGAAAACAACTGCTTGTTACACCCTATGAACCAGCATGGGATGGAT
GATCCGGAGAAAGAAGTGTTACAG

TGGAAGTTTGACAGTCGCCTAGCATTTCACCATGTGGCCCGAGAGCTACATCCGGAGTACTACAAG
AACTGATGACACTGAGCTTTCTAC

AAGGGACTTTCCACTGGGGACTTTCCAGGGGAGGTGTGGCCAGGGCGGGACTGGGGAGTGGCGA
GCCCTCAGATGCTGCATATAAGCAGC

TGCTTTTTGCCTGTACTGGGTCTCTCTGGTTAGACCAGATCTGAGCCTGGGAGCTCTCTGGCTGAC
TAGGGAACCCACTGCTTAAGCCTC

AATAAAGCTTGCCTTGAGTGCTTTAAGTAGTGTGTGCCCGTCTGTTGTGTGACTCTGGTAACTAGAG
ATCCCTCAGACCCTTTAGTCAGT

GTGGAAAATCTCTAGCA

>02006_chr10_sc (9748 bp) Sequence of the provirus with ID chr10:41845251-42110306

TGGAAGGGATGATTTACTCCCAGAAAAGACAAGAAATCCTTGACTTGTGGGTCTACCACACACAAG
GCTACTTCCCTGATTGGCAGAACT

ACACACCAGGGCCAGGGACCAGATATCCACTGACCTTTGGGTGGTGCTTCAAGTTAGTACCAGTG
GATCCAGATAAAGTAGAAGAGGCCA

CTGAAGGAGAAAACAACTGCTTGTTACACCCTCTGAACCAGCACGGGATGGATGATCCAGAGAAG
GAAGTGTTAATATGGCAGTTTGACA

GTCACCTAGCATTTCACCATGTAGCTCGAGAGAAACATCCGGAGTACTACAAGAACTGATGACACT
GAGCTTTCTACAAGGGACTTTCCA

CTGGGGACTTTCCAGGGGAGGTGTGGCCTGGGCGGGACTGGGGAGTGGCGAGCCCTCAGATGCT
GCATATAAGCAGCTGCTTTTCGCCTG

TACTGGGTCTCTCTGGTTAGACCAGATCTGAGCCTGGGAGCTCTCTGGCTGACTAGGGAACCCACT
GCTTAAGCCTCAATAAAGCTTGCC

TTGAGTGTTTTAAGTAGTGTGTGCCCGTCTGTTGTGTGACTCTGGTAACTAGAGATCCCTCAGACCA
TTTAGTCAGTGTGGAAAATCTCT

AGCAGTGGCGCCCGAACAGGGACTTGAAAACGAAAGTAAGGCCAGAGGAGATCTCTCGACGCAG
GACTCGGCTTGCTGAAGCGCGCACAG

CAAGAGGCGAGGGGCGGCGACTGGTGAGTACGCCATTTTTGACTAGCGGAGGCTAGAAGGAGAG
AGATGGGTGCGAGAGCGTCAATATTA

AGCGGGGGAAAATTAGATGCATGGGAAAAAATTCGGTTAAGGCCAGGAGGAAAGAAACAATATAAG
CTAAAACATTTAGTATGGGCAAGC

AGGGAGTTAGAACGATTCGCGGTTAACCCTGGCCTGTTAGAAACATCTGAAGGCTGTAAACAAATA
ATAGCACAGCTACAACCATCCTTA

AAGACAGGATCAGAAGAACTTAAATCATTATTTAATACAGTAGCAGTACTCTATTGTGTACACCAGA
GGATAGAGGTAAGAGACACCAAG

GAAGCTTTAGATAGGATAGAGGAAGAGCAAAATAAAAGTAAGAAAAAGGAACAGCAAGCAGCAGCT
GACGCAGGAAGCAGCAGCAAGGTC

AGCCAAAATTACCCTATAGTGCAAAATCTACAGGGACAAATGGTACATCAGCCTATATCACCTAGAA
CTTTAAATGCATGGGTGAAGGTA

GTAGAAGAGAAGGCTTTCAGCCCAGAAGTAATACCCATGTTTACAGCATTATCAGAAGGAGCCACC
CCACAAGATTTAAACACCATGCTA

AACACGGTGGGGGGACACCAAGCAGCCATGCAAATGTTAAAAGAGACCATCAATGAGGAAGCTGC
AGAATGGGATAGATTGCATCCAGTA

CAGGCAGGGCCTGTTGCACCAGGCCAGATAAGAGAACCAAGGGGAAGTGACATAGCAGGAACTAC
TAGTACCCTTCAGGAACAAATAGCA

TGGATGACACATAATCCACCTGTCCCAGTAGGAGAAATCTATAAAAGATGGATAATCCTGGGATTAA
ATAAGATAGTAAGAATGTATAGC

CCTACCAGCATTCTGGACATAAGACAAGGACCAAAGGAACCCTTTAGAGACTATGTAGACCGGTTC
TATAAGACTCTAAGAGCTGAGCAA

GCTTCACAGGATGTAAAAAATTGGATGACAGAAACCTTGTTGGTCCAAAATTCAAACCCAGATTGTA
AGACTATTTTAAAAGCATTGGGA

CCAGGAGCTACACTAGAAGAAATGATGACAGCATGTCAGGGAGTAGGGGGACCCGGCCACAAAGC
AAGAATTTTGGCGGAAGCAATGAGC

CAAGCAACAGGTGCAGCTACCATAATGATGCAGAAAGGCAATTTTAGGAACCAAAGAAAAACTGTT
AAGTGTTTCAATTGTGGCAAAGAG

GGGCACATAGCCAGAAATTGCAGGGCCCCTAGGAAAAAGGGCTGTTGGAAATGTGGAAAGGAAGG
ACATCAAATGAAAGATTGTATTGAG

AGACAGGCTAATTTTTTAGGGAAAATCTGGCCTTCCCACAAGGGAAGGCCAGGGAATTTTCTTCAG
AGCAGACCAGAGCCAACAGCCCCA

CCAGCCCCACCAGAAGAAAGCTTCAGGTTTGGGGAAGACACAACAACTCCCCATCAGAAGCCGGA
GGTGATAGACAAGGAAATGTATCCT

TTGACTTCCCTCAAATCACTCTTTGGCAGCGACCCCTCGTCACAATAAAGATAGGGGGGCAGCTAA
AGGAAGCTCTATTAGATACAGGAG

CAGATGACACAGTATTAGAAGAAATGAATTTGCCAGGAAGATGGAAACCAAAAATGATAGGGGGAA
TTGGAGGTTTTATCAAAGTAAGAC

AGTATGATCAGGTGCCACTAGAAATATGTGGACATAAAGCTATAGGTACAGTATTAGTAGGACCTAC
ACCTGTCAACATAATTGGAAGAA

ATCTGTTGACTCAGCTTGGCTGTACTTTAAATTTTCCCATTAGTCCTATTGAAACTGTACCAGTAAAA
TTAAAGCCAGGAATGGATGGCC

CAAAAGTTAAACAATGGCCATTGACAGAAGAAAAAATAAAAGCATTAGTAGAAATTTGTACAGAAAT GGAAAAGGAAGGAAAAATTTCAA

AAATTGGGCCTGAAAATCCATACAACACTCCAGTATTTGCTATAAAGAAAAAAGATAGTACTAAATG GAGAAAATTAGTAGATTTCAGAG

AACTTAATAAGAGAACTCAAGACTTCTGGGAAGTTCAGTTAGGAATACCACATCCTGCAGGGTTAAA AAAGAAAAAATCAGTAACAGTAC

TAGATGTGGGTGATGCATATTTTTCAGTTCCCTTAGATAAAGACTTCAGGAAGTATACTGCATTTACC ATACCCAGTACAAACAATGAGA

CACCAGGGATTAGATATCAGTACAATGTGCTTCCACAGGGATGGAAAGGATCACCAGCAATATTCC AATGTAGCATGACAAAAATTTTAG

AGCCTTTTAGAAAACAACACCCAGATATAGTTATCTATCAATACATGGATGATTTGTATGTAGGATCT GACTTAGAAATAGGACAGCATA

GAACAAAAATAGAAGAACTGAGACAACACTTGTTAAAATGGGGATTTACTACACCAGACAAAAAACA TCAGAAAGAACCTCCATTCCTTT

GGATGGGTTATGAACTCCATCCTGATAAGTGGACAGTACAGCCTATAGTGCTGCCAGAAAAAGACA GCTGGACTGTCAATGACATACAGA

AGTTAGTAGGAAAATTAAATTGGGCAAGTCAGATTTACCCAGGGATTAAAGTAAGACAATTATGTAA ACTCCTTAGAGGAACCAAAGCAC

TAACAGAAGTAATAACTCTAACAGAAGAAGCAGAGTTAGAACTGGCAGAAAACAGGGAAATTCTAAA AGAACCAGTACATGGAGTGTATT

ATGACCCATCAAAAGACTTAATAGCAGAAATACAGAAGCAGGAGCAAGGCCAGTGGACATATCAAA TTTATCAAGAGCCATTTAAAAACC

TGAAAACAGGAAAATATGCAAGAGTGAGGGGTGCCCACACTAATGATGTAAGACAATTAACAGAGG CAGTGCAAAAAATAGCCACAGAAA

GCATAGTGATATGGGGAAAGACTCCTAAATTTAAACTGCCCATACAAAAAGAAACATGGGAAGCAT GGTGGACAGAGTATTGGCAAGCCA

CCTGGATTCCTGAATGGGAGTTTGTCAATACCCCTCCCCTAGTGAAGTTATGGTACCAGTTAGAAAA AGAACCCATAGTAGGAGCAGAAA

CTTTCTATGTAGATGGGGCAGCTAACAGAGAAACTAAACTAGGAAAAGCAGGATATGTAACTGACA GAGGAAGACAAAAGGTTGTCCCCC

TAACTGACACAACAAATCAGAAGACTGAGTTACAAGCAATTCAGCTAGCTTTGCAAGATTCGGGATC AGAAGTAAACATAGTAACAGACT

CACAATATGCATTAGGAATCATTCAAGCACAACCAGATAAGAGCGAATCAGAGGTAGTCAGTCAAAT AATAGAGCAGCTAATAAACAAGG

AAAAGGTCTACCTGGCATGGGTGCCAGCACACAAAGGAATAGGAGGAAATGGACAGGTAGATAAG TTAGTCAGTTCTGGAATCAGGAAGG

TACTATTCTTAGATGGAATAGATAAGGCCCAAGAAGACCATGAGAAATATCACAGTAATTGGAGAGC AATGGCTAGTGATTTTAACCTGC

CACCTGTAATAGCAAAAGAAATAATAGCCAGCTGTGATAAATGTCAGCTAAAAGGAGAAGCCATGC ATGGACAAGTAGACTGTAGTCCAG

GAATATGGCAGTTAGATTGTACACACTTAGAAGGAAAAATTATCCTGGTAGCAGTTCATGTAGCCAG
TGGATACATAGAAGCAGAAGTTA

TTCCAGCAGAGACAGGACAAGAAACAGCATACTTTATCTTAAAATTAGCAGGAAGGTGGCCAGTAA
AAACAATACATACAGACAATGGTA

GCAATTTCACCAGTACTACAGTTAAGGCCGCCTGCTGGTGGGCAGGGATCAAGCAGGAATTTGGC
ATTCCCTACAATCCCCAAAGTCAAG

GAGTAGTGGAATCTATGAATAAAGAATTAAAGAAAATTATAGGGCAAGTAAGAGATCAGGCTGAACA
TCTTAAGACAGCAGTACAAATGG

CAGTATTCATCCACAATTTTAAAAGAAAAGGGGGGATTGGGGGGTACAGTGCAGGGGAAAGAATAG
TAGACATAATAGCAACAGACATAC

AAACTAAAGAACTACAAAACCAAATTACAAAAATTCAAAAATTTCGGGTTTATTACAGGGACAGCAGA
GATCCACTTTGGAAAGGACCAG

CAAAACTTCTGTGGAAAGGTGAAGGGGCAGTGGTAATACAAGATAATAGTGACATAAAAGTAGTGC
CAAGGAGAAAAGCAAAGATCATTA

GGGATTATGGAAAACAGATGGCAGGTGATGATTGTGTGGCAAGTAGACAGGATGAGGATTAAAACA
TGGCACAGTTTAGTAAAACACCAT

ATGTATATTTCAAAGAAAGCTAAGGGATGGTCTTATAAACATCACTATGAAAACCCCCATCCAAGAG
TAGGTTCAGAAGTACATATCCCA

CTAGGGGATGCTAAATTGGTAGTAATAACATATTGGGGTCTGCATACAGGAGAGAGAGACTGGCAT
ATGGGTCAGGGAGTCTCCATAGAA

TGGAGGAAAAAGGAATATAGCACACAAGTAGACCCTAATACAGCAGACCAACTAATCCATCTGTATT
ACTTTGATTGTTTTTCAGAATCT

GCTATAAGAAGGGCCATATTAGGACATAGAGTTAGTCCTAGGTGTGAATATCAAGCAGGACATAAC
AAGGTAGGATCTCTACAGTACCTG

GCACTAGTAGCATTAGTAAAACAAAAAAGGACAAAGCCACCTTTGCCTAGTATTACAAAGCTGACAG
AGGATAAATGGAACAAGCCCCAG

AAGACCAAGGGCCACAGAGGGAGCCATACAATGAATGGACACTAGAGCTTTTAGAGGAGCTTAAAC
AGGAAGCTGTCAGACATTTTCCTA

GGCCATGGCTTCATGGCTTAGGGCAACACATCTATGAAACTTATGGAGATACTTGGGCAGGAGTGG
AGGCCATAACAAGAATATTGCAAC

AACTACTGTTTATTCATTTCAGAATTGGGTGTCGACATAGCAGAATAGGCATTATTCGACAGAGGAG
AGCAAGAAATGGAGCCAGTAGAT

CCTAGACTAGAGCCCTGGAAACATCCAGGAAGTCAGCCTAAAACTGCTTGTACCAATTGCTATTGTA
AAAAGTGTTGCTTTCATTGCCAA

GTCTGTTTCACAAAAAAAGCCTTAGGCATCTCCTATGGCAGGAAGAAGCGGAGACAGCGACGAAGA
GCTCCTCAAAGCAATCAGAATAAT

CAAGTTCCTCTATCAGAACAGTAAGTAATATATGTAATGCATTCTGAAGTAATATTAACAATAGTAGC
ATTAGTAGTAGCAGCAATAATA

GCAATAGTTGTGTGGACCATAGTACTCATAGAATATAGGAAAATATTAAGACAAAGAAAAATAGACA
GGTTAATTGATAGAATAATAGTT

AGAGCAGAAGACAGTGGCAATGAGAGTGAAGGGGACCAGGAAGAATTGTCAGCACTTGTGGACAT
GGGGCATGATGCTCCTTGGGTTGTT

AATGATCTGTAGTGCTGCAGACAATTTGTGGGTCACAGTCTATTATGGGGTACCTGTGTGGAAAGA
AGCAACCACCACTCTATTTTGTGC

ATCAGATGCTAAAGCATATGATGCAGAGGTACATAATGTTTGGGCTACACATGCCTGTGTACCCACA
GACCCCAACCCACAAGAAATAAA

ATTGGAAAATGTGACAGAAAATTTTAATATGTGGAGAAATGACATGGTAGAACAGATGCATGAGGAT
ATAATCAGTTTATGGGATCAAAG

CCTAAAGCCATGTGTAAAATTAACCCCACTCTGTGTTACTATGGATTGCCATCATGAAGTAAAGGTA
GAAAATGCTACTGGATTACAGAA

TACCACTTTTATTAATAATAAAACAGAAGAAATGAGAAACTGCTCTTTCAATACCACCACAAGCATAA
GAGATAAGATGCAGAAAGAATA

TGCAATTTTTTATAAACTTGATTTAGTACCACTAAATAATAATAAGGATAATAGTAGTAGTAATAGTAG
CTATAGATTGATAAATTGTAA

TACCTCAGTTATCACACAGGCCTGTCCAAAGATAACCTTTGAACCAATTCCCATACATTATTGTACC
CCGGCTGGTTTTGCACTTTTAAA

GTGTAATGATAAACAGTTTAATGGAACAGGGCCATGCTCAAATGTCAGCACAGTACAATGTACACAT
GGAATTAAGCCAGTAGTATCAAC

TCAATTGCTGTTAAATGGCAGTTTAGCAGAAGAAGAGGTAGTAATTAGATCTGAAAATTTCACTGAC
AATGCTAAGACCATAATAGTACA

GCTAAAAGAACCTGTAGAAATTAACTGTACAAGACCTGGCAACAATACAAGTAAAAGTATATCTATA
GGACCAGGGAGAGCATTTTATGC

AACAGGAAGAATAATAGGAGATATAAGACAAGCACATTGTAACCTTAGTAGATCACAATGGAATAAC
ACTTTAAAAGATATAGTTGCAAA

ATTAAGAGAACAATTTGGGAATAAAACAATAGTCTTTAATCGATCCTCAGGAGGGGACCCAGAAATA
GTAATGCACATCTTTAATTGTAG

AGGGGAATTTTTCTACTGTAATACAACAAAGCTGTTTAATAGTACTTGGCTGTCTAATAGTACTTTGA
ATTACACTGACCAGTCAAATGA

CACCATCACACTCCCATGCAAAATAAAACAAATTATAAACATGTGGCAGGAAGTAGGAAAAGCAATG
TATGCCCCTCCCATCCAAGGAAA

CATTAGTTGTTCATCAAAAATTACAGGGCTGTTATTAACAAGAGATGGTGGTAATAACAATAATGAGA
GCGCCCCAGAGATCTTCAGACC

TGGAGGAGGAAACATGAAGGACAATTGGAGAAGTGAATTATATAAATATAAAGTAGTAAAAATTGAA
CCATTAGGAGTAGCACCCACCAG

GGCAAAGAGAAGAGTGGTGCAGAGAGAAAAAAGAGCAGTAACACTAGGAGCTGTGTTCCTTGGGT
TCTTGGGCGCAGCAGGAAGCACTAT

GGGCGCAGCGTCACTGACGCTGACGGTACAGGCCAGACAATTATTGTCTGGTATAGTGCAACAGC
AGAACAATCTGCTGAGGGCTATTGA

TGCGCAACAGCATCTGTTGCAGCTCACAGTCTGGGGCATCAAGCAGCTCCAGGCAAGAGTCCTGG
CTGTAGAAAGATACCTACGGGATCA

ACAGCTCCTAGGGATTTGGGGTTGCTCTGGAAAACTCATTTGCACCACTACTGTGCCTTGGAATAC
TAGTTGGAGTAATAAATCTCTGGA

TCACATTTGGGGTAACATGACCTGGATGGAGTGGGAAAGAGAAATTGACAATTACACAAGCTTAATA
TATAACTTAATTGAAGAATCACA

GAACCAACAAGAAAAGAATGAAAAAGATTTATTAGAATTAGATAAATGGGACAGTTTGTGGAGTTGG
TTTGACATAACAAACTGGCTGTG

GTATATAAGAATATTCATAATGATAGTAGGAGGCTTGATAGGTTTAAAAATAGTTTTTACTGTAATTTC
TATAATAAATAGAGTTAGGCA

GGGATACTCACCATTATCGTTGCAGACCCGCTTCCCAGCCCCGGGGGGACCCGACAGGCCCGAA
GGAATCGAAGGAGAAGGTGGAGAGAG

AGACAGAGAACGATCAACTCGATTAGTGCATGGATTCTTCGCACTCATCTGGGACGATCTACGGAG
CCTGTGCCTCTTCAGCTACCACCG

CTTGAGAGACTTACTCTTGATTGCAGCGAGGGTTGTGGAACTTCTGGGACGCAGGGGGTGGGAAG
CTCTCAAATATTGGGGGAGTCTTCT

GCAGTATTGGAGTCAGGAGCTAAAGAATAGTGCTGTTAGTTTGCTTAATACCACAGCTATAGTAGTA
GCAGAAGGAACAGATAGGATTAT

AGAAGTAGCACAAAGAATTTGTAGAGCTATTCGCCATATACCTAGAAGAATAAGACAGGGCTTTGAG
AGAGCTTTGCTATAAGATGGGTG

GCAAGTGGTCAAAACGTAGTAGGGAGGGATGGTCAGTTATAAGGGAAAGAATGAGACGAGCTGAG
CCAGCAGCAGAGCCAGCAGCAGAGG

GGGTGGGAGCAGCATCTCGAGACTTGGAAAAATATGGAGCAATCACAAGTAGCAATACAGCAGCTA
CCAATGCTGATTGTGCCTGGCTGG

AAGCACAAGAGGAGGAGGAGGTGGGTTTTCCAGTCAGACCTCAGGTACCTTTAAGACCAATGAATT
ACAAGGGAGCTGTAGATCTTAGCC

ACTTTTTAAAAGAAAAGGGGGGACTGGAAGGGATGATTTACTCCCAGAAAAGACAAGAAATCCTTG
ACTTGTGGGTCTACCACACACAAG

GCTACTTCCCTGATTGGCAGAACTACACACCAGGGCCAGGGACCAGATATCCACTGACCTTTGGGT
GGTGCTTCAAGTTAGTACCAGTGG

ATCCAGATAAAGTAGAAGAGGCCACTGAAGGAGAAAACAACTGCTTGTTACACCCTCTGAACCAGC
ACGGGATGGATGATCCAGAGAAGG

AAGTGTTAATATGGCAGTTTGACAGTCACCTAGCATTTCACCATGTAGCTCGAGAGAAACATCCGGA
GTACTACAAGAACTGATGACACT

GAGCTTTCTACAAGGGACTTTCCACTGGGGACTTTCCAGGGGAGGTGTGGCCTGGGCGGGACTGG
GGAGTGGCGAGCCCTCAGATGCTGC

ATATAAGCAGCTGCTTTTCGCCTGTACTGGGTCTCTCTGGTTAGACCAGATCTGAGCCTGGGAGCT
CTCTGGCTGACTAGGGAACCCACT

GCTTAAGCCTCAATAAAGCTTGCCTTGAGTGTTTTAAGTAGTGTGTGCCCGTCTGTTGTGTGACTCT
GGTAACTAGAGATCCCTCAGACC

ATTTAGTCAGTGTGGAAAATCTCTAGCA

>02006_cen 9760 bp Sequence of the provirus with ID chr13:16283554-17732159

TGGAAGGGTTGATTTATTCCCAGCAAAGACAAGAAATCCTTGACTTGTGGGTCTACCACACACAAG GCTACTTCCCTGATTGGCAGAACT

ACACACCAGGGCCAGGGACCAGATATCCACTGACCTTTGGGTGGTGCTTCAAGTTAGTACCAGTG GATCCAGATAAAGTAGAAGAGGCCA

CTGAAGGAGAAAACAACTGCTTGTTACACCCTCTGAACCAGCACGGGATGGATGATCCAGAGAAG GAAGTGTTAATGTGGAAGTTTGACA

GTCGCCTAGCATTTCACCATGTGGCCCGAGAGCTACATCCGAGTACTACAAGAACTGATGACACTG TGCTTTCTACAAGGGACTTTCCAC

TGGGGACTTTCCAGGGGAGGTGTGGCCTGGGCGGGACTGGGGAGTGGCGAGCCCTCAGATGCTG CATATAAGCAGCTGCTTTTTGCCTGT

ACTGGGTCTCTCTGGTTAGACCAGATCTGAGCCTGGGAGCTCTCTGGCTGACTAGGGAACCCACT GCTTAAGCCTCAATAAAGCTTGCCT

TGAGTGCTTTAAGTAGTGTGTGCCCGTCTGTTGTGTGACTCTGGTAACTAGAGATCCCTCAGACCA TTTAGTCAGTGTGGAAAATCTCTA

GCAGTGGCGCCTGAACAGGGACTTGAAAGCGAAAGTAAGGCCAGAGGAGATCTCTCGACGCAGG ACTCGGCTTGCTGAAGCGCGCACGGC

AAGAGGCGAGGGGCGGCGACTGGTGAGTACGCCATTTTTTGACTAGCGGAGGCTAGAAGGAGAG AGATGGGTGCGAGAGCGTCAATATTA

AGCGGGGGAAAATTAGATGCATGGGAAAAAATTCGGTTAAGGCCAGGAGGAAAGAAACAATATAAG CTAAAACATCTAGTATGGGCAAGC

AGGGAGTTAGAACGATTCGCGGTTAACCCTGGCCTGTTAGAAACATCAGAAGGCTGTAAACAAATA ATAGGACAGCTACAACCATCCTTA

AAGACAGGATCAGAAGAACTTAAATCATTATTTAATACAGTAGCAGTACTCTATTGTGTACACCAGA GGATAGAGGTAAGAGACACCAAG

GAAGCTCTGGATAAGATAGAGGAAGAGCAAAATAAAAGTAAGAAAAAGGAACAGCAAGCAGCAGCT GACGCAGGAAGCAGCAGCAAGGTC

AGCCAAAATTACCCTATAGTGCAGAACTTACAGGGACAAATGGTACATCAGCCTATATCACCTAGAA CTTTAAATGCATGGGTGAAAGTA

GTAGAAGAGAAGGCTTTCAGCCCAGAAGTAATACCCATGTTTACAGCATTATCAGAAGGAGCCACC CCACAAGATTTAAACACCATGCTA

AACACGGTGGGGGGACACCAAGCAGCCATGCAAATGTTAAAAGAGACCATCAATGAGGAAGCTGC AGAATGGGATAGATTGCATCCAGTG

CAGGCAGGGCCTGTCGCACCAGGCCAAATAAGAGAACCAAGGGGAAGTGACATAGCAGGAACTAC TAGTACCCTTCAGGAACAAATAGCA

TGGATGACACATAATCCACCTGTCCCAGTAGGAGAAATCTATAAAAGATGGATAATCCTGGGATTAA ATAAAATAGTAAGAATGTATAGC

CCTACCAGCATTCTGGACATAAGACAAGGACCAAAGGAACCCTTTAGAGACTATGTAGACCGGTTC TATAAGACTCTAAGAGCTGAGCAA

GCTTCACAGGATGTAAAAAATTGGATGACAGAAACCTTGTTGGTCCAAAATTCAAACCCAGATTGTA
AGACTATTTTAAAAGCATTGGGA

CCAGGAGCTACACTAGAAGAAATGATGACAGCATGTCAGGGAGTGGGGGGACCCGGCCACAAAG
CAAGAATTTTGGCGGAAGCAATGAGC

CAAGCAACAGGTGCAGCTACCATAATGATGCAGAAAGGCAATTTTAGGAACCAAAGGAAAACTGTT
AAGTGTTTCAATTGTGGCAAAGAA

GGGCATATAGCCAAAAATTGCAGGGCCCCTAGGAAAAAGGGCTGTTGGAAATGTGGAAAGGAAGG
ACACCAAATGAAAGATTGTATTGAG

AGACAGGCTAATTTTTTAGGGAAAATCTGGCCTTCCCACAAGGGAAGGCCAGGGAATTTTCTTCAG
AGCAGACCAGAGCCAACAGCCCCA

CCAGCCCCACCAGAAGAGAGCTTCAGGTTTGGGGAAGAGACAACAACTCCCCATCAGAAGCCGGA
GGTGATAGACAAGGAAATGTATCCT

TTGGCTTCCCTCAAATCACTCTTTGGCAGCGACCCCTCGTCACAATAAAGATAGGGGGGCAGCTAA
AGGAAGCTCTATTAGATACAGGAG

CAGATGACACAGTATTAGAAGAAATGAATTTGCCAGGAAGATGGAAACCAAAAATGATAGGGGGAA
TTGGAGGTTTTATCAAAGTAAGAC

AGTATGATCAGGTACACATAGAAATATGTGGACATAAAGCTATAGGTACAGTATTAATAGGACCTAC
ACCTGTCAACATAATTGGAAGAA

ATCTGTTGACTCAGCTTGGCTGTACTTTAAATTTTCCCATTAGTCCTATTGAAACTGTACCAGTAAAA
TTAAAACCAGGAATGGATGGCC

CAAAAGTTAAACAATGGCCATTGACAGAAGAAAAAATAAAAGCATTAATAGAAATTTGTACAGAAATG
GAAAAGGAAGGGAAAATTTCAA

AAATTGGGCCTGAAAATCCATACAACACTCCAGTATTTGCCATAAAGAAAAAGGATAGTACTAAATG
GAGAAAATTAGTAGATTTCAGAG

AACTTAATAAGAGAACTCAAGACTTCTGGGAAGTTCAATTAGGAATACCACACCCTGCAGGGTTAAA
AAAGAAAAAATCAGTAACAGTAC

TAGATGTGGGTGATGCATATTTTTCAGTTCCCTTAGATAAAGACTTCAGGAAGTATACTGCATTTACC
ATACCTAGTACAAACAATGAGA

CACCAGGGATTAGATATCAGTACAATGTGCTTCCACAAGGATGGAAAGGATCACCAGCAATATTCC
AATGTAGTATGACAAAAATTTTAG

AGCCTTTTAGAAAACAACATCCAGATATAGTTATCTATCAATACATGGATGATTTGTATGTAGGATCT
GACTTAGAAATAGGACAGCATA

GAACAAAAATAGAAGAACTGAGACAACACTTGTTGAAGTGGGGATTTACTACACCAGACAAAAAACA
TCAGAAAGAACCTCCATTCCTTT

GGATGGGTTATGAACTCCATCCTGATAAATGGACAGTACAGCCTATAGTGCTGCCAGAAAAAGACA
GCTGGACTGTCAATGACATACAGA

AATTAGTGGGAAAATTGAATTGGGCAAGTCAGATTTACCCAGGGATTAAAGTAAGGCAATTATGTAA
ACTCCTTAGGGGAACCAAAGCAC

TAACAGAAGTAATAACTCTAACAGCAGAAGCAGAGTTAGAACTGGCAGAAAACAGGGAAATTCTAAA
AGAACCAGTACATGGAGTGTATT

ATGACCCCTCAAAAGACTTAATAGCAGAAATACAGAAGCAGGATCAAGGCCAATGGACATATCAAAT
TTATCAAGAGCCATTTAAAAACC

TGAAAACAGGAAAATATGCAAAAATGAGGGGTGCCCACACTAACGATGTAAGACAATTAACAGAGG
CAGTGCAAAAAATAGCCACAGAAA

GCATAGTAATATGGGGAAAGACTCCTAAATTTAAACTACCCATACAAAAAGAAACATGGGAAGCATG
GTGGACAGAGTATTGGCAAGCCA

CCTGGATTCCTGAATGGGAGTTTGTCAATACCCCTCCCCTAGTGAAGTTATGGTACCAGTTAGAAAA
AGAACCCATAGTAGGAGCAGAAA

CTTTCTATGTAGATGGGGCAGCTAACAGGGAAACTAAACTAGGAAAAGCAGGATATGTTACTGACA
GAGGAAGACAAAAGGTTGTCCCCC

TAACTGACACAACAAATCAGAAGACTGAGTTACAAGCAATTCAGCTAGCTTTGCAAGATTCGGGATC
AGAAGTAAACATAGTAACAGACT

CACAATATGCATTGGGAATCATTCAAGCACAACCAGATAAGAGCGAATCAGAGGTAGTCAGTCAAA
TAATAGAGCAGCTAATAAACAAGG

AAAAGGTCTACCTGGCCTGGGTGCCAGCACATAAAGGGATAGGAGGAAATGAACAGGTAGATAAAT
TAGTCAGTTCTGGAATCAGGAAAA

TACTATTTTTAGATGGAATAGATAAAGCCCAAGAAGATCATGAGAAATATCACAGTAATTGGAGAGC
AATGGCTAGTGATTTTAACCTGC

CACCGGTAATAGCAAAAGAAATAATAGCCAGCTGTGATAAATGTCAGCTAAAAGGAGAAGCCATGC
ATGGACAAGTAGACTGTAGTCCAG

GAATATGGCAACTAGATTGTACACATTTAGAAGGAAAAATTATCCTGGTAGCAGTTCATGTAGCCAG
TGGATACATAGAAGCAGAAGTTA

TTCCAGCAGAGACAGGGCAAGAAACAGCATACTTTATCTTAAAATTAGCAGGAAGGTGGCCAGTAA
AAACAATACATACAGACAATGGCA

GCAATTTCACCAGTACTACAGTTAAGGCCGCCTGCTGGTGGGCGGGGATCAAGCAGGAATTTGGC
ATTCCCTACAATCCCCAAAGTCAAG

GAGTAGTGGAATCCATGAATAAAGAATTAAAGAAAATTATAGGGCAAGTAAGAGATCAGGCTGAACA
TCTTAAGACAGCAGTACAAATGG

CAGTATTCATCCACAATTTTAAAAGAAAAGGGGGGATTGGGGGGTACAGTGCAGGGGAAAGAATAG
TAGACATAATAGCAACAGACATAC

AAACTAAAGAACTACAAAATCAAATTACAAAAATTCAAAAATTTCGGGTTTATTACAGGGACAGCAGA
GATCCACTTTGGAAAGGACCAG

CAAAACTTCTGTGGAAAGGTGAAGGGGCAGTGGTAATACAAGATAATAGTGACATAAAAGTAGTGC
CAAGGAGAAAAGCAAAGATCATTA

GGGATTATGGAAAACAGATGGCAGGTGATGATTGTGTGGCAAGTAGACAGGATGAAGATTAGAACA
TGGCACAGTTTAGTAAAACATCAT

ATATATATTTCAAAGAAAGCTAGGGGGTGGTCTTATAAACATCACTATGAAAACCCCCATCCAAGAG
TAGGTTCAGAAGTACATATCCCA

CTAGGGGATGCTAAATTGGTAGTAACAACATATTGGGGTCTGCATACAGGAGAAAGAGACTGGCAT
ATGGGTCAGGGAGTCTCCATAGAA

TGGAGGAAAAAGGAATATAGCACACAAGTAGACCCTAACACAGCAGACCGACTAATCCATCTGTAT
TACTTTGATTGTTTTTCAGAATCT

GCTATAAGAAAGGCCATATTAGGACATAGAGTTAGTCCTAGGTGTGAATATCAAGCAGGACATAACA
AGGTAGGATCTCTACAGTACCTG

GCACTAGTAGCATTAGTAAAACAAAAAAGAACAAAGCCACCTTTGCCTAGTATTTCAAAGCTGACAG
AGGATAGATGGAACAAGCCCCAG

AAGATCAAGGGCCACAGAGGGAGCCATACAATGAATGGACACTAGAGCTTTTAGAGGAGCTTAAGC
AAGAAGCTGTCAGACATTTTCCTA

GGCCATGGCTTCATGGCTTAGGGCAACACATCTATGAAACCTATGGAGATACTTGGGCAGGAGTG
GAGGCCATAACAAGAATATTGCAAC

AACTACTGTTTATTCATTTCAGAATTGGGTGTCGACATAGCAGAATAGGCATTACTCGACAGAGGAG
AGCAAGAAATGGAGCCAGTAGAT

CCTAGACTAGAGCCCTGGAAGCATCCAGGAAGTCAGCCTAAAACTGCTTGTACCAATTGCTATTGT
AAAAAGTGTTGCTTTCATTGCCAA

GTCTGTTTCACAAAAAAAGCCTTAGGCATCTCCTATGGCAGGAAGAAGCGGAGACAGCGACGAAGA
GCTCCTCAAGGCAATCAGAATAAT

CAAGTTCCTCTATCAGAACAGTAAGTAATATATGTAATGCATTCTGAAGTAATATTAACAATAGTAGC
ATTAGTAGTAGCAGCAATAATA

GCAATAGTTGTGTGGACCATAGTATTAATAGAATATAGGAGAATATTAAGACAAAGAAAAATAGACA
GGTTAATTGATAGAATAATAGAT

AGAGCAGAAGACAGTGGCAATGAGAGTGAAGGGGACCAGGAAGAATTGTCAGCACTTGTGGAGAT
GGGGCATGATGCTCCTTGGGTTGTT

AATGATCTGTAGTGCTGCAGACAATTTGTGGGTCACAGTCTATTATGGGGTACCTGTGTGGAAAGA
AGCAACCACCACTCTATTTTGTGC

ATCAGATGCTAAAGCATATGATGCAGAGGTACATAATGTTTGGGCTACACATGCCTGTGTACCCACA
GACCCCAACCCACAAGAAATAAA

ATTGAAAAATGTGACAGAAAATTTTAATATGTGGAGAAATGACATGGTAGAACAGATGCATGAGGAT
ATAATCAGTTTATGGGATCAAAG

CCTAAAGCCATGTGTGAAATTAACCCCACTCTGTGTTACTTTAGATTGCCATTATGAAGTAAAGATA
GAAAATGCCACTGGATTTGGGAA

TACCACTTTTATTAATAATAAAACAGAAGAAATGAGAAACTGCTCTTTCAATACCACCACAAGCATAA
GAGATAAGATGCAGAAAGAATA

TGCAATTTTTTATAAACTTGATTTAGTACAACTAGATAGTAATAAGCATAATAGTAGTAATAATACCAG
ATATAGATTGATAAATTGTAA

TACCTCAGTTATCACACAAGCCTGTCCAAAGATAACCTTTGAACCAATTCCCATACATTATTGTACCC
CGGCTGGTTTTGCGCTTCTAAA

GTGTAATGATAAAAAGTTTAATGGAACAGGGCCTTGCTCAAATGTCAGCACAGTACAATGCACACAT
GGAATTAAGCCAGTAGTATCAAC

TCAATTGCTGTTAAATGGCAGTTTAGCAGAAGAAGAGGTAGTAATTAGATCTGAAAATTTCACTGAC
AATGCTAAGACCATAATAGTACA

GCTAAAAGAACCTGTAGAAATTAACTGTACAAGACCCGGCAACAATACAAGTAAAAGTATAACTATA
GGACCAGGGAGAGCATTTTATGC

AACAGGGAGAATAATAGGAGATATAAGACAAGCACATTGTAACCTTAGTAGATCACAATGGAATAAC
ACTTTAAAAGATATAGTTAAAAA

ATTAAGAGAACAATTTGGGAATAAAACAATAGCCTTTAAAAATTCCTCAGGAGGGGACCCAGAAATA
GTAATGCACATCTTTAATTGTAG

AGGGGAATTTTTCTACTGTAATACAACAAAGCTGTTTAGTAGTATTTGGCTGTCTAATAGTACTTTGA
ATTACACTAAAAAGTCAAATAG

CACCATCAATGACACCATCACACTCCCATGCAGAATAAAACAAATCATAAACATGTGGCAGGAAGTA
GGAAAAGCAATGTATGCCCCTCC

CATCCAAGGAAACATTAGTTGTTCATCAAAAATTACAGGGCTGTTATTAACAAGAGATGGTGGTAAT
AACAATAATGAGAGCGCCCCAGA

AATCTTCAGACCTGGAGGAGGAAACATGAAGGACAATTGGAGAAGTGAATTATATAAATATAAAGTA
GTAAAAATTGAACCATTAGGAGT

AGCACCCACCAGGGCAAAGAGAAGAGTGGTGCAGAGAGAAAAAAGAGCAGTAACACTAGGAGCTG
TGTTCCTTGGGTTCTTGGGAGCAGC

AGGAAGCACTATGGGCGCAGCGTCATTGACGCTGACGGTACAGGCCAGACAATTATTGTCTGGTAT
AGTGCAACAGCAGAACAACCTGCT

GAGGGCTATTGATGCGCAACAGCATCTGTTGCAGCTCACAGTCTGGGGCATCAAGCAGCTCCAGG
CAAGAGTCCTGGCTGTAGAAAGATA

CCTAAGAGATCAACAGCTCCTAGGGATTTGGGGTTGCTCTGGAAAACTCATTTGCACCACTACTGT
GCCCTGGAATACTAGTTGGAGTAA

TAAATCTCTGGATCACATTTGGGGTAACATGACCTGGATGGAGTGGGAAAGAGAAATTGACAATTA
CACAAGCTTAATATACACCTTAAT

TGAAGAATCACAGAACCAACAAGAAAAGAATGAACAAGATTTATTAGAATTAGATAAATGGGACAGT
TTGTGGAGTTGGTTTGACATAAC

AAACTGGCTGTGGTATATAAGAATATTCATAATGATAGTAGGAGGCTTGATAGGTTTAAGAATAGTG
TTTACTGTAATTTCTATAGTAAA

TAGAGTTAGGCAGGGATACTCACCATTATCATTGCAGACCCGCTTCCCAGCCCCGGGGGGACTCG
ACAGGCCCGAAGGAATCGAAGGAGA

AGGTGGAGAGAGAGACAGAGAACGATCAACTCGATTAGTGCATGGATTCTTCGCACTCATCTGGGA
CGATCTACGGAGCCTGTGCCTCTT

CAGCTACCACCGCTTGAGAGACTTACTCTTGATTGCAGCGAGGGTTGTGGAACTTCTGGGACGCA
GGGGGTGGGAAGCCCTCAAGTATTG

GGGGAGTCTTCTGCAGTATTGGAGTCAGGAGCTAAAGAATAGTGCTGTTAGTTTGCTTAATACCAC
AGCTATAGTAGTAGCAGAAGGAAC

AGATAGGATTATAGAAGCAATACAAAGAATTTGCAGAGCTATTCGCAACGTACCTAGAAGAATAAGA
CAGGGCTTTGAAAGAGCTTTGCT

ATAAGATGGGTGGCAAGTGGTCAAAACGTGTTGGGGATGGATGGTCAGTTGTAAGGGAAAGAATG
AGACGAGCTGAGCCAGTAGCAGAAC

CAGCAGCAGAGGGGGTGGGAGCAGCATCTCGAGACTTGCAAAAATATGGAGCAATCACAAGTAGC
AATACAGCAGCTACCAATGCTGATT

GTGCCTGGCTGGAAGCACAAGAGGATGAGGAGGTGGGTTTTCCAGTCAAACCTCAGGTACCTTTA
AGACCAATGACTTACAAGGGAGCTG

TAGATCTTAGCCACTTTTTAAAAGAAAAGGGGGGACTGGAAGGGTTGATTTATTCCCAGCAAAGACA
AGAAATCCTTGACTTGTGGGTCT

ACCACACACAAGGCTACTTCCCTGATTGGCAGAACTACACACCAGGGCCAGGGACCAGATATCCA
CTGACCTTTGGGTGGTGCTTCAAGT

TAGTACCAGTGGATCCAGATAAAGTAGAAGAGGCCACTGAAGGAGAAAACAACTGCTTGTTACACC
CTCTGAACCAGCACGGGATGGATG

ATCCAGAGAAGGAAGTGTTAATGTGGAAGTTTGACAGTCGCCTAGCATTTCACCATGTGGCCCGAG
AGCTACATCCGGAGTACTACAAGA

ACTGATGACACTGTGCTTTCTACAAGGGACTTTCCACTGGGGACTTTCCAGGGGAGGTGTGGCCTG
GGCGGGACTGGGGAGTGGCGAGCC

CTCAGATGCTGCATATAAGCAGCTGCTTTTTGCCTGTACTGGGTCTCTCTGGTTAGACCAGATCTGA
GCCTGGGAGCTCTCTGGCTGACT

AGGGAACCCACTGCTTAAGCCTCAATAAAGCTTGCCTTGAGTGCTTTAAGTAGTGTGTGCCCGTCT
GTTGTGTGACTCTGGTAACTAGAG

ATCCCTCAGACCATTTAGTCAGTGTGGAAAATCTCTAGCA

>BosT_ERV_chr11_APOB 6777 bp

TGCGGGGAGCCGGTGAGGCATTCCACTCGTGACAAAGGTCATGAGGAAGGAGGCTCGGCATACG
CAAAGGCGGGATCGAGCCTCAGGAGT

CCCCCCGGATATTCTCGAGCATTTTCCCCCAAAAAACCAGAGTCTGCCTACTTTATTGCTTTGTGCT
CTCACCTCTGACTTTACTGGGGG

CTGTCCCCTACCACCGTCTCTCTCTCTCTGTGTCAAAGAGTTAACTTACAGCTCCAATTAATAAAGTT
CCTGGGCAATTAGGAGTGTTTA

AATCCAAACCCCTCTGATGGCTCTCTAACTCGCCTGACAAGTTTACCCGGACTCCTGCAGCTATGC
ATACGATTGTTTACAGTCTCCCAG

CCTCGAGAGGCATGGGAAGCTTAAGATATTCAAATAGCTTAGAGCCTCTCAGAGAGTTAAAAACTG
TCAGAATAAACTAGTAAAGGATTT

CATTGATGAGTCAATGCTTGTTGCCAAGTTTTCACATCCCCTGAATTGTATCCTTGAATATGTATCAA
TTAATAGTGGGTATGTAGAAAA

AATAAGTAGTGGCCTTGGTGTTAGTAACTTTAGACCCTTAAGGTAATAAATTCTTTCTTTGTTGTAAA
CCCATTACACATCCGCCCTATA

GGAATGCAATTTTATCTTTGGAAGATGGTGCCAAACCTTGAAATAATTACTCTTAGAGAAAGTAAGT
CTTTGTTGATAAGTCCTTGTCAA

GAGTCATAAAATGTTAGTAGGCCTTCTGGCCAGAAGATGATGTAAATCACCTAAACCATTTGTATAC
GATACATTTGCAGGAAAGAAACC

TTGGTTTTTGATAAGAATCAAAGACTGCTGACTTTGCATCCCCTATTATCCTCTATGTGTAACTTAGG
GTATAAAAGCCCCTGTTAAAAA

TAAAGCTACGGGCCTTGCTCACCAACGCTTGGTCTCCCCATGTCATTCTTTTAACTTCCAGCTGAGT
CTCCATCTGGAGCGCGGAACCCA

CCACGCTTACTAATCATGCCTGGGCTTCTAAGACCCACTCGAGAAGGTGTCTAGGGTGAGACACCT
TCCGCTATTCGAGAGGGCGCCTGC

GGCCTACGTAAGTGGTGCAAACTTCTTGTCTTGAAGTTTTATTGGTCTCCCGCGTAAACCAAGCTAC
TCAGCTTCTTTTCTCCACTGAAA

TTTCCTACTGAGCTATCCTCATTCTATTGTTCTCTATATCCCTAATTAGCATATAAATAGTCGCCGAC
GCCGTCTCCCCTTCGAATACCC

TGGATCAGCCGGGGCTGGTCCTCGGCAGGTGGCGCCCGATACAGGGATTTCGAAGGTAAGTCCC
CAACCCCATTCCCCCAGTGTTGAGTT

TTCGGGACGGATAGGACCCCACTTAGGGTGCTGCAGACCCCCCTGTAGAATAGACAGGGAGAGAG
GAGTGGGAAGTGTTGAAAGTGTTGA

AGAGTTAGAGAGAAAAAACGATTTCTAAAAAGGCTGACAAAAAAGGCCTAATCTTTTGATAGCTAAA
AGCAAAATCTTTTACTATACTTA

ATTTTCTGACATGGGTAATACTGAATCAAATGAAAGACAGCTCTTTATAGGAGTAATTTTACAGTTAT
TAGGTAAAAGAGGAATTAAAGT

TAAAAAATCTGCCATTCAATCATTTCTTTCATTTGTACAAGAGCACTGTCCCTGGTTTCCAGACGAAG
GCTCTGTTAACTTAGATGTCTG

GGAAAAAGTAGGAAAACAGTTAAAAACTTACCATGCAGAACATGGCTCAGAAAAGGTGCCTAATGA
CGCCTTTTCCTTATGGAATATTAT

TAGAGATGTCTTAGACCCTGCCCCAGATTCAGAAAAAGTACATCTTAAAAGGGATAGTGAAGAAAAT
GCTGTAGTTAAACCTACCCCTGA

ACCTAAAAAAGTAACCTTTAAAGAGGAAAATGAAGTCGAAATCGTAGTTAAACCTGAAGAAAATGAG
AAAAACGAAGACCCGCCTGACTA

TCGGCAACTAAGAAAAATGTTAATAGCCGTGACTACTCAGGAACAACCTAAAGATAGGGATGAGGA
ACAAGATCAGCCTTCCCCAAAACA

AAAAGAGAATTTAGGCGAGATAACAACTAAATATCACTCTGATGGAGATTGGCATCTCTTAAACAAA
GATGCCCCAAAAGGCCTAAGAGA

AACTTCCCCTGTCAGACCATCCGCTCCTAGGAGCTTGAGGGAAACATCCCCGATCAGACAGTTTGT
AAGATTTAGTCACCAAACAGTTAA

AGGATCTCCGGAGCAGGAGAAGAGAAATAGGGGACGCTCCCATCCCTCCATGCCTCCTCCTCTGT
GTGGGGGTAAAGGGCCTCCACTAGG

AGTTTCCCCGAGAAGGGTTTGCTCTAGTCCCAAGGATAAATTTGATCCTCACCTTGAGGCTTCTTTT
CTAGTCCCCTCTGCAGTTTAGAT

AGGGTAAAGAAGGTACAAAATAAATAAAAATATTCTCAAAAAAAAAAAAATAAATAAAAAAAAAAATG
GGTTTCTCTGCATCTAAGAATA

TACAAATGTTTATTGGCTCTGTGAAATATGATTAGAAATGTCCTGGACCCCTCTCATGAGGCTGTTA
AATAGCCTTTGAGGGAGGCTATA

GCGGTAGATGGTGGTGAGTCTCCAGTTTCTGCATAGATCATAATTTCTGCTATTGGAAAAAAAAAAA
AAAAGGAGGGAGAAAGTGCTTTA

CCTCCCGAGCAAGGAGAGGGTTTACAGGACGCTGCGGCCGAGCGCCCTGGCGAGCTCCCTCCCC
CTCTGCGCAAACCTTTAAAAAACTTA

TCCACCACTTTCTGATTTAAGGTGATCGCCACCGCCTCCGTTTGTGCCTCCCAGGACCCAGGAGTT
CCCCACTTTGCCCCCAAAGCCTCT

CAAAGCGTTGCCTAAGCCTGAGGAAGATAAAAAGTTTTTTGAACAGTGGAGCTTTTAAAACAGACTG
CGTGCACAATATGCAGAGCATGC

TTCTTAGAGAAAACCCCCTCAGGGGGGGTCTCACCCAGGCTAGGAGAAAAGCGAAACCGCAAGGG
GATTTAGCAACAATATTAACCCCTG

ATGTGCCGCTTACTCCAATTCCAACGGGAGTGGCTGGCCCCCTACCTTTGGACATTGTAAGATCTG
TCCTGGGGCATAGCTCGCTTTCTT

TTCAAAAAAAAAAAAAAAAAAATTCGGTGGTGCATGGTGTAGTAGATTCTGATTATATTGAGAAATT
AAAGTCTTGATATCACCGCCTA

CCAAAACTGTACAAATTAATAAAGGTCAAAGAGTAACACAGCCTTTGCTTTTACCTTATTATCAGACA
AGAAAAAAACTTGACTTCTCAAG

TTAAGAGCCACGAAACATTTGGATCTAGTGATCTAGCCTTTTGGATGCAGGAAATTACAGCTCCTGG
CCAACACATACTACTGAAAATGA

GTTGGTGGGATTAGAGAAAGTGGTATGTGAGGTGGGGATGCTATACAGGTGGTACATTTAACAATA
CTTTATATATTGTTATAAATACAT

AATATAAATATATTTGCCTAATTACAGTTTGCCTAATTAGGTATGGGTATAAATAAAATATAATAAAGG
TATACCTAATTCTACTTATAG

ATCTATACTTAATTTGTATATAAATTAATATGTATACTATATATACATATATATATACTGTATAATTAAAT
GTATATTGCAGTAATATAA

TGTGTGTGGCTGATATTAATTGGTATGGATTGGTGTGCTGTGATGATATATGTTCTATATACTGTATA
ATTGTATATGTGTGACATGCAT

TATTGCAGTACATTGGTTTGTGTTGGTTGGTATTAGCTGTGTATGTGTTGTATTGCTGTAATATATAT
TAATTAATATATTAATTATAAA

GATTAATTCAAATATATTGATTTATATATATTATATGTCAATAAGTTTATACTTGTTGCCATATATAAAT
ACATATATAAATACATTTTA

CATTTAGGTATGTCTGTATACCAAAATGAGATAAGGAGGTTATACATTTATTACTTAAATTTATACAG
AGACCTAAACTAAACATTAGAC

CTAAATTAACATGTCCCTAGGAAAACAGCTGGACAAATAGTAAAGTTATGTCCAAATTGTAATTAATC
ACTTAAAGGTAAACACACAGGG

GCAGACTACAATGATGAGACACTAGATGCCTGAATTACAGGCTACGACTCGGCCCCTGGTCAAGTA
AAAAGACCTCCTTACTGGAGAGTG

TAAAAGGCCAGATGTGTTGCTAACTTGTGGGAGAGGGTATGCTTATATATTTCTACAGAATGCAGAT
TCTCCGATTTGGATCTCAGAAAA

ATTCGTCATGTCACTTTCCCCAAAGACCAGGTTCGCGGCGCGCGCACTCGCTCGCTCCCCCTCCC
CCAGCCAGCTCTCTCCGCCGCCAGC

GGCGCCCTGCACCCTCCTTGCTGCACCCCGAGACCTAGAGCAAAGAAGTCTGTGCGGCGAGTGA
GGGCCAGAGAGGAAAGCGCGCCCGCG

CAGTGCAGTCCAGACCAGCGCGCTCCCGCCGCGGGCGACAGGAAAACGCCCCGAGCCCAAGCG
GCGCGGCTAGCCCGAGTCCGCGAACCC

CGCCCCTCCGCCCGCCGTAGACGCCTCGGCCCACAACCTGCCGTTCGCGTTCTGCCTCCTGATGC
TGCGGACACTGCTCCGCCGAGCCGA

TGCCTACAGCTGCTCCTTGTCTCATCTTGTTTAAATCCTCAATATAATGTTTGCTCCTTTGTAATGTT
ACAATGTCCACCTTATCTTATG

ATGTAACCACTTATTGAATTATGCTCTTGCTGTATTACAACAACTGCAGGATTTAATGCGAACACGAC
GGTTTGTGGGCTTACTTATCTT

GGGAATAGCAGCTTTGATAAGCGCAATTACTTCTGTTACTGTGGCAGCAATATCATTGACTCAACAA
GTACATACTGCTCAATATGTTGA

TTCTATGTCCAAAAATGTTTCTTTAGCATTGGCAACACAGGAAGCTATAGACAGGAAATTAGAAATG
AGGGTAGATGCCCTAGAGGAAGC

AGTAATACATATTGGGACTGAATTGCAGGCTTTAAAGGTGAAAATGGCATTGTCCTGTCATGCTGAC
TACCGGTGGATATGTGTAACACC

CCTGAAAGTAAATGAGACAGATTTTGAATGGGAAAAGATTAAAAACCATATTTCAGGTATTTGGAAC
AGCTCTGACATTAGCTTAGACTT

AGGGAAACTTCACAATCAAATAGCAACCCTGGAACACTCCCGATTAGATTTTACTGCCGCTGGAAC
AGCAAATGATTTCTTCCATACTTT

CTCTAACTACATTTCAGGAAAAAATATTCTGTCTACCTTCCTCGGCTACGCTACCTTGGCTGTTTTAA
TTTTATTTCTAATAATCATTCT

TCCTTGTATTGTCAGGATTCTTCGGCAGAGCATTCAGAGGCTCGCGACTGAGCTACATCTGGCTGT
TTTAAGAAATAAAAAAGGGGGAGA

TGCGGGGAGCCGGTGAGGCATTCCACTCGTGACAAAGGTCATGAGGAAGGAGGCTCGGCATACG
CAAAGGCGGGATCGAGCCTCAGGAGT

CCCCCCGGATATTCTCGAGCATTTTCCCCCAAAAAACCAGAGTCTGCCTACTTTATTGCTTTGTGCT
CTCACCTCTGACTTTACTGGGGG

CTGTCCCCTACCACCGTCTCTCTCTCTCTGTGTCAAAGAGTTAACTTACAGCTCCAATTAATAAAGTT
CCTGGGCAATTAGGAGTGTTTA

AATCCAAACCCCTCTGATGGCTCTCTAACTCGCCTGACAAGTTTACCCGGACTCCTGCAGCTATGC
ATACGATTGTTTACAGTCTCCCAG

CCTCGAGAGGCATGGGAAGCTTAAGATATTCAAATAGCTTAGAGCCTCTCAGAGAGTTAAAAACTG
TCAGAATAAACTAGTAAAGGATTT

CATTGATGAGTCAATGCTTGTTGCCAAGTTTTCACATCCCCTGAATTGTATCCTTGAATATGTATCAA
TTAATAGTGGGTATGTAGAAAA

AATAAGTAGTGGCCTTGGTGTTAGTAACTTTAGACCCTTAAGGTAATAAATTCTTTCTTTGTTGTAAA
CCCATTACACATCCGCCCTATA

GGAATGCAATTTTATCTTTGGAAGATGGTGCCAAACCTTGAAATAATTACTCTTAGAGAAAGTAAGT
CTTTGTTGATAAGTCCTTGTCAA

GAGTCATAAAATGTTAGTAGGCCTTCTGGCCAGAAGATGATGTAAATCACCTAAACCATTTGTATAC
GATACATTTGCAGGAAAGAAACC

TTGGTTTTTGATAAGAATCAAAGACTGCTGACTTTGCATCCCCTATTATCCTCTATGTGTAACTTAGG
GTATAAAAGCCCCTGTTAAAAA

TAAAGCTACGGGCCTTGCTCACCAACGCTTGGTCTCCCCATGTCATTCTTTTAACTTCCAGCTGAGT
CTCCATCTGGAGCGCGGAACCCA

CCACGCTTACTAATCATGCCTGGGCTTCTAAGACCCACTCGAGAAGGTGTCTAGGGTGAGACACCT
TCCGCTATTCGAGAGGGCGCCTGC

GGCCTACGTAAGTGGTGCAAACTTCTTGTCTTGAAGTTTTATTGGTCTCCCGCGTAAACCAAGCTAC
TCAGCTTCTTTTCTCCACTGAAA

TTTCCTACTGAGCTATCCTCATTCTATTGTTCTCTATATCCCTAATTAGCATATAAATAGTCGCCGAC
GCCGTCTCCCCTTCGAATACCC

TGGATCAGCCGGGGCTGGTCCTCGGCA
