# Supplementary material for: PCIP-seq: simultaneous sequencing of integrated viral genomes and their insertion sites with long reads
Source: Genome Biol. 2021 Apr 6;22:97. doi: 10.1186/s13059-021-02307-0 (PMC8025556; doi:10.1186/s13059-021-02307-0)
Supplement: Supplementary file 5 — Additional file 5: Review history. [file 13059_2021_2307_MOESM5_ESM.docx]

Review History

**First round of review**

**Reviewer 1**

**Were you able to assess all statistics in the manuscript, including the appropriateness of statistical tests used?** Yes: There is no problem.

**Were you able to directly test the methods?** No

**Comments to author:**

Maria Artesi et al demonstrate that new method they developed is quite useful to analyse viral genome integrate in the host DNA. There are several protocols available for the characterisation of integrated viral genome. However, each protocol has advantages and disadvantages. As authors mentioned in this manuscript, ligation-mediated PCR is one of major methods to analyse integrated viral genome. The protocol is very sensitive to detect viral integration sites but not to characterise whole viral sequence. FLIPS gives us proviral sequence information but does not tell us viral integration site.

Both whole viral sequence and viral integration site are critical information to study viral persistence and pathogenesis. MIP-seq method is able to provide both information but it is not easy to perform due to its technical and economical requirements.

PCIP-seq utilised CRISPR-Cas9 and ONT system to increase sensitivity of viral sequence detection as well as sequencing length to cover whole viral sequence.

This new method seems to be an outstanding invention to characterise viral genome integrated into the host DNA with comprehensive and highly sensitive manner.

The paper will be informative for the readers of Genome biology, but there several points they need to revise to make the paper clear for the readers.

Major points

1. The figures in current version are not clear.

I do recommend to think more carefully how to show their data to the readers.

I understand the importance of the study, but I need to spend some time to get the points.

For example,

a. why Fig1a does not contain DNA fragment with 3'LTR

b. Font size is quite variable even in the same figure 1. Some characters are hard to read.

c. Table1 It took some time to understand what pure host or pure viral reads mean.

d. Figure 2 b, c, d

Current IGV pictures is difficult to understand, Please revise them for readers to get the points without spending much time.

e. Figure 4&5 Pie charts are quite big but chromosome numbers are hard to see.

2. Title

It seems to me that there is a gap between content of the paper and the title of the paper "Unravelling virus-host dynamics by simultaneous sequencing of integrated viral genomes and their insertion sites with long reads". If we see the abstract, new technology the authors developed in this study is the main topic.

3. Contents of the data

There are so much data, including HTLV-1, HIV-1, BLV, ERV, and HPV.

The manuscript would be much clearer than current version if the authors remove some of the data, such as HPV.

Then they will be able to have more space to describe their findings with detailed explanation. That should be useful to increase the understanding of the readers.

Minor points

1. Abstract

The authors analyse ERV but the information is missing in the abstract.

2. p4 line 3-4 "the provirus itself is largely unexplored"

Some studies actually analysed the provirus itself. However, there are very limited studies to analyse both the insertion point and the provirus itself.

3. p6 line 12-15,"We found that using a pool of CRISPR guides for each region increased the efficiency and by multiplexing the guide pools"

It would be informative for the readers if the authors show the data to support the idea.

4. p7 line 26-27, "In general, PCIP-seq identified more insertion sites, using less input DNA than our Illumina based method"

This is quite appealing point of this method. I wonder what is the possible reason(s) for this advantage.

Because sequencing depth does affect the depth of information we get from the data, it would be more convincing if they show the advantage is not due to sequencing depth.

To show this point clearly, it may be informative to show the data with different sequencing depth in silico.

5. p10 line 7 "HIV-1 reservoir"

HIV-1 reservoir contain not only HIV-1 provirus but also tissue reservoir, no-T cell reservoir. The authors used U1 cells in this study. The U1 cell is not CD4 T cells. This is not the main point of study, but I suggest the authors to reconsider how to describe this part.

6. p11 line 20- MDA experiment

I am not sure that PCIP-seq with MDA experiment is essential to include this study.

7. p13 line 11 "DNA extracted from Pap"

What does Pap mean?

8. p16 line 15-17

To tackle with the HIV-1 provirus that can serve as viral reservoir, we need to characterise intact provirus without mutation.

For the purpose, we don't have to analyse all HIV-1 provirus.

I mean the author can describe that PCIP-seq will be potent to detect intact HIV-1 proviruses, which should be evaluated to measure HIV-1 reservoir in the infected cells.

**Reviewer 2**

**Were you able to assess all statistics in the manuscript, including the appropriateness of statistical tests used?** No

**Were you able to directly test the methods?** No

**Comments to author:**

The paper developed an interesting method called PCIP-seq based on the third generation sequencing for analyzing integration of viral genomes to host genomes. Taking advantage of long reads of the third generation sequencing, other than detecting integration sites, PCIP-seq also allows detailed investigation of viral genomes integrated in the host genomes and allows accurate detection of integration sites in repetitive regions. The proposed PCIP-seq method is potentially very useful for studying virus-host dynamics. However, the paper needs to be considerably improved before publication.

1. The resolutions of the main figures are very low (the supplementary figures are OK), making it very hard to read the main figures. The figure legends are not clear. How to understand Figure 2 b,d,e, Figure 3b, etc.? Circus plots in Figure 4 and 5 cannot provide much information. They can only roughly show integration sites. More detailed analyses of the integration sites are needed.

2. More analysis of PCIP-seq data is needed to show the advantage of PCIP-seq. For example, what percentage of nanopore reads cover integrate sites? If a read covers an integration site, usually how many nucleotides are from the host genome and how many are from the viral genome (it is more helpful to show the length distributions)? The authors showed the percentage of Illumina integration sites also found in PCIP-seq, but what about the percentage of PCIP-seq integration sites also found in the Illumina data (to measure the specificity of the PCIP-seq method)?

3. One big advantage of PCIP-seq over existing methods is that it allows investigation of proviral sequences in the host genome such as analyzing SNPs, indels in the proviral sequences. However, nano-pore sequencing has a very high sequencing error rate and SNP/indel analysis generally need a very high coverage to achieve reasonable sensitivity and specificity. It seems that for most integration sites, there are usually only a handful of supporting PCIP-seq reads, making SNP/indel analysis very difficult for the majority of the integration sites. The advantage of PCIP-seq over existing methods thus seems significantly jeopardized. More analysis should be provided in this respect.

**Authors Response**

**Point-by-point responses to the reviewers’ comments:**

Reviewer 1:

Maria Artesi et al demonstrate that new method they developed is quite useful to analyse viral genome integrate in the host DNA. There are several protocols available for the characterisation of integrated viral genome. However, each protocol has advantages and disadvantages. As authors mentioned in this manuscript, ligation-mediated PCR is one of major methods to analyse integrated viral genome. The protocol is very sensitive to detect viral integration sites but not to characterise whole viral sequence. FLIPS gives us proviral sequence information but does not tell us viral integration site.

Both whole viral sequence and viral integration site are critical information to study viral persistence and pathogenesis. MIP-seq method is able to provide both information but it is not easy to perform due to its technical and economical requirements.

PCIP-seq utilised CRISPR-Cas9 and ONT system to increase sensitivity of viral sequence detection as well as sequencing length to cover whole viral sequence.

This new method seems to be an outstanding invention to characterise viral genome integrated into the host DNA with comprehensive and highly sensitive manner.

The paper will be informative for the readers of Genome biology, but there several points they need to revise to make the paper clear for the readers.

Major points

1. The figures in current version are not clear.

I do recommend to think more carefully how to show their data to the readers.

I understand the importance of the study, but I need to spend some time to get the points.

For example,

a. why Fig1a does not contain DNA fragment with 3'LTR

b. Font size is quite variable even in the same figure 1. Some characters are hard to read.

c. Table1 It took some time to understand what pure host or pure viral reads mean.

d. Figure 2 b, c, d

Current IGV pictures is difficult to understand, Please revise them for readers to get the points without spending much time.

e. Figure 4&5 Pie charts are quite big but chromosome numbers are hard to see.

*Unfortunately, the low resolution of the figures was caused by the PDF generation system. The original files provided were high-resolution. We hope the resolution of the updated figures will be satisfactory in the PDF generated by the journal upon this new submission. We also provide high-resolution figures in TIFF format and would recommend examining these original figures/files to avoid any technical concern. Regarding the reviewer’s specific remarks (a to e), we have revised Figures 1, 2 and 3 and have updated the corresponding figure legends to make them clearer. We agree the original figures requested some expertise in retrovirus genomics and a good knowledge of the IGV genomic viewer tool, making it hard for the reader to get the points without spending too much time. We thank the reviewer for pointing to this weakness.*

*For Figures 4 & 5 we have made the chromosome numbers clearer as requested. Table 1: we have clarified the meaning of “chimeric reads” and “pure host/pure viral reads” by adding a description below the table. Legends to Figures 1-5 have been updated to improve detail and clarity.*

2. Title

It seems to me that there is a gap between content of the paper and the title of the paper "Unravelling virus-host dynamics by simultaneous sequencing of integrated viral genomes and their insertion sites with long reads". If we see the abstract, new technology the authors developed in this study is the main topic.

*We have modified the title to: PCIP-seq: simultaneous sequencing of integrated viral genomes and their insertion sites with long reads.*

3. Contents of the data

There are so much data, including HTLV-1, HIV-1, BLV, ERV, and HPV.

The manuscript would be much clearer than current version if the authors remove some of the data, such as HPV.

Then they will be able to have more space to describe their findings with detailed explanation. That should be useful to increase the understanding of the readers.

*We feel that by having data from multiple viruses in the manuscript this helps show the versatility of the method. Regarding HPV, we especially think that including this virus shows the flexibility of the method and its applicability to viruses for which integration in the host genome is not part of the natural life cycle. Also, because existing methods are not capable of identifying HPV integrations at early time points and given the disease burden caused by HPV we think it useful to keep this in the main text.*

Minor points

1. Abstract

The authors analyse ERV but the information is missing in the abstract.

*We have added this to the revised abstract.*

2. p4 line 3-4 "the provirus itself is largely unexplored"

Some studies actually analysed the provirus itself. However, there are very limited studies to analyse both the insertion point and the provirus itself.

*This phrase has been modified in the revised manuscript.*

3. p6 line 12-15,"We found that using a pool of CRISPR guides for each region increased the efficiency and by multiplexing the guide pools"

It would be informative for the readers if the authors show the data to support the idea.

*This information has been added in Supplementary Information, section “Supplementary Note”.*

4. p7 line 26-27, "In general, PCIP-seq identified more insertion sites, using less input DNA than our Illumina based method"

This is quite appealing point of this method. I wonder what is the possible reason(s) for this advantage. Because sequencing depth does affect the depth of information we get from the data, it would be more convincing if they show the advantage is not due to sequencing depth. To show this point clearly, it may be informative to show the data with different sequencing depth in silico.

*We did not systematically explore this in greater depth as the amount of input DNA varied quite a bit across the PCIP-seq libraries but was consistent in the Illumina libraries. In samples 220 and 1439 (BLV ovine and bovine samples respectively; see Table 1 for sample characteristics) the amount of DNA used and number of raw reads in PCIP-seq was lower than in the Illumina library, but the number of integration sites identified by PCIP-seq was higher or equivalent. (In 560, PCIP-seq only used ¼ of the input DNA, had lower raw reads numbers, but only identified ~55% less integration sites than the Illumina approach). For 221 (032014) both PCIP-seq and the Illumina libraries used 4μg, the PCIP-seq library had fewer raw reads but identified more integration sites. For 221 (022016) the number of Illumina reads is considerably lower than that in the Nanopore experiment. We down-sampled the PCIP-seq reads to approximately the same level as the Illumina library. Using 9396 PCIP-seq reads approximately 3000 insertion sites were identified. As a consequence, we do not think the difference in efficiency is due to sequencing depth.*

5. p10 line 7 "HIV-1 reservoir"

HIV-1 reservoir contain not only HIV-1 provirus but also tissue reservoir, no-T cell reservoir. The authors used U1 cells in this study. The U1 cell is not CD4 T cells. This is not the main point of study, but I suggest the authors to reconsider how to describe this part.

*We agree the HIV-1 reservoir is broader and comprises no-T cell tissue reservoir in addition to (CD4) T cells. However, the U1 (pro-monocytic cell line) experiments here are mainly presented as a proof of concept to show that the method can capture integration sites even when the proviral load is very low rather than to address its applicability to no-T cell types. In the HIV-1 section we then further focused on CD4 T cells of primary samples from two patients on long term cART.*

6. p11 line 20- MDA experiment

I am not sure that PCIP-seq with MDA experiment is essential to include this study.

*We included this section as researchers working in the HIV-1 field were concerned that the amount of template DNA required for PCIP-seq could be excessive for precious samples.*

7. p13 line 11 "DNA extracted from Pap"

What does Pap mean?

*Pap means “Papanicolaou” test. We have added this in the text.*

8. p16 line 15-17

To tackle with the HIV-1 provirus that can serve as viral reservoir, we need to characterise intact provirus without mutation.

For the purpose, we don't have to analyse all HIV-1 provirus.

I mean the author can describe that PCIP-seq will be potent to detect intact HIV-1 proviruses, which should be evaluated to measure HIV-1 reservoir in the infected cells.

*We agree that it is not necessary to analyze all HIV-1 provirus although we think that by being able to examine the sequence of many defective HIV-1 proviruses we may gain some insights. Work by Siliciano’s group showed that defective proviral sequences can also have clinical implications, as a large proportion of these are still capable of producing viral protein detectable by CTLs. This contributes to chronic inflammation. In that regard, we feel it may be relevant to track defective proviruses. Citation: Ross et al 2017, Defective HIV-1 Proviruses Are Expressed and Can Be Recognized by Cytotoxic T Lymphocytes, which Shape the Proviral Landscape, Cell Host & Microbe 2017, 21, 494-506.*

*Regarding the viral reservoir, we agree that the main strength of PCIP-seq is its capacity to tackle intact proviruses. In the manuscript, we point out that we were able to capture two full length HIV-1 proviruses, underlining that this technique will simplify identifying such intact proviruses to measure the HIV-1 reservoir. In addition, the ability of PCIP-seq to link intact proviral sequences to the integration site has significant relevance given the emerging evidence for the presence of hotspots where intact proviruses are enriched.*

Reviewer 2:

The paper developed an interesting method called PCIP-seq based on the third generation sequencing for analyzing integration of viral genomes to host genomes. Taking advantage of long reads of the third generation sequencing, other than detecting integration sites, PCIP-seq also allows detailed investigation of viral genomes integrated in the host genomes and allows accurate detection of integration sites in repetitive regions. The proposed PCIP-seq method is potentially very useful for studying virus-host dynamics. However, the paper needs to be considerably improved before publication.

1. The resolutions of the main figures are very low (the supplementary figures are OK), making it very hard to read the main figures. The figure legends are not clear. How to understand Figure 2 b,d,e, Figure 3b, etc.? Circus plots in Figure 4 and 5 cannot provide much information. They can only roughly show integration sites. More detailed analyses of the integration sites are needed.

*Resolution: Unfortunately, the low resolution of the figures was caused by the PDF*

*generation system. The original files provided were high-resolution. We hope the*

*resolution of the updated figures will be satisfactory in the PDF generated by the*

*journal upon this new submission. We also provide high-resolution figures in TIFF*

*format and would recommend examining these original figures/files to avoid any*

*technical concern.*

*Figure clarity: We have updated Figures 1, 2 and 3 and modified the corresponding*

*figure legends to make them clearer. While we agree that the circus plots in Figures 4*

*and 5 cannot give extensive information about individual integration sites, they do give*

*an overview and provide information on which proviruses contain deletions, which we*

*feel is useful. To avoid overloading the figures, we provided Supplementary Dataset_1*

*(HIV-1) and Tables S5 & S6 (ERVs) in which the reader will find detailed information*

*regarding each individual integration site. In the case of HIV-1, the table reports*

*extensive information including technical sequencing-related data (coverage, shear*

*sites, …), information regarding the integrated provirus (intact, deletions,*

*hypermutations, …) and a description of the genomic region in the vicinity of the*

*integration site (gene(s), genomic features …). In the revised manuscript we have*

*added a reference to this Supplementary information in the legends of Figures 4 and 5.*

*We also have made the chromosome numbers clearer in both figures.*

2. More analysis of PCIP-seq data is needed to show the advantage of PCIP-seq. For example, what percentage of nanopore reads cover integrate sites? If a read covers an integration site, usually how many nucleotides are from the host genome and how many are from the viral genome (it is more helpful to show the length distributions)? The authors showed the percentage of Illumina integration sites also found in PCIP-seq, but what about the percentage of PCIP-seq integration sites also found in the Illumina data (to measure the specificity of the PCIP-seq method)?

*For the percentage of Nanopore reads covering the integration sites, this equates to*

*the “Chimeric reads %” shown in Table S8 in which we report the efficiency estimations*

*of PCIP-seq for BLV (ovine and bovine) and HIV-1 samples. These reads contain both*

*virus and host DNA and include the integration site. As can be seen in the table, in*

*most cases >50% of the reads cover the integration site. We have added information in*

*the description of Table S8 to clarify this.*

*For the number of nucleotides from the host genome captured at each integration site*

*we did not calculate this in BLV due to the large number of integrations but did do this*

*for HIV-1. In the case of BLV, additionally many proviral integrations in sheep fall in*

*“hotspots” (described in Rosewick et al. Nature Communications. 2017;8:15264.)*

*making it difficult to calculate. In the case of HIV-1, the number of nucleotides for the*

*provirus where coverage is >20 as well as the number of bases from the host that were*

*captured at each integration site were calculated. The numbers are indicated in*

*Supplementary dataset 1. This is also summarized in Table S8 (four last columns:*

*“Average and range of coverage at integration site”, “Base pairs of host DNA*

*sequenced”, and “Base pairs of provirus sequenced with coverage >20”). We have*

*also updated Figures 1 and 2 to show more clearly how PCIP-seq read coverage is*

*distributed across the provirus and in the host genome.*

*We have added the percentage of PCIP-seq integration sites also found in the Illumina*

*data in Table S1, columns “U-IS PCIP in ILL. (%)” and “U-IS PCIP in ILL. (%) (>3)”. As*

*the number of integration sites identified by Illumina is considerably lower than that*

*seen in PCIP-seq the % of Illumina integration sites identified in PCIP-seq is lower than*

*the fraction of PCIP-seq integration sites identified in Illumina.*

3. One big advantage of PCIP-seq over existing methods is that it allows investigation of proviral sequences in the host genome such as analyzing SNPs, indels in the proviral sequences. However, nano-pore sequencing has a very high sequencing error rate and SNP/indel analysis generally need a very high coverage to achieve reasonable sensitivity and specificity. It seems that for most integration sites, there are usually only a handful of supporting PCIP-seq reads, making SNP/indel analysis very difficult for the majority of the integration sites. The advantage of PCIP-seq over existing methods thus seems significantly jeopardized. More analysis should be provided in this respect.

*When we initially developed PCIP-seq, single read accuracy for Nanopore sequences was about ~90%. As a consequence, we were quite conservative in how we called variants, requiring >10 non-PCR duplicated reads. When PCR duplicates are included, the coverage in the proviruses used for SNP calling is much higher (for example in animal 233, the proviruses with >10 non-PCR duplicated reads had an average coverage of ~270, when all reads are counted.) The accuracy of single Nanopore reads has increased in the intervening time to ~95% (https://nanoporetech.com/aboutus/news/new-research-algorithms-yield-accuracy-gains-nanopore-sequencing). As a result, this cut off used is probably overly conservative. For example the artic network protocol (https://artic.network/ncov-2019) which is being used extensively for SARSCoV-2 whole genome sequencing uses 20 nanopore reads as the cut off for calling variants. Despite our conservative cut off, we still examined ~3000 proviruses for SNPs in these six samples. If we were to use the cut off of 20 reads used in the artic network this would increase to ~7000. Additionally, if one were to sequence the PCIP-seq library using PacBio HiFi reads, which have an accuracy of >99.8%, one could call variants in the majority of the provirus identified.*

*At the moment, the software for base calling and variant calling of Nanopore reads is evolving rapidly. As a consequence, if we were to redo this analysis using the latest base callers, variant callers etc, with less conservative cut offs, we would generate substantially more variants. Additionally, we could resequence the libraries with PacBio HiFi, which would further increase the numbers. Nevertheless, the message of the paper, namely that PCIP-seq can identify large numbers of de novo variants in proviruses and link them to specific integrations sites in a single experiment, would remain unchanged.*

*We have added this information to the discussion to address these points.*

*Final note to referees 1 & 2:*

*We have updated the main manuscript, Table 1, Figures 1 to 5 and all figure legends, as well as Additional File 1 (Supplementary information) according to the reviewers’ comments and the point-by-point response outlined above. All changes have been highlighted in the revised material.*

**Second round of review**

**Reviewer 1**

The authors revised manuscript according to reviewers' comment and suggestion. Revised figures are much clearer than original ones. I think the manuscript is ready to be accepted.

**Reviewer 2**

The authors did not do any further analysis according to my comments about the SNP/INDEL analysis. I would suggest the authors to more comprenhensively evalualte the SNP/INDEL calling (e.g. its sensitivity, specificity at different coverages), becasue this is really important for the application of this technology.

**Authors Response**

**Point-by-point responses to the reviewers’ comments:**

Reviewer #2: The authors did not do any further analysis according to my comments about the SNP/INDEL analysis. I would suggest the authors to more comprehensively evaluate the SNP/INDEL calling (e.g. its sensitivity, specificity at different coverages), because this is really important for the application of this technology.

*Once again we thank the reviewer for taking the time to review the manuscript We agree with the reviewer’s point that a more comprehensive evaluation of SNP calling is important for the broad application of this technology. However, estimating the impact of differing coverages on calling SNPs is hampered by the difficulty of distinguishing between true SNPs and false positives/negatives in the provirus as we adjust thresholds without extensive labour intensive validation efforts.*

*In order to address this point we carried out a new experiment taking the human HIV-1 sample subset as starting material. We performed Illumina sequencing of the two HIV1 patient PCIP-seq libraries in order to call SNPs in the host DNA flanking the integration site as proxy for the provirus itself. The shearing necessary to generate the Illumina library prevents us linking viral reads to a specific provirus/integration site, precluding a comparison of variants within the provirus using the two technologies and at different depths. We thus reasoned that instead we could compare variants found in the DNA flanking the integration site sequenced by both technologies. This strategy allowed us to generate a set of SNPs that we were confident are “true” (especially as the majority identified in the flanking DNA will be common SNPs) and see what effect down sampling has on the SNPs called.*

*We have added a section describing the results in the Supplementary data (Additional file 1, Supplementary Note 2, Effect of coverage on SNP calling, pages 38 & 39) and have modified the main manuscript accordingly (Discussion, page 15 with reference to Additional file 1, and Methods, page 25).*

*Briefly, in the Illumina data after filtering we found 157 SNPs in the host DNA flanking the provirus integrations. Importantly 90% of the SNPs called were common human SNPs giving us confidence in the calls. We then called SNPs in the Nanopore data for the same regions of the genome, identifying 142 SNPs. All these SNPs were also found in the Illumina data (no false positives compared to Illumina), indicating a ~10% false negative rate in the Nanopore data. Downsampling did not produce any false positives, instead the number of false negatives increased (13.4%, 15.3% and 19.1% in the ~100X, ~50X and ~20X downsampled data respectively).*
 *As a consequence, we can conclude that higher coverage helps reduce false negatives, but even when we have relatively modest coverage false positives are not an issue, instead we are more likely to get false negatives as the coverage goes down.*

*As regards short INDELS we did not examine them in this work (we do look at larger insertions and deletions) as we felt that the high error rate around homopolymers in R9.4 Nanopore data could generate excessive false positives. The newer R10.3 flow cells may be better suited to calling INDELS, especially given recent improvement in base calling, alternatively PacBio Hi-Fi reads could also be used.*

*We have updated the main manuscript and the Additional File 1 (Supplementary information) according to the point-by-point response outlined above. All changes have been highlighted in the revised material.*

**Second round of review**

**Reviewer 2**

I have no further comments.
